# Rational Design and Greener Synthesis of Selenylated Indolamides as Potential Anti-Alzheimer’s Agents

**DOI:** 10.1021/acsomega.5c08265

**Published:** 2025-11-13

**Authors:** Angélica Justino Dias, Aldo Sena Oliveira, Angélica Ceci Silva, Antonio Luiz Braga

**Affiliations:** † Department of Chemistry, Center for Physical and Mathematical Sciences, Federal University of Santa Catarina, Florianópolis, Santa Catarina 88040-900, Brazil; ‡ Faculty of Medicine, University of Lisbon, Gulbenkian Institute for Molecular Medicine (GIMM), Lisbon 1649-028, Portugal

## Abstract

This study presents the rational design and sustainable synthesis of selenylated indolamides as potential therapeutic agents for Alzheimer’s disease. Through computational approaches, including molecular docking and pharmacokinetic analyses, we identified key structural modifications that improve acetylcholinesterase inhibition, a critical target for AD treatment. We employed an environmentally benign I_2_/DMSO oxidation system to optimize the synthetic protocol, enabling the efficient selenylation of 27 indolamide derivatives (**5a–6a**) via a straightforward and practical transformation, delivering high yields of up to 99%. Importantly, the methodology proved scalable, delivering an 88% yield on a gram-scale reaction. *In silico* ADMET predictions using the pkCSM platform indicated that C2-selenylated indolamides possess an improved safety profile and promising pharmacokinetic properties, suggesting their potential for further drug development. In particular, compounds **5a** and **5y** demonstrated the best balance between reaction yield and docking score (94% and 86.17; 98% and 93.71, respectively). These findings highlight the significance of incorporating green chemistry principles alongside advanced in silico methodologies to drive innovation in drug discovery.

## Graphical abstract

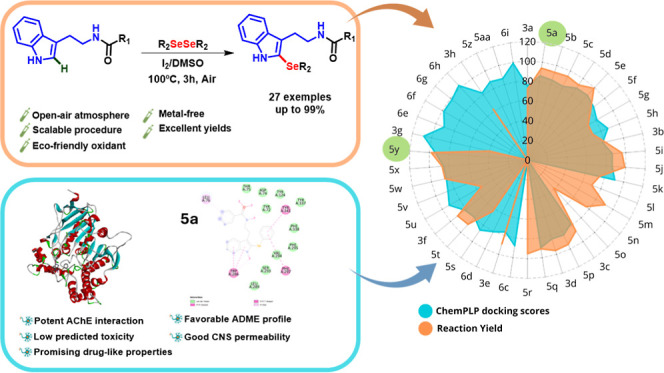

## Introduction

Alzheimer’s disease (AD) is a progressive neurodegenerative disorder and one of the leading causes of dementia worldwide. It is estimated that more than 50 million people currently live with AD, a number projected to increase dramatically with the aging population, thereby placing a heavy burden on patients, families, and healthcare systems.^1–4^ At the biochemical level, AD pathology is associated not only with the extracellular deposition of amyloid-β (Aβ) plaques and intracellular neurofibrillary tangles of hyperphosphorylated tau protein, but also with oxidative stress, neuroinflammation, mitochondrial dysfunction, and metabolic alterations that contribute to disease progression.^5–7^ Current therapeutic strategies focus mainly on symptomatic relief. Clinically approved drugs such as donepezil and galantamine act by inhibiting acetylcholinesterase (AChE), which increases acetylcholine availability in the synaptic cleft and enhances cholinergic neurotransmission (Figure 1A). While this approach temporarily improves cognitive function, it does not halt or reverse the underlying neurodegeneration, highlighting the urgent need for innovative therapies capable of addressing multiple disease mechanisms simultaneously.

**Figure 1:**
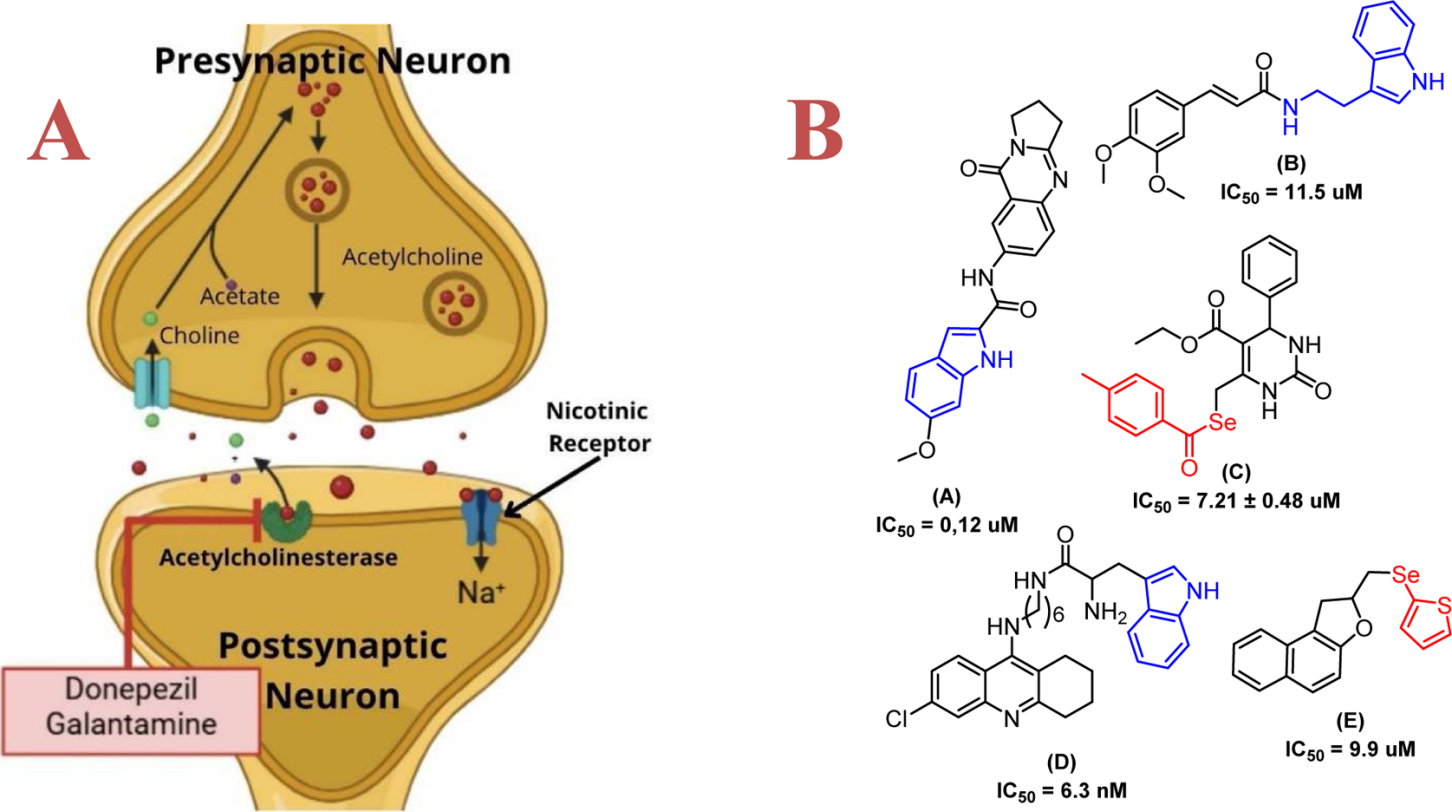
(A) Scheme of cholinergic system and ACh inhibition (B) and examples of AChE inhibitors.

Given the multifactorial nature of AD, the development of multitarget-directed ligands (MTDLs) has emerged as a promising strategy to modulate multiple pathological pathways in a single therapeutic agent. MTDLs offer the possibility of integrating antioxidant, anti-inflammatory, and neuroprotective actions with the ability to restore neurotransmitter balance. Within this context, two classes of compounds stand out as particularly promising scaffolds: indolamides and organoselenium derivatives.^8–12^ Both groups have exhibited pharmacological activities, including inhibition of AChE, antioxidant effects, and anti-inflammatory activity, which make them suitable candidates for designing novel multitarget therapies.^6,13–15^

Indolamides represent an important class of heterocycles with well-documented neuroprotective activities. Several indolamide derivatives have demonstrated inhibitory activity against AChE, underscoring their potential for symptomatic relief in AD (Figure 1B, molecules **A**, **B**, and **D**).^16^ Beyond cholinesterase inhibition, indolamides have also shown antioxidant, anti-inflammatory, and cytoprotective properties, broadening their therapeutic relevance.^6,8,12,13,17^ Their structural diversity and capacity to establish π-stacking and hydrogen-bonding interactions with biomolecular targets make them especially attractive for rational design of MTDLs. Furthermore, their synthetic versatility enables functional modifications that fine-tune pharmacological profiles and minimize off-target effects.

On the other hand, organoselenium compounds have gained increasing interest due to their unique redox properties and wide range of biological activities. Initially recognized as antioxidant mimics of glutathione peroxidase (GPx), organoselenium derivatives are now known for anticancer, antimicrobial, antiviral, antidiabetic, and anti-inflammatory effects.^18^ Diselenides, in particular, have been explored in asymmetric catalysis and in green oxidative protocols, reinforcing their dual role in medicinal and sustainable chemistry.^19^ High-throughput screenings further demonstrated that selenium derivatives display superior antifungal hit rates compared to nonselenium compounds, while the synthesis of selenium-containing sugars enhanced solubility and bioavailability, overcoming a common limitation in organoselenium pharmacology.^20,21^

From a therapeutic perspective, compounds such as ebselen and diphenyl diselenide (DPDSe) act as mixed-type AChE inhibitors, binding to both the catalytic active site (CAS) and the peripheral anionic site (PAS).^22–24^ Moreover recently developed derivatives have also demonstrated AChE inhibitory activity, thereby reinforcing the multitarget potential of this chemical family (Figure 1B, molecules **C** and **E**).^14,15,22,23,25^

The convergence of these two classes provides an exciting opportunity for the development of indole–selenium hybrids, which may combine and even potentiate the pharmacological properties of their parent scaffolds. Hybrid molecules can exhibit synergistic effects, leading not only to improved inhibition of AChE but also to complementary antioxidant and anti-inflammatory actions. This molecular hybridization strategy is increasingly recognized as a powerful tool for the design of next-generation drugs to combat multifactorial diseases such as AD.^26^

To support this rational design, computational medicinal chemistry tools have become indispensable. Platforms such as SwissADME and pkCSM allow the prediction of key pharmacokinetic and toxicological properties, including absorption, distribution, metabolism, excretion (ADME), and potential safety liabilities.^6,27–32^

Other computational approaches—such as docking, molecular dynamics, and in silico toxicology—provide insights into enzyme–ligand interactions and predict efficacy and off-target effects.^6,33,34^ By combining these predictive platforms, it is possible to optimize candidate molecules before synthesis, thereby reducing costs, accelerating development timelines, and improving the likelihood of clinical success.

In parallel, integrating green chemistry principles into drug discovery is essential for advancing more sustainable practices in Medicinal Chemistry and Organic Synthesis. Employing environmentally benign oxidants, recyclable catalysts, and less toxic reagents reduces the ecological impact of synthetic processes, while still delivering molecules with high therapeutic potential.^35–37^ Our research group has contributed to this area by developing greener selenylation protocols using safer oxidants, and by applying computational technologies to guide the synthesis of targeted indole–selenium hybrids.^15,38–43^

In summary, the combination of indolamide and organoselenium scaffolds, guided by computational medicinal chemistry and implemented through sustainable synthetic methodologies, represents a promising avenue for the development of novel MTDLs against Alzheimer’s disease. This integrated approach not only aligns with the urgent clinical need for more effective and safer therapies but also with the global imperative of advancing environmentally responsible pharmaceutical innovation.

## Results and Discussion

### Synthetic Procedure

Following the outlined synthetic strategy, eight indolamide derivatives (**3a–3h**) were successfully obtained using the described procedure, affording good yields ranging from 40% to 96% (Figure 2). Among these, compounds **3e** and **3g** are novel and have not been previously reported in the literature.^44^ The biological relevance of these compounds has been highlighted in several studies, particularly for their antioxidant, antimicrobial, and acetyl/butyrylcholinesterase inhibitory activities.^45–47^

**Figure 2:**
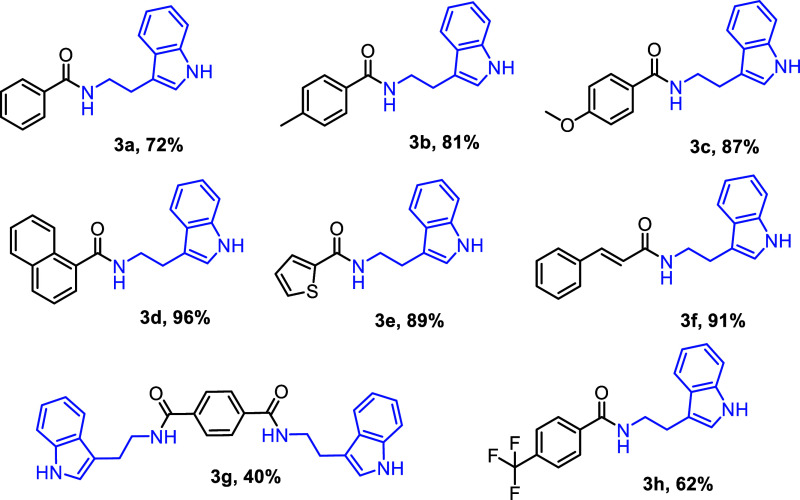
Indolamides obtained using adapted methodology.

To initiate our study, the reaction between compound **3a** and diphenyl diselenide (**4a**) under an iodine/DMSO oxidative system was selected as a model to optimize the selenylation protocol. A mixture of diphenyl diselenide (0.1 mmol) and iodine (1.0 equiv., 0.2 mmol) was added to DMSO (1.3 mL) and stirred for 15 min at room temperature. Subsequently, compound **3a** (0.2 mmol) was added to the reaction mixture, and then heated to 100 °C under open-air conditions and maintained for 3 h. Under these conditions, the desired product **5a** was obtained in excellent yield (94%, Table 1, entry 1).

**Table 1:**
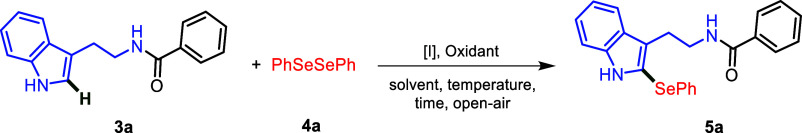
Optimization of the Reaction Conditions^a^ ^c^

entry	[I] (equiv)	oxidant	solvent	temperature	time	yield (%)
1	I_2_ (1.0)	DMSO	-	100 °C	3 h	94
2	I_2_ (1.0)	DMSO	-	100 °C	30 min	79
3	I_2_ (1.0)	DMSO	-	100 °C	1 h	80
4	I_2_ (1.0)	DMSO	-	100 °C	2 h	83
**5**	**I**_**2**_ **(0.75)**	**DMSO**	-	**100 °C**	**3 h**	**94**
6	I_2_ (0.5)	DMSO	-	100 °C	3 h	88
7	I_2_ (0.24)	DMSO	-	100 °C	3 h	54
8^b^	I_2_ (0.75)	DMSO	-	100 °C	1 h	80
9	I_2_ (0.75)	DMSO	-	r.t.	3 h	29
10	I_2_ (0.75)	DMSO	-	60 °C	3 h	36
11	TBAI (0.5)	DMSO	-	100 °C	3 h	60
12	KI (0.5)	DMSO	-	100 °C	3 h	N.R.
13	I_2_ (0.75)	oxone	DMSO	100 °C	3 h	90
14	TBAI (0.5)	oxone	DMSO	r.t.	40 min	60
15	I_2_ (0.75)	-	cyrene	100 °C	3 h	N.R.
16	I_2_ (0.75)	-	CH_3_CN	80 °C	3 h	16
17	I_2_ (0.75)	-	DMF	100 °C	3 h	31

a General conditions: All the reactions were conducted with **3a** (0.2 mmol) **4a** (0.1 mmol), substochiometric amount, oxidant in 1.3 mL solvent.

b Argon atmosphere.

c Quenched by saturated Na_2_S_2_O_3_ solution before purification.

To evaluate the time-dependence, additional experiments were performed by reducing the reaction duration to 30 min, 1 h, and 2 h (Table 1, entries 2–4). These experiments demonstrated that a 3 h reaction time was optimal, providing the highest yield of compounds **5a** under the established conditions (Table 1, entry 1). Next, the influence of iodine loading on the reaction outcome was evaluated. The amount of iodine was reduced to 0.75, 0.5, and 0.24 equiv, relative to **3a**. A significant decrease in yield was observed when iodine was used in amounts below 0.3 equiv. These results suggest that, due to the lower reactivity of the C2 position compared to C3 in the indole ring of compounds **3a**, a substoichiometric amount of iodine is required for efficient selenylation distinct from the typical behavior observed in reactions targeting the more reactive C3 position of the indole derivatives, as described by us.^40^ To evaluate the influence of the reaction atmosphere, the standard conditions were also carried out under argon, which resulted in a slight decrease in yield, suggesting that an oxidizing atmosphere favors the transformation (entry 8).

To assess the effect of temperature, the reaction was conducted at room temperature and at 60 °C (Table 1, entries 9 and 10). In both cases, a significant drop in yield was observed, confirming that elevated temperature (100 °C) is essential for optimal conversion. Subsequently, the iodine source was varied to tetrabutylammonium iodide (TBAI) and potassium iodide (KI), but no improvement in yield was observed (Table 1, entries 11 and 12).

Alternative oxidative systems, including I_2_/oxone and TBAI/oxone—both reported in related transformations^48^ — were also tested (Table 1, entries 13 and 14). While the I_2_/oxone system produced yields comparable to the optimized conditions, the TBAI/oxone system was less effective, confirming that the original I_2_/DMSO protocol remains the most reliable and efficient.

Notably, the I_2_/DMSO system proved to be the most effective, with the conditions of entry 5 (0.75 equiv, corresponding to 0.15 mmol of I_2_) provided the best overall performance. Finally, solvent screening was performed, but none of the tested alternatives improved the reaction outcome (Table 1, entries 15–17).

Interestingly, the very low yields obtained in entries 16 and 17 (CH_3_CN and DMF) provide additional insight into the reaction mechanism. Under these conditions, the oxidative pathway was not efficiently, which suggests that the DMSO is essential not only as a solvent but also as a co-oxidant that stabilizes ionic intermediates. This observation supports the predominance of the ionic pathway over the radical one, in agreement with the results obtained from the mechanistic control experiments (Schemes 2 and 3).

A detailed analysis of the results revealed that the reaction outcome is significantly influenced by electronic effects on the aromatic ring of the indolamide, rather than on the diselenide moiety, contrary to initial expectations. For instance, compound **5m**, bearing a methyl substituent on the indolamide and a perfluorophenylselanyl group on the diselenide, afforded a high yield (91%), while compounds **5s** and **5u**, which contain electron-withdrawing groups on the indolamide ring, yielded only 63% and 54%, respectively (Table 2).

**Table 2:**
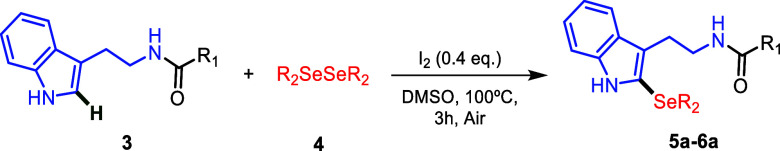

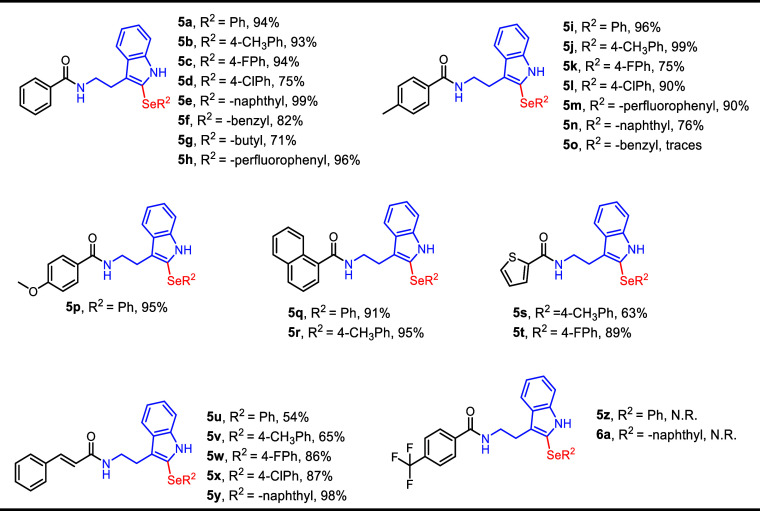
Scope for the Reaction of Indolamides with Diselenides

This trend can be rationalized by considering the proposed mechanism (Scheme 3). The iodination of the indole ring at C2 (**A**) is favored when electron-donating substituents increase the electron density, thereby facilitating electrophilic attack by iodine. In contrast, strong electron-withdrawing groups markedly reduce the nucleophilicity of the indole ring and destabilize the cationic intermediate, which explains the poor yields observed for substrates such as **5s** and **5u**, and the complete suppression of reactivity in compounds **5z** and **6a** bearing −CF_3_ substituents. These observations substantiate the mechanistic proposal that electronic effects on the indolamide ring play a decisive role in controlling reactivity.

Despite these electronic limitations, the methodology proved to be robust, delivering moderate to excellent yields (ranging from 39% to 99%), including for substrates with aliphatic diselenides, as exemplified by the preparation of the compound **5g** (71% yield). In total, 27 selenylated indolamide derivatives were successfully synthesized under the optimized conditions (Table 2).

### Control Experiments

To further demonstrate the efficiency and scalability of the developed methodology, the synthesis of compound **5a** was carried out on a multigram scale. The reaction of indolamide **3a** (5 mmol) under the optimized conditions afforded **5a** in an excellent 88% yield (Scheme 1, eq 1). Achieving high yields on a larger scale is particularly important given the potential pharmaceutical relevance of the target compounds.

**Scheme 1:**
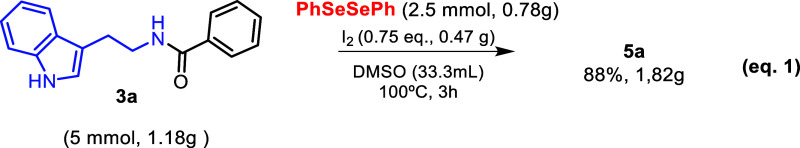
Gram-Scale Reaction

In the context of drug development, maintaining reaction efficiency and reproducibility at scale is crucial for industrial applicability. Scalable synthetic methods not only reduce production costs but also enhance sustainability by minimizing reagent consumption and waste generation. These factors are essential for enabling the transition from laboratory-scale synthesis to industrial manufacturing, while ensuring quality, consistency, and regulatory compliance in the pharmaceutical sector.

To investigate the possible reaction pathway, ten experiments were performed (Scheme 2). In the first part of the control experiments, the presence of radical-trapping reagents under optimized conditions was examined. Three different radical-trapping reagents: butylated hydroxytoluene (BHT), 2,2,6,6-tetramethyl-1-piperidinyloxy (TEMPO), and hydroquinone, were individually added 1 equiv under standard conditions (eq 2), affording **5a** in yields of 65%, 31%, and 85%, respectively. These results suggest that a radical pathway plays a minor role in this transformation. Next, the reaction **was conducted using phenylselenyl bromide instead of diphenyl diselenide** to probe whether a radical pathway could contribute to the formation of the electrophilic organoselenium species (Scheme 2, eq 3).

**Scheme 2:**
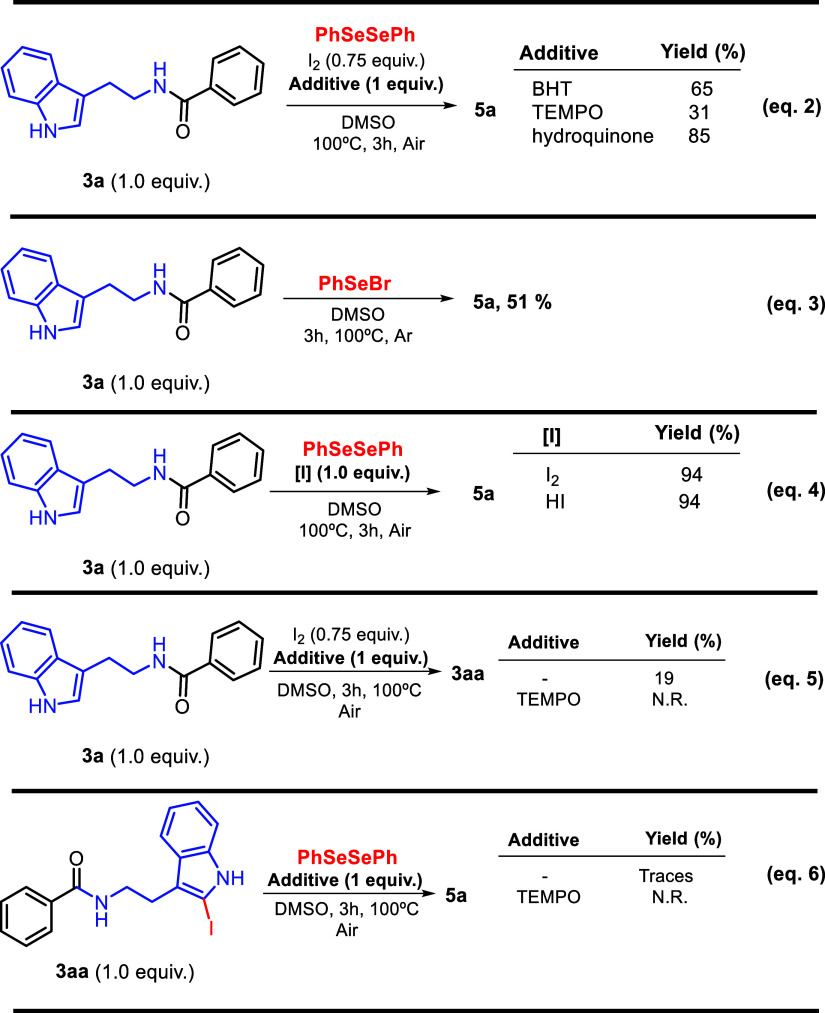
Control Experiments to Support the Proposed Mechanism

This idea is supported by previous reports, since some iodine/DMSO oxidation systems proceed through the phenylselenyl radical species **B** (Scheme 3).^39,40^ In our system, a 51% yield was obtained, suggesting the possible involvement of such intermediates (Scheme 3, eq 3). To probe the ionic contribution, the reaction was repeated with 1.0 equiv of hydroiodic acid under standard conditions (Scheme 3, eq 4), which afforded excellent yields, indicating that the mechanism is predominantly ionic.

**Scheme 3:**
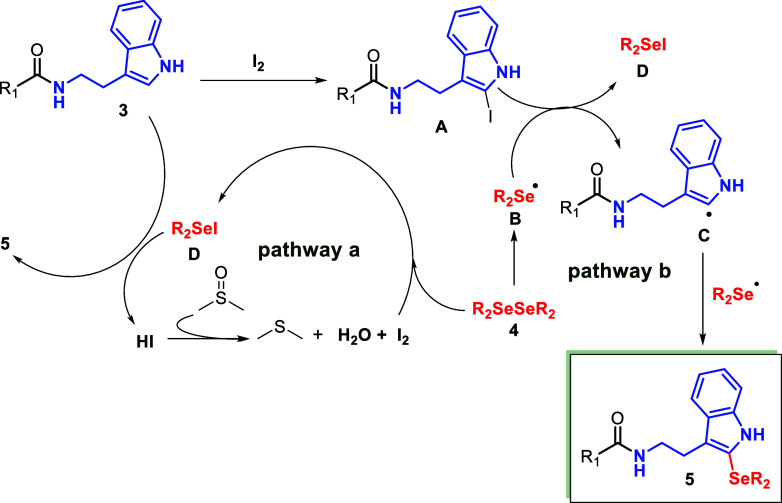
Proposed Reaction Mechanism of Regioselective Selenylation of Indolamides

To determine where radical processes may intervene, the experiments in eqs 5 and 6 were performed. These results suggest that radical steps are likely associated with the formation of the iodinated precursor (Scheme 2). Although literature commonly describes I_2_/DMSO-mediated chalcogenations as proceeding mainly through ionic pathways,^39,40,49,50^ our control experiments indicate that, for the C2-selenylation of indolamides, radical species also contribute. Partial inhibition in the presence of radical scavengers, and the complete suppression of product formation when iodinated indolamide **3aa** was subjected to reaction in the presence of a radical scavenger (Scheme 2, eq 6), reinforce the role of radical intermediates. This divergence can be attributed to the intrinsic reactivity of the indole C2 position, which may favor parallel radical pathways alongside the dominant ionic process. Therefore, we propose a hybrid mechanism: predominantly ionic, but with participation of radical species under the present conditions.

Based on these findings and consistent with previous reports, a plausible mechanistic pathway for the C2-selenylation of indolamides is outlined in Scheme 3. Pathway b involves iodination of indolamide **3** to intermediate **A** in the presence of I_2_/DMSO, as corroborated by the control experiment confirmed that the iodinated intermediate could be evolved to the selenylated product under optimized conditions.^51^ In this pathway, diphenyl diselenide **4** is activated to form the organoselenyl radical (**B**), which reacts with intermediate **A** to generate the indolyl radical **C** and organoselenyl iodide (**D**). Radical **C** couples with another **B** to afford the selenylated product **5**, while **D** can also react with **3** to furnish **5** and HI, latter is reoxidized to I_2_ by DMSO, closing the cycle of pathway a. Also, upon treatment of the diorganyl diselenide with I_2_, an electrophilic species of the type R_2_SeI (**D**) is likely generated. This reactive intermediate subsequently undergoes substitution at the 2-position of indole, yielding the corresponding 2-selenylindole **5** along with HI as a byproduct, that is regenerated by reaction with DMSO.

Although direct evidence of iodine radical species was not detected, prior studies on I_2_/DMSO systems suggest that such transient intermediates contribute to diselenide activation and generation of electrophilic selenium species.^39,40,50^ Consistently, radical-trapping experiments only partially reduced the yield, supporting the view that radical pathways operate as secondary contributors, while the ionic mechanism remains predominant (pathway a).

### In Silico Assays

#### Molecular Docking

All scoring functions available in the GOLD 2022.3.0 software suite (CCDC) were initially tested, including GoldScore, ChemScore, ASP, and ChemPLP. Among these, the ChemPLP function yielded the lowest RMSD in the redocking validation and was thus selected for all subsequent analyses.^52,53^ The docking protocol was validated by redocking the cocrystallized ligand with human acetylcholinesterase (AChE, PDB: 4M0E) using the ChemPLP scoring function. The RMSD obtained was only 0.002 Å, demonstrating agreement with the experimental pose and confirming the reliability of the method (Figure 3).

**Figure 3:**
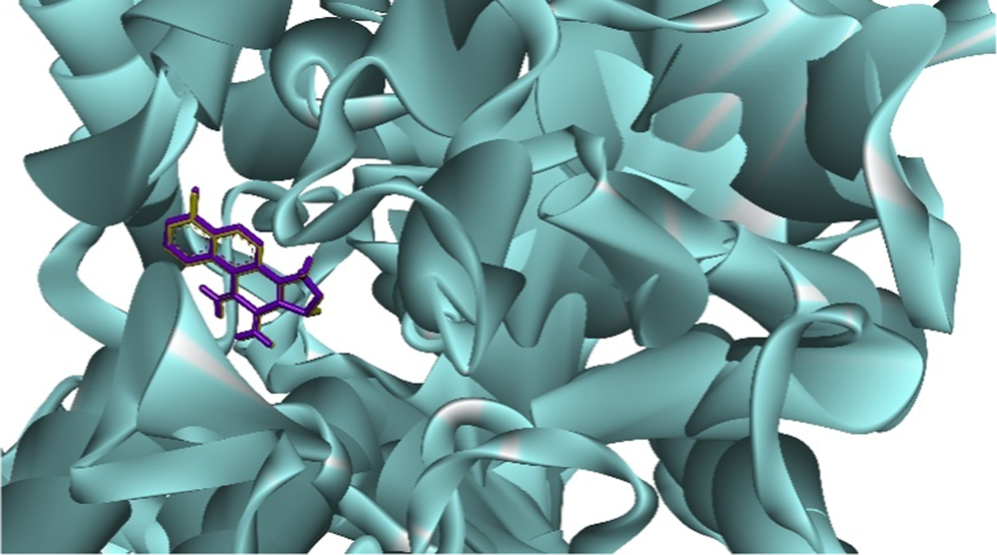
Redocking validation using ChemPLP scoring function in Discovery Studio. The crystallographic ligand (yellow) and the redocked pose (purple) show near-perfect overlap within the active site of the target protein (PDB ID: 4M0E). The exceptionally low RMSD value (0.002 Å) indicates high accuracy in reproducing the experimental binding mode, validating the reliability of the docking protocol for virtual screening and structure-based drug design (SBDD).

The ANOVA results confirmed that the differences in docking scores across compound groups were statistically significant (*p* < 0.05), as illustrated in Figure 4, reinforcing the role of specific substitutions in modulating receptor interactions. (*p* < 0.05), reinforcing the role of specific substitutions in modulating receptor interactions. These results indicate that structural modifications in selenylated derivatives directly impact binding affinity with AChE. Para-substitution proved to be the most favorable from both electronic and steric perspectives, optimally aligning the ligand in the active site and enhancing interactions with catalytic residues.

**Figure 4:**
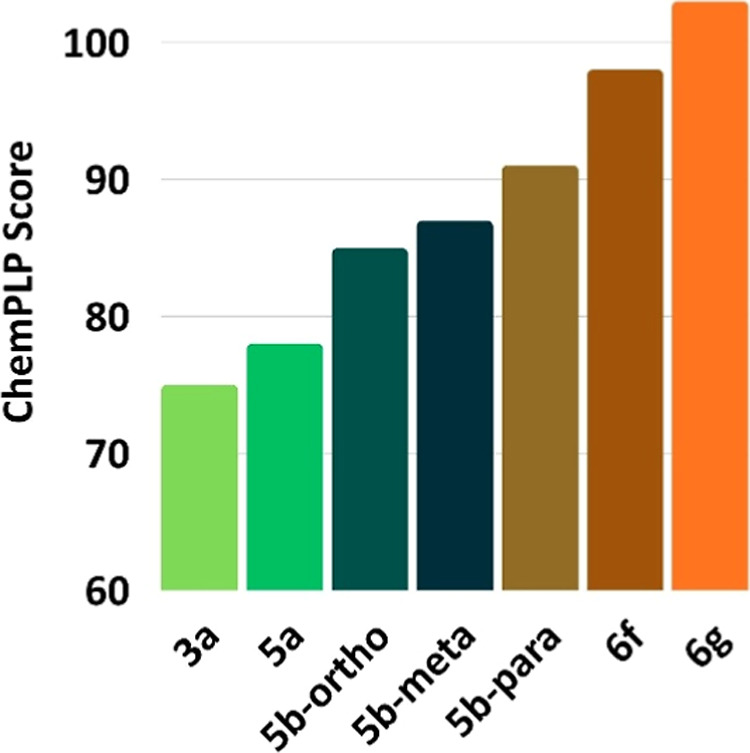
Boxplot showing the distribution of ChemPLP docking scores grouped by substitution type. The graph highlights statistically significant differences (ANOVA, *p* < 0.05) among nonselenylated, para-substituted, and selenylated derivatives, reinforcing the influence of chemical modifications on receptor affinity.

The comparison between compounds **3a** and **5a** (Figure 5) illustrates the direct impact of selenium incorporation on ligand–receptor interactions. The nonselenylated analogue **3a** displays moderate π–π interactions with residues such as **Tyr341**, **Trp286**, and **Phe297**, supported by van der Waals contacts. However, its overall interaction network is limited in spatial reach and intensity. In contrast, selenylated compound **5a** exhibits a more diverse and stronger interaction profile, including π-alkyl contacts with **Trp286**, **Val294**, and **Phe295**, in addition to π–π stacking. The presence of selenium appears to enhance molecular polarizability, allowing better alignment within the binding pocket and reinforcing interactions with critical residues like **Leu76** and **Tyr341**. These findings suggest that selenylation not only increases binding affinity—as reflected in the higher docking score of **5a**—but also expands the pharmacophoric envelope of the ligand, offering greater potential for stabilization within the AChE active site.

**Figure 5:**
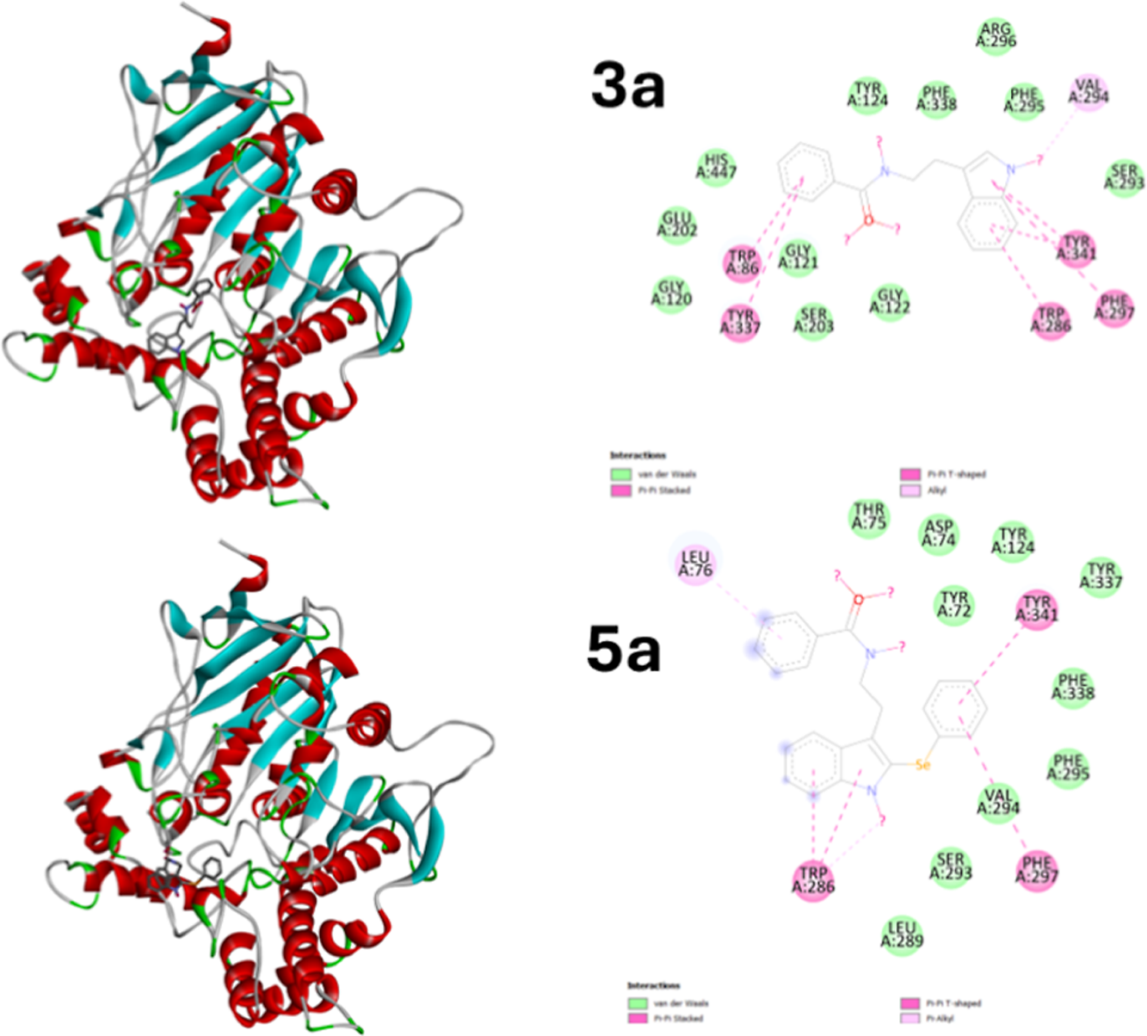
2D and 3D interaction diagrams of compounds **3a** (nonselenylated control) and **5a** (selenylated analogue) with AChE. While **3a** shows moderate π–π stacking and van der Waals interactions, **5a** engages in a broader interaction network, including π-alkyl contacts and enhanced stacking due to selenium incorporation. The improved interaction profile of **5a** highlights the structural and electronic contribution of selenium to binding affinity.

The interaction diagrams for the **5b**-ortho, **5b**-meta, and **5b**-para derivatives (Figure 6) reveal a clear positional effect of methyl substitution on binding affinity and interaction profile within the AChE active site. While all three variants maintain core π–π interactions with **Trp286** and **Tyr341**, the para-substituted analogue exhibits a richer and more strategically oriented interaction network.

**Figure 6:**
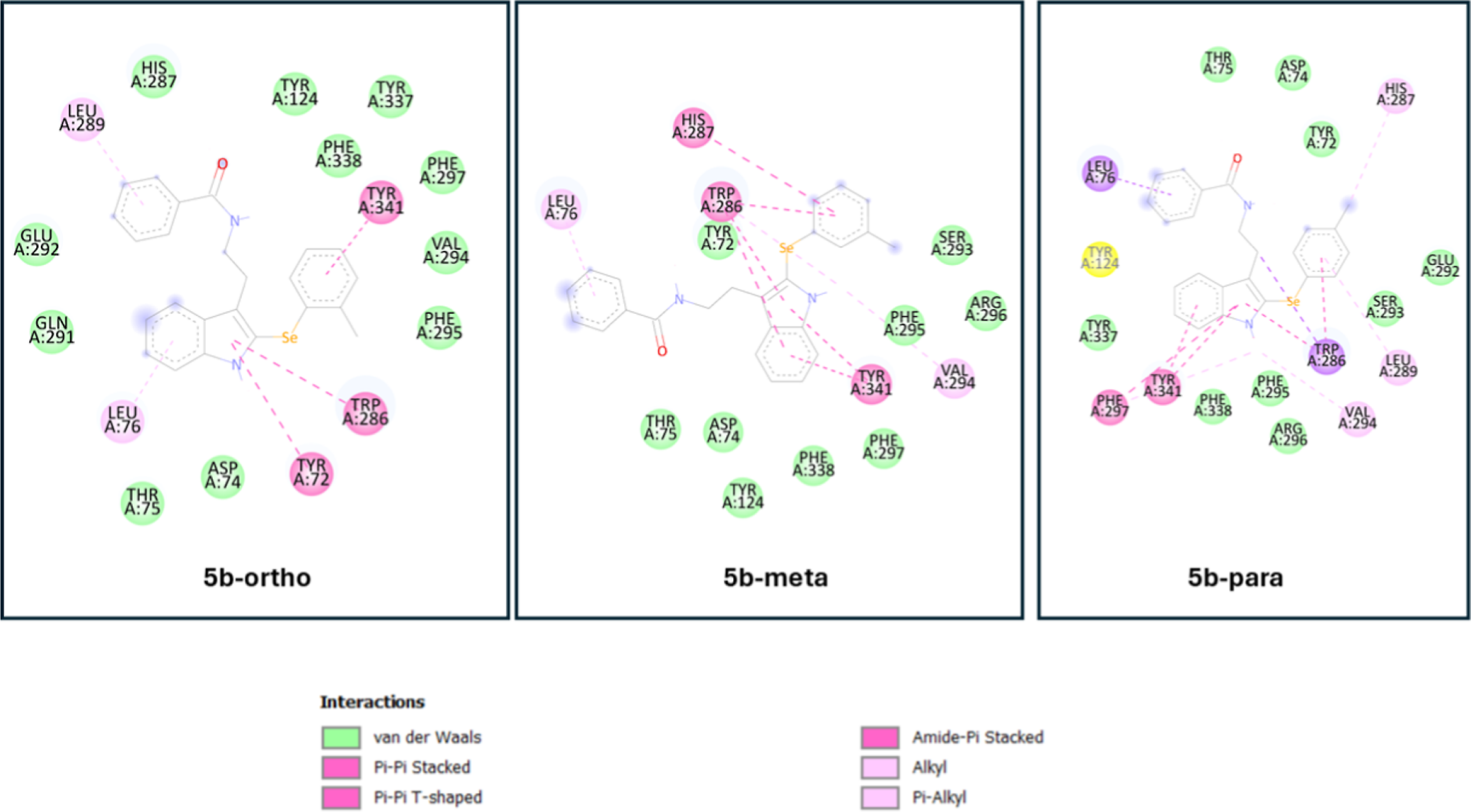
Comparative 2D interaction diagrams of the **5b**-ortho, **5b**-meta, and **5b**-para derivatives with AChE. While all compounds share core π–π interactions, only the para-substituted analogue (**5b**-para) establishes a key *T*-shaped interaction with the catalytic residue His287, along with enhanced π-alkyl and hydrophobic contacts. The interaction profile supports the superior binding affinity observed for **5b**-para and highlights the influence of methyl positioning on active site.

Notably, **5b-para** engages in a *T*-shaped π–π interaction with **His287**, a residue central to the catalytic triad, as well as hydrophobic and π-alkyl interactions with **Leu289**, **Val294**, and **Phe338**. In contrast, **5b**-ortho displays a more limited spatial alignment, with steric hindrance likely restricting its engagement with deeper active site residues. **5b**-meta, although slightly better aligned than ortho, still lacks the optimal orientation and electronic distribution seen in the para isomer. These findings demonstrate that para-substitution not only improves the ligand’s spatial orientation but also maximizes productive contacts, justifying its superior docking score and its selection as the lead compound.

To contextualize the in silico findings, we also performed docking simulations with the reference acetylcholinesterase inhibitors donepezil and galantamine, both of which are clinically employed in the management of Alzheimer’s disease. As expected, donepezil exhibited the strongest binding affinity to AChE, with a ChemPLP score of ∼120, consistent with its reported subnanomolar potency (IC_50_ = 6 nM). Galantamine showed an intermediate binding score (∼70), which aligns with its micromolar activity profile (IC_50_ = 17 μM).^54^ When compared with these standards, several of the synthesized selenylated indolamides (notably compounds **5a** and **5y**) reached ChemPLP values in the 85–95 range, surpassing galantamine and approaching the performance of donepezil. These results provide a robust comparative framework, further reinforcing the potential of the designed compounds as promising AChE inhibitors.

#### Binding Free Energy and Interaction Stability

To complement docking predictions, MM-GBSA calculations were performed using the Prime module of Schrödinger Suite 2022–3. Binding free energy (Δ*G*_bind) was calculated with the OPLS4 force field and VSGB solvent model. This refinement confirmed the stabilization trends observed in docking results.^55,56^ The binding free energy was used to estimate the binding free energy (Δ*G*_bind) for four representative compounds: **3a**, **5a**, **5b-para**, and **6g**. The calculated Δ*G*_bind values were −41.5 kcal/mol for **3a**, −52.7 kcal/mol for **5a**, −62.3 kcal/mol for **5b**-para, and −66.7 kcal/mol for **6g**. These results clearly reflect the progressive stabilization of the ligand–AChE complex as selenylation and substituent optimization are introduced.

The ∼11 kcal/mol improvement from **3a** to **5a** highlights the role of selenium in enhancing molecular polarizability and π–π/π-alkyl interactions. The further gain in **5b**-para confirms that para-substitution not only improves spatial orientation within the active site but also enables deeper insertion into the gorge, optimizing hydrophobic and electrostatic contacts. While **6g** exhibited the most favorable Δ*G*_bind, this came at the cost of poor solubility and limited CNS permeability, making it less suitable for therapeutic development despite its strong in vitro profile.

Notably, the ∼25 kcal/mol difference between **3a** and **6g** emphasizes how dense networks of noncovalent interactions—particularly π-stacking and van der Waals forces—can significantly enhance thermodynamic stability. However, when ADMET constraints are applied, this energetic advantage becomes secondary to factors like drug-likeness and bioavailability. In this context, **5b**-para represents the most promising balance between affinity and pharmacokinetic feasibility.

These results also underscore the value of MM-GBSA as a postdocking validation tool. Unlike docking scores, which rely on heuristic fit, MM-GBSA incorporates solvation and side-chain flexibility to deliver a more realistic estimate of binding stability. Its alignment with SAR trends and ADMET profiles strengthens confidence in the compound triage process and supports the selection of **5b**-para as a lead for in vivo evaluation.

#### ADMET Profiling

Pharmacokinetic properties were predicted using SwissADME, pkCSM, and ProTox-II. Parameters included gastrointestinal absorption, blood–brain barrier permeability, cytochrome P450 inhibition, hERG inhibition, solubility, Lipinski’s rule of five, CNS multiparameter optimization (MPO) score, and acute toxicity (oral LD_50_).^57–59^ ADMET screening was conducted on three representative compounds: the nonselenylated control (**3a**), a highly selenylated derivative (**6g**), and the optimized compound **5b**-para. Compound **3a** displayed excellent gastrointestinal absorption (92.17%) and moderate blood-brain barrier (BBB) permeability (logBB = 0.152). However, it showed potential for drug–drug interactions, acting as both a substrate and inhibitor of key cytochrome P450 enzymes (CYP1A2, CYP2C19, CYP2D6). While nonmutagenic (AMES negative), its predicted hepatotoxicity and chronic rat toxicity raise concerns for long-term use.

Compound **6g**, despite its top docking score (102.82), suffered from extremely poor solubility and negligible BBB permeability (logBB = −0.706), making it unsuitable for central nervous system (CNS) applications. It also exhibited potential cardiotoxicity (hERG II positive) and flagged concerns regarding liver toxicity. These limitations overshadow its in vitro affinity and suggest a need for structural optimization or redirection toward non-CNS indications.

Compound **5b**-para emerged as the most balanced candidate. It combined high GI absorption (96.17%) and favorable BBB penetration (logBB = 0.312) with compliance to Lipinski’s rule of five and absence of PAINS or REOS alerts. Although flagged for possible mutagenicity (AMES positive) and moderate hepatotoxicity, its favorable CNS multiparameter optimization (MPO) score of 4.7 underscores its potential for further development.

#### Toxicity and CNS-Targeting Filters

In addition to ADMET screening, targeted computational filters were employed to evaluate structural alerts, CNS drug-likeness, and predicted toxicity of the lead compound **5b**-para. These analyses aimed to ensure not only pharmacological efficacy but also chemical tractability and safety, which are critical in CNS drug development.

PAINS (pan-assay interference structures) and REOS (Rapid Elimination of Swill) filters, applied via SwissADME and FAF-Drugs4, confirmed that **5b**-para and the majority of high-scoring analogues lacked problematic substructures that could cause false positives or assay interference. This structural “clean profile” supports the reliability of their observed bioactivities and simplifies downstream hit-to-lead optimization.^60,61^

CNS drug-likeness was quantitatively assessed using the CNS multiparameter optimization (MPO) scoring system, which accounts for key physicochemical parameters such as cLogP, cLogD, MW, TPSA, p*K*_a_, and number of H-bond donors. **5b**-para achieved a MPO score of 4.7, surpassing the ideal CNS threshold (>4.0) and indicating a favorable balance between brain permeability and metabolic stability.^62^ In contrast, compounds like **6g**, despite superior binding energy, scored poorly in CNS MPO due to excessive lipophilicity and size, highlighting the limitations of affinity-based selection alone.

Toxicity profiles were estimated using pkCSM and ProTox-II platforms. **5b**-para was flagged as AMES-positive, suggesting potential mutagenicity, but exhibited no significant alerts for hERG I inhibition or acute hepatotoxicity. The compound showed moderate chronic liver toxicity risk but remained within acceptable thresholds for early phase development. Its predicted LD_50_ (rat, oral) was 2450 mg/kg, placing it in GHS toxicity class IV—considered low to moderate risk.

Together, these results support the selection of **5b**-para as a chemically viable and pharmacologically balanced lead. Its strong CNS MPO performance, structural alert clearance, and manageable toxicological predictions position it favorably for further validation, including in vivo efficacy and toxicity studies. Additional structural tuning may be warranted to address mutagenic potential while preserving its desirable CNS profile.

#### Computational–Experimental Integration

To bridge virtual screening with laboratory feasibility, a correlation analysis between synthetic yields and docking scores was performed for 27 selenylated derivatives. Compounds such as **5d** (-SePhCl), **5e** (-SeNaph), and **5g** (-SeBut) from series **3a**, as well as **5k** (series 3b), **5q** (series **3d**), and **5y** (series **3f**), exhibited both high synthetic yields (>70%) and docking scores above 87. This convergence underscores their potential as scalable drug prototypes.

In contrast, some high-performing in silico candidates from series **3g** and **3h** were not realized experimentally due to the unavailability of starting materials or synthetic challenges. These cases illustrate a key limitation of virtual-only approaches, reaffirming the need for holistic design frameworks that align predictive potency with synthetic accessibility.

The reaction conditions used to synthesize the compounds also align with green chemistry principles, employing a I_2_/DMSO system under open-air conditions, with in situ HI generation. These eco-friendly attributes enhance the industrial relevance of the method and make the compounds suitable for future scale-up.

In addition to docking validation and yield correlation, a structure–activity relationship (SAR) trend was observed across series **3** and **5**. Compounds bearing electron-donating substituents in the para-position (e.g., −CH_3_, −OMe) generally enhanced π-alkyl and hydrophobic interactions with key AChE residues, particularly **His287** and **Leu289**. Conversely, derivatives with electron-withdrawing groups on the indolamide ring, such as CF_3_ or halogens in meta/ortho positions, exhibited lower yields and weaker interactions due to steric hindrance or suboptimal alignment within the binding pocket. Notably, **5b**-para and **5k** combined electronic favorability and spatial complementarity, enabling high affinity and CNS-permeable profiles. Compounds from the **5q–5y** series, despite some having bulkier diselenide partners, maintained high yields and strong docking scores (>90), confirming that substitution on the amide side chain is more tolerant to structural variation compared to the indole core.

The in silico strategy proved effective in identifying high-affinity, synthetically accessible, and pharmacologically promising selenylated indolamides. Compound **5b**-para emerged as a strong lead candidate for further preclinical development due to its well-balanced docking, pharmacokinetic, and safety profile.

## Conclusions

This study presents a successful strategy for the rational design and greener synthesis of selenylated indolamides as potential AChE inhibitors for AD therapy. An environmentally benign iodine/DMSO oxidative system was efficiently implemented for the selenylation of 27 indolamide derivatives (**5a–6a**) via a straightforward and practical transformation, delivering high yields of up to 99%. Through an integrated approach combining structure-based design, sustainable chemistry, and comprehensive in silico profiling, we identified key structural features that enhance binding affinity, CNS permeability, and synthetic accessibility.

Selenylation at the C2 position of indolamides significantly improved ligand-AChE interactions, as confirmed by molecular docking, MM-GBSA binding energy, and structure–activity relationship (SAR) analyses. *P*-Substitution, particularly in **5b**-para, was critical for optimizing ligand alignment and interaction networks within the active site. Among the 27 synthesized compounds, **5b**-para emerged as a lead candidate, offering a strong balance between docking performance, pharmacokinetics, and safety.

In addition, compounds **5a** and **5y** demonstrated an outstanding combination of high synthetic yields (94% and 98%) and excellent docking scores (86.17 and 93.71, respectively). This dual advantage highlights their potencial as highly promising candidates for industrial applications, where scalability, efficiency, and biological performance are critical for drug development pipelines.

Pharmacokinetic predictions showed that **5b**-para met essential criteria for CNS-targeting drugs, including favorable blood–brain barrier permeability, high gastrointestinal absorption, and a CNS MPO score above 4.5. Despite a mutagenicity flag in AMES prediction, the compound remained within acceptable hepatotoxicity and cardiotoxicity thresholds, supporting its viability for further investigation. Importantly, its clean structural profile (free of PAINS and REOS alerts) and scalable synthesis using an environmentally benign I_2_/DMSO oxidation system align well with industry demands for sustainable and robust drug candidates.

Overall, the combination of greener synthetic methods and high-throughput computational screening proved effective for guiding compound selection and prioritization. This approach not only accelerated the discovery process but also minimized experimental costs and environmental impact. These findings provide a strong foundation for future in vivo validation of **5b**-para and related derivatives, with the potential to advance toward preclinical development. These results provide a strong foundation for future in vivo evaluation of **5b**-para, **5a**, and **5y**, with the potential progression toward preclinical development.

## Experimental Section

### General Procedure

To a mixture of diselenides (0.1 mmol), I_2_ (0.15 mmol, 0.019 g and DMSO (1.3 mL) was maintained for 15 min, after that the indolamide (0.2 mmol) was added. The reaction was stirred at 100 °C for 3 h (monitored by TLC). Saturated sodium thiosulfate (1 mL) was added to quench the reaction. The reaction was diluted with ethyl acetate (5 mL) and washed by H_2_O (20 mL). Aqueous phase was extracted with ethyl acetate (5 mL × 2). The organic layer was combined, ethyl acetate was removed by rotary evaporator and the crude product was purified by silica column chromatography (elute: ethyl acetate/hexane 30%) to afford the desired products **5a–5y**.

#### *N*-(2-(2-(Phenylselanyl)-1*H*-indol-3-yl)­ethyl)­benzamide (**5a**)

This compound was obtained as an orange solid (0.083 g, 94%); %); ^1^H NMR (400 MHz, DMSO-*d*_6_): δ 11.47 (s, 1H), 8.66 (t, *J* = 5.7 Hz, 1H), 7.85–7.80 (m, 2H), 7.71 (d, *J* = 7.9 Hz, 1H), 7.64–7.61 (m, 2H), 7.51–7.48 (m, 1H), 7.45 (d, *J* = 7.6 Hz, 1H), 7.35–7.31 (m, 3H), 7.24–7.19 (m, 3H), 7.16–7.12 (m, 1H), 7.04 (td, *J* = 7.5, 7.0, 1.0 Hz, 1H), 3.47–3.43 (m, 2H), 3.08 (dd, *J* = 8.8, 6.3 Hz, 2H). ^13^C NMR (101 MHz, DMSO): δ 166.4, 138.1, 134.7, 132.5, 131.2, 129.6, 129.2, 128.4, 127.3, 126.6, 122.7, 120.1, 119.1, 119.1, 118.5, 111.5, 40.7, 26.0.^77^Se NMR (76 MHz, DMSO-*d*_6_): δ 248.6; HRMS (ESI) calcd for C_23_H_20_N_2_OSe [M – H]^−^ *m*/*z*, 419.0703; found, 419.0664.

#### *N*-(2-(2-(*p*-Tolylselanyl)-1*H*-indol-3-yl)­ethyl)­benzamide (**5b**)

This compound was obtained as an orange solid (0.081 g, 93%); ^1^H NMR (400 MHz, DMSO-*d*_6_): δ 11.41 (s, 1H), 8.65 (t, *J* = 5.6 Hz, 1H), 7.85–7.80 (m, 2H), 7.70 (d, *J* = 7.9 Hz, 1H), 7.54–7.47 (m, 1H), 7.47–7.41 (m, 2H), 7.33 (d, *J* = 8.1 Hz, 1H), 7.14 (td, *J* = 7.9, 1.6 Hz, 4H), 7.04 (dd, *J* = 8.2, 2.3 Hz, 3H), 3.48–3.42 (m, 2H), 3.08 (dd, *J* = 8.8, 6.2 Hz, 2H), 2.20 (s, 3H).·^13^C NMR (101 MHz, DMSO): δ 166.1, 137.9, 135.9, 134.6, 131.0, 130.1, 129.5, 128.4, 128.2, 127.1, 122.4, 119.6, 118.9, 118.8, 111.3, 40.5, 25.9, 20.5. ^77^Se NMR (76 MHz, DMSO-*d*_6_): δ 242.9. HRMS (ESI) calcd for C_24_H_22_N_2_OSe [M – H]^−^ *m*/*z*, 433.0821; found, 433.0820.

#### *N*-(2-(2-((4-Fluorophenyl)­selenyl)-1*H*-indol-3-yl)­ethyl)­benzamide (**5c**)

This compound was obtained as an orange solid (0.082 g, 94%); ^1^H NMR (400 MHz, Acetonitrile-*d*_3_): δ 9.56 (s, 1H), 7.58 (ddt, *J* = 14.5, 8.0, 1.1 Hz, 3H), 7.38–7.32 (m, 1H), 7.29–7.22 (m, 3H), 7.16–7.09 (m, 3H), 7.04 (ddd, *J* = 8.2, 7.0, 1.2 Hz, 1H), 6.93 (ddd, *J* = 8.0, 7.1, 1.0 Hz, 1H), 6.81–6.74 (m, 2H), 3.51–3.44 (m, 2H), 3.05 (t, *J* = 7.1 Hz, 2H). ^13^C NMR (101 MHz, Acetonitrile-*d*_3_): δ 168.0, 162.9 (d, *J* = 244.1 Hz), 139.1, 135.9, 133.2 (d, *J* = 7.9 Hz), 132.1, 129.3, 128.6, 128.0, 127.7, 127.7, 123.9, 121.1, 120.5, 120.4, 120.1, 117.3 (d, *J* = 22.0 Hz), 112.1, 41.4, 26.8. ^77^Se NMR (76 MHz, DMSO-*d*_6_): δ 247.4. HRMS (ESI) calcd for C_23_H_19_FN_2_OSe: [M + Na]^+^ *m*/*z*, 437.0569; found, 437.0568.

#### *N*-(2-(2-((4-Chlorophenyl)­selanyl)-1*H*-indol-3-yl)­ethyl)­benzamide (**5d**)

This compound was obtained as an orange solid (0.097 g, 92%). ^1^H NMR (400 MHz, Acetonitrile-*d*_3_): δ 9.51 (s, 1H), 7.69 (ddd, *J* = 11.7, 8.2, 1.3 Hz, 3H), 7.51–7.45 (m, 1H), 7.42–7.34 (m, 3H), 7.21–7.05 (m, 7H), 3.60–3.54 (m, 2H), 3.14 (t, *J* = 7.0 Hz, 2H). ^13^C NMR (101 MHz, CD_3_CN): δ 167.8, 139.2, 135.9, 133.0, 132.1, 132.0, 131.9, 130.3, 129.3, 128.6, 127.9, 124.0, 121.7, 120.5, 120.1, 119.6, 112.1, 41.3, 26.8. ^77^Se NMR (76 MHz, DMSO): δ 251.7. HRMS (ESI) calcd for C_23_H_19_ClN_2_OSe: [M + Na]^+^ *m*/*z*, 477.0247; found, 477.0224.

#### *N*-(2-(2-(Naphthalen-1-ylselanyl)-1*H*-indol-3-yl)­ethyl)­benzamide (**5e**)

This compound was obtained as an orange solid (0.093 g, 99%). ^1^H NMR (400 MHz, Acetonitrile-*d*_3_): δ 9.39 (s, 1H), 8.15–8.08 (m, 1H), 7.80 (dd, *J* = 7.6, 1.6 Hz, 1H), 7.67 (dt, *J* = 7.7, 1.1 Hz, 1H), 7.63–7.58 (m, 3H), 7.48 (m, 2H), 7.41–7.36 (m, 1H), 7.32–7.23 (m, 3H), 7.21–7.13 (m, 2H), 7.03 (m, 3H), 3.54–3.48 (m, 2H), 3.10 (t, *J* = 7.1 Hz, 2H). ^13^C NMR (101 MHz, CD_3_CN) δ 167.8, 139.3, 136.0, 135.0, 133.4, 132.0, 131.5, 130.1, 129.7, 129.3, 128.8, 128.6, 127.9, 127.8, 127.5, 127.3, 126.7, 123.8, 121.4, 120.4, 119.9, 119.6, 112.0, 41.4, 26.8. HRMS (ESI) calcd for C_27_H_22_N_2_OSe: [M + Na]^+^ *m*/*z*, 493.0797; found, 493.0787.

#### *N*-(2-(2-(Benzylselanyl)-1*H*-indol-3-yl)­ethyl)­benzamide (**5f**)

This compound was obtained as an orange solid (0.072 g, 82%). ^1^H NMR (400 MHz, Acetonitrile-*d*_3_): δ 9.22 (s, 1H), 7.64–7.60 (m, 2H), 7.51 (dd, *J* = 7.9, 1.0 Hz, 1H), 7.42–7.38 (m, 1H), 7.35–7.30 (m, 2H), 7.28–7.24 (m, 2H), 7.09–7.03 (m, 4H), 6.99–6.92 (m, 3H), 3.93 (s, 2H), 3.38–3.32 (m, 2H), 2.81 (t, *J* = 7.1 Hz, 2H). ^13^C NMR (101 MHz, CD_3_CN): δ 167.7, 140.3, 138.9, 136.0, 132.1, 129.6, 129.3, 129.3, 128.6, 128.1, 127.9, 127.7, 123.4, 121.2, 120.6, 120.1, 119.7, 111.8, 41.3, 33.4, 26.6. HRMS (ESI) calcd for C_24_H_22_N_2_OSe: [M + Na]^+^ *m*/*z*, 457.0797; found, 457.0796.

#### *N*-(2-(2-(Butylselanyl)-1*H*-indol-3-yl)­ethyl)­benzamide (**5g**)

This compound was obtained as an beige solid (0.056 g, 71%); ^1^H NMR (400 MHz, DMSO-*d*_6_): δ 11.21 (s, 1H), 8.67 (t, *J* = 5.8 Hz, 1H), 7.87 (dd, *J* = 8.1, 1.4 Hz, 2H), 7.62 (d, *J* = 7.9 Hz, 1H), 7.55–7.42 (m, 3H), 7.34–7.30 (m, 1H), 7.12–7.06 (m, 1H), 7.03–6.96 (m, 1H), 3.46 (q, 3H), 3.04 (dd, *J* = 8.8, 6.3 Hz, 2H), 2.84 (t, *J* = 7.3 Hz, 2H), 1.52 (p, *J* = 7.3 Hz, 2H), 1.33 (h, *J* = 7.3 Hz, 2H), 0.82 (t, *J* = 7.3 Hz, 3H). ^13^C (101 MHz, DMSO): δ 166.6, 138.1, 135.2, 131.5, 128.7, 127.8, 127.6, 122.1, 120.4, 119.1, 118.8, 118.4, 111.3, 41.0, 32.5, 28.8, 26.5, 22.6, 13.9. HRMS (ESI) calcd for C_21_H_24_N_2_OSe: [M – H]^−^ *m*/*z*, 399.0982; found, 399.0976.

#### *N*-(2-(2-((Perfluorophenyl)­selanyl)-1*H*-indol-3-yl)­ethyl)­benzamide (**5h**)

This compound was obtained as an orange solid (0.097 g, 96%). ^1^H NMR (400 MHz, DMSO-*d*_6_): δ 11.37 (s, 1H), 8.66 (t, *J* = 5.7 Hz, 1H), 7.84 (dt, *J* = 7.0, 1.5 Hz, 2H), 7.66 (d, *J* = 7.9 Hz, 1H), 7.55–7.42 (m, 3H), 7.31 (t, *J* = 8.3 Hz, 1H), 7.14 (ddd, *J* = 8.2, 6.9, 1.2 Hz, 1H), 7.02 (ddd, *J* = 8.0, 7.0, 1.0 Hz, 1H), 3.47–3.40 (m, 2H), 3.13 (dd, *J* = 8.4, 6.4 Hz, 2H). ^13^C NMR (101 MHz, DMSO): δ 166.0, 147.3, 144.9, 142.2, 139.6, 138.0, 137.7, 135.6, 134.5, 130.9, 128.1, 127.0, 126.7, 122.6, 119.6, 118.9, 118.9, 116.8, 111.3, 40.1, 25.8. ^77^Se NMR (76 MHz, DMSO): δ 125.0. HRMS (ESI) calcd for C_23_H_15_N_2_OSe: [M + Na]^+^ *m*/*z*, 533.0168; found, 533.0166.

#### 4-Methyl-*N*-(2-(2-(phenylselanyl)-1*H*-indol-3-yl)­ethyl)­benzamide (**5i**)

This compound was obtained as an orange solid (0.084 g, 96%). ^1^H NMR (400 MHz, DMSO-*d*_6_): δ 11.46 (s, 0H), 8.57 (t, *J* = 5.7 Hz, 0H), 7.73 (t, *J* = 8.3 Hz, 1H), 7.34 (d, *J* = 8.2 Hz, 0H), 7.28–7.11 (m, 2H), 7.05 (t, *J* = 7.5 Hz, 0H), 3.48–3.41 (m, 1H), 3.08 (dd, *J* = 8.8, 6.2 Hz, 1H), 2.34 (s, 1H). ^13^C NMR (101 MHz, DMSO): δ 166.0, 140.8, 137.9, 132.4, 131.9, 129.4, 128.9, 128.7, 127.16, 127.1, 126.3, 122.5, 120.0, 118.9, 118.9, 118.2, 111.3, 40.5, 25.9, 20.9. ^77^Se NMR (76 MHz, DMSO-*d*_6_): δ 248.7. HRMS (ESI) calcd for C_24_H_22_N_2_OSe: [M + Na]^+^ *m*/*z*, 457.0797; found, 457.0791.

#### 4-Methyl-*N*-(2-(2-(*p*-tolylselanyl)-1*H*-indol-3-yl)­ethyl)­benzamide (**5j**)

This compound was obtained as an orange solid (0.090 g, 99%). ^1^H NMR (400 MHz, Acetonitrile-*d*_3_): δ 9.45 (s, 1H), 7.61–7.55 (m, 1H), 7.54–7.48 (m, 2H), 7.25 (dd, *J* = 8.3, 0.9 Hz, 1H), 7.14–7.04 (m, 6H), 6.96 (m, 1H), 6.90 (d, *J* = 8.1 Hz, 2H), 3.51–3.44 (m, 2H), 3.04 (t, *J* = 7.1 Hz, 2H), 2.26 (s, 3H), 2.12 (s, 3H). ^13^C NMR (101 MHz, CD_3_CN): δ 167.8, 142.5, 139.1, 137.8, 133.1, 131.6, 131.2, 131.1, 131.1, 129.9, 129.1, 128.6, 128.0, 123.8, 120.9, 120.7, 120.4, 120.0, 112.0, 41.3, 26.8, 21.4, 21.0. ^77^Se NMR (76 MHz, DMSO): δ 243.8. HRMS (ESI) calcd for C_25_H_24_N_2_OSe: [M + Na]^+^ *m*/*z*, 471.0953; found, 471.0950.

#### *N*-(2-(2-((4-Fluorophenyl)­selanyl)-1*H*-indol-3-yl)­ethyl)-4-methylbenzamide (**5k**)

This compound was obtained as an orange solid (0.070 g, 75%). ^1^H NMR (400 MHz, Acetonitrile-*d*_3_): δ 9.44 (s, 1H), 7.61–7.57 (m, 1H), 7.53–7.46 (m, 2H), 7.27 (dd, *J* = 8.2, 1.0 Hz, 1H), 7.23–7.15 (m, 2H), 7.14–7.05 (m, 3H), 7.04–6.94 (m, 2H), 6.89–6.80 (m, 2H), 3.51–3.44 (m, 2H), 3.05 (t, *J* = 7.1 Hz, 2H), 2.26 (s, 3H). ^13^C NMR (101 MHz, DMSO): δ 167.8, 164.0, 161.5, 142.5, 139.1, 133.2, 133.2, 133.1, 129.9, 128.6, 127.9, 127.7, 127.6, 123.9, 121.1, 120.5, 120.4, 120.1, 117.4, 117.2, 112.1, 41.3, 26.8, 21.4. ^77^Se NMR (76 MHz, DMSO): δ 247.5. HRMS (ESI) calcd for C_24_H_20_FN_2_OSe [M – H]^+^ *m*/*z*, 451.0726; found, 451.0717.

#### *N*-(2-(5-((4-Chlorophenyl)­selanyl)-1*H*-indol-3-yl)­ethyl)-4-methylbenzamide (**5L**)

This compound was obtained as an orange solid (0.066 g, 70%). ^1^H NMR (400 MHz, Acetonitrile-*d*_3_): δ 9.52 (s, 1H), 7.64–7.56 (m, 1H), 7.49–7.45 (m, 2H), 7.30–7.26 (m, 1H), 7.10–6.98 (m, 9H), 3.47 (q, *J* = 6.8 Hz, 2H), 3.03 (t, *J* = 7.1 Hz, 2H), 2.25 (s, 3H). ^13^C NMR (101 MHz, CD_3_CN): δ 167.8, 142.6, 139.2, 133.1, 133.0, 131.9, 131.9, 130.2, 129.9, 128.6, 127.9, 124.0, 121.7, 120.5, 120.1, 119.6, 112.2, 41.3, 26.8, 21.4. ^77^Se NMR (76 MHz, DMSO): δ 251.8. HRMS (ESI) calcd for C_24_H_20_ClN_2_OSe [M – H]^+^ *m*/*z*, 467.0428; found, 467.0416.

#### 4-Methyl-*N*-(2-(2-((perfluorophenyl)­selanyl)-1*H*-indol-3-yl)­ethyl)­benzamide (**5m**)

This compound was obtained as an orange solid (0.093 g, 91%). ^1^H NMR (400 MHz, Acetonitrile-*d*_3_): δ 9.45 (s, 1H), 7.57 (dt, *J* = 8.0, 1.0 Hz, 1H), 7.53–7.49 (m, 2H), 7.27 (dt, *J* = 8.3, 1.0 Hz, 1H), 7.16–7.12 (m, 2H), 7.09 (ddd, *J* = 8.2, 7.1, 1.1 Hz, 1H), 6.97 (ddd, *J* = 8.1, 7.0, 1.0 Hz, 2H), 3.52–3.43 (m, 2H), 3.10 (t, *J* = 7.0 Hz, 2H), 2.28 (s, 3H). ^13^C NMR (101 MHz, CD_3_CN): δ 167.7, 142.6, 139.1, 133.1, 129.9, 128.2, 127.9, 124.3, 121.7, 120.6, 120.2, 112.1, 41.0, 26.8, 21.4. HRMS (ESI) calcd for C_24_H_17_F_5_N_2_OSe: [M + Na]^+^ *m*/*z*, 547.0325; found, 547.0332.

#### 4-Methyl-*N*-(2-(2-(naphthalen-1-ylselanyl)-1*H*-indol-3-yl)­ethyl)­benzamide (**5n**)

This compound was obtained as an orange solid (0.073 g, 76%). ^1^H NMR (400 MHz, Acetonitrile-*d*_3_): δ 9.39 (d, *J* = 11.1 Hz, 1H), 8.13–8.08 (m, 1H), 7.82–7.78 (m, 1H), 7.70–7.58 (m, 2H), 7.50–7.44 (m, 4H), 7.28–7.22 (m, 1H), 7.19–7.05 (m, 5H), 7.02–6.95 (m, 2H), 3.54–3.46 (m, 2H), 3.09 (t, *J* = 7.0 Hz, 2H), 2.25 (d, *J* = 4.1 Hz, 3H). ^13^C NMR (101 MHz, CD_3_CN): δ 167.8, 142.5, 139.3, 135.0, 133.4, 133.1, 131.6, 130.1, 129.9, 129.7, 128.8, 128.5, 127.9, 127.8, 127.5, 127.5, 127.3, 127.1, 126.7, 123.8, 121.5, 120.4, 119.9, 119.6, 112.0, 41.3, 26.9, 21.4. ^77^Se NMR (76 MHz, DMSO): δ 212.9. HRMS (ESI) calcd for C_28_H_24_N_2_OSe: [M + Na]^+^ *m*/*z*, 507.0953; found, 507.0954.

#### 4-Methoxy-*N*-(2-(2-(phenylselanyl)-1*H*-indol-3-yl)­ethyl)­benzamide (**5p**)

This compound was obtained as an orange solid (0.067 g, 95%). ^1^H NMR (400 MHz, Acetonitrile-*d*_3_): δ 9.47 (s, 1H), 7.75–7.61 (m, 3H), 7.36 (dt, *J* = 8.2, 1.0 Hz, 1H), 7.31–7.15 (m, 6H), 7.08 (m, 1H), 6.99 (s, 1H), 6.94–6.89 (m, 2H), 3.81 (s, 3H), 3.59–3.50 (m, 2H), 3.13 (dd, *J* = 8.8, 5.5 Hz, 2H). ^13^C NMR (101 MHz, CD_3_CN): δ 163.0, 139.1, 130.7, 130.4, 129.7, 128.6, 127.6, 127.1, 123.9, 121.4, 120.4, 120.1, 118.8, 114.5, 112.1, 56.1, 41.3, 26.9. ^77^Se NMR (76 MHz, DMSO-*d*_6_): δ 248.7. HRMS (ESI) calcd for C_24_H_22_N_2_O_2_Se: [M + Na]^+^ *m*/*z*, 473.0746; found, 473.0741.

#### *N*-(2-(2-(Phenylselanyl)-1*H*-indol-3-yl)­ethyl)-1-naphthamide (**5q**)

This compound was obtained as an beige solid (0.084 g, 91%). ^1^H NMR (400 MHz, DMSO-*d*_6_): δ 11.50 (s, 1H), 8.67 (t, *J* = 5.8 Hz, 1H), 8.20–8.14 (m, 1H), 8.01–7.93 (m, 2H), 7.76 (d, *J* = 7.9 Hz, 1H), 7.58–7.46 (m, 4H), 7.36 (d, *J* = 8.0 Hz, 1H), 7.30–7.13 (m, 6H), 7.05 (t, *J* = 7.5 Hz, 1H), 3.58–3.51 (m, 2H), 3.16 (t, 2H). ^13^C NMR (101 MHz, DMSO): δ 169.0, 138.4, 135.4, 133.6, 132.9, 130.2, 130.1, 130.0, 129.9, 129.8, 129.4, 128.6, 127.7, 127.0, 126.8, 126.6, 126.0, 125.6, 125.3, 123.0, 120.4, 119.5, 119.4, 118.8, 111.8, 40.8, 26.4. ^77^Se NMR (76 MHz, DMSO): δ 249.3. HRMS (ESI) calcd for C_27_H_22_N_2_OSe: [M + K]^+^ *m*/*z*, 509.0536; found, 509.0547.

#### *N*-(2-(2-(*p*-Tolylselanyl)-1*H*-indol-3-yl)­ethyl)-1-naphthamide (**5r**)

This compound was obtained as an orange solid (0.078 g, 95%). ^1^H NMR (400 MHz, Acetonitrile-*d*_3_): δ 9.39 (s, 1H), 8.05–7.99 (m, 1H), 7.82 (m, 2H), 7.64 (dd, *J* = 8.0, 1.0 Hz, 1H), 7.46–7.22 (m, 6H), 7.11–6.95 (m, 4H), 6.87 (d, *J* = 7.8 Hz, 2H), 3.60 (q, *J* = 6.8 Hz, 2H), 3.12 (q, *J* = 7.1 Hz, 2H), 2.08 (s, 3H). ^13^C NMR (101 MHz, CD_3_CN): δ 169.9, 139.1, 137.8, 136.0, 134.6, 133.6, 132.0, 131.5, 131.2, 131.1, 131.1, 131.0, 130.9, 129.1, 128.7, 127.6, 127.2, 126.6, 125.9, 125.8, 123.8, 120.8, 120.4, 120.0, 112.0, 41.2, 26.8, 21.0. HRMS (ESI) calcd for C_28_H_24_N_2_OSe: [M + Na]^+^ *m*/*z*, 507.0953; found, 507.0957.

#### *N*-(2-(2-(*p*-Tolylselanyl)-1*H*-indol-3-yl)­ethyl)­thiophene-2-carboxamide (**5s**)

This compound was obtained as an yellow solid (0.053 g, 63%). ^1^H NMR (400 MHz, Acetonitrile-*d*_3_): δ 9.43 (s, 1H), 7.58 (dd, *J* = 7.9, 1.0 Hz, 1H), 7.52–7.36 (m, 2H), 7.34–7.20 (m, 2H), 7.17–7.04 (m, 3H), 7.02–6.90 (m, 4H), 3.58–3.33 (m, 2H), 3.16–2.92 (m, 2H), 2.11 (s, 3H). ^13^C NMR (101 MHz, CD_3_CN): δ 162.5, 141.1, 139.0, 137.9, 131.6, 131.3, 131.2, 131.1, 131.0, 131.0, 128.7, 128.6, 128.5, 123.8, 120.8, 120.8, 120.4, 119.9, 112.0, 41.2, 26.9, 21.0. HRMS (ESI) calcd for C_22_H_20_N_2_OSSe: [M + Na]^+^ *m*/*z*, 463.0360; found, 463.0348.

#### *N*-(2-(2-((4-Fluorophenyl)­selanyl)-1*H*-indol-3-yl)­ethyl)­thiophene-2-carboxami de (**5t**)

This compound was obtained as an yellow solid (0.073 g, 89%). ^1^H NMR (400 MHz, Acetonitrile-*d*_3_): δ 9.37 (s, 1H), 7.59 (dd, *J* = 7.9, 1.0 Hz, 1H), 7.48–7.44 (m, 1H), 7.32–7.19 (m, 4H), 7.09 (ddd, *J* = 8.2, 7.0, 1.2 Hz, 1H), 7.02–6.95 (m, 3H), 6.91–6.83 (m, 2H), 3.46 (q, *J* = 6.7 Hz, 2H), 3.10–3.01 (m, 2H). ^13^C NMR (101 MHz, CD_3_CN): δ 164.1, 139.1, 133.4, 133.3, 131.0, 128.8, 128.7, 128.6, 128.5, 123.9, 121.0, 120.6, 120.5, 120.0, 119.8, 119.5, 117.4, 117.2, 112.1, 41.2, 26.9. HRMS (ESI) calcd for C_21_H_17_FN_2_OSSe: [M + Na]^+^ *m*/*z*, 467.0109; found, 467.0133.

#### *N*-(2-(2-(Phenylselanyl)-1*H*-indol-3-yl)­ethyl)­cinnamamide (**5u**)

This compound was obtained as an yellow solid (0.050 g, 54%). ^1^H NMR (400 MHz, Acetonitrile-*d*_3_): δ 9.41 (s, 1H), 7.61 (d, *J* = 7.9 Hz, 1H), 7.44–7.40 (m, 2H), 7.36 (d, *J* = 15.7 Hz, 1H), 7.30 (dd, *J* = 7.7, 2.5 Hz, 3H), 7.19–7.08 (m, 7H), 7.01 (td, *J* = 7.5, 6.9, 1.0 Hz, 1H), 6.28 (d, *J* = 15.7 Hz, 1H), 3.42 (q, *J* = 6.8 Hz, 2H), 3.01 (t, *J* = 7.0 Hz, 2H). ^13^C NMR (101 MHz, CD_3_CN): δ 166.3, 140.1, 139.2, 136.2, 133.3, 130.6, 130.4, 130.4, 129.8, 128.6, 128.5, 127.6, 123.9, 122.9, 121.3, 120.4, 120.2, 120.1, 112.1, 40.8, 26.9. ^77^Se NMR (76 MHz, DMSO): δ 249.3. HRMS (ESI) calcd for C_25_H_22_N_2_OSe: [M + Na]^+^ *m*/*z*, 469.0797; found, 469.0788.

#### *N*-(2-(2-(*p*-Tolylselanyl)-1*H*-indol-3-yl)­ethyl)­cinnamamide (**5v**)

This compound was obtained as an yellow solid (0.059 g, 65%). ^1^H NMR (400 MHz, Acetonitrile-*d*_3_): δ 9.39 (s, 1H), 7.59 (dq, *J* = 8.0, 0.9 Hz, 1H), 7.43–7.40 (m, 2H), 7.36 (d, *J* = 15.7 Hz, 1H), 7.31–7.26 (m, 4H), 7.12–7.07 (m, 3H), 7.00 (ddd, *J* = 8.0, 7.0, 1.1 Hz, 1H), 6.97–6.93 (m, 2H), 6.28 (d, *J* = 15.7 Hz, 1H), 3.45–3.40 (m, 2H), 3.01 (t, *J* = 7.0 Hz, 2H), 2.12 (s, 3H). ^13^C NMR (101 MHz, CD_3_CN): δ 166.3, 140.1, 139.1, 137.8, 136.2, 131.5, 131.1, 130.4, 129.8, 129.2, 128.6, 128.5, 123.8, 122.9, 120.8, 120.7, 120.4, 120.0, 112.0, 58.0, 40.8, 26.9, 21.0. ^77^Se NMR (76 MHz, DMSO): δ 243.8. HRMS (ESI) calcd for C_26_H_23_N_2_OSe: [M + Na]^+^ *m*/*z*, 459.0977; found, 459.0973.

#### *N*-(2-(2-((4-Fluorophenyl)­selanyl)-1*H*-indol-3-yl)­ethyl)­cinnamamide (**5w**)

This compound was obtained as an yellow solid (0.080 g, 86%) ^1^H NMR (400 MHz, Acetonitrile-*d*_3_): δ 9.54 (s, 1H), 7.58–7.55 (m, 1H), 7.41–7.33 (m, 3H), 7.30–7.24 (m, 4H), 7.19–7.15 (m, 2H), 7.07 (ddd, *J* = 8.2, 7.0, 1.2 Hz, 1H), 6.97 (ddd, *J* = 8.0, 7.0, 1.0 Hz, 1H), 6.86–6.79 (m, 2H), 6.30 (d, *J* = 15.8 Hz, 1H), 3.41 (q, *J* = 6.8 Hz, 2H), 3.00 (t, *J* = 7.0 Hz, 2H). ^13^C NMR (101 MHz, CD_3_CN): δ 166.5, 140.3, 139.1, 136.1, 133.2, 133.1, 130.4, 129.8, 128.6, 128.5, 123.9, 122.8, 121.0, 120.6, 120.4, 120.1, 117.4, 117.2, 112.1, 40.9, 26.9. ^77^Se NMR (76 MHz, DMSO): δ 248.3. HRMS (ESI) calcd for C_25_H_20_FN_2_OSe [M – H]^+^ *m*/*z*, 463.0726; found, 463.0732.

#### *N*-(2-(2-((4-Chlorophenyl)­selanyl)-1*H*-indol-3-yl)­ethyl)­cinnamamide (**5x**)

This compound was obtained as an yellow solid (0.083 g, 87%). ^1^H NMR (400 MHz, DMSO-*d*_6_): δ ^1^H NMR (400 MHz, Acetonitrile-*d*_3_) δ ^1^H NMR (400 MHz, Acetonitrile-*d*_3_) δ 9.47 (s, 1H), 7.60 (dd, *J* = 7.9, 1.0 Hz, 1H), 7.41–7.25 (m, 7H), 7.17–7.08 (m, 4H), 7.01 (ddd, *J* = 8.1, 7.0, 1.1 Hz, 1H), 6.26 (dd, *J* = 15.8, 1.3 Hz, 1H), 3.42 (q, *J* = 6.7 Hz, 2H), 2.99 (t, *J* = 6.9 Hz, 2H). ^13^C NMR (101 MHz, DMSO): δ 166.4, 140.2, 139.2, 136.2, 133.0, 132.4, 132.1, 131.9, 130.4, 130.3, 129.8, 128.6, 128.5, 124.1, 122.8, 121.6, 120.5, 120.1, 119.7, 112.1, 40.8, 26.9. ^77^Se NMR (76 MHz, DMSO): δ 252.7. HRMS (ESI) calcd for C_25_H_20_ClN_2_OSe [M – H]^+^ *m*/*z*, 479.0428; found, 479.0425.

#### *N*-(2-(2-(Naphthalen-1-ylselanyl)-1*H*-indol-3-yl)­ethyl)­cinnamamide (**5y**)

This compound was obtained as an yellow solid (0.097 g, 98%). ^1^H NMR (400 MHz, Acetonitrile-*d*_3_): δ 9.40 (d, *J* = 12.3 Hz, 1H), 8.14–8.08 (m, 1H), 7.77 (dd, *J* = 8.0, 1.5 Hz, 1H), 7.68–7.58 (m, 2H), 7.50–7.42 (m, 3H), 7.34 (dq, *J* = 4.3, 2.0 Hz, 3H), 7.29–7.24 (m, 4H), 7.20–7.16 (m, 2H), 7.11–7.05 (m, 1H), 7.00 (ddd, *J* = 8.0, 7.0, 1.1 Hz, 1H), 6.20 (dd, *J* = 15.6, 1.7 Hz, 1H), 3.43 (q, *J* = 6.7 Hz, 2H), 3.04 (t, *J* = 6.9 Hz, 2H). ^13^C NMR (101 MHz, DMSO): δ 166.3, 146.0, 140.1, 136.1, 135.0, 133.3, 131.8, 130.4, 129.8, 129.8, 129.7, 128.6, 128.5, 127.8, 127.5, 127.3, 126.6, 123.8, 122.8, 121.4, 120.4, 119.9, 119.6, 112.0, 40.8, 26.9. ^77^Se NMR (76 MHz, DMSO): δ 213.4. HRMS (ESI) calcd for C_29_H_23_N_2_OSe [M – H]^+^ *m*/*z*, 495.0977; found, 495.0969.

## Supplementary Material



## Data Availability

All data supporting the findings of this study are available within the article and the Supporting Information file. Spectroscopic and chromatographic data for all synthesized compounds, as well as computational results, are fully provided.

## Notes

It includes the complete computational methods, general experimental information and procedures, characterization data for all products and NMR spectra of the synthesized compounds (PDF)

## Abbreviations

Aβ: amyloid-beta
AChE: acetylcholinesterase
AD: Alzheimer’s disease
ADME: absorption, distribution, metabolism, excretion
ADMET: absorption, distribution, metabolism, excretion, and toxicity
AMES: Ames test (mutagenicity assay)
BBB: blood-brain barrier
BHT: butylated hydroxytoluene
CNS: central nervous system
CYP: cytochrome P450
DMSO: dimethyl sulfoxide
GI: gastrointestinal
hERG: human Ether-à-go-go-Related Gene
HIA: human intestinal absorption
LogP: partition coefficient (lipophilicity)
Log S: solubility
MTD: maximum tolerated dose
PAINS: pan-assay interference compounds
PDB: Protein Data Bank
RMSD: root-mean-square deviation
SAR: structure–activity relationship
SBDD: structure-based drug design
TEMPO: 2,2,6,6-tetramethyl-1-piperidinyloxy
TLC: thin layer chromatography
VDss: volume of distribution at steady state
