# Supplementary material for: Rational Design and Greener Synthesis of Selenylated Indolamides as Potential Anti-Alzheimer’s Agents
Source: ACS Omega. 2025 Nov 13;10(46):56334–48. doi: 10.1021/acsomega.5c08265 (PMC12658801; doi:10.1021/acsomega.5c08265)
Supplement: Supplementary file 1 [file ao5c08265_si_001.pdf]

Supporting Information

Rational Design and Greener Synthesis of Selenylated Indolamides as Potential Anti-Alzheimer's Agents

Angélica Justino Dias†, Aldo Sena Oliveira‡, Angélica Ceci Silva†, and Antonio Luiz Braga†**

†Department of Chemistry, Center for Physical and Mathematical Sciences, Federal University of Santa Catarina, 88040-900 Florianópolis, Santa Catarina, Brazil;
‡Gulbenkian Institute for Molecular Medicine (GIMM), Faculty of Medicine, University of Lisbon, 1649-028, Lisbon, Portugal.

Email: justino.a@posgrad.ufsc.br; braga.antonio@ufsc.br

October, 2025

EXPERIMENTAL SECTION 2

1. Computational Methods 2

1.1 Molecular Docking: 2

1.2 MM-GBSA Binding Energy Calculations: 2

1.3 ADMET Prediction: 2

1.4 PAINS and REOS Filtering: 3

1.5 CNS Multiparameter Optimization (MPO) Score: 3

2. General Procedure 3

3. Synthesis of substrates 4

4. Optimization of the Selenylation Reaction 7

5. General procedure for synthesis of 5a – 6a 8

6. Control experiments 16

7. NMR spectra 18

8. References 63

EXPERIMENTAL SECTION

1. Computational Methods

1.1 Molecular Docking: All docking simulations were carried out using the GOLD 2022.3.0 software suite (CCDC). The crystallographic structure of human acetylcholinesterase (AChE) in complex with donepezil (PDB ID: 4M0E) was retrieved from the Protein Data Bank and prepared using Discovery Studio Visualizer. Water molecules were removed, polar hydrogens were added, and the co-crystallized ligand was used to define the binding site. Redocking of the original ligand was performed to validate the protocol. Among the available scoring functions (GoldScore, ChemScore, ASP, ChemPLP), ChemPLP produced the lowest RMSD (0.002 Å) and was thus selected for all subsequent calculations. Docking was performed with 30 genetic algorithm runs per ligand, and the active site was defined as a 10 Å radius around the centroid of the native ligand. No constraints were applied. The top-scoring pose for each ligand was used for further analysis.[1,2]

1.2 MM-GBSA Binding Energy Calculations: To estimate the thermodynamic stability of ligand–protein complexes, MM-GBSA binding free energy calculations were performed using the Prime module (Schrödinger Suite 2022-3). The top-ranked docking poses of selected ligands (3a, 5a, 5b-para, and 6g) were minimized in the protein environment, and ΔG_bind values were calculated using the OPLS4 force field with VSGB solvent model. Default settings were used. This analysis provided a more accurate estimate of binding affinity by incorporating solvation and side-chain flexibility.[3,4]

1.3 ADMET Prediction: Pharmacokinetic and drug-likeness properties were predicted using SwissADME, pkCSM, and ProTox-II. SMILES strings of all compounds were used as input. The following parameters were assessed: gastrointestinal absorption, blood–brain barrier permeability (logBB), cytochrome P450 inhibition, hERG inhibition, solubility, Lipinski's Rule of Five violations, CNS multiparameter optimization (MPO) score, and predicted acute toxicity (oral LD_{50}, rat model). Toxicity classes were interpreted according to GHS classification.[5–7]

1.4 PAINS and REOS Filtering: Compounds were screened for structural alerts using the PAINS (Pan-Assay Interference Compounds) and REOS (Rapid Elimination of Swill) filters via SwissADME and FAF-Drugs4. Molecules containing reactive functional groups, frequent hitters, or assay-interfering substructures were excluded from further consideration.[8,9]

1.5 CNS Multiparameter Optimization (MPO) Score: The CNS MPO score was calculated following the original algorithm by Wager et al., based on six physicochemical parameters: cLogP, cLogD, molecular weight, topological polar surface area (TPSA), number of hydrogen bond donors, and pKa. Scores range from 0 to 6, with values above 4.0 considered favorable for CNS penetration.[10]

2. General Procedure

Unless specified otherwise, all reagents were procured from commercial suppliers and utilized without additional purification. All reactions of selenylation of indolamides were conducted under ambient atmospheric conditions. Indolamides substrates **3** and diselenides **2** were either obtained from commercial sources or synthesized following adaptation of previously reported protocols.[11–13] TLC analysis was conducted on glass-backed silica plates and visualized under UV light. Column chromatography was carried out using silica gel flash (200-300 mesh) and eluted with a mixture of hexane and ethyl acetate. Proton (^{1}H), carbon (^{13}C), and selenium (^{77}Se) nuclear magnetic resonance (NMR) spectra were acquired on Bruker 400 MHz NMR spectrometers using deuterated solvents as indicated. Chemical shifts are reported in parts per million (ppm) relative to tetramethylsilane and diphenyl diselenides with the solvent resonance serving as the internal standard. The following abbreviations were employed to describe chemical shift multiplicities: s = singlet, d = doublet, t = triplet, q = quartet and m = multiplet. Melting points were determined using a FISATOM Mod. 431D apparatus without any corrections. Infrared High-resolution mass spectrometry (HRMS) data were obtained using a Xevo G2-S QTof (Waters) mass spectrometer with an electrospray ionization (ESI) probe operating in both positive and negative ionization modes. TLC analysis was performed on aluminium silica plates and visualized with UV light.

3. Synthesis of substrates

3.1 Preparation of indolamides from tryptamine and Aromatic acyl chlorides

Scheme S1. Synthesis of Indolamide Derivatives from Tryptamine and Aromatic Acyl Chlorides

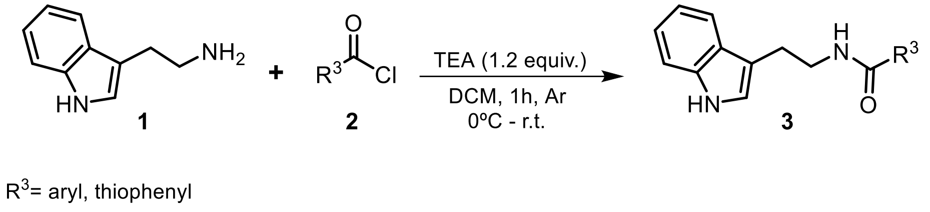

General procedure: Tryptamine (0,63 mmol, 0,1 g) was mixture in DCM (2,5 mL) under the argon atmosphere. The mixture was dropwise at 0 °C and TEA (0,75 mmol, 0,104 mL) and Aromatic acyl chlorides (0,63 mmol) was dispersed.[13] The reaction was then heated to room temperature and maintained for 1h. The resulting solution was diluted with ethyl acetate (50 mL) and the organic layer was washed by H_2O (20 mL) and dried with $MgSO_4$. Ethyl acetate was removed by rotary evaporator and the crude product was purified by silica column chromatography (elute: Ethyl acetate/ Hexane 60%) to afford the desired product indolamides **3a** – **3h**.

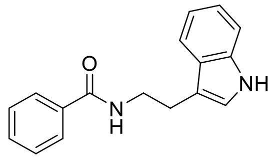

N-(2-(*1H*-indol-3-yl)ethyl)benzamide (**3a**): This compound was obtained as a white solid (0.120 g, 72%); m.p. 135-138 °C; ^{1}H NMR (400 MHz, DMSO-d_6) δ 10.83 (s, 1H), 8.63 (t, J = 5.7 Hz, 1H), 7.86 – 7.83 (m, 3H), 7.59 (d, J = 7.8 Hz, 1H), 7.54 – 7.43 (m, 2H), 7.34 (m, 1H), 7.18 (d, J = 2.4 Hz, 1H), 7.07 (m, 1H), 6.98 (m, 1.1 Hz, 1H), 3.54 (m, 2H), 2.95 (t, J = 7.5 Hz, 2H); ^{13}C NMR (101 MHz, DMSO) δ 166.1, 136.2, 134.7, 131.0, 128.2, 127.2, 127.1, 122.6, 120.9, 118.3, 118.2, 111.9, 111.4, 40.2, 25.2; HRMS (ESI): calcd for $C_{17}H_{16}N_2O$ $[M + Na]^+$ *m/z* 287.1160, found 287.1164.

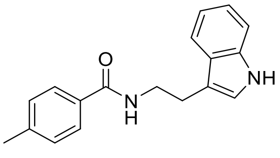

N-(2-(*1H*-indol-3-yl)ethyl)-4-methylbenzamide (**3b**): This compound was obtained as a white solid (0.142 g, 81%); m.p. 122-125 °C; ^{1}H NMR (400 MHz, DMSO-d_6) δ 10.82 (s, 1H), 8.54 (t, J = 5.5 Hz, 1H), 7.75 (d, J = 7.9 Hz, 2H), 7.58 (d, J = 7.9 Hz, 1H), 7.33 (d, J = 8.1 Hz, 1H), 7.26 (d, J = 7.8 Hz, 2H), 7.19 – 7.16 (m, 1H), 7.06 (t, J = 7.5 Hz, 1H), 6.98 (t, J = 7.4 Hz, 1H), 3.57 – 3.46 (m, 2H), 2.93 (t, J = 7.7 Hz, 2H), 2.35 (s, 3H); ^{13}C NMR (101 MHz, DMSO) δ 165.9, 140.8, 136.2, 131.9, 128.7, 127.3, 127.1,

122.6, 120.9, 118.3, 118.2, 111.9, 111.4, 25.2, 20.9. HRMS (ESI): calcd for $C_{18}H_{18}N_2O$ $[M + Na]^+$ *m/z* 301.1317, found 287.1164

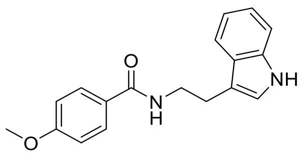

N-(2-(*1H*-indol-3-yl)ethyl)-4-methoxybenzamide (**3c**): This compound was obtained as a white solid (0.161 g, 87%). ^{1}H NMR (400 MHz, DMSO-d_6) δ 10.85 – 10.80 (m, 1H), 8.50 (t, J = 5.6 Hz, 1H), 7.86 – 7.82 (m, 2H), 7.59 (d, J = 7.8 Hz, 1H), 7.36 – 7.33 (m, 1H), 7.18 (d, J = 2.3 Hz, 1H), 7.07 (m, 1H), 6.98 (m, 3H), 3.80 (s, 3H), 3.58 – 3.48 (m, 3H), 2.94 (t, J = 7.5 Hz, 2H). ^{13}C NMR (101 MHz, DMSO) δ 165.6, 161.4, 136.3, 128.9, 127.3, 126.9, 122.6, 120.9, 118.3, 118.2, 113.4, 112.0, 111.4, 55.3, 25.3. HRMS (ESI) calculated for $C_{18}H_{18}N_2O_2$ $[M +H]^+$ 295.1441, found 295.1454.

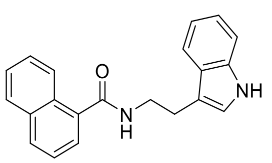

N-(2-(*1H*-indol-3-yl)ethyl)-1-naphthamide (**3d**): This compound was obtained as a white solid (0.190 g, 96%); m.p. 154-156 °C; ^{1}H NMR (400 MHz, DMSO-d_6) δ 10.93 – 10.84 (m, 1H), 8.67 (t, J = 5.7 Hz, 1H), 8.17 – 8.12 (m, 1H), 7.98 (m, 2H), 7.64 (d, J = 7.8 Hz, 1H), 7.58 – 7.49 (m, 4H), 7.38 (d, J = 8.1 Hz, 1H), 7.26 (d, J = 2.3 Hz, 1H), 7.13 – 7.06 (m, 1H), 7.04 – 6.98 (m, 1H), 3.69 – 3.60 (m, 2H), 3.04 (t, J = 7.4 Hz, 2H); ^{13}C NMR (101 MHz, DMSO) δ 168.5, 136.3, 135.2, 133.1, 129.8, 129.6, 128.1, 127.3, 126.6, 126.2, 125.5, 125.0, 125.0, 122.8, 120.9, 118.4, 118.2, 111.8, 111.4, 40.0, 25.1. HRMS (ESI): calcd for $C_{21}H_{18}N_2O$ $[M + Na]^+$ *m/z* 337.1317, found 337.1353.

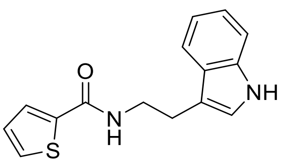

N-(2-(*1H*-indol-3-yl)ethyl)thiophene-2-carboxamide (**3e**): This compound was obtained as a gray solid (0.159 g, 93%); m.p. 142-145 °C; ^{1}H NMR (400 MHz, DMSO-d6) δ 10.83 (s, 1H), 8.65 (t, J = 5.6 Hz, 1H), 7.76 – 7.72 (m, 2H), 7.57 (d, J = 7.8 Hz, 1H), 7.33 (d, J = 8.1 Hz, 1H), 7.17 (d, J = 2.3 Hz, 1H), 7.14 (m, J = 4.9, 3.7 Hz, 1H), 7.08 – 7.03 (m, 1H), 7.01 – 6.95 (m, 1H), 3.50 (q, J = 7.0 Hz, 2H), 2.93 (t, J = 7.5 Hz, 2H); ^{13}C NMR (101 MHz, DMSO) δ 161.0, 140.3, 136.2, 130.5, 127.8, 127.8, 127.2, 122.6, 120.9, 118.3, 118.2, 111.8, 111.4, 40.1, 25.3. HRMS (ESI): calcd for $C_{15}H_{14}N_2OS$ $[M + Na]^+$ *m/z* 293.0724 found 293.0727.

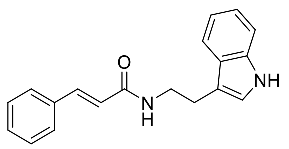

N-(2-(*1H*-indol-3-yl)ethyl)cinnamamide (**3f**): This compound was obtained as a white solid (0.159 g, 93%); m.p. 178-182 °C; ^{1}H NMR (400 MHz, DMSO-d6) δ 10.86 (s, 1H), 8.27 (t, J = 5.8 Hz, 1H), 7.57 (m, 3H), 7.49 – 7.33 (m, 6H), 7.20 – 7.17 (m, 1H), 7.08 (t, J = 7.5 Hz, 1H), 6.99 (t, J = 7.4 Hz, 1H), 6.66 (m, 1H), 3.50 (d, J = 7.0 Hz, 2H), 2.91 (t, J = 7.5 Hz, 2H); ^{13}C NMR (101 MHz, DMSO) δ 164.9, 138.6, 138.5, 136.3, 135.0, 129.4, 128.9, 127.5, 127.3, 122.7, 122.4, 120.9, 118.3, 118.3, 111.8, 111.4, 39.7, 25.3; HRMS (ESI): calcd for $C_{19}H_{18}N_2O$ $[M + Na]^+$ *m/z* 313.1317 found 313.1318.

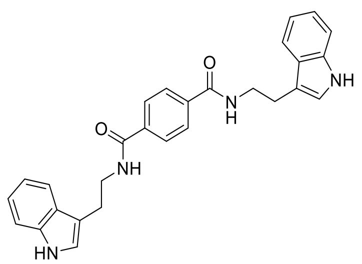

N^1,N^4-bis(2-*(1H*-indol-3-yl)ethyl)terephthalamide (**3g**): This compound was obtained as a white solid (0.111 g, 40%); ^{1}H NMR (400 MHz, DMSO-d_6) δ 10.89 – 10.80 (m, 2H), 8.77 (t, J = 5.7 Hz, 2H), 8.36 (d, J = 1.9 Hz, 1H), 7.98 (m, 2H), 7.62 – 7.52 (m, 3H), 7.35 (d, J = 8.0 Hz, 2H), 7.20 (d, J = 2.4 Hz, 2H), 7.10 – 7.04 (m, 2H), 6.99 (t, J = 7.3 Hz, 2H), 3.62 – 3.52 (m, 4H), 2.97 (t, J = 7.6 Hz, 4H). Molecular formula:^{13}C NMR (101 MHz, DMSO) δ 165.78, 136.27, 134.90, 129.57, 128.30, 127.29, 126.21, 122.64, 120.95, 118.30, 118.26, 111.87, 111.40, 40.30, 25.19. HRMS (ESI): calcd for $C_{28}H_{26}N_4O_2$ $[M + Na]^+$ *m/z* 473.1953 found 473.1954.

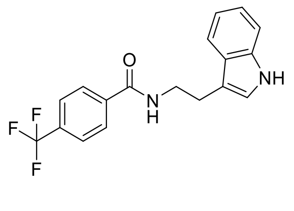

N-(2-(1H-indol-3-yl)ethyl)-4-(trifluoromethyl)benzamide (**3h**) ^{1}H NMR (400 MHz, DMSO) δ 10.82 (s, 1H), 8.38 (s, 1H), 7.94 (d, *J* = 8.1 Hz, 3H), 7.81 (d, *J* = 8.4 Hz, 2H), 7.58 (d, *J* = 7.7 Hz, 1H), 7.33 (d, *J* = 8.1 Hz, 1H), 7.15 (d, *J* = 2.5 Hz, 1H), 7.06 (ddd, *J* = 8.2, 6.9, 1.4 Hz, 1H), 6.97 (td, *J* = 7.6, 1.3 Hz, 1H), 3.92 (td, *J* = 7.3, 1.3 Hz, 2H), 3.06 (t, *J* = 7.3 Hz, 2H). ^{13}C NMR (101 MHz, DMSO) δ 192.72, 159.79, 139.82, 138.95, 136.22, 130.82, 130.51, 130.19, 129.88, 128.47, 128.19, 127.29, 126.20, 125.66, 125.62, 125.58, 125.54, 125.48, 122.90, 122.77, 120.90, 118.48, 118.22, 112.16, 111.36, 61.45, 40.15, 39.94, 39.73, 39.31, 39.10, 38.89, 26.53. HRMS (ESI): calcd for $C_{18}H_{15}F_3N_2O$ $[M - H]^+$ *m/z* 331.1058 found 331.1068.

4. Optimization of the Selenylation Reaction

Table S1. Optimization of the Reaction Conditions *a,b*

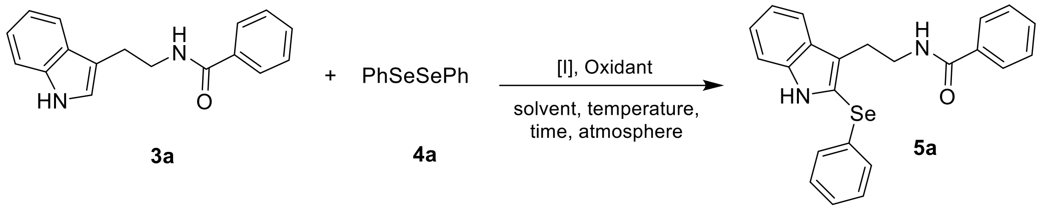

Entry	[I] (equiv.)	Oxidant (equiv.)	Solvent	Temperature	Time	Yield (%)
1	I_2 (1.0)	DMSO	-	100ºC	3 h	94
2	I_2 (1.0)	DMSO	-	100ºC	30 min	79
3	I_2 (1.0)	DMSO	-	100ºC	1 h	80
4	I_2 (1.0)	DMSO	-	100ºC	2 h	83
**5**	**I_2 (0.75)**	**DMSO**	**-**	**100ºC**	**3 h**	**94**
6	I_2 (0.5)	DMSO	-	100ºC	3 h	88
7	I_2 (0.24)	DMSO	-	100ºC	3 h	54
8[c]	I_2 (0.75)	DMSO	-	100ºC	1 h	80
9	I_2 (0.75)	DMSO	-	r.t.	3 h	29
10	I_2 (0.75)	DMSO	-	60ºC	3 h	36
11	TBAI (0.5)	DMSO	-	100ºC	3h	60
12	KI (0.5)	DMSO	-	100ºC	3 h	N.R.
13	-	DMSO	-	100ºC	3 h	N.R.
14	I_2 (0.75)	Oxone	DMSO	100ºC	3 h	90
15	TBAI (0.5)	Oxone	DMSO	r.t.	40 min	60
16	I_2 (0.75)	-	Cyrene	100ºC	3 h	N.R.
17	I_2 (0.75)	-	CH_3CN	80ºC	3 h	16
18	I_2 (0.75)	-	DMF	100ºC	3 h	31

a All the reactions were conducted with *N*-(2-(*1H*-indol-3-yl)ethyl)benzamide **3a** (0.2 mmol), diphenyl diselenide (0.1 mmol), sub-stochiometric amount, oxidant in solvent. Isolated yields were given. 1.3 mL solvent. *b* argon atmosphere. *c* Quenched by saturated $Na_2S_2O_3$ solution before purification.

5. General procedure for synthesis of 5a – 6a

Scheme S2. Synthesis of Selenylated Indolamides from Indolamide Derivatives and Diselenides

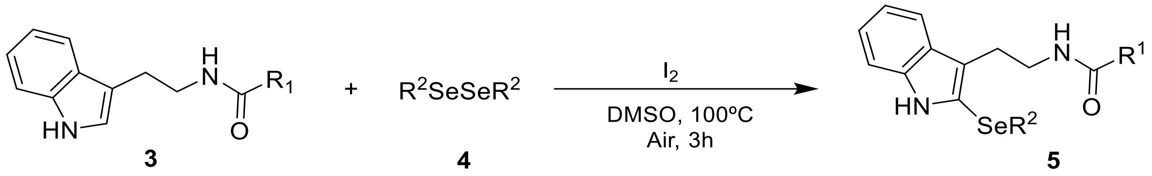

General procedure: To a mixture of diselenides (0.1 mmol), I2 (0.15 mmol, 0.019 g and DMSO (1,3 mL) was maintained for 15 minutes, after that the indolamide (0,2 mmol) was added. The reaction was stirred at 100ºC for 3h (monitored by TLC). Saturated sodium thiosulfate (1 mL) was added to quench the reaction. The reaction was diluted with ethyl acetate (5 mL) and washed by H_2O (20 mL). Aqueous phase was extracted with ethyl acetate (5 mL × 2). The organic layer was combined, ethyl acetate was removed by rotary evaporator and the crude product was purified by silica column chromatography (elute: ethyl acetate / hexane 30%) to afford the desired products **5a** – **5y**.

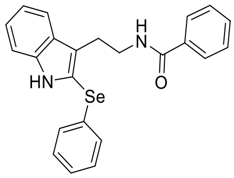

N-(2-(2-(phenylselanyl)-*1H*-indol-3-yl)ethyl)benzamide (**5a**): This compound was obtained as an orange solid (0.083 g, 94%); %); ^{1}H NMR (400 MHz, DMSO-d_6) δ 11.47 (s, 1H), 8.66 (t, J = 5.7 Hz, 1H), 7.85 – 7.80 (m, 2H), 7.71 (d, J = 7.9 Hz, 1H), 7.64 – 7.61 (m, 2H), 7.51 – 7.48 (m, 1H), 7.45 (d, J = 7.6 Hz, 1H), 7.35 – 7.31 (m, 3H), 7.24 – 7.19 (m, 3H), 7.16 – 7.12 (m, 1H), 7.04 (td, J = 7.5, 7.0, 1.0 Hz, 1H), 3.47 – 3.43 (m, 2H), 3.08 (dd, J = 8.8, 6.3 Hz, 2H). ^{13}C NMR (101 MHz, DMSO) δ 166.4, 138.1, 134.7, 132.5, 131.2, 129.6, 129.2, 128.4, 127.3, 126.6, 122.7, 120.1, 119.1, 119.1, 118.5, 111.5, 40.7, 26.0.^{77}Se NMR (76 MHz, DMSO-d_6) δ 248.6; HRMS (ESI): calcd for C23H20N2OSe [M-H]- m/z 419.0703 found 419.0664.

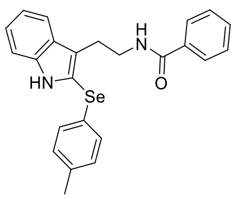

N-(2-(2-(p-tolylselanyl)-*1H*-indol-3-yl)ethyl)benzamide (**5b**): This compound was obtained as an orange solid (0.081 g, 93%); ^{1}H NMR (400 MHz, DMSO-d_6) δ 11.41 (s, 1H), 8.65 (t, J = 5.6 Hz, 1H), 7.85 – 7.80 (m, 2H), 7.70 (d, J = 7.9 Hz, 1H), 7.54 – 7.47 (m, 1H), 7.47 – 7.41 (m, 2H), 7.33 (d, J = 8.1 Hz, 1H), 7.14 (td, J = 7.9, 1.6 Hz, 4H), 7.04 (dd, J = 8.2, 2.3 Hz, 3H), 3.48 – 3.42 (m, 2H), 3.08 (dd, J = 8.8, 6.2 Hz, 2H), 2.20 (s, 3H).^{13}C NMR (101 MHz, DMSO) δ 166.1, 137.9, 135.9, 134.6, 131.0, 130.1, 129.5, 128.4, 128.2, 127.1, 122.4, 119.6, 118.9, 118.8, 111.3, 40.5, 25.9, 20.5. ^{77}Se NMR (76 MHz, DMSO-d_6) δ 242.9. HRMS (ESI): calcd for $C_{24}H_{22}N_2OSe$ $[M–H]^-$ m/z 433.0821; found 433.0820

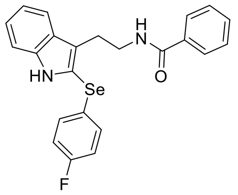

N-(2-(2-((4-fluorophenyl)selenyl)-*1H*-indol-3-yl)ethyl)benzamide (**5c**): This compound was obtained as an orange solid (0.082 g, 94%); ^{1}H NMR (400 MHz, Acetonitrile-d_3) δ 9.56 (s, 1H), 7.58 (ddt, *J* = 14.5, 8.0, 1.1 Hz, 3H), 7.38 – 7.32 (m, 1H), 7.29 – 7.22 (m, 3H), 7.16 – 7.09 (m, 3H), 7.04 (ddd, *J* = 8.2, 7.0, 1.2 Hz, 1H), 6.93 (ddd, *J* = 8.0, 7.1, 1.0 Hz, 1H), 6.81 – 6.74 (m, 2H), 3.51 – 3.44 (m, 2H), 3.05 (t, *J* = 7.1 Hz, 2H). ^{13}C NMR (101 MHz, Acetonitrile-d_3) δ 168.0, 162.9 (d, J = 244.1 Hz), 139.1, 135.9, 133.2 (d, J = 7.9 Hz), 132.1, 129.3, 128.6, 128.0, 127.7, 127.7, 123.9, 121.1, 120.5, 120.4, 120.1, 117.3 (d, J = 22.0 Hz), 112.1, 41.4, 26.8.^{77}Se NMR (76 MHz, DMSO-d_6) δ 247.4. HRMS (ESI): calcd for $C_{23}H_{19}FN_2OSe$ $[M + Na]^+$ *m/z* 437.0569 found 437.0568.

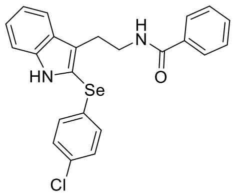

N-(2-(2-((4-chlorophenyl)selanyl)-*1H*-indol-3-yl)ethyl)benzamide (**5d**): This compound was obtained as an orange solid (0.097 g, 92 %). ^{1}H NMR (400 MHz, Acetonitrile-d_3) δ 9.51 (s, 1H), 7.69 (ddd, *J* = 11.7, 8.2, 1.3 Hz, 3H), 7.51 – 7.45 (m, 1H), 7.42 – 7.34 (m, 3H), 7.21 – 7.05 (m, 7H), 3.60 – 3.54 (m, 2H), 3.14 (t, *J* = 7.0 Hz, 2H). ^{13}C NMR (101 MHz, CD_3CN) δ 167.8, 139.2, 135.9, 133.0, 132.1, 132.0, 131.9, 130.3, 129.3, 128.6, 127.9, 124.0, 121.7, 120.5, 120.1, 119.6, 112.1, 41.3, 26.8. ^{77}Se NMR (76 MHz, DMSO) δ 251.7. HRMS (ESI): calcd for $C_{23}H_{19}ClN_2OSe$ $[M + Na]^+$ *m/z* 477.0247 found 477.0224.

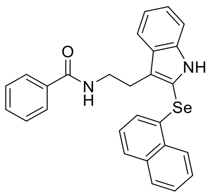

N-(2-(2-(naphthalen-1-ylselanyl)-*1H*-indol-3-yl)ethyl)benzamide (**5e**): This compound was obtained as an orange solid (0.093 g, 99 %). ^{1}H NMR (400 MHz, Acetonitrile-d_3) δ 9.39 (s, 1H), 8.15 – 8.08 (m, 1H), 7.80 (dd, *J* = 7.6, 1.6 Hz, 1H), 7.67 (dt, *J* = 7.7, 1.1 Hz, 1H), 7.63 – 7.58 (m, 3H), 7.48 (m, 2H), 7.41 – 7.36 (m, 1H), 7.32 – 7.23 (m, 3H), 7.21 – 7.13 (m, 2H), 7.03 (m, 3H), 3.54 – 3.48 (m, 2H), 3.10 (t, *J* = 7.1 Hz, 2H). ^{13}C NMR (101 MHz, CD_3CN) δ 167.8, 139.3, 136.0, 135.0, 133.4, 132.0, 131.5, 130.1, 129.7, 129.3, 128.8, 128.6, 127.9, 127.8, 127.5, 127.3, 126.7, 123.8, 121.4, 120.4, 119.9, 119.6, 112.0, 41.4, 26.8. HRMS (ESI): calcd for $C_{27}H_{22}N_2OSe$ $[M + Na]^+$ *m/z* 493.0797 found 493.0787.

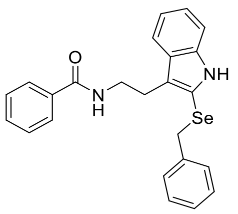

N-(2-(2-(benzylselanyl)-*1H*-indol-3-yl)ethyl)benzamide (**5f**): This compound was obtained as an orange solid (0.072 g, 82 %). ^{1}H NMR (400 MHz, Acetonitrile-d_3) δ 9.22 (s, 1H), 7.64 – 7.60 (m, 2H), 7.51 (dd, *J* = 7.9, 1.0 Hz, 1H), 7.42 – 7.38 (m, 1H), 7.35 – 7.30 (m, 2H), 7.28 – 7.24 (m, 2H), 7.09 – 7.03 (m, 4H), 6.99 – 6.92 (m, 3H), 3.93 (s, 2H), 3.38 – 3.32

(m, 2H), 2.81 (t, J = 7.1 Hz, 2H). ^{13}C NMR (101 MHz, CD_3CN) δ 167.7, 140.3, 138.9, 136.0, 132.1, 129.6, 129.3, 129.3, 128.6, 128.1, 127.9, 127.7, 123.4, 121.2, 120.6, 120.1, 119.7, 111.8, 41.3, 33.4, 26.6. HRMS (ESI): calcd for $C_{24}H_{22}N_2OSe$ $[M + Na]^+$ *m/z* 457.0797 found 457.0796.

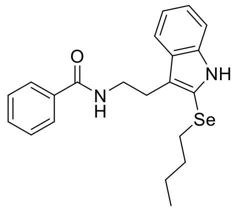

N-(2-(2-(butylselanyl)-1H-indol-3-yl)ethyl)benzamide (**5g**): This compound was obtained as an beige solid (0.056 g, 71%); ^{1}H NMR (400 MHz, DMSO-d6) δ 11.21 (s, 1H), 8.67 (t, J = 5.8 Hz, 1H), 7.87 (dd, J = 8.1, 1.4 Hz, 2H), 7.62 (d, J = 7.9 Hz, 1H), 7.55 – 7.42 (m, 3H), 7.34 – 7.30 (m, 1H), 7.12 – 7.06 (m, 1H), 7.03 – 6.96 (m, 1H), 3.46 (q, 3H), 3.04 (dd, J = 8.8, 6.3 Hz, 2H), 2.84 (t, J = 7.3 Hz, 2H), 1.52 (p, J = 7.3 Hz, 2H), 1.33 (h, J = 7.3 Hz, 2H), 0.82 (t, J = 7.3 Hz, 3H). ^{13}C (101 MHz, DMSO) δ 166.6, 138.1, 135.2, 131.5, 128.7, 127.8, 127.6, 122.1, 120.4, 119.1, 118.8, 118.4, 111.3, 41.0, 32.5, 28.8, 26.5, 22.6, 13.9. HRMS (ESI): calcd for $C_{21}H_{24}N_2OSe$ $[M - H]^-$ *m/z* 399.0982 found 399.0976.

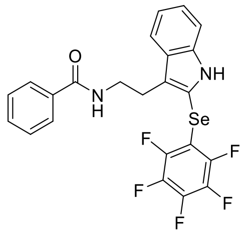

N-(2-(2-((perfluorophenyl)selanyl)-1*H*-indol-3-yl)ethyl)benzamide (**5h**): This compound was obtained as an orange solid (0.097 g, 96 %). ^{1}H NMR (400 MHz, DMSO-d_6) δ 11.37 (s, 1H), 8.66 (t, J = 5.7 Hz, 1H), 7.84 (dt, J = 7.0, 1.5 Hz, 2H), 7.66 (d, J = 7.9 Hz, 1H), 7.55 – 7.42 (m, 3H), 7.31 (t, J = 8.3 Hz, 1H), 7.14 (ddd, J = 8.2, 6.9, 1.2 Hz, 1H), 7.02 (ddd, J = 8.0, 7.0, 1.0 Hz, 1H), 3.47 – 3.40 (m, 2H), 3.13 (dd, J = 8.4, 6.4 Hz, 2H). ^{13}C NMR (101 MHz, DMSO) δ 166.0, 147.3, 144.9, 142.2, 139.6, 138.0, 137.7, 135.6, 134.5, 130.9, 128.1, 127.0, 126.7, 122.6, 119.6, 118.9, 118.9, 116.8, 111.3, 40.1, 25.8. ^{77}Se NMR (76 MHz, DMSO) δ 125.0. HRMS (ESI): calcd for $C_{23}H_{15}N_2OSe$ $[M + Na]^+$ *m/z* 533.0168 found 533.0166.

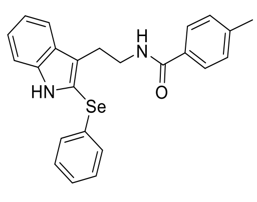

4-methyl-*N*-(2-(2-(phenylselanyl)-*1H*-indol-3-yl)ethyl)benzamide (**5i**): This compound was obtained as an orange solid (0.084 g, 96%). ^{1}H NMR (400 MHz, DMSO-d_6) δ 11.46 (s, 0H), 8.57 (t, J = 5.7 Hz, 0H), 7.73 (t, J = 8.3 Hz, 1H), 7.34 (d, J = 8.2 Hz, 0H), 7.28 – 7.11 (m, 2H), 7.05 (t, J = 7.5 Hz, 0H), 3.48 – 3.41 (m, 1H), 3.08 (dd, J = 8.8, 6.2 Hz, 1H), 2.34 (s, 1H). ^{13}C NMR (101 MHz, DMSO) δ 166.0, 140.8, 137.9, 132.4, 131.9, 129.4, 128.9, 128.7, 127.16, 127.1, 126.3, 122.5, 120.0, 118.9, 118.9, 118.2, 111.3, 40.5, 25.9, 20.9. ^{77}Se NMR (76 MHz, DMSO-d_6) δ 248.7. HRMS (ESI): calcd for $C_{24}H_{22}N_2OSe$ $[M + Na]^+$ *m/z* 457.0797 found 457.0791.

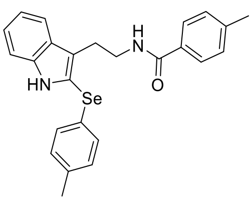

4-methyl-*N*-(2-(2-(p-tolylselanyl)-*1H*-indol-3-yl)ethyl)benzamide (**5j**): This compound was obtained as an orange solid (0.090 g, 99%). ^{1}H NMR (400 MHz, Acetonitrile-d_3) δ 9.45 (s, 1H), 7.61 – 7.55 (m, 1H), 7.54 – 7.48 (m, 2H), 7.25 (dd, *J* = 8.3, 0.9 Hz, 1H), 7.14 – 7.04 (m, 6H), 6.96 (m, 1H), 6.90 (d, *J* = 8.1 Hz, 2H), 3.51 – 3.44 (m, 2H), 3.04 (t, *J* = 7.1 Hz, 2H), 2.26 (s, 3H), 2.12 (s, 3H). ^{13}C NMR (101 MHz, CD_3CN) δ 167.8, 142.5, 139.1, 137.8, 133.1, 131.6, 131.2, 131.1, 131.1, 129.9, 129.1, 128.6, 128.0, 123.8, 120.9, 120.7, 120.4, 120.0, 112.0, 41.3, 26.8, 21.4, 21.0. ^{77}Se NMR (76 MHz, DMSO) δ 243.8. HRMS (ESI): calcd for $C_{25}H_{24}N_2OSe$ $[M + Na]^+$ *m/z* 471.0953 found 471.0950.

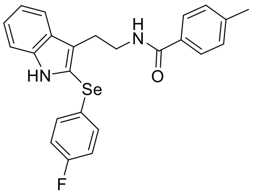

N-(2-(2-((4-fluorophenyl)selanyl)-*1H*-indol-3-yl)ethyl)-4-methylbenzamide (**5k**): This compound was obtained as an orange solid (0.070 g, 75%). ^{1}H NMR (400 MHz, Acetonitrile-d_3) δ 9.44 (s, 1H), 7.61 – 7.57 (m, 1H), 7.53 – 7.46 (m, 2H), 7.27 (dd, *J* = 8.2, 1.0 Hz, 1H), 7.23 – 7.15 (m, 2H), 7.14 – 7.05 (m, 3H), 7.04 – 6.94 (m, 2H), 6.89 – 6.80 (m, 2H), 3.51 – 3.44 (m, 2H), 3.05 (t, *J* = 7.1 Hz, 2H), 2.26 (s, 3H). ^{13}C NMR (101 MHz, DMSO) δ 167.8, 164.0, 161.5, 142.5, 139.1, 133.2, 133.2, 133.1, 129.9, 128.6, 127.9, 127.7, 127.6, 123.9, 121.1, 120.5, 120.4, 120.1, 117.4, 117.2, 112.1, 41.3, 26.8, 21.4. ^{77}Se NMR (76 MHz, DMSO) δ 247.5. HRMS (ESI): calcd for $C_{24}H_{20}FN_2OSe$ $[M - H]^+$ *m/z* 451.0726 found 451.0717.

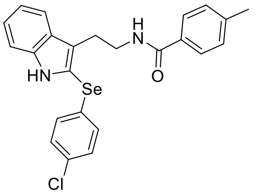

N-(2-(5-((4-chlorophenyl)selanyl)-*1H*-indol-3-yl)ethyl)-4-methylbenzamide (**5l**): This compound was obtained as an orange solid (0.066 g, 70%). ^{1}H NMR (400 MHz, Acetonitrile-d_3) δ 9.52 (s, 1H), 7.64 – 7.56 (m, 1H), 7.49 – 7.45 (m, 2H), 7.30 – 7.26 (m, 1H), 7.10 – 6.98 (m, 9H), 3.47 (q, *J* = 6.8 Hz, 2H), 3.03 (t, *J* = 7.1 Hz, 2H), 2.25 (s, 3H). ^{13}C NMR (101 MHz, CD_3CN) δ 167.8, 142.6, 139.2, 133.1, 133.0, 131.9, 131.9, 130.2, 129.9, 128.6, 127.9, 124.0, 121.7, 120.5, 120.1, 119.6, 112.2, 41.3, 26.8, 21.4. ^{77}Se NMR (76 MHz, DMSO) δ 251.8. HRMS (ESI): calcd for $C_{24}H_{20}ClN_2OSe$ $[M - H]^+$ *m/z* 467.0428 found 467.0416.

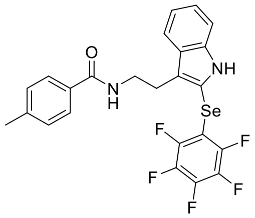

4-methyl-*N*-(2-(2-((perfluorophenyl)selanyl)-1*H*-indol-3-yl)ethyl)benzamide (**5m**): This compound was obtained as an orange solid (0.093 g, 91%). ^{1}H NMR (400 MHz, Acetonitrile-d_3) δ 9.45 (s, 1H), 7.57 (dt, *J* = 8.0, 1.0 Hz, 1H), 7.53 – 7.49 (m, 2H), 7.27 (dt, *J* = 8.3, 1.0 Hz, 1H), 7.16 – 7.12 (m, 2H), 7.09 (ddd, *J* = 8.2, 7.1, 1.1 Hz, 1H), 6.97 (ddd, *J* = 8.1, 7.0, 1.0 Hz, 2H), 3.52 – 3.43 (m, 2H), 3.10 (t, *J* = 7.0 Hz, 2H), 2.28 (s, 3H). ^{13}C NMR (101 MHz, CD_3CN) δ 167.7, 142.6, 139.1, 133.1, 129.9, 128.2, 127.9, 124.3, 121.7, 120.6, 120.2, 112.1, 41.0, 26.8, 21.4. HRMS (ESI): calcd for $C_{24}H_{17}F_5N_2OSe$ $[M + Na]^+$ *m/z* 547.0325 found 547.0332.

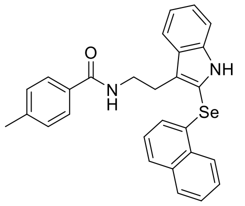

4-methyl-*N*-(2-(2-(naphthalen-1-ylselanyl)-1*H*-indol-3-yl)ethyl)benzamide (**5n**): This compound was obtained as an orange solid (0.073 g, 76%). ^{1}H NMR (400 MHz, Acetonitrile-d_3) δ 9.39 (d, *J* = 11.1 Hz, 1H), 8.13 – 8.08 (m, 1H), 7.82 – 7.78 (m, 1H), 7.70 – 7.58 (m, 2H), 7.50 – 7.44 (m, 4H), 7.28 – 7.22 (m, 1H), 7.19 – 7.05 (m, 5H), 7.02 – 6.95 (m, 2H), 3.54 – 3.46 (m, 2H), 3.09 (t, *J* = 7.0 Hz, 2H), 2.25 (d, *J* = 4.1 Hz, 3H). ^{13}C NMR (101 MHz, CD_3CN) δ 167.8, 142.5, 139.3, 135.0, 133.4, 133.1, 131.6, 130.1, 129.9, 129.7, 128.8, 128.5, 127.9, 127.8, 127.5, 127.5, 127.3, 127.1, 126.7, 123.8, 121.5, 120.4, 119.9, 119.6, 112.0, 41.3, 26.9, 21.4. ^{77}Se NMR (76 MHz, DMSO) δ 212.9. HRMS (ESI): calcd for $C_{28}H_{24}N_2OSe$ $[M + Na]^+$ *m/z* 507.0953 found 507.0954.

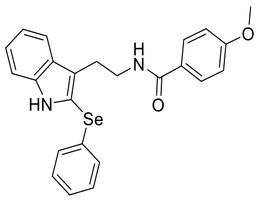

4-methoxy-*N*-(2-(2-(phenylselanyl)-1*H*-indol-3-yl)ethyl)benzamide (**5p**): This compound was obtained as an orange solid (0.067 g, 95%). ^{1}H NMR (400 MHz, Acetonitrile-d_3) δ 9.47 (s, 1H), 7.75 – 7.61 (m, 3H), 7.36 (dt, *J* = 8.2, 1.0 Hz, 1H), 7.31 – 7.15 (m, 6H), 7.08 (m, 1H), 6.99 (s, 1H), 6.94 – 6.89 (m, 2H), 3.81 (s, 3H), 3.59 – 3.50 (m, 2H), 3.13 (dd, *J* = 8.8, 5.5 Hz, 2H). ^{13}C NMR (101 MHz, CD_3CN) δ 163.0, 139.1, 130.7, 130.4, 129.7, 128.6, 127.6, 127.1, 123.9, 121.4, 120.4, 120.1, 118.8, 114.5, 112.1, 56.1, 41.3, 26.9. ^{77}Se NMR (76 MHz, DMSO-d_6) δ 248.7. HRMS (ESI): calcd for $C_{24}H_{22}N_2O_2Se$ $[M + Na]^+$ *m/z* 473.0746 found 473.0741.

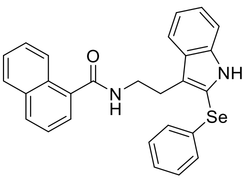

N-(2-(2-(phenylselanyl)-1*H*-indol-3-yl)ethyl)-1-naphthamide (**5q**): This compound was obtained as an beige solid (0.084 g, 91%). ^{1}H NMR (400 MHz, DMSO-d_6) δ 11.50 (s, 1H), 8.67 (t, *J* = 5.8 Hz, 1H), 8.20 – 8.14 (m, 1H), 8.01 – 7.93 (m, 2H), 7.76 (d, *J* = 7.9 Hz, 1H), 7.58 – 7.46 (m, 4H), 7.36 (d, *J* = 8.0 Hz, 1H), 7.30 – 7.13 (m, 6H), 7.05 (t, *J* = 7.5 Hz, 1H), 3.58 – 3.51 (m, 2H), 3.16 (t, 2H). ^{13}C NMR (101 MHz, DMSO) δ 169.0, 138.4, 135.4, 133.6, 132.9, 130.2, 130.1, 130.0, 129.9, 129.8, 129.4, 128.6, 127.7, 127.0, 126.8, 126.6, 126.0, 125.6, 125.3, 123.0, 120.4, 119.5, 119.4, 118.8, 111.8, 40.8, 26.4. ^{77}Se NMR (76 MHz, DMSO) δ 249.3. HRMS (ESI): calcd for $C_{27}H_{22}N_2OSe$ $[M + K]^+$ *m/z* 509.0536 found 509.0547.

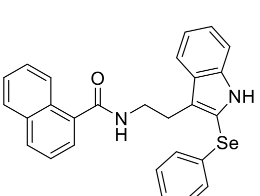

N-(2-(2-(p-tolylselanyl)-1*H*-indol-3-yl)ethyl)-1-naphthamide (**5r**): This compound was obtained as an orange solid (0.078 g, 95%).^{1}H NMR (400 MHz, Acetonitrile-d_3) δ 9.39 (s, 1H), 8.05 – 7.99 (m, 1H), 7.82 (m, 2H), 7.64 (dd, *J* = 8.0, 1.0 Hz, 1H), 7.46 – 7.22 (m, 6H), 7.11 – 6.95 (m, 4H), 6.87 (d, *J* = 7.8 Hz, 2H), 3.60 (q, *J* = 6.8 Hz, 2H), 3.12 (q, *J* = 7.1 Hz, 2H), 2.08 (s, 3H).^{13}C NMR (101 MHz, CD_3CN) δ 169.9, 139.1, 137.8, 136.0, 134.6, 133.6, 132.0, 131.5, 131.2, 131.1, 131.1, 131.0, 130.9, 129.1, 128.7, 127.6, 127.2, 126.6, 125.9, 125.8, 123.8, 120.8, 120.4, 120.0, 112.0, 41.2, 26.8, 21.0. HRMS (ESI): calcd for $C_{28}H_{24}N_2OSe$ $[M + Na]^+$ *m/z* 507.0953 found 507.0957.

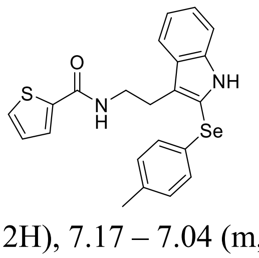

N-(2-(2-(p-tolylselanyl)-1*H*-indol-3-yl)ethyl)thiophene-2-carboxamide (**5s**): This compound was obtained as an yellow solid (0.053 g, 63%). ^{1}H NMR (400 MHz, Acetonitrile-d_3) δ 9.43 (s, 1H), 7.58 (dd, *J* = 7.9, 1.0 Hz, 1H), 7.52 – 7.36 (m, 2H), 7.34 – 7.20 (m, 2H), 7.17 – 7.04 (m, 3H), 7.02 – 6.90 (m, 4H), 3.58 – 3.33 (m, 2H), 3.16 – 2.92 (m, 2H), 2.11 (s, 3H). ^{13}C NMR (101 MHz, CD_3CN) δ 162.5, 141.1, 139.0, 137.9, 131.6, 131.3, 131.2, 131.1, 131.0, 131.0, 128.7, 128.6, 128.5, 123.8, 120.8, 120.8, 120.4, 119.9, 112.0, 41.2, 26.9, 21.0. HRMS (ESI): calcd for $C_{22}H_{20}N_2OSSe$ $[M + Na]^+$ *m/z* 463.0360 found 463.0348.

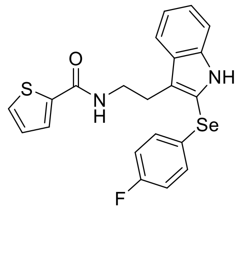

N-(2-(2-((4-fluorophenyl)selanyl)-1*H*-indol-3-yl)ethyl)thiophene-2-carboxami de (**5t**): This compound was obtained as an yellow solid (0.073 g, 89%). ^{1}H NMR (400 MHz, Acetonitrile-d_3) δ 9.37 (s, 1H), 7.59 (dd, *J* = 7.9, 1.0 Hz, 1H), 7.48 – 7.44 (m, 1H), 7.32 – 7.19 (m,

4H), 7.09 (ddd, J = 8.2, 7.0, 1.2 Hz, 1H), 7.02 – 6.95 (m, 3H), 6.91 – 6.83 (m, 2H), 3.46 (q, J = 6.7 Hz, 2H), 3.10 – 3.01 (m, 2H). ^{13}C NMR (101 MHz, CD_3CN) δ 164.1, 139.1, 133.4, 133.3, 131.0, 128.8, 128.7, 128.6, 128.5, 123.9, 121.0, 120.6, 120.5, 120.0, 119.8, 119.5, 117.4, 117.2, 112.1, 41.2, 26.9. HRMS (ESI): calcd for $C_{21}H_{17}FN_2OSSe$ [M + Na]$^+$ *m/z* 467.0109 found 467.0133.

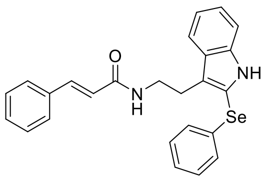

N-(2-(2-(phenylselanyl)-1*H*-indol-3-yl)ethyl)cinnamamide (**5u**): This compound was obtained as an yellow solid (0.050 g, 54%). ^{1}H NMR (400 MHz, Acetonitrile-d_3) δ 9.41 (s, 1H), 7.61 (d, J = 7.9 Hz, 1H), 7.44 – 7.40 (m, 2H), 7.36 (d, J = 15.7 Hz, 1H), 7.30 (dd, J = 7.7, 2.5 Hz, 3H), 7.19 – 7.08 (m, 7H), 7.01 (td, J = 7.5, 6.9, 1.0 Hz, 1H), 6.28 (d, J = 15.7 Hz, 1H), 3.42 (q, J = 6.8 Hz, 2H), 3.01 (t, J = 7.0 Hz, 2H). ^{13}C NMR (101 MHz, CD_3CN) δ 166.3, 140.1, 139.2, 136.2, 133.3, 130.6, 130.4, 130.4, 129.8, 128.6, 128.5, 127.6, 123.9, 122.9, 121.3, 120.4, 120.2, 120.1, 112.1, 40.8, 26.9. ^{77}Se NMR (76 MHz, DMSO) δ 249.3. HRMS (ESI): calcd for $C_{25}H_{22}N_2OSe$ [M + Na]$^+$ *m/z* 469.0797 found 469.0788.

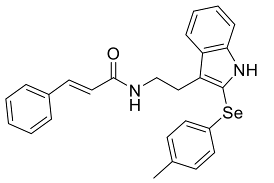

N-(2-(2-(p-tolylselanyl)-1*H*-indol-3-yl)ethyl)cinnamamide (**5v**): This compound was obtained as an yellow solid (0.059 g, 65%). ^{1}H NMR (400 MHz, Acetonitrile-d_3) δ 9.39 (s, 1H), 7.59 (dq, J = 8.0, 0.9 Hz, 1H), 7.43 – 7.40 (m, 2H), 7.36 (d, J = 15.7 Hz, 1H), 7.31 – 7.26 (m, 4H), 7.12 – 7.07 (m, 3H), 7.00 (ddd, J = 8.0, 7.0, 1.1 Hz, 1H), 6.97 – 6.93 (m, 2H), 6.28 (d, J = 15.7 Hz, 1H), 3.45 – 3.40 (m, 2H), 3.01 (t, J = 7.0 Hz, 2H), 2.12 (s, 3H). ^{13}C NMR (101 MHz, CD_3CN) δ 166.3, 140.1, 139.1, 137.8, 136.2, 131.5, 131.1, 130.4, 129.8, 129.2, 128.6, 128.5, 123.8, 122.9, 120.8, 120.7, 120.4, 120.0, 112.0, 58.0, 40.8, 26.9, 21.0. ^{77}Se NMR (76 MHz, DMSO) δ 243.8. HRMS (ESI): calcd for $C_{26}H_{23}N_2OSe$ [M + Na]$^+$ *m/z* 459.0977 found 459.0973.

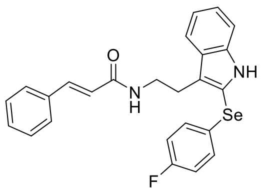

N-(2-(2-((4-fluorophenyl)selanyl)-1*H*-indol-3-yl)ethyl)cinnamamide (**5w**): This compound was obtained as an yellow solid (0.080 g, 86%) ^{1}H NMR (400 MHz, Acetonitrile-d_3) δ 9.54 (s, 1H), 7.58 – 7.55 (m, 1H), 7.41 – 7.33 (m, 3H), 7.30 – 7.24 (m, 4H), 7.19 – 7.15 (m, 2H), 7.07 (ddd, J = 8.2, 7.0, 1.2 Hz, 1H), 6.97 (ddd, J = 8.0, 7.0, 1.0 Hz, 1H), 6.86 – 6.79 (m, 2H), 6.30 (d, J = 15.8 Hz, 1H), 3.41 (q, J = 6.8 Hz, 2H), 3.00 (t, J = 7.0 Hz, 2H). ^{13}C NMR (101 MHz, CD_3CN) δ 166.5, 140.3, 139.1, 136.1,

133.2, 133.1, 130.4, 129.8, 128.6, 128.5, 123.9, 122.8, 121.0, 120.6, 120.4, 120.1, 117.4, 117.2, 112.1, 40.9, 26.9. ^{77}Se NMR (76 MHz, DMSO) δ 248.3. HRMS (ESI): calcd for $C_{25}H_{20}FN_2OSe$ $[M - H]^+$ *m/z* 463.0726 found 463.0732.

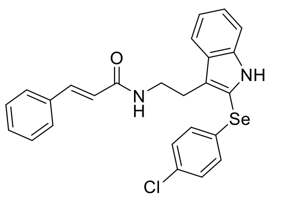

N-(2-(2-((4-chlorophenyl)selanyl)-1*H*-indol-3-yl)ethyl)cinnamamide (**5x**): This compound was obtained as an yellow solid (0.083 g, 87%). ^{1}H NMR (400 MHz, DMSO-d_6) δ ^{1}H NMR (400 MHz, Acetonitrile-d_3) δ ^{1}H NMR (400 MHz, Acetonitrile-d_3) δ 9.47 (s, 1H), 7.60 (dd, *J* = 7.9, 1.0 Hz, 1H), 7.41 – 7.25 (m, 7H), 7.17 – 7.08 (m, 4H), 7.01 (ddd, *J* = 8.1, 7.0, 1.1 Hz, 1H), 6.26 (dd, *J* = 15.8, 1.3 Hz, 1H), 3.42 (q, *J* = 6.7 Hz, 2H), 2.99 (t, *J* = 6.9 Hz, 2H). ^{13}C NMR (101 MHz, DMSO) δ 166.4, 140.2, 139.2, 136.2, 133.0, 132.4, 132.1, 131.9, 130.4, 130.3, 129.8, 128.6, 128.5, 124.1, 122.8, 121.6, 120.5, 120.1, 119.7, 112.1, 40.8, 26.9. ^{77}Se NMR (76 MHz, DMSO) δ 252.7. HRMS (ESI): calcd for $C_{25}H_{20}ClN_2OSe$ $[M - H]^+$ *m/z* 479.0428 found 479.0425.

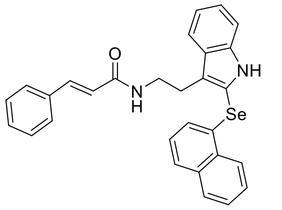

N-(2-(2-(naphthalen-1-ylselanyl)-1*H*-indol-3-yl)ethyl)cinnamamide (**5y**): This compound was obtained as an yellow solid (0.097 g, 98%). ^{1}H NMR (400 MHz, Acetonitrile-d_3) δ 9.40 (d, *J* = 12.3 Hz, 1H), 8.14 – 8.08 (m, 1H), 7.77 (dd, *J* = 8.0, 1.5 Hz, 1H), 7.68 – 7.58 (m, 2H), 7.50 – 7.42 (m, 3H), 7.34 (dq, *J* = 4.3, 2.0 Hz, 3H), 7.29 – 7.24 (m, 4H), 7.20 – 7.16 (m, 2H), 7.11 – 7.05 (m, 1H), 7.00 (ddd, *J* = 8.0, 7.0, 1.1 Hz, 1H), 6.20 (dd, *J* = 15.6, 1.7 Hz, 1H), 3.43 (q, *J* = 6.7 Hz, 2H), 3.04 (t, *J* = 6.9 Hz, 2H). ^{13}C NMR (101 MHz, DMSO) δ 166.3, 146.0, 140.1, 136.1, 135.0, 133.3, 131.8, 130.4, 129.8, 129.8, 129.7, 128.6, 128.5, 127.8, 127.5, 127.3, 126.6, 123.8, 122.8, 121.4, 120.4, 119.9, 119.6, 112.0, 40.8, 26.9. ^{77}Se NMR (76 MHz, DMSO) δ 213.4. HRMS (ESI): calcd for $C_{29}H_{23}N_2OSe$ $[M - H]^+$ *m/z* 495.0977 found 495.0969.

Scheme S3. Gram-Scale Selenylation of **5a**

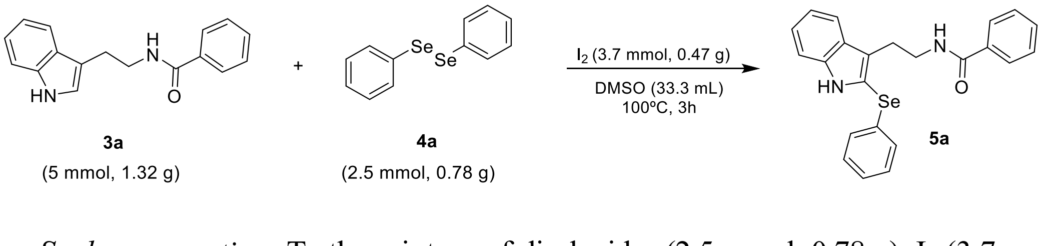

Gram-Scale preparation: To the mixture of diselenides (2.5 mmol, 0.78 g), I_2 (3.7 mmol, 0.47 g) and DMSO (33.3 mL) was maintained for 15 minutes, after that indolamide **3a** (5 mmol, 1.32 g) was added. The reaction was stirred at 100ºC for 3h (monitored by TLC). Saturated sodium thiosulfate (1 mL) was added to quench the reaction. The reaction

was diluted with ethyl acetate (50 mL) and washed by H_2O (200 mL). Aqueous phase was extractedwith ethyl acetate (50 mL × 2). The organic layer was combined, ethyl acetate was removed by rotary evaporator and the crude product was purified by silica column chromatography (elute: ethyl acetate / hexane 30%) to afford the **5a.** This compound was obtained as a yellow solid (1.8249 g, 88%).

6. Control experiments

Scheme S4. Selenylation Control Reactions with Radical Scavengers

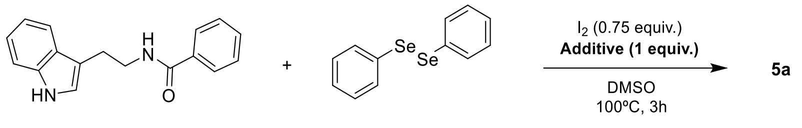

3a (1.0 equiv.) **4a** (0.5 equiv.)

Additive	5a
BHT	65%
TEMPO	31%
hydroquinone	85%

To the mixture of diphenyl diselenide **4a** (0.1 mmol, 0,031 g), I_2 (0,15 mmol, 0,019 g), additive (TEMPO or BHT or hydroquinone, 0.2 mmol) and DMSO (1,3 mL) was maintained for 15 minutes, after that indolamide **3a** (0,2 mmol, 0,0528 g) was added. The reaction was stirred at 100ºC for 3h (monitored by TLC). Saturated sodium thiosulfate (1 mL) was added to quench the reaction. The reaction was diluted with ethyl acetate (5 mL) and washed by H_2O (20 mL). Aqueous phase was extractedwith ethyl acetate (5 mL × 2). The organic layer was combined, ethyl acetate was removed by rotary evaporator and the crude product was purified by silica column chromatography (elute: ethyl acetate / hexane 30%) to afford the **5a**.

Scheme S5. Selenylation of Indolamide **3a** Using Phenyl Selenyl Bromide

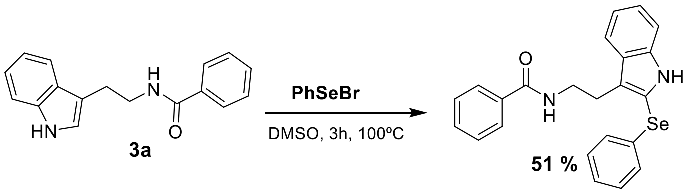

To the mixture of phenyl phenyselenyl bromide **4’** (0.2 mmol, 0.047 g), DMSO (1.3 mL) and indolamide **3a** (0.2 mmol, 0.052 g) was added. The reaction was stirred at 100°C for 3h (monitored by TLC). Saturated sodium thiosulfate (1 mL) was added to quench the reaction. The reaction was diluted with ethyl acetate (5 mL) and washed by H_2O (20 mL). Aqueous phase was extracted with ethyl acetate (5 mL × 2). The organic layer was combined, ethyl acetate was removed by rotary evaporator and the crude product was purified by silica column chromatography (elute: ethyl acetate / hexane 30%) to afford **5a.**

Scheme S6. Selenylation of Indolamide **3a** Using Different Iodine Sources

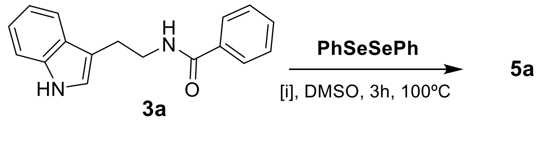

[I]	Yield (%)
I_2	94
HI	94

To the mixture of diphenyl diselenide **4a** (0.1 mmol, 0.031 g), hydroiodic acid (0.1 mmol) and DMSO (1.3 mL) was maintained for 15 minutes, after that indolamide **3a** (0.2 mmol,

0.0528 g) was added. The reaction was stirred at 100ºC for 3h (monitored by TLC). Saturated sodium thiosulfate (1 mL) was added to quench the reaction. The reaction was diluted with ethyl acetate (5 mL) and washed by H_2O (20 mL). Aqueous phase was extracted with ethyl acetate (5 mL × 2). The organic layer was combined, ethyl acetate was removed by rotary evaporator and the crude product was purified by silica column chromatography (elute: ethyl acetate / hexane 30%) to afford the **5a**.

Scheme S7. Control experiments

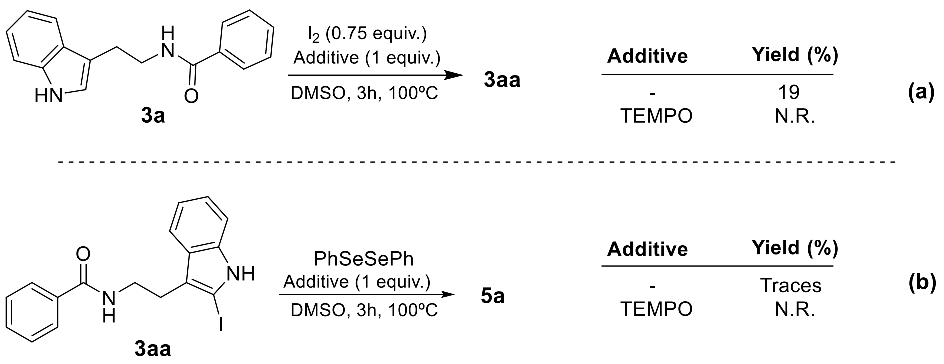

Control reaction ***(a):*** Indolamide **3a** (0.2 mmol, 0.0528 g), I_2 (0.15 mmol, 0.019 g), additive (TEMPO, 0.2 mmol) and DMSO (1.3 mL) was maintained in stirred at 100ºC for 3h (monitored by TLC). Saturated sodium thiosulfate (1 mL) was added to quench the reaction. The resulting mixture was diluted with ethyl acetate (5 mL) and washed with water (20 mL). The aqueous phase was further extracted with ethyl acetate (5 mL × 2). The combined organic layer was concentrated by rotary evaporation to remove ethyl acetate, and the resulting crude product was purified by silica column chromatography using a ethyl acetate / hexane (50%) eluent system. This purification process afforded the product **3aa** with a yield of 19%. *Control reaction* ***(b):*** To the mixture of diphenyl diselenide **4a** (0.1 mmol), I_2 (0.15 mmol) and additive (TEMPO, 0.2 mmol) was added to **3aa** (0.2 mmol) in DMSO (1.3 mL). The reaction was stirred at 100ºC for 3h (monitored by TLC).

7. NMR spectra

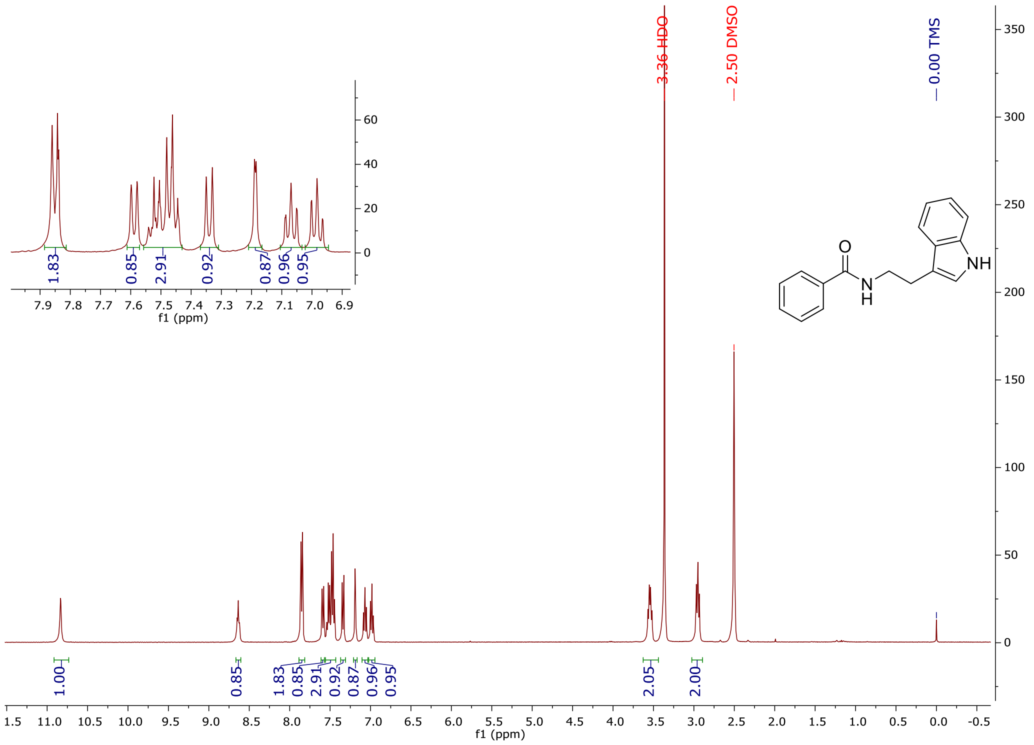

Figure S1. NMR of ^{1}H (400 MHz) of compound **3a**

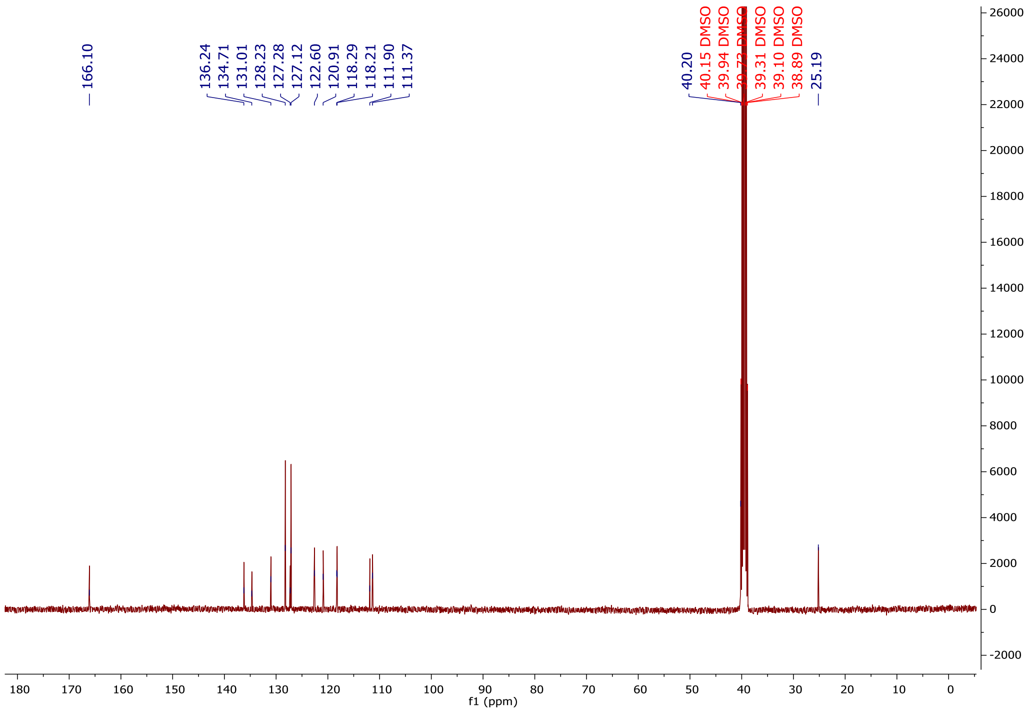

Figure S2. NMR of ^{13}C (101 MHz) of compound **3a**

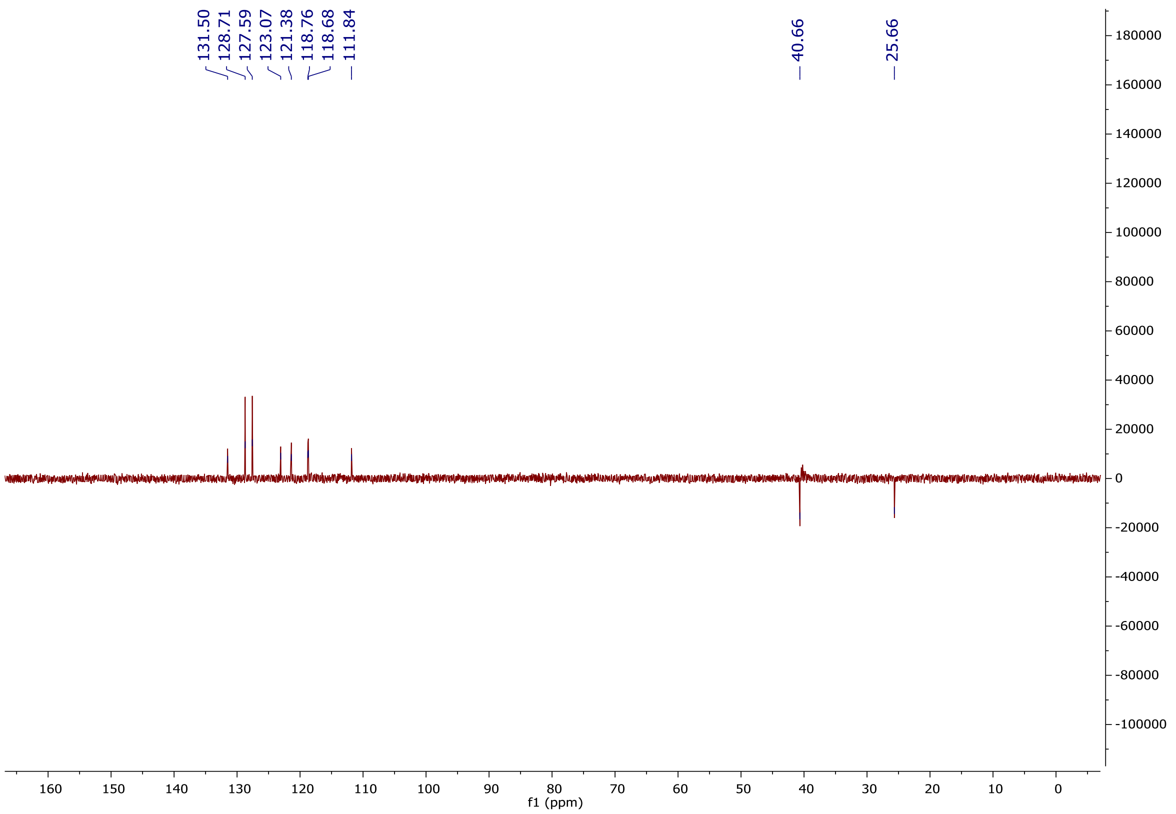

Figure S3. DEPT-135 in DMSO-d_6 of compound **3a**

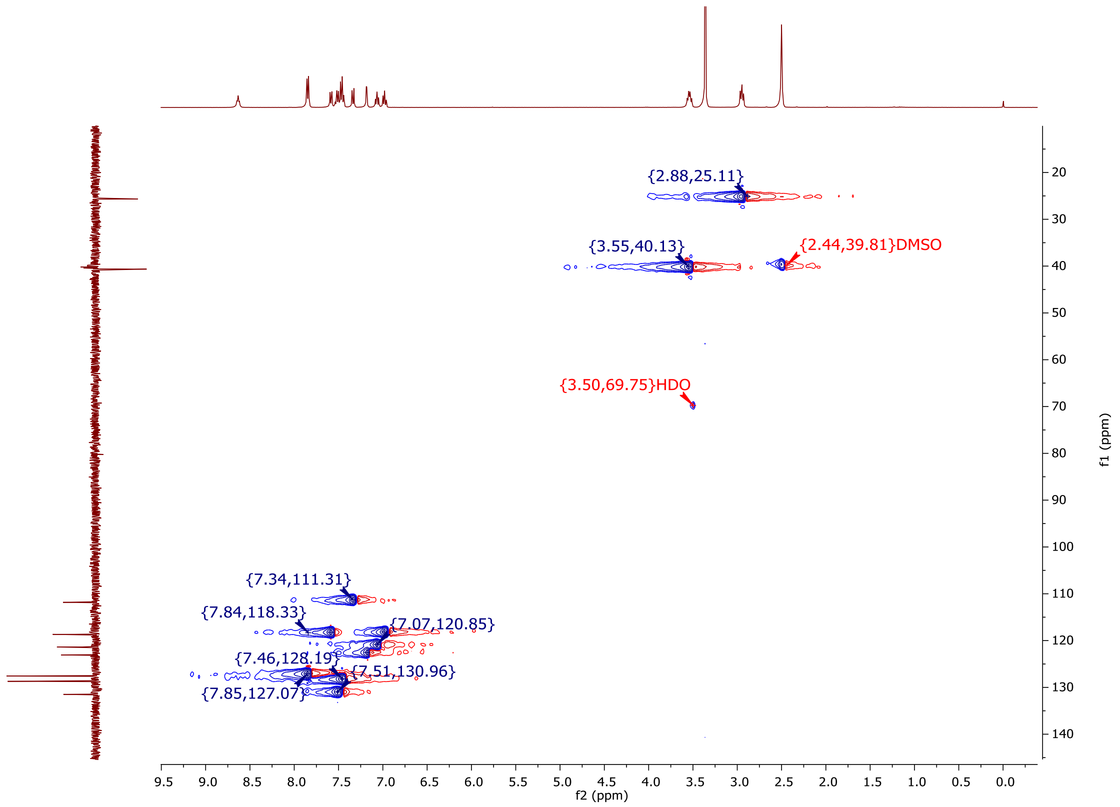

Figure S4. HSQC in DMSO-d_6 of compound **3a**

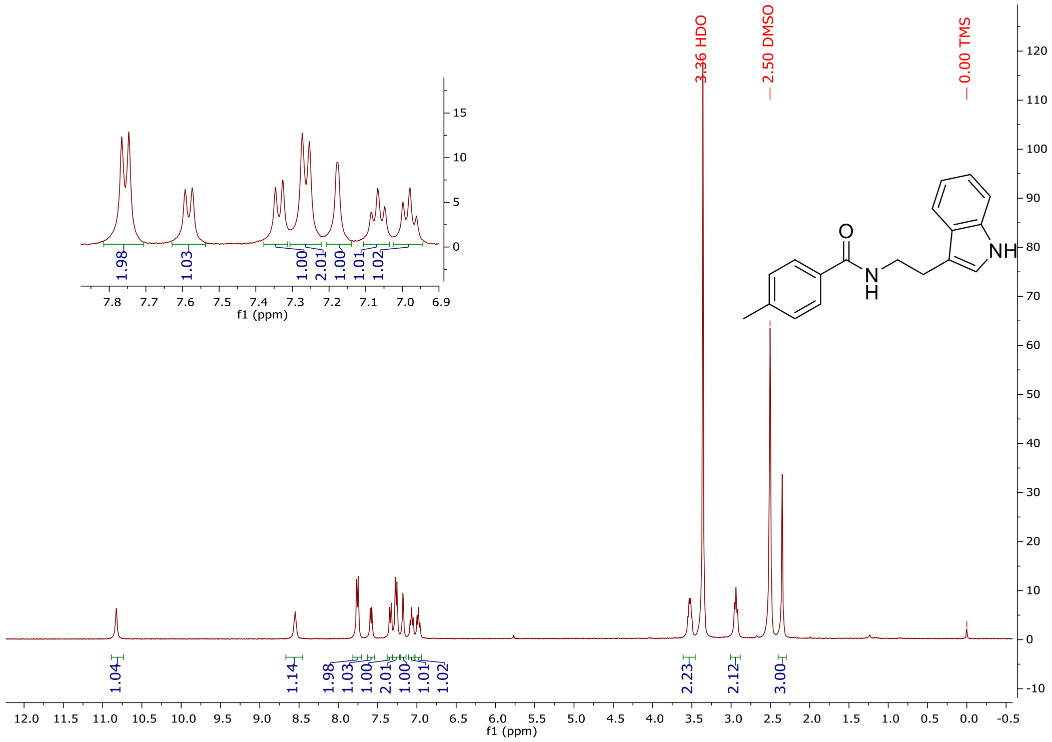

Figure S5. NMR of ^{1}H (400 MHz) in DMSO-d_6 of compound **3b**

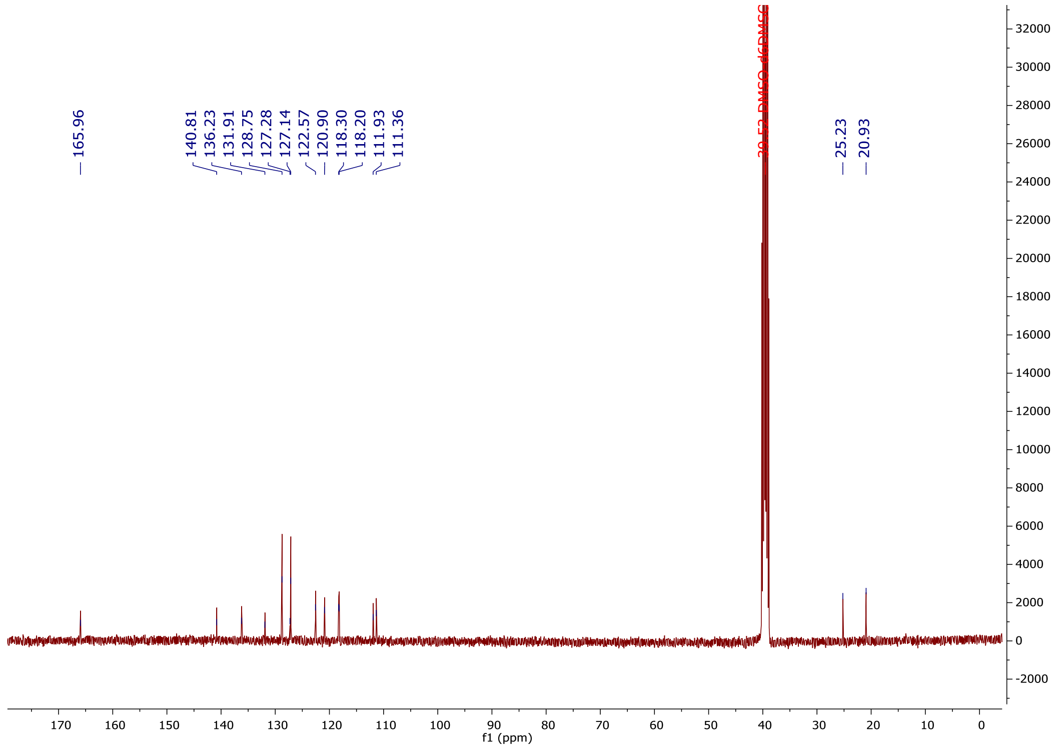

Figure S6. NMR of ^{13}C (101 MHz) in DMSO-d_6 of compound **3b**

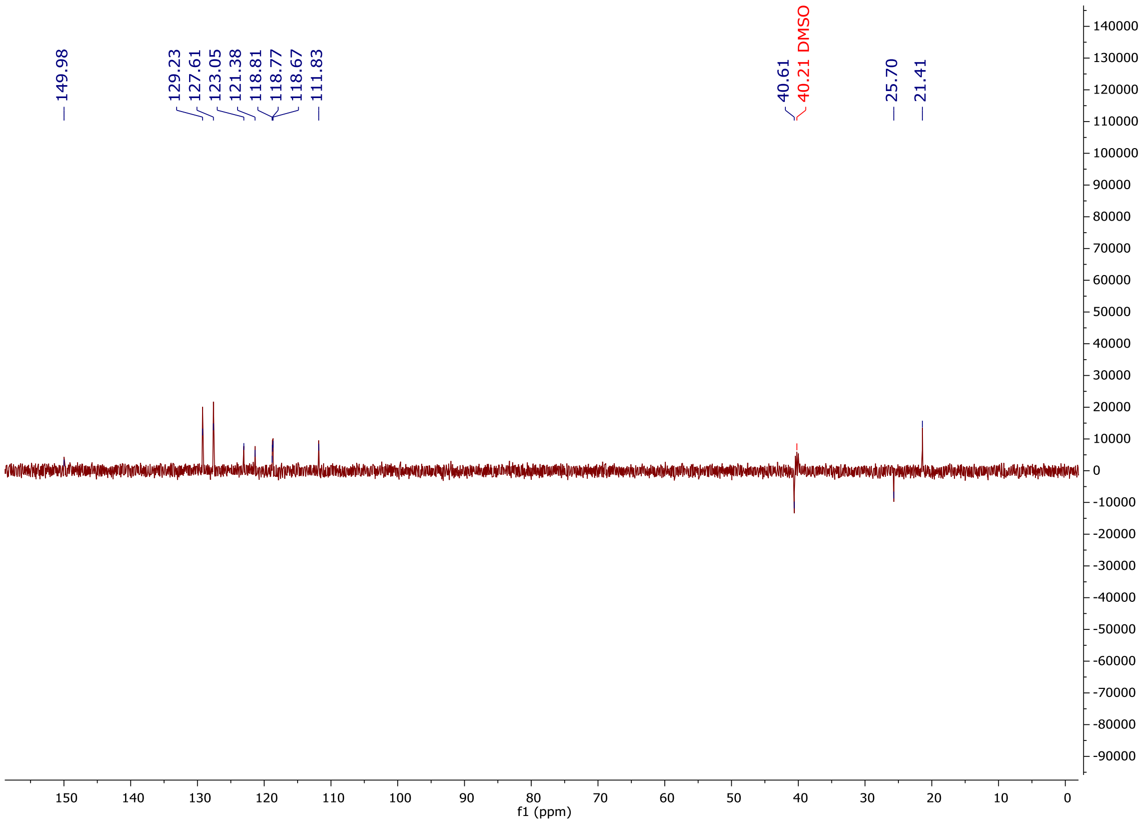

Figure S7. DEPT -135 in DMSO-d$_6$ of compound **3b**

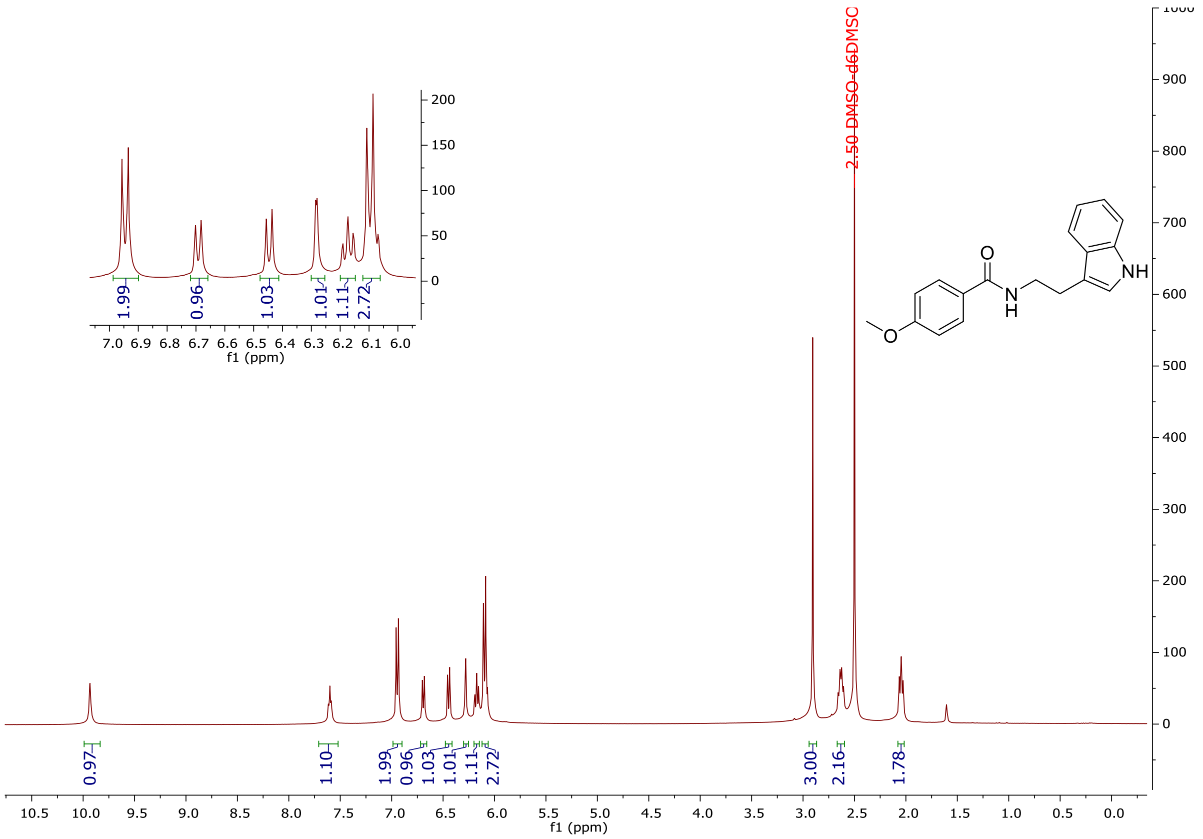

Figure S8. NMR of ^{1}H (400 MHz) in DMSO-d$_6$ of compound **3c**

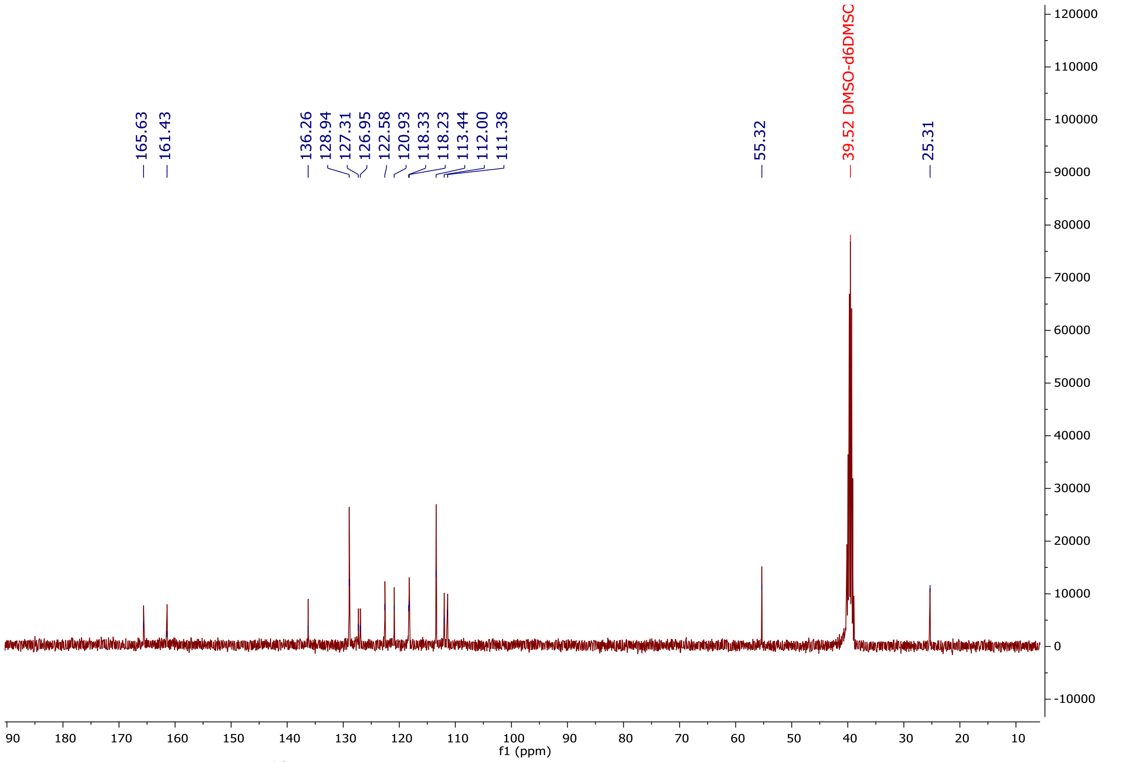

Figure S9. NMR of ^{13}C (101 MHz) in DMSO-d$_6$ of compound **3c**

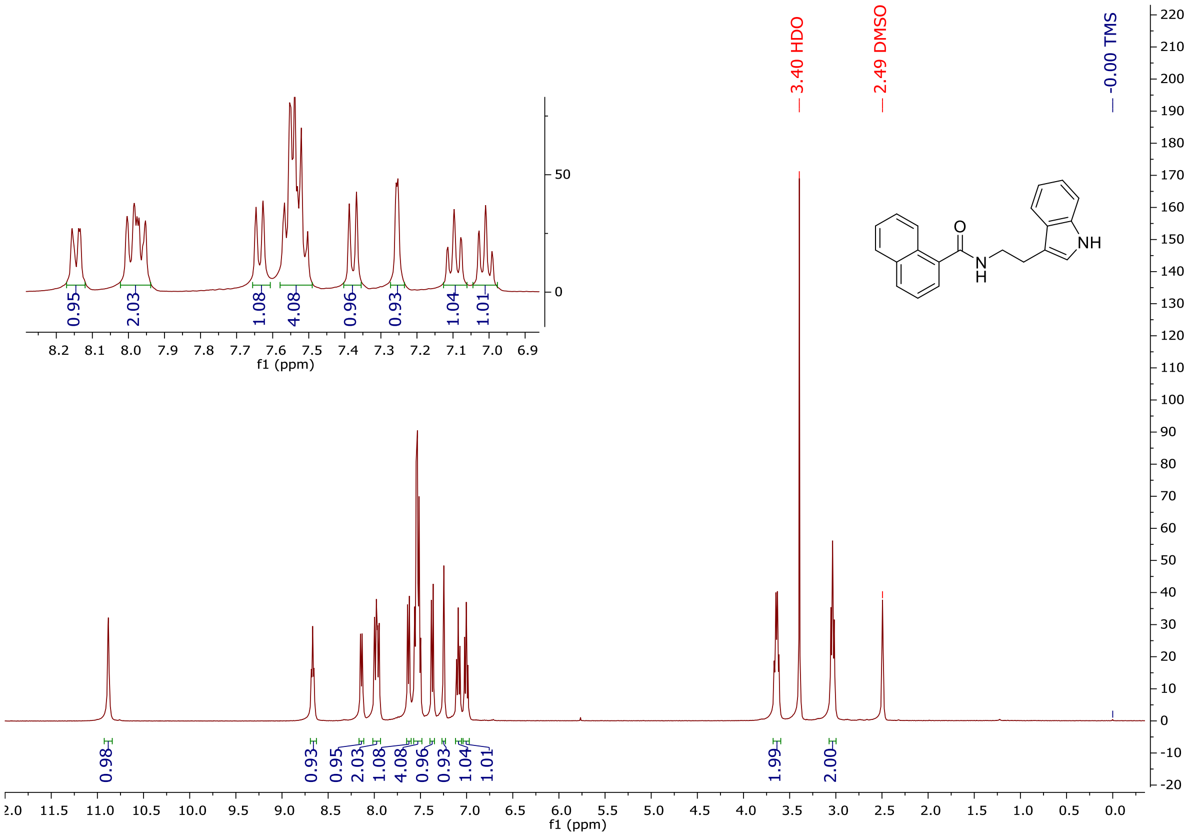

Figure S10. NMR of ^{1}H (400 MHz) in DMSO-d$_6$ of compound **3d**.

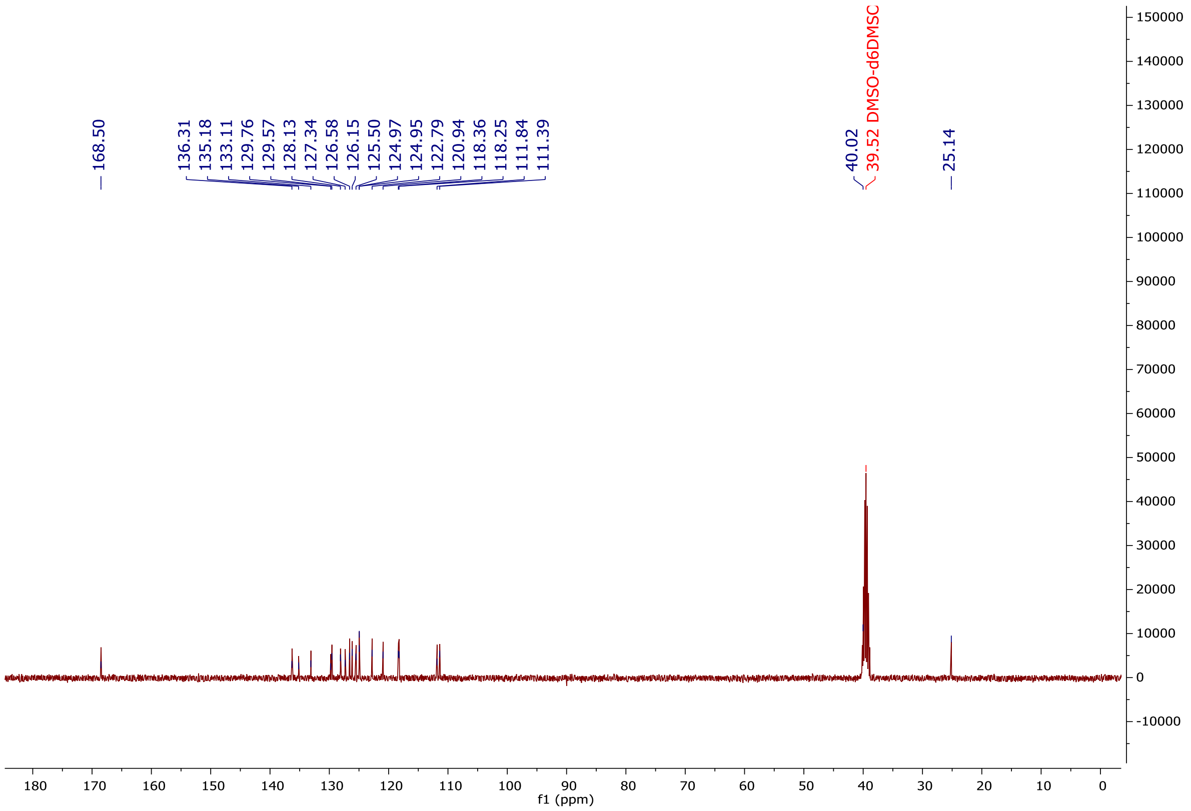

Figure S11. NMR of ^{13}C (101 MHz) in DMSO-d$_6$ of compound **3d**.

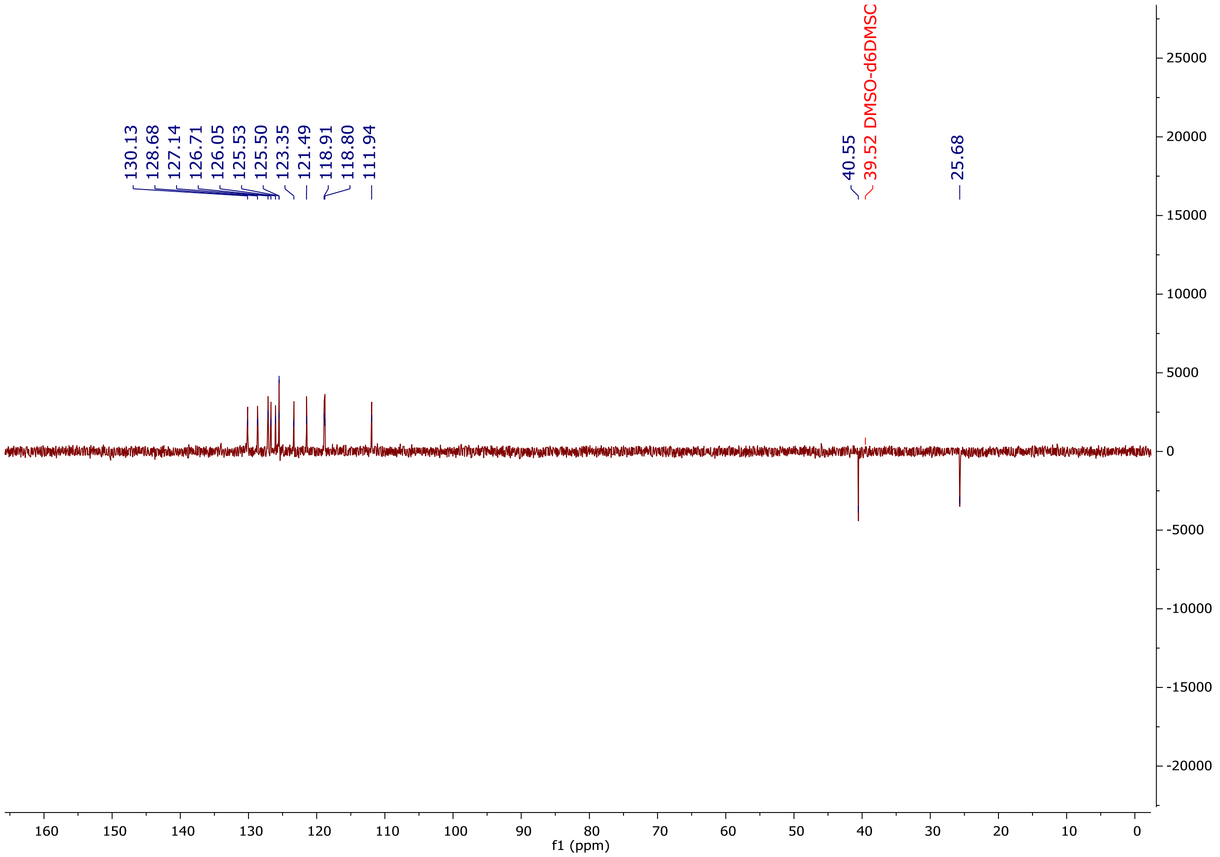

Figure S12. DEPT-135 in DMSO-d$_6$ of compound **3d**.

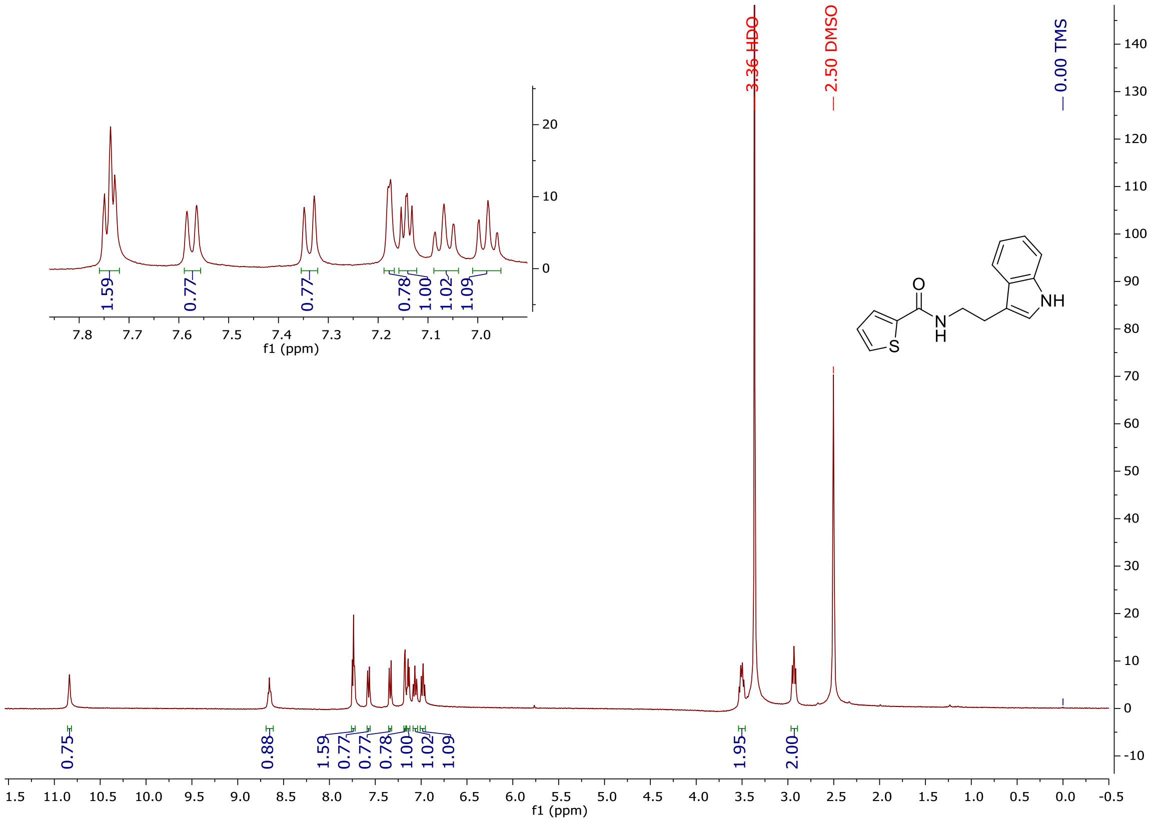

Figure S13. NMR of ^{1}H (400 MHz) in DMSO-d$_6$ of compound **3e.**

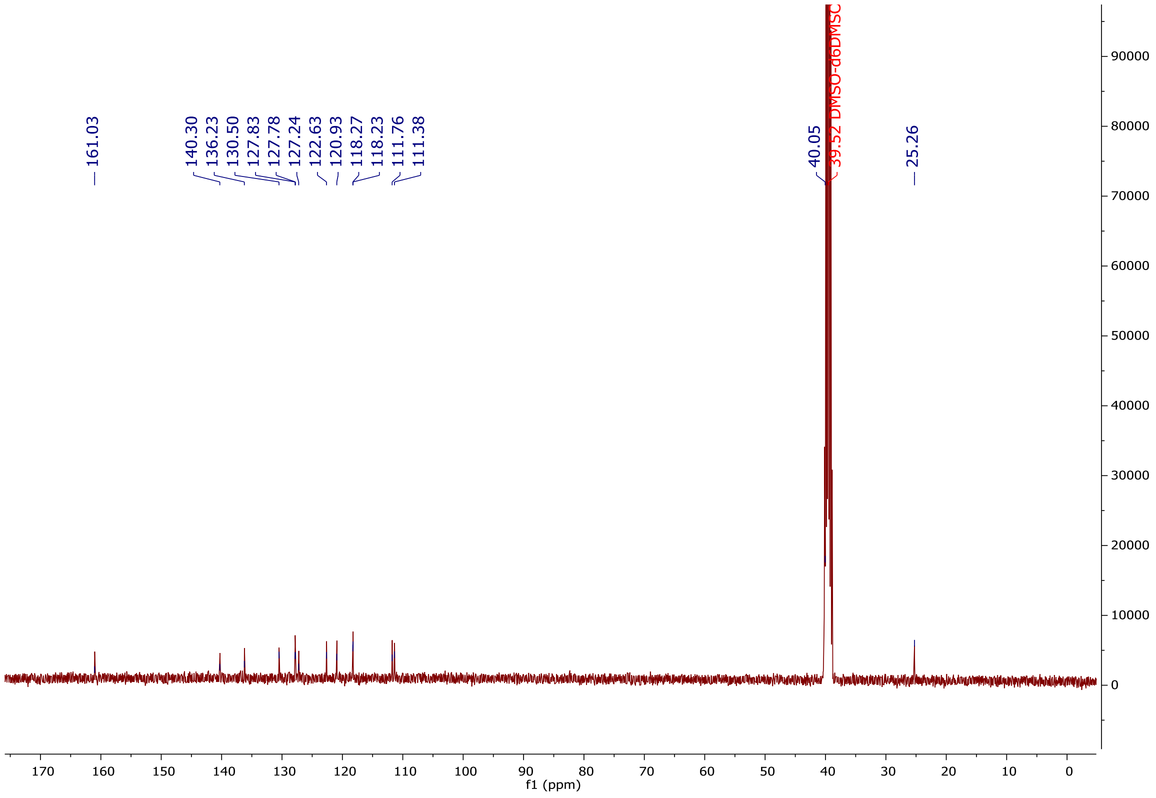

Figure S14. NMR of ^{13}C (101 MHz) in DMSO-d$_6$ of compound **3e.**

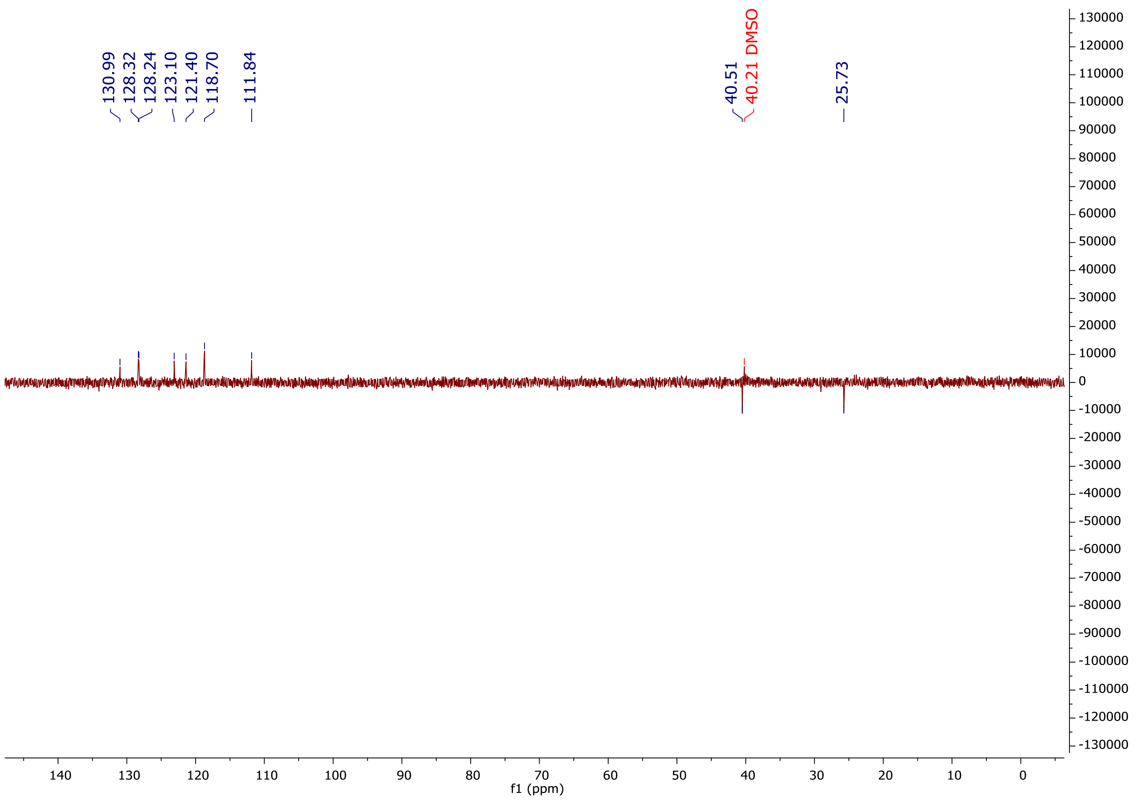

Figure S15. DEPT-135 in DMSO-d$_6$ of compound **3e.**

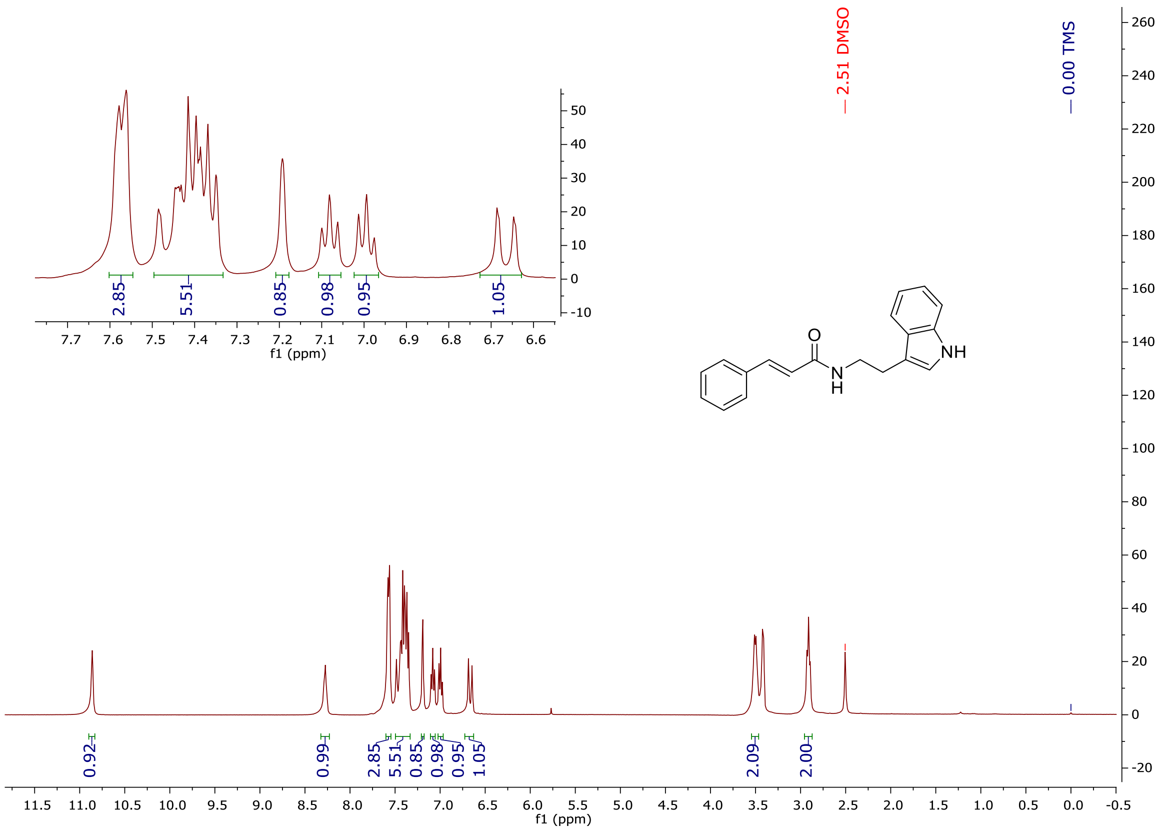

Figure S16. NMR of ^{1}H (400 MHz) in DMSO-d$_6$ of compound **3f**

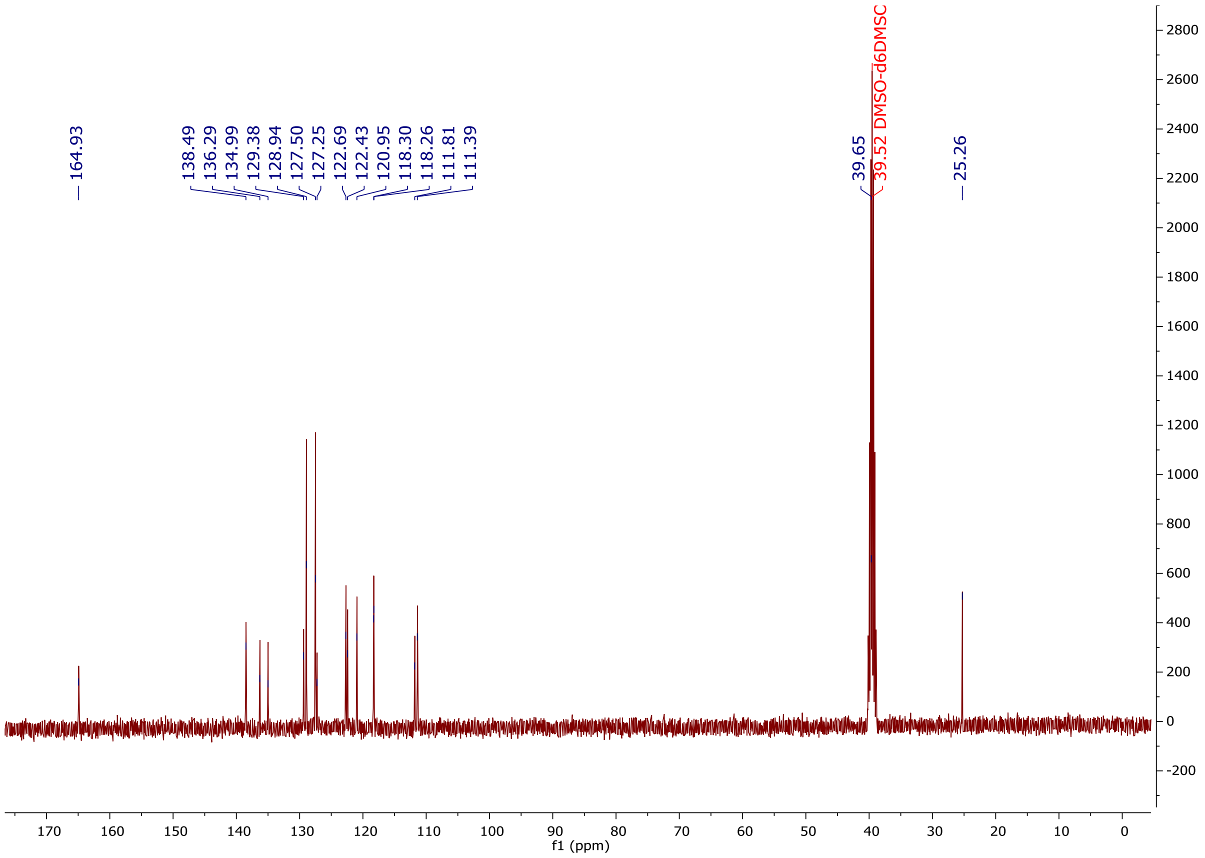

Figure S17. NMR of and ^{13}C (101 MHz) in DMSO-d$_6$ of compound **3f**

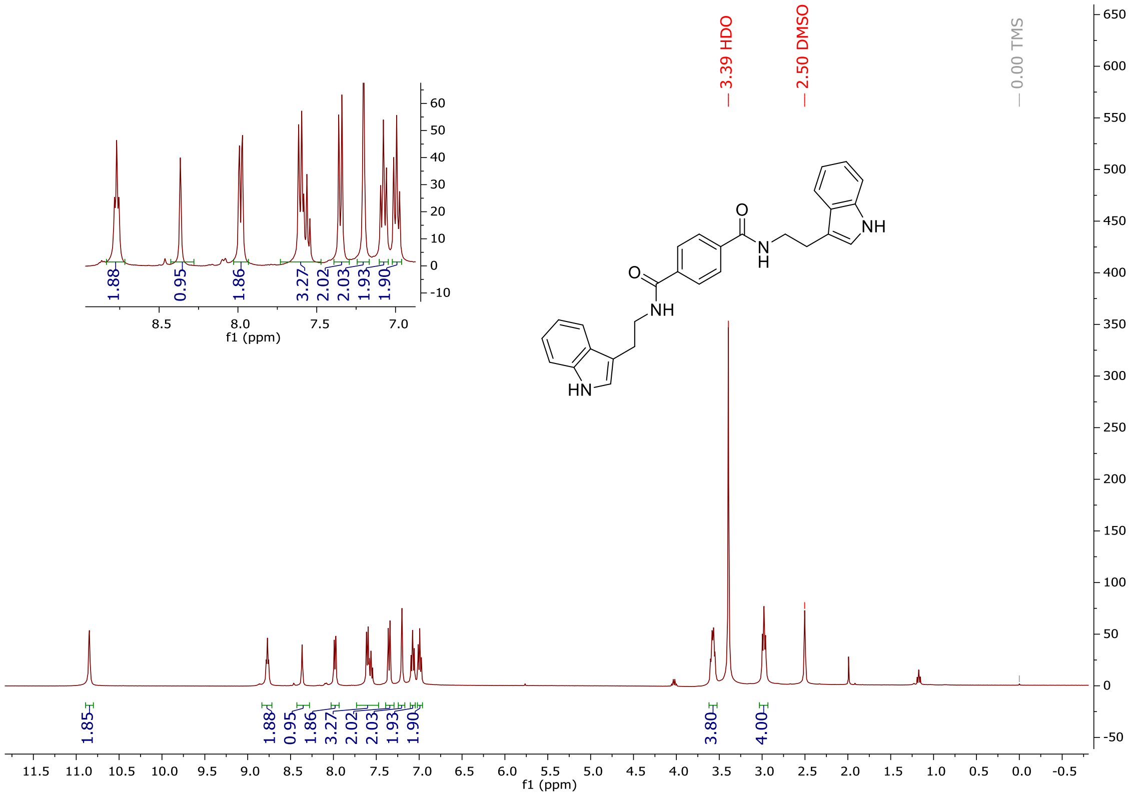

Figure S18. NMR of ^1H (400 MHz) in DMSO-d$_6$ of compound **3g**

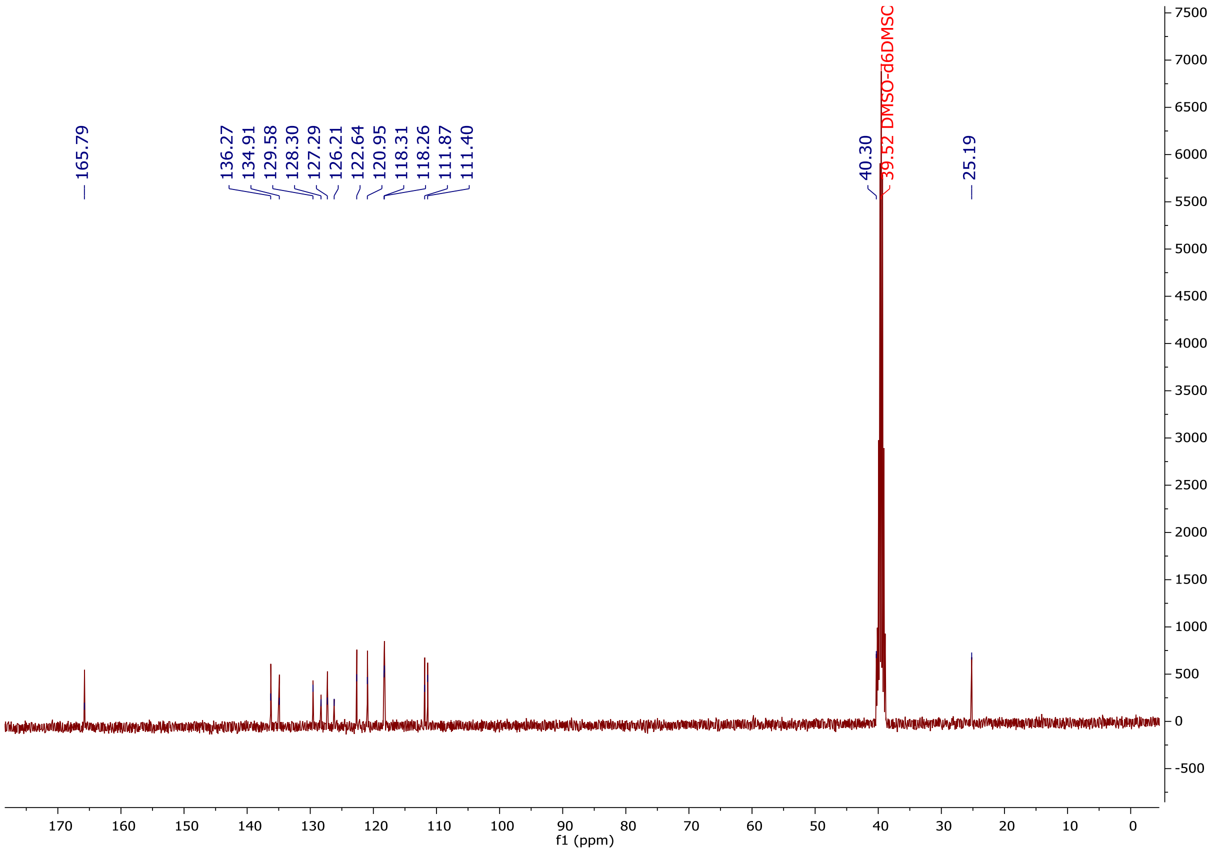

Figure S19. NMR of ^{13}C (101 MHz) in DMSO-d$_6$ of compound **3g**

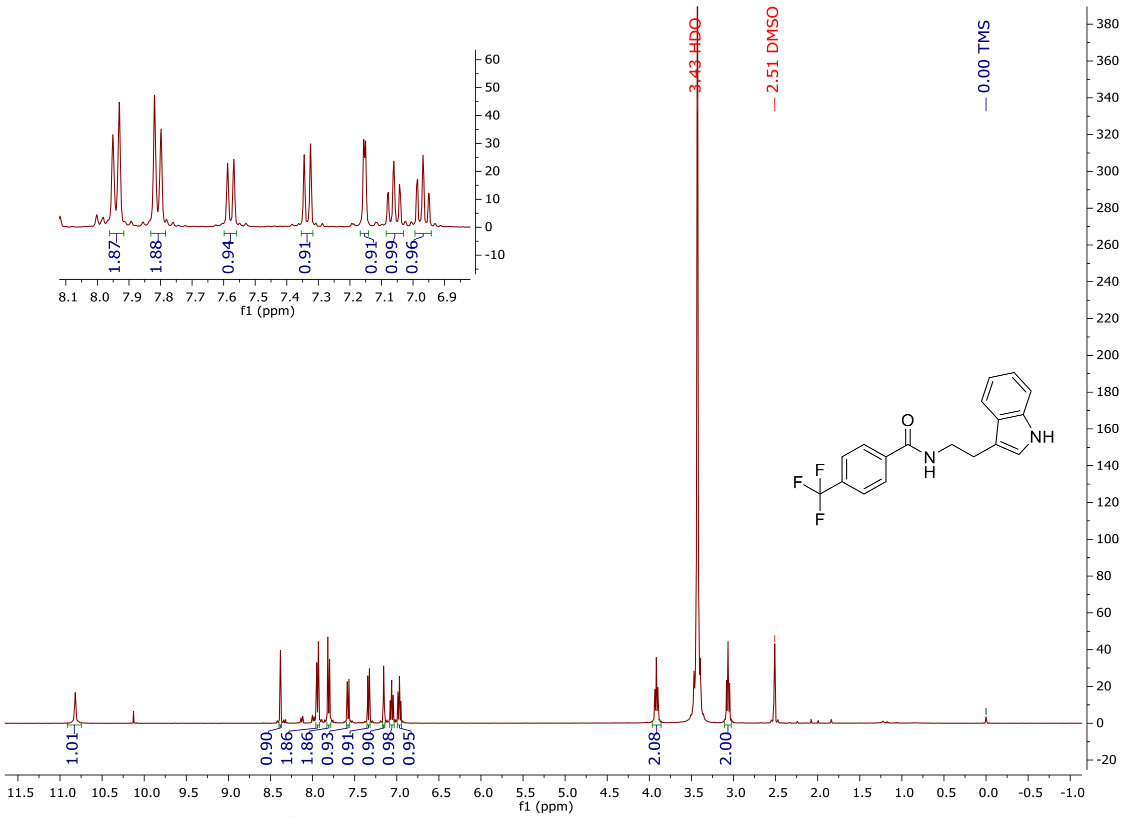

Figure S20. NMR of ^{1}H (400 MHz) in DMSO-d$_6$ of compound **3h**

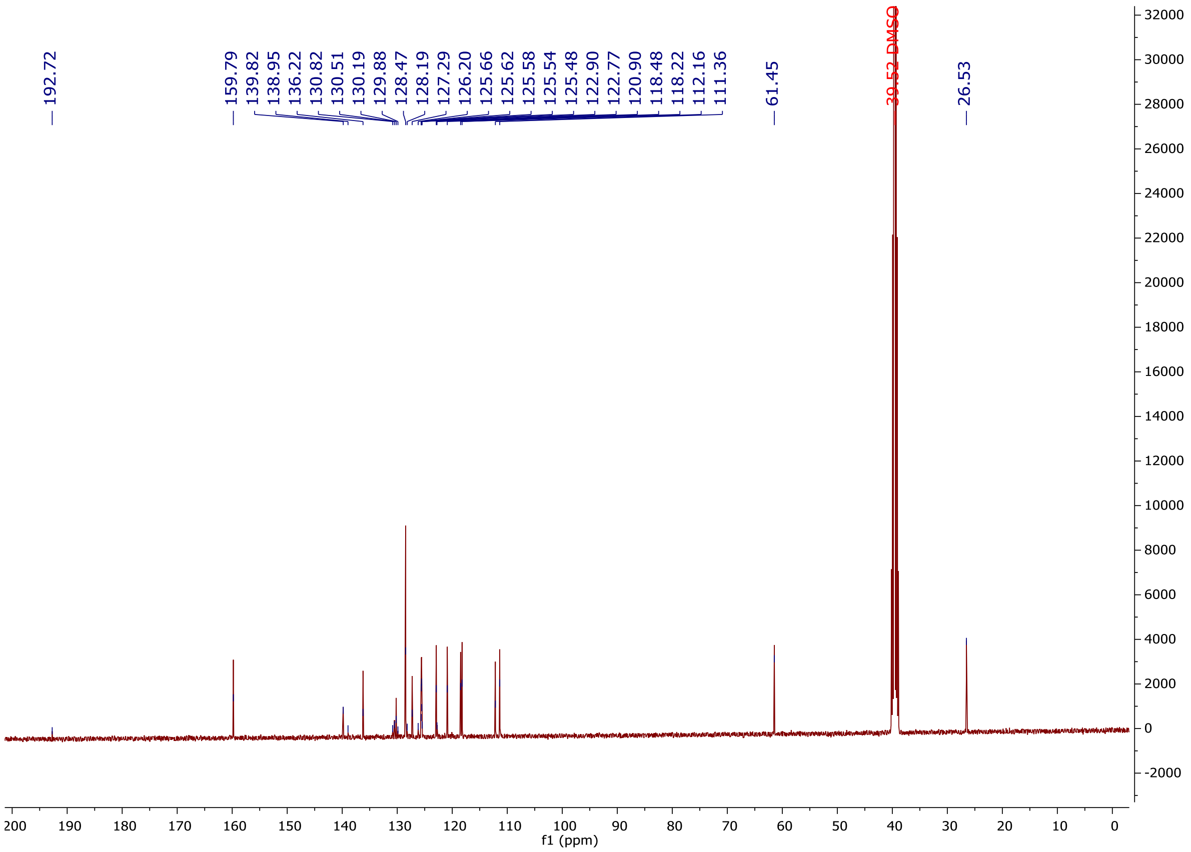

Figure S21. NMR of ^{13}C (101 MHz) in DMSO-d_6 of compound **3h**

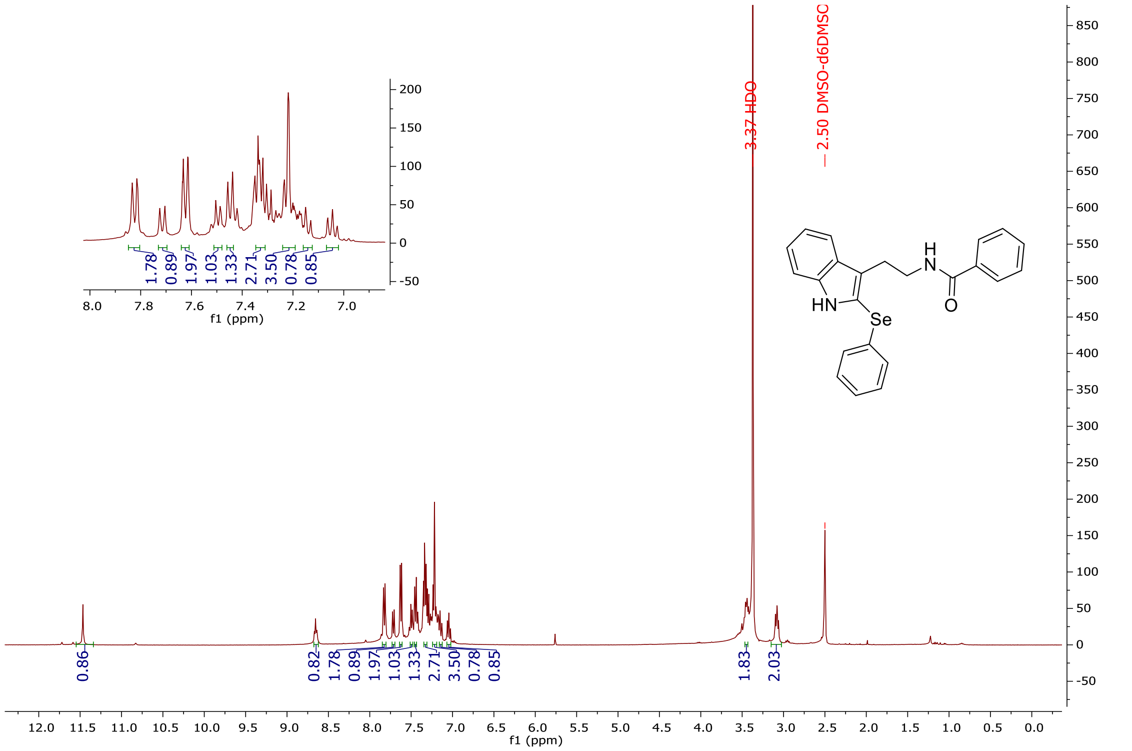

Figure S22. NMR of ^{1}H (400 MHz) in DMSO-d_6 of compound **5a.**

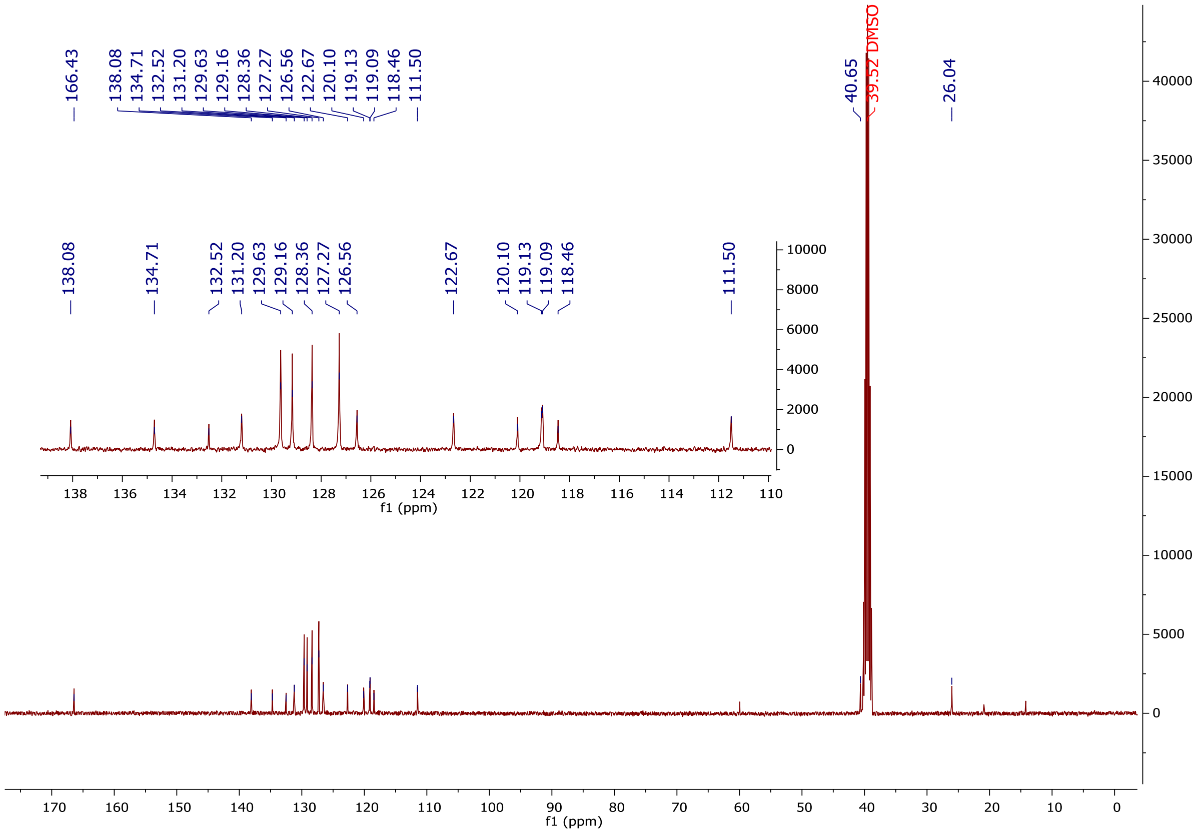

Figure S23. NMR of ^{13}C (101 MHz) in DMSO-d_6 of compound **5a**

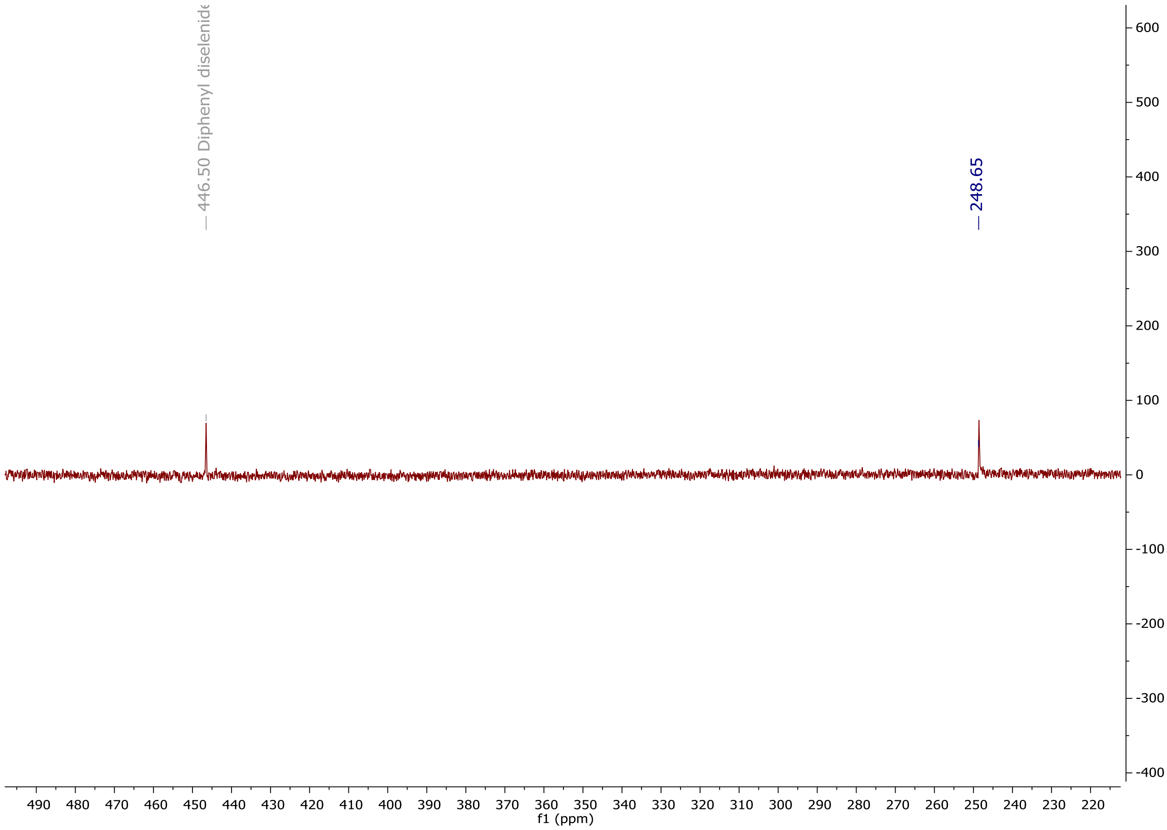

Figure S24. NMR of ^{77}Se (76 MHz) in DMSO-d_6 of compound **5a**

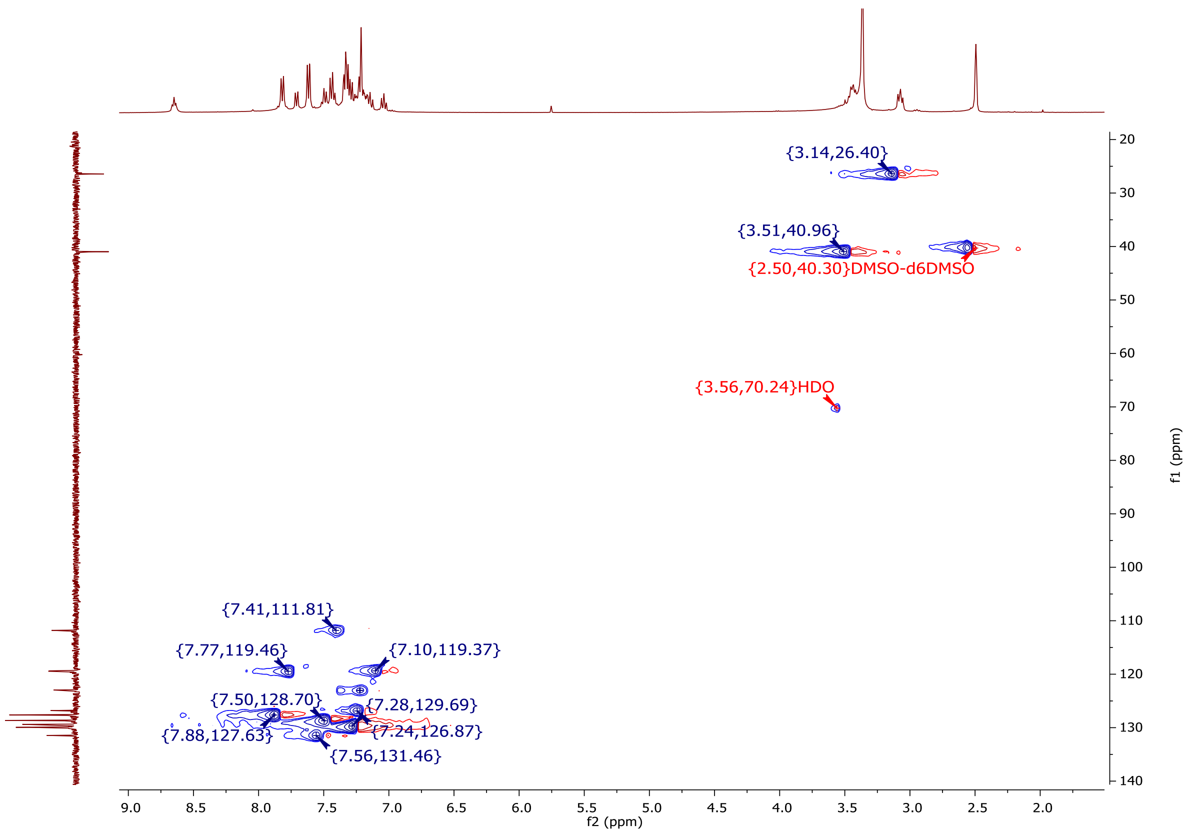

Figure S25. HSQC in DMSO-d6 of compound **5a**

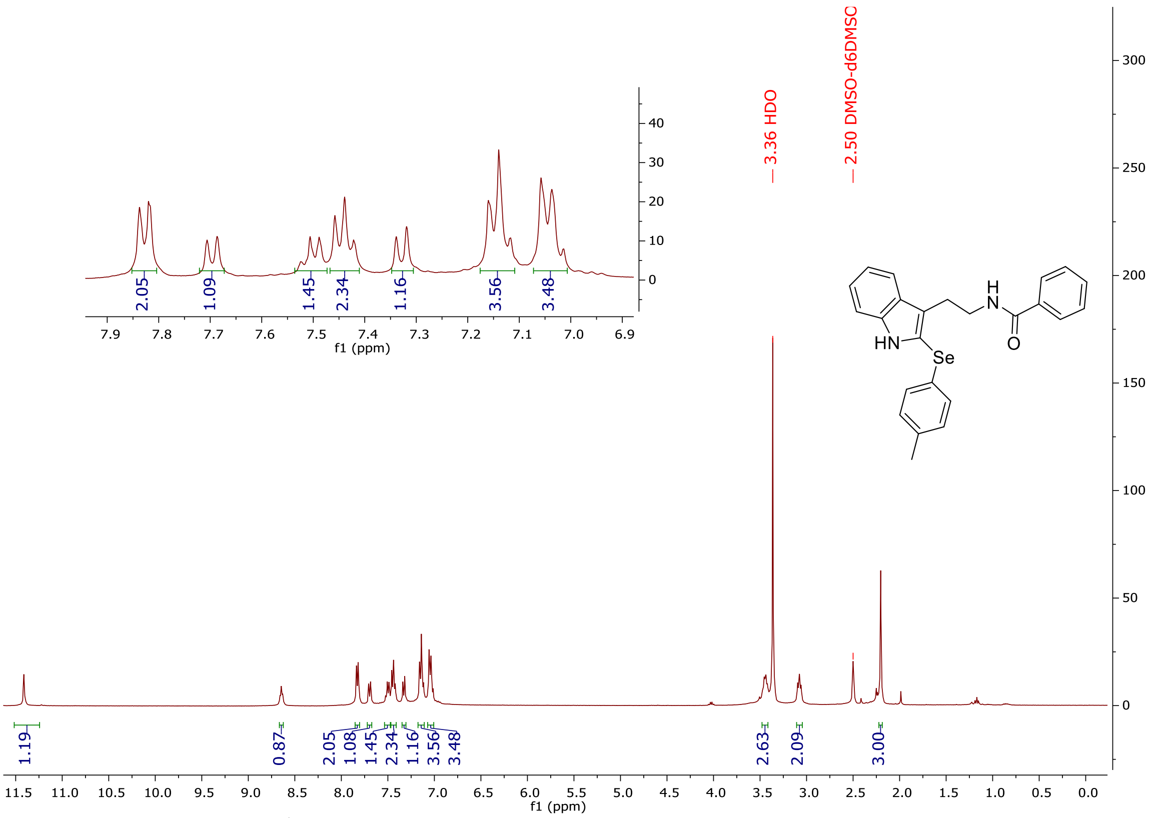

Figure S26. NMR of ^{1}H (400 MHz) in DMSO-d_6 of compound **5b**

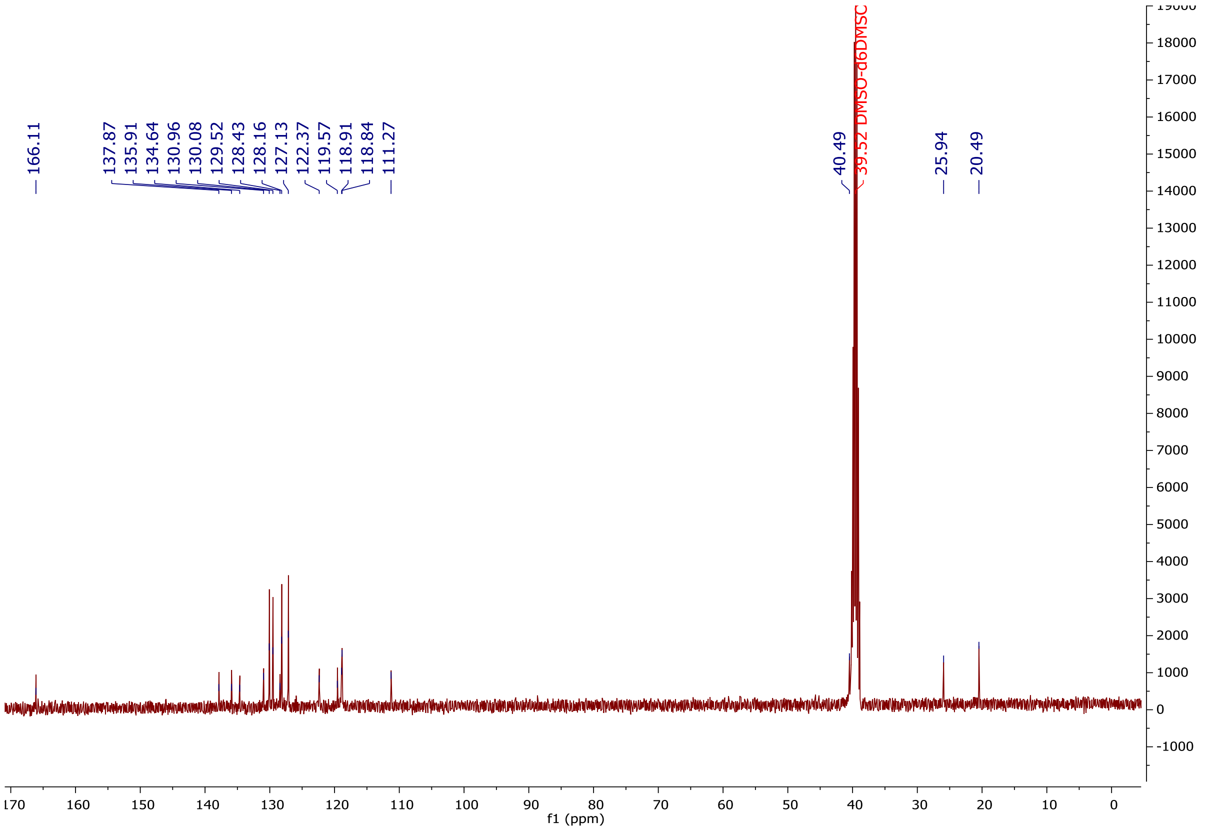

Figure S27. NMR of ^{13}H (101 MHz) in DMSO-d$_6$ of compound **5b**

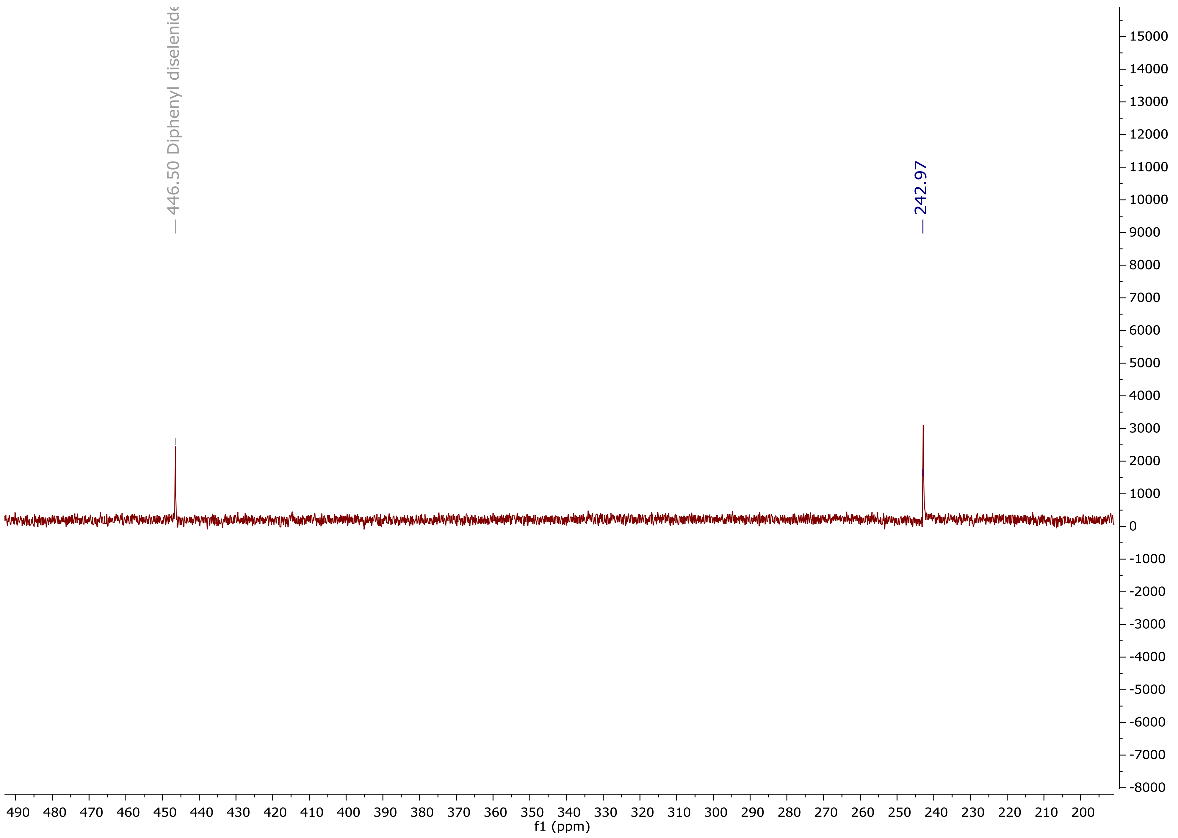

Figure S28. NMR of ^{77}Se (76 MHz) in DMSO-d$_6$ of compound **5b**

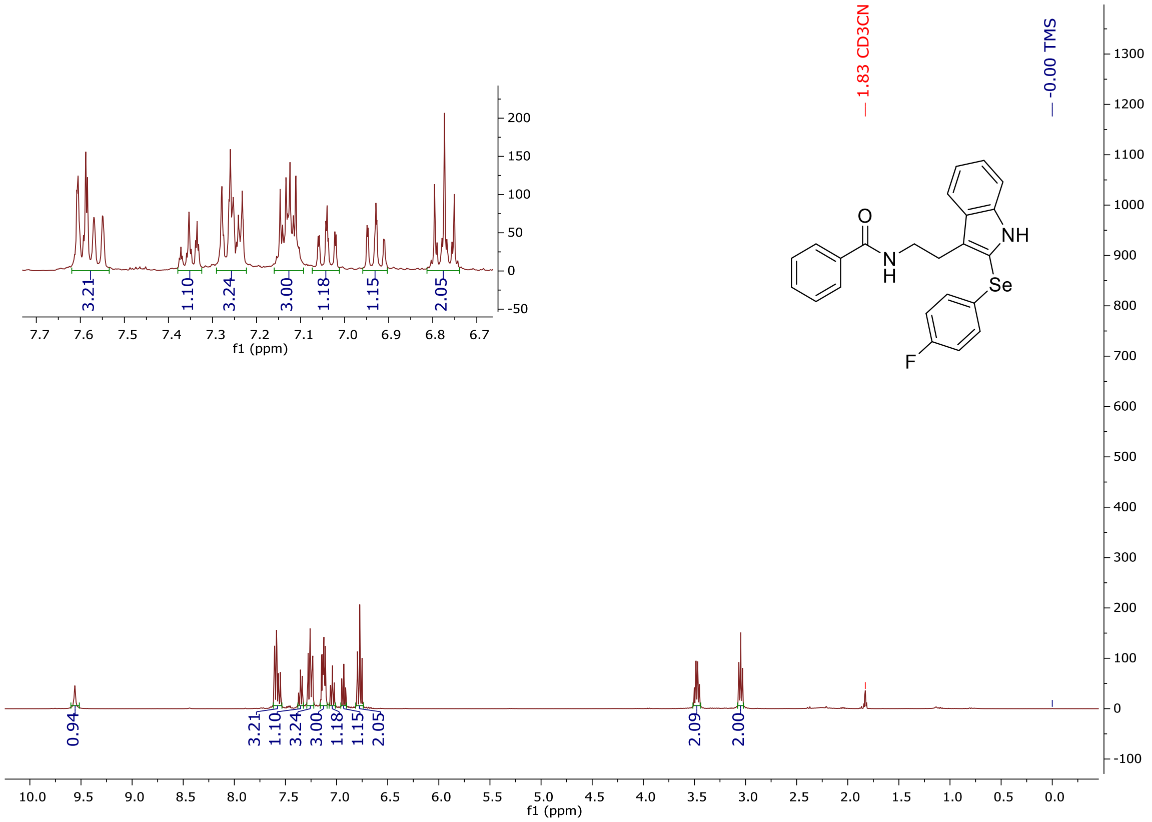

Figure S29. NMR of ^{1}H (400 MHz) in Acetonitrile -d_3 of compound **5c**

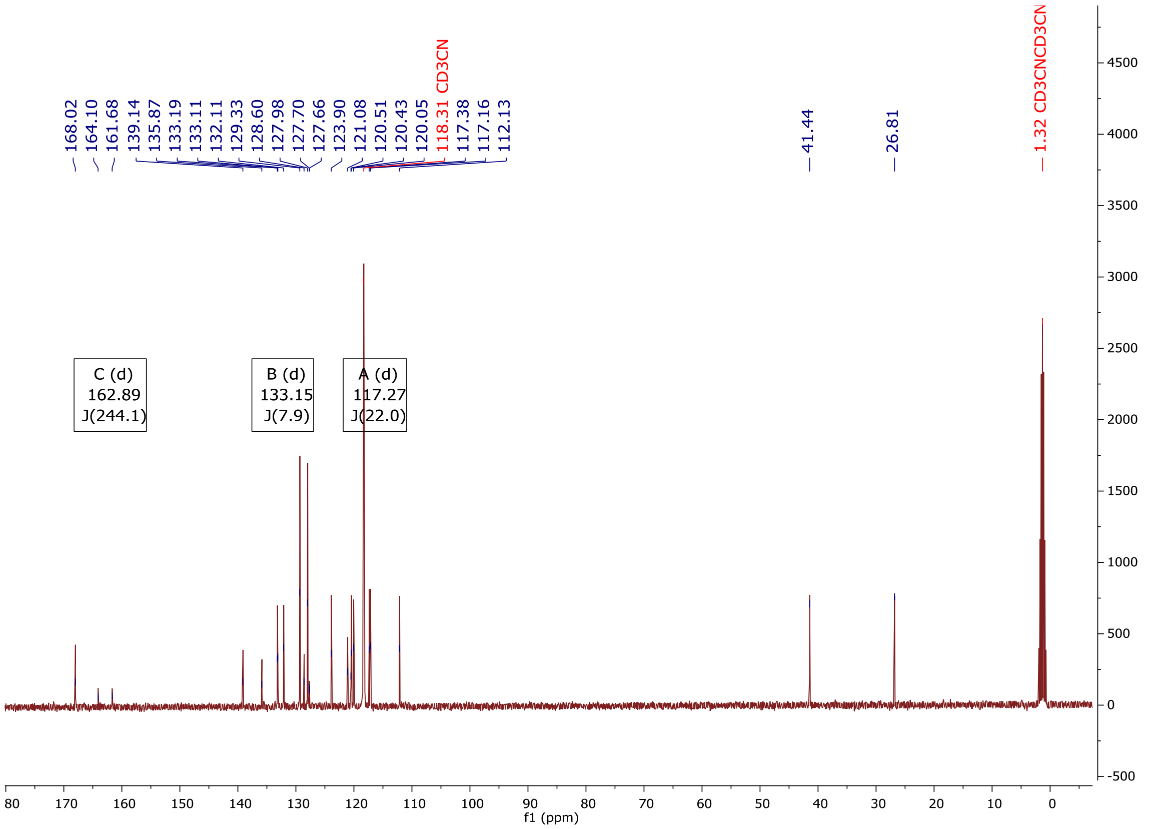

Figure S30. NMR of ^{13}C (101 MHz) in Acetonitrile -d_3 of compound **5c**

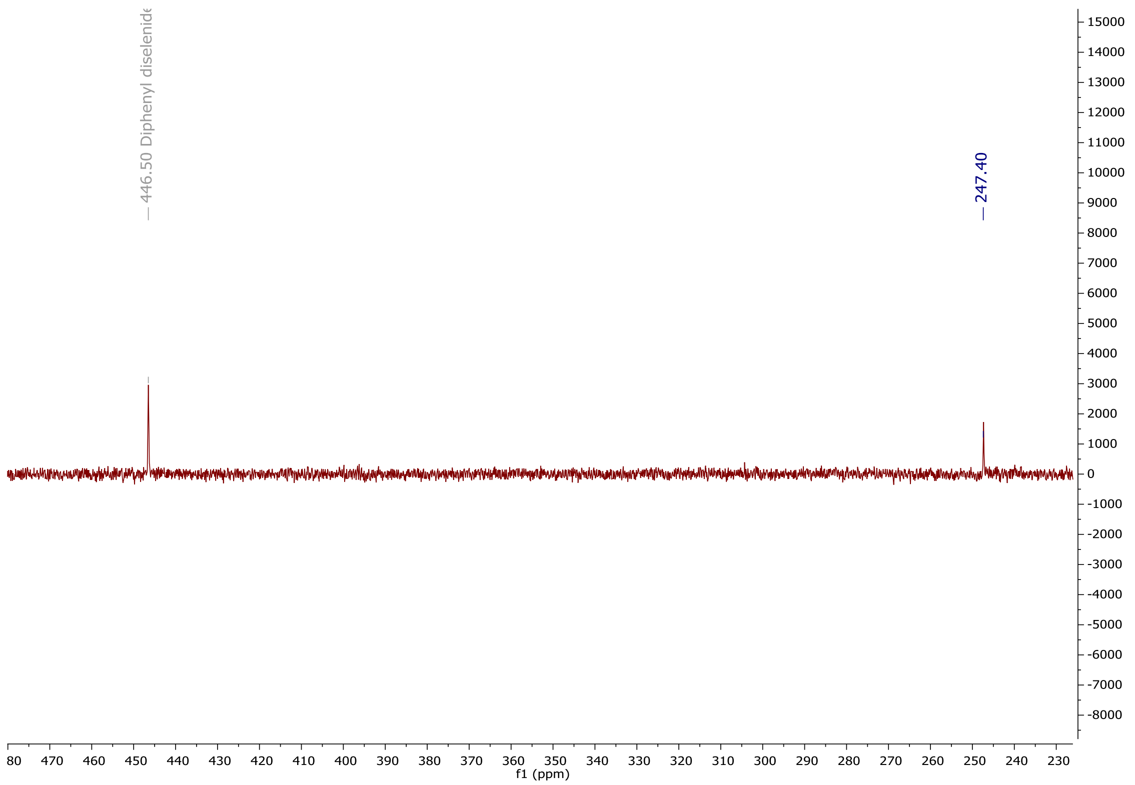

Figure S31. NMR of ^{77}Se (76 MHz) in Acetonitrile -d_3 of compound **5c**

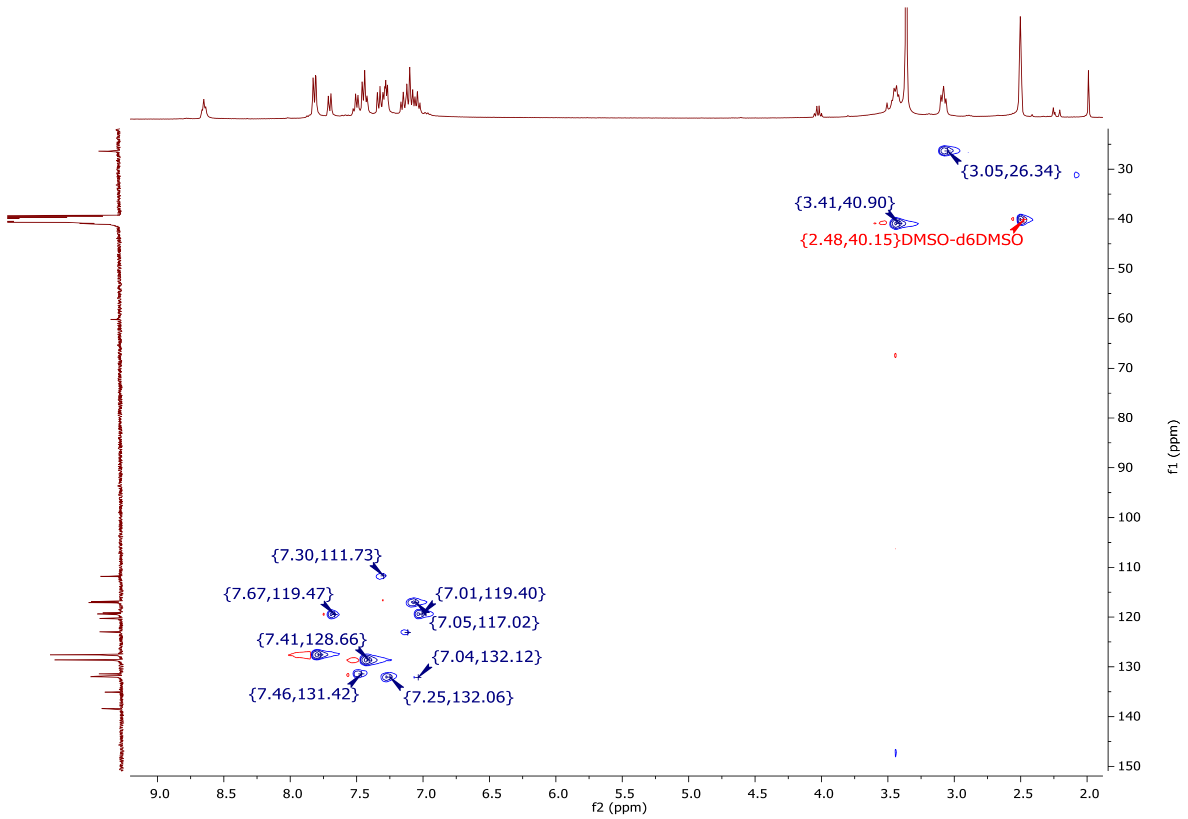

Figure S32. HSQC in Acetonitrile -d_3 of compound **5c**

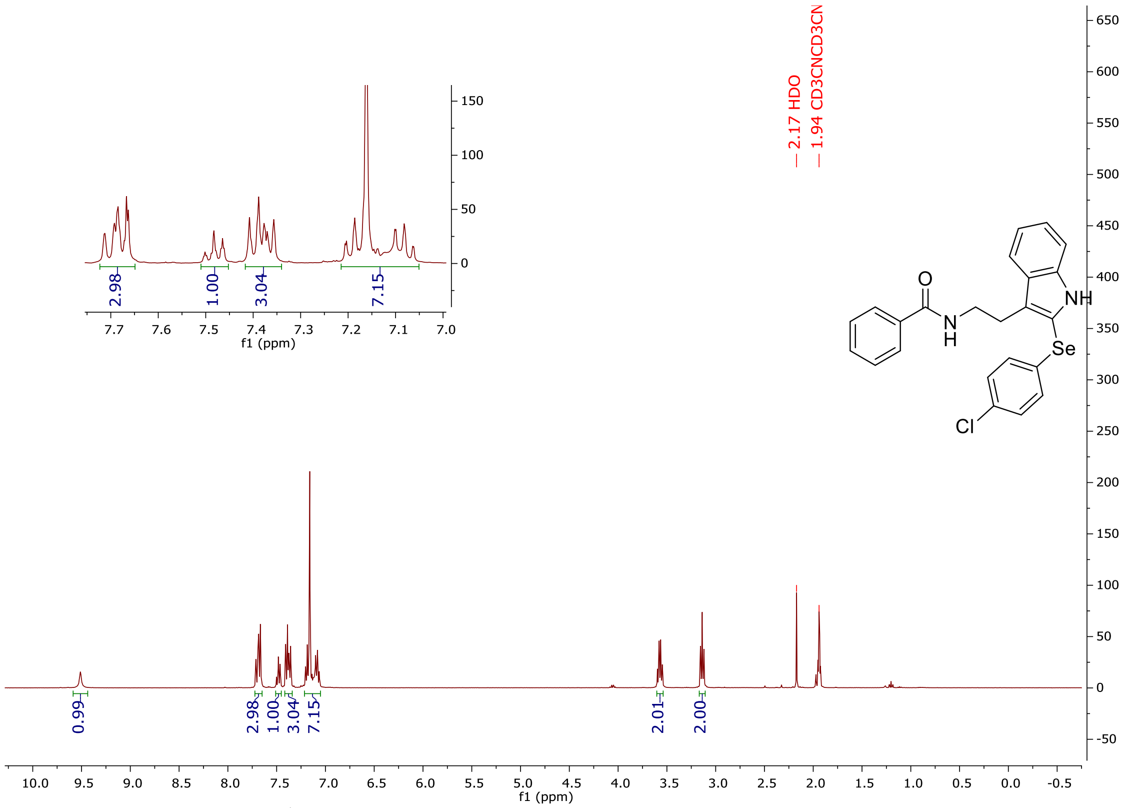

Figure S33. NMR of ^{1}H (400 MHz) in Acetonitrile -d$_3$ of compound **5d**

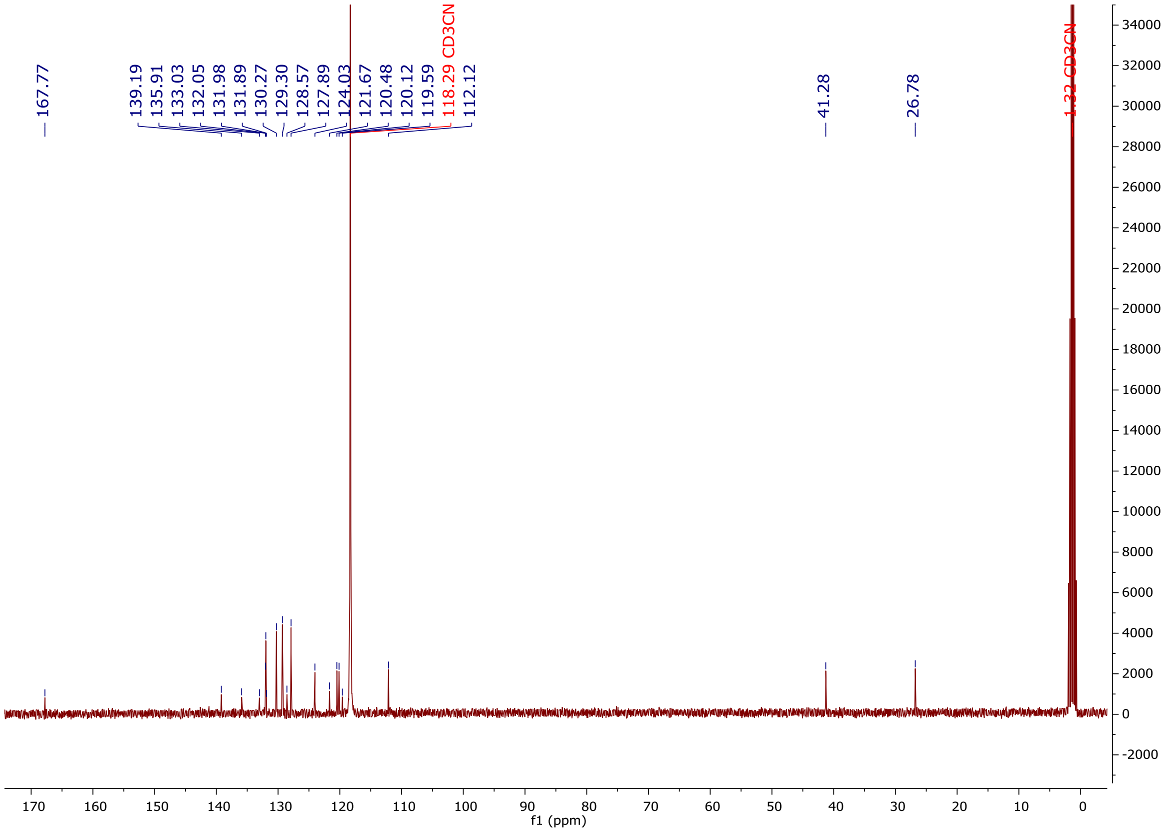

Figure S34. NMR of ^{13}C (101 MHz) in Acetonitrile -d$_3$ of compound **5d**

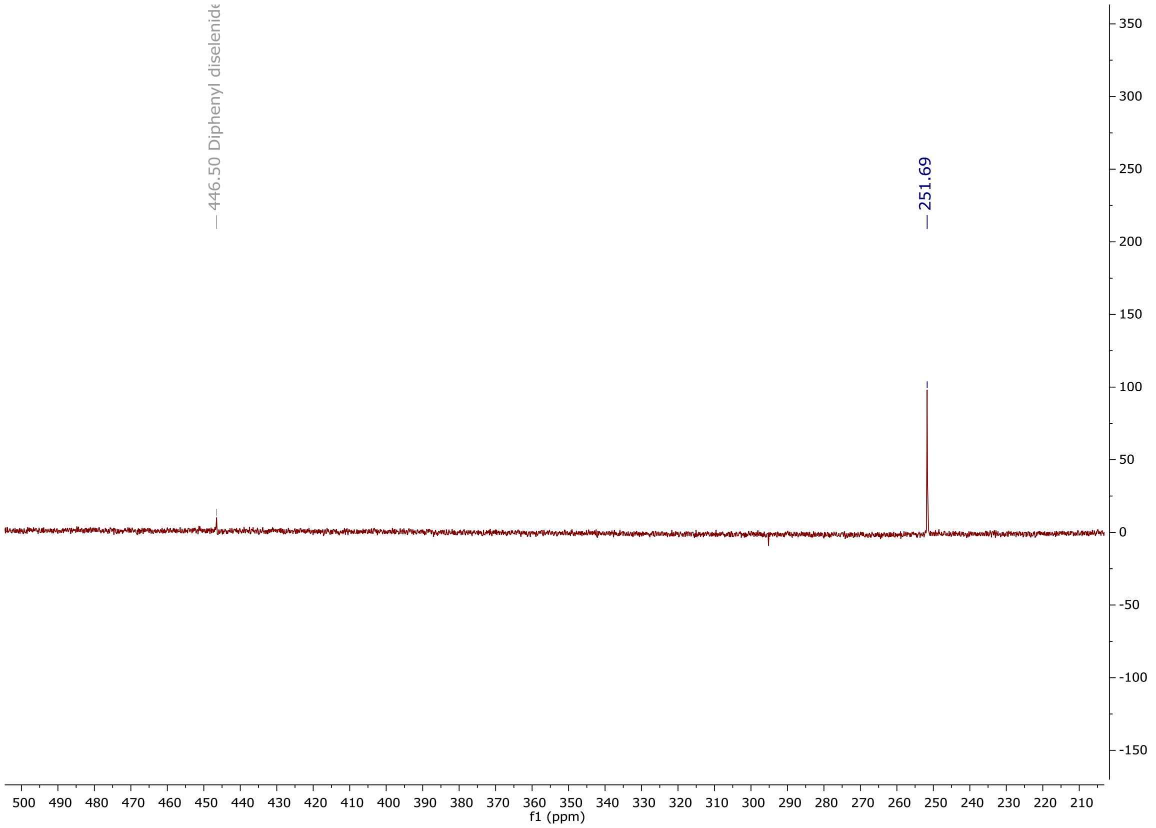

Figure S35. NMR of ^{77}Se (76 MHz) in Acetonitrile -d$_3$ of compound **5d**

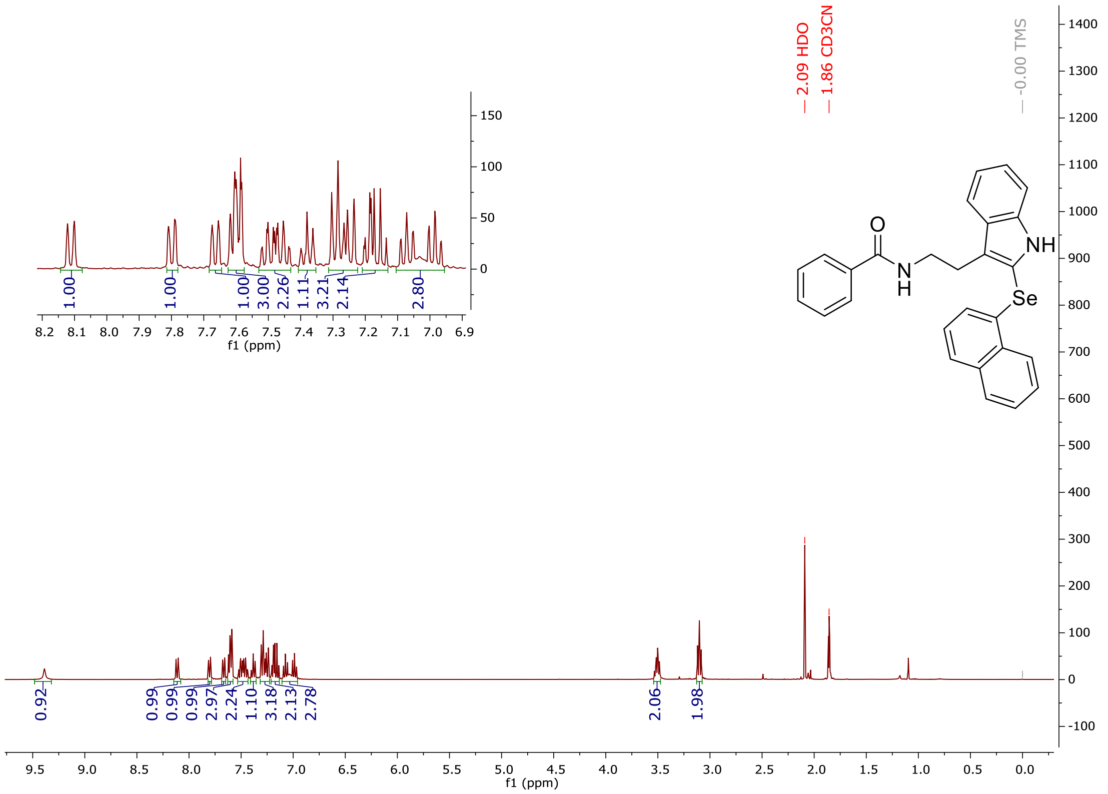

Figure S36. NMR of ^{1}H (400 MHz) in Acetonitrile -d$_3$ of compound **5e**

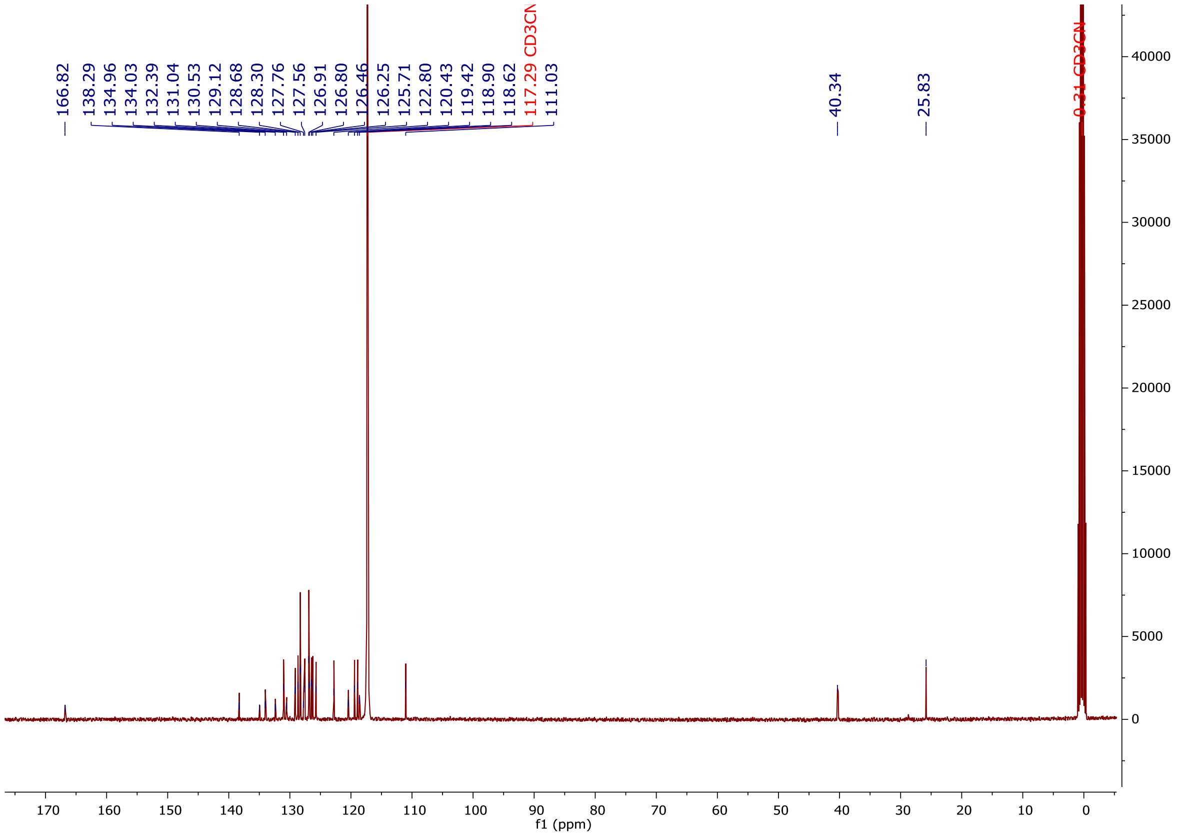

Figure S37. NMR of ^{13}C (101 MHz) in Acetonitrile -d_3 of compound **5e**

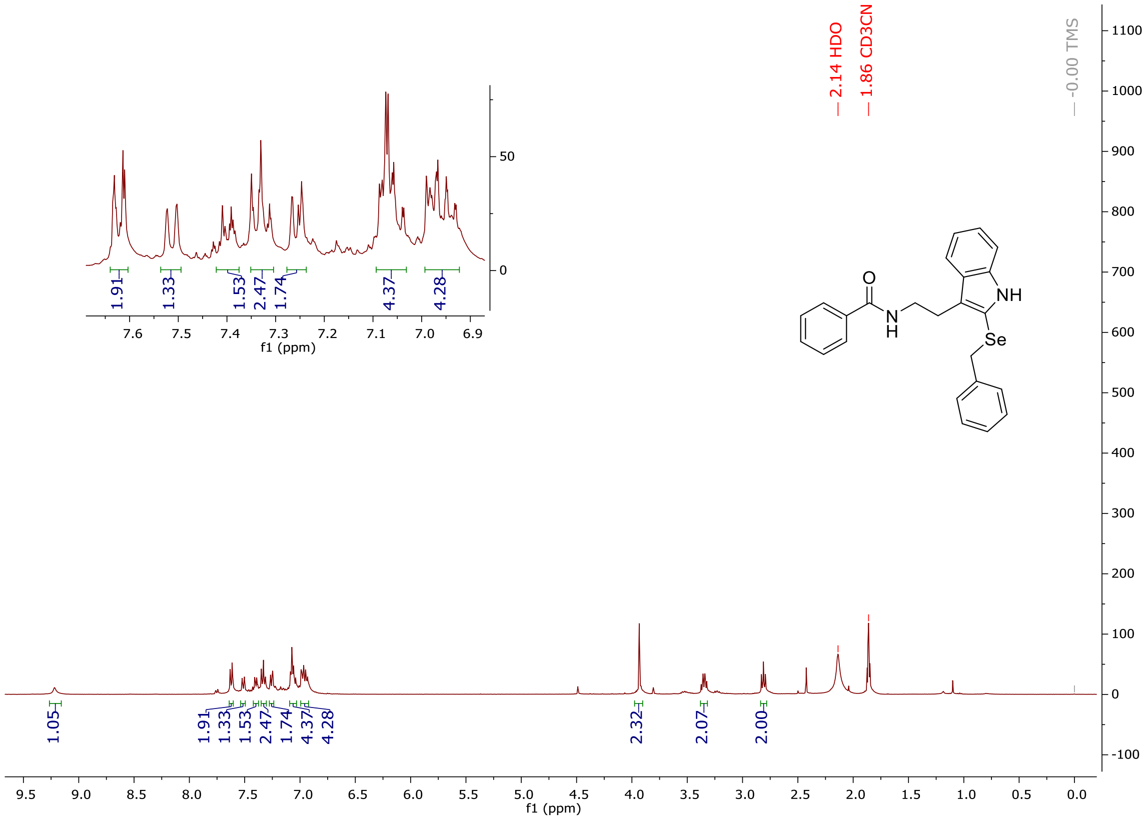

Figure S38. NMR of ^{1}H (400 MHz) in Acetonitrile -d_3 of compound **5f**

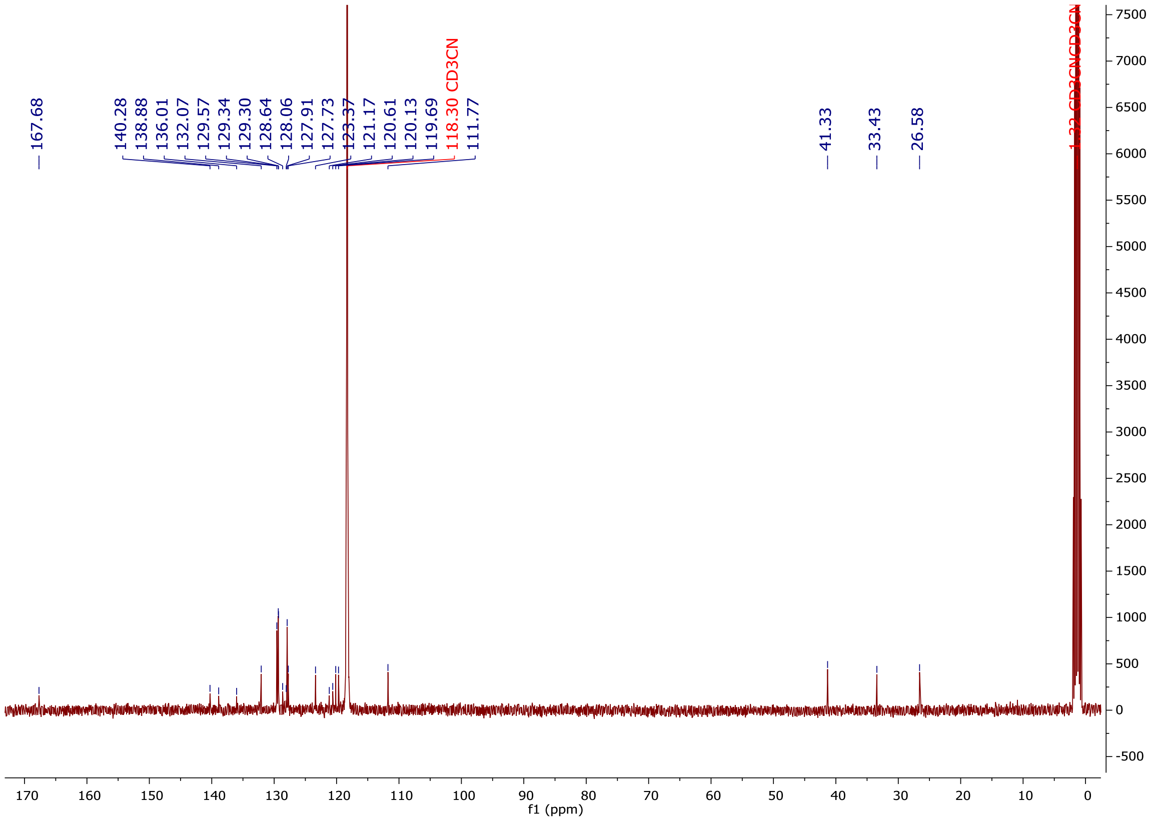

Figure S39. NMR of ^{13}C (101 MHz) in Acetonitrile -d_3 of compound **5f**

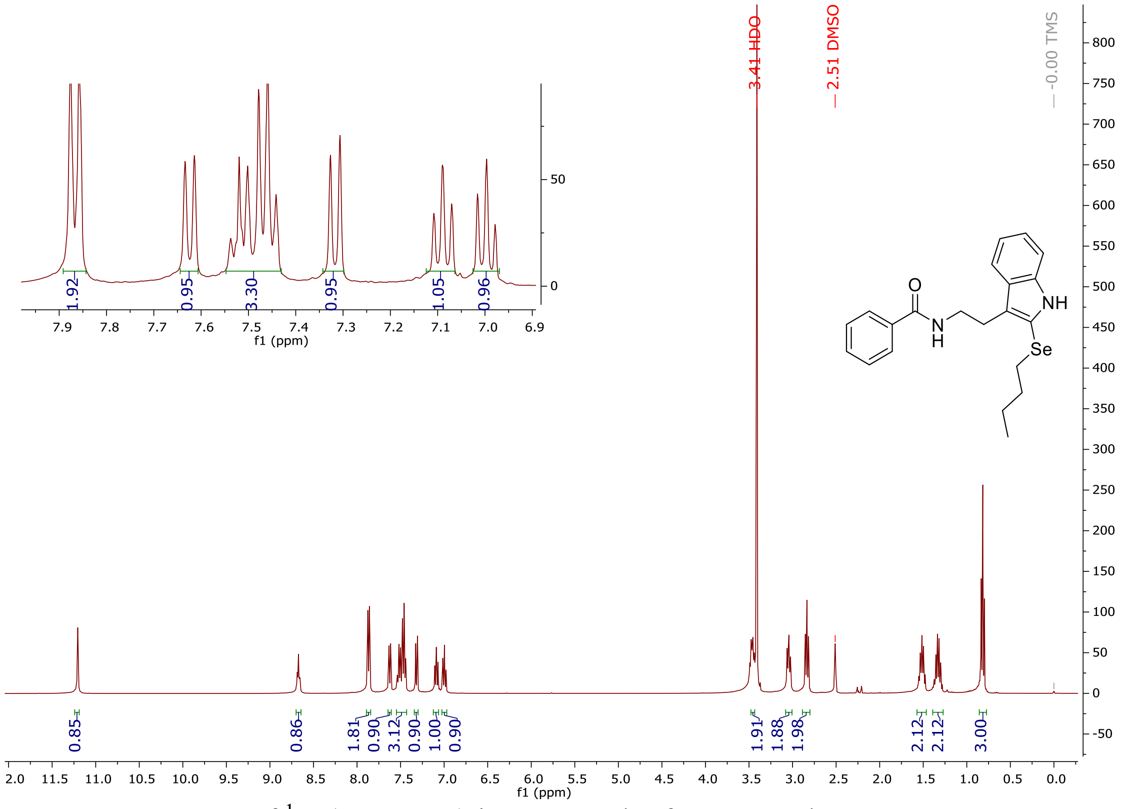

Figure S40. NMR of ^{1}H (400 MHz) in DMSO-d_6 of compound **5g**

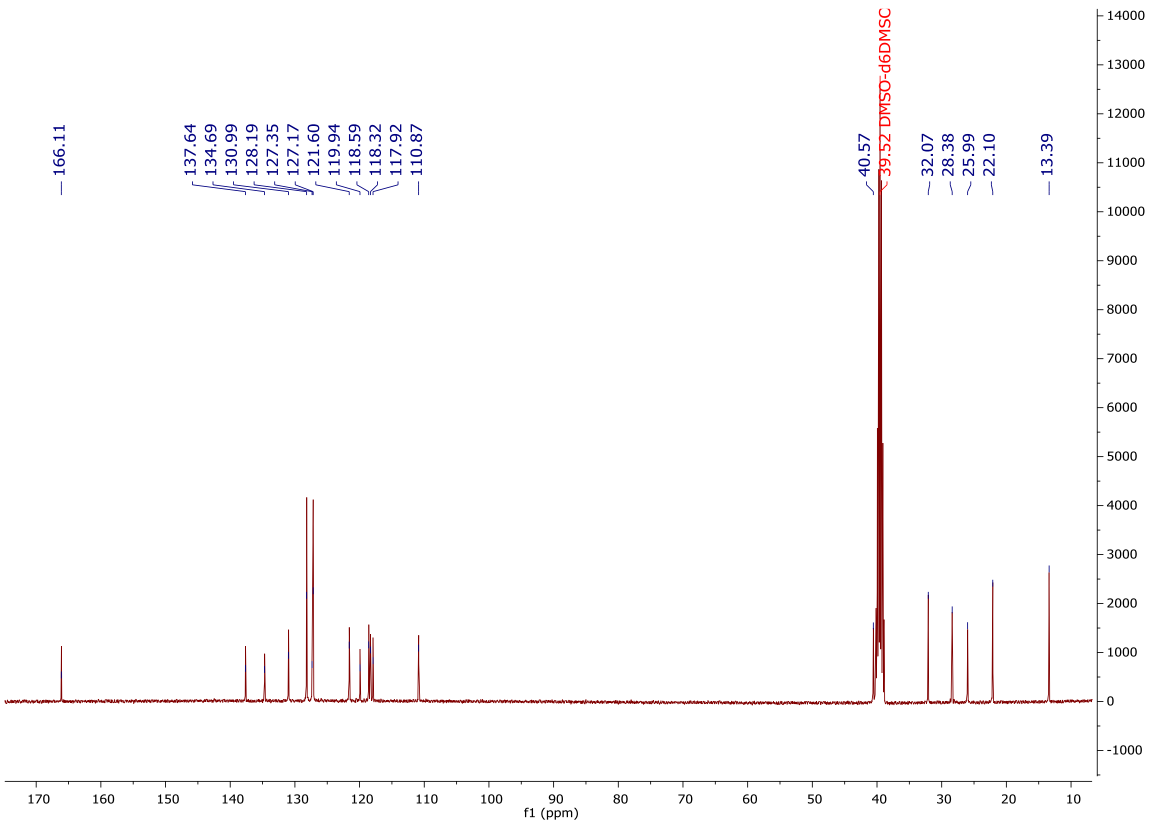

Figure S41. NMR of ^{13}C (101 MHz) in DMSO-d_6 of compound **5g**

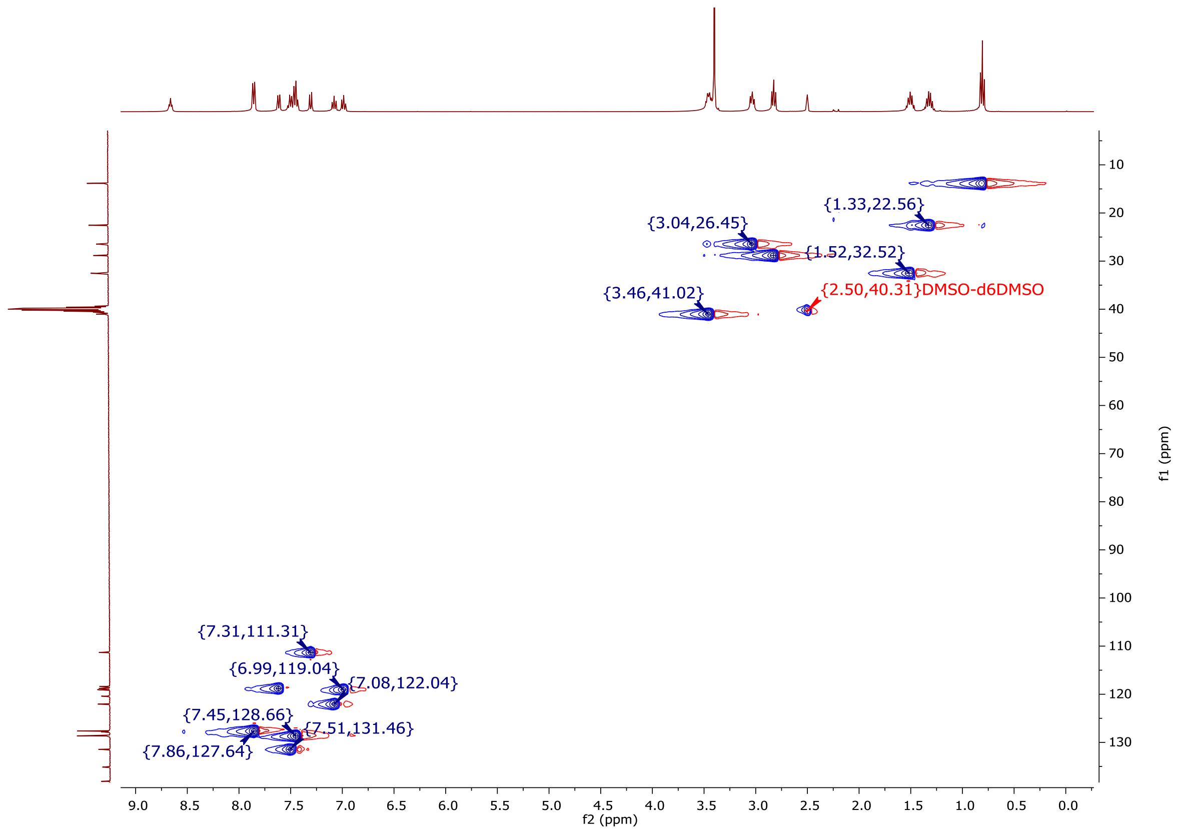

Figure S42. HSQC in DMSO-d_6 of compound **5g**

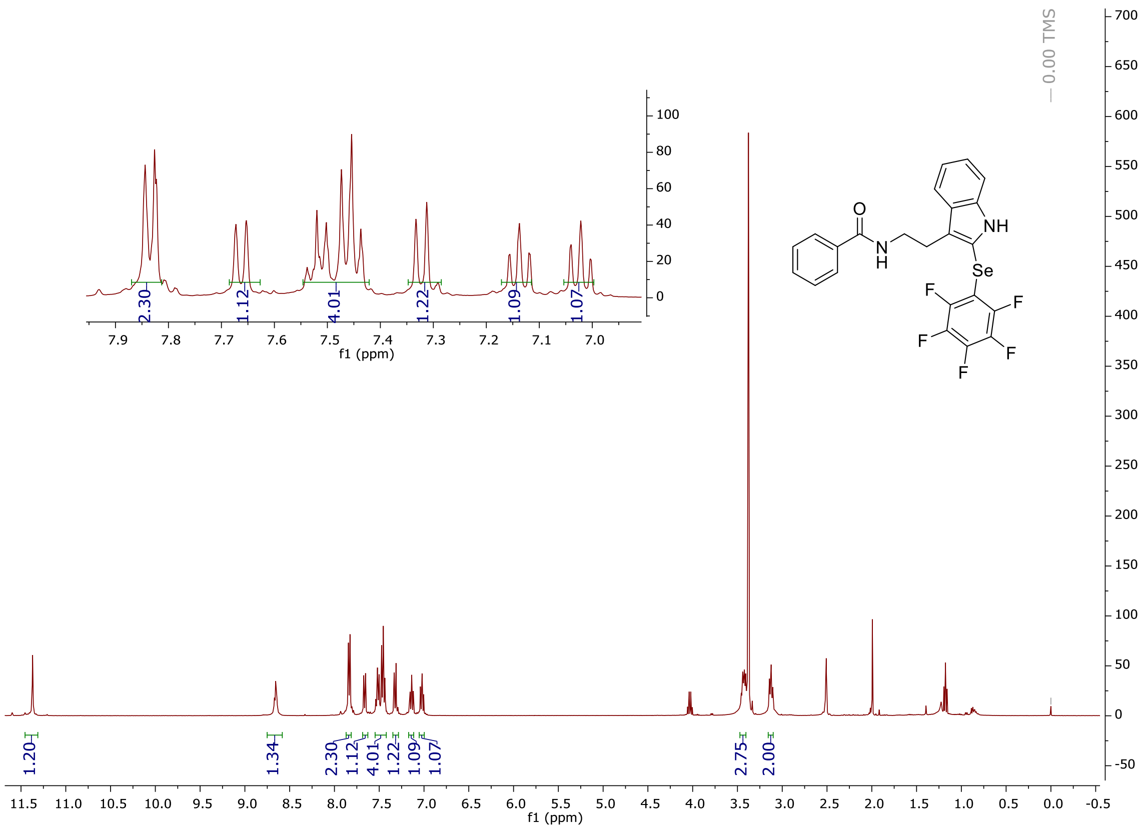

Figure S43. NMR of ^{1}H (400 MHz) in DMSO-d_6 of compound **5h**

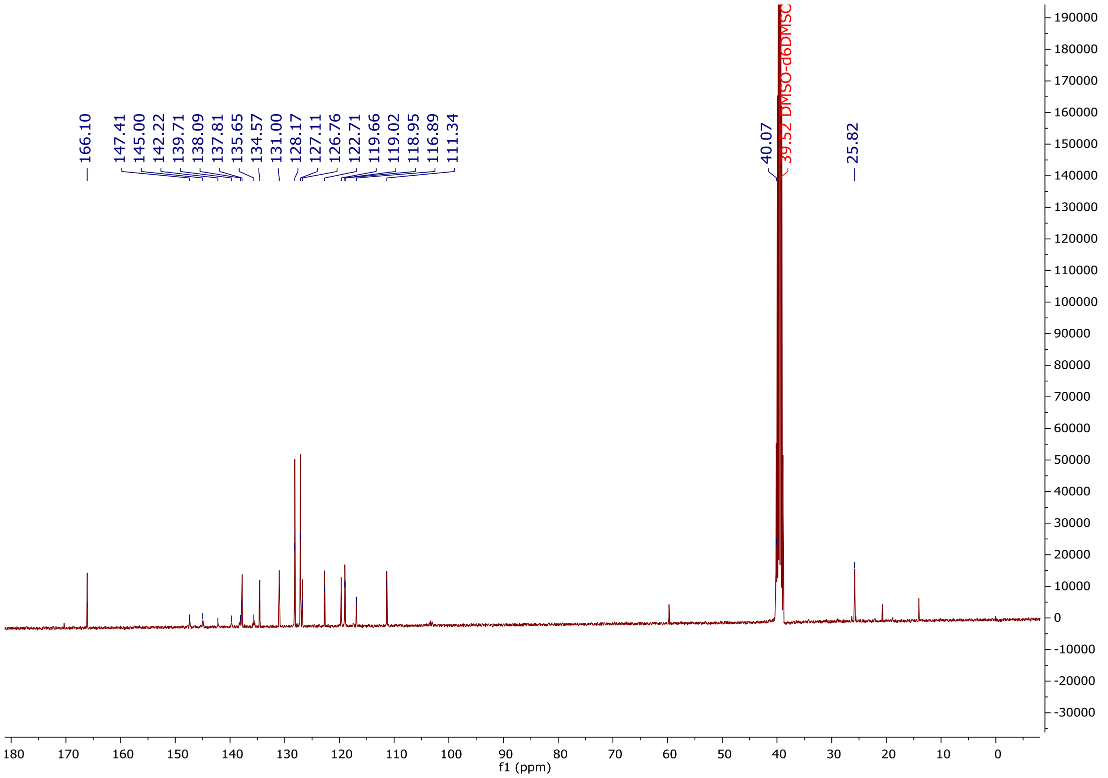

Figure S44. NMR of ^{13}C (101 MHz) in DMSO-d_6 of compound **5h**

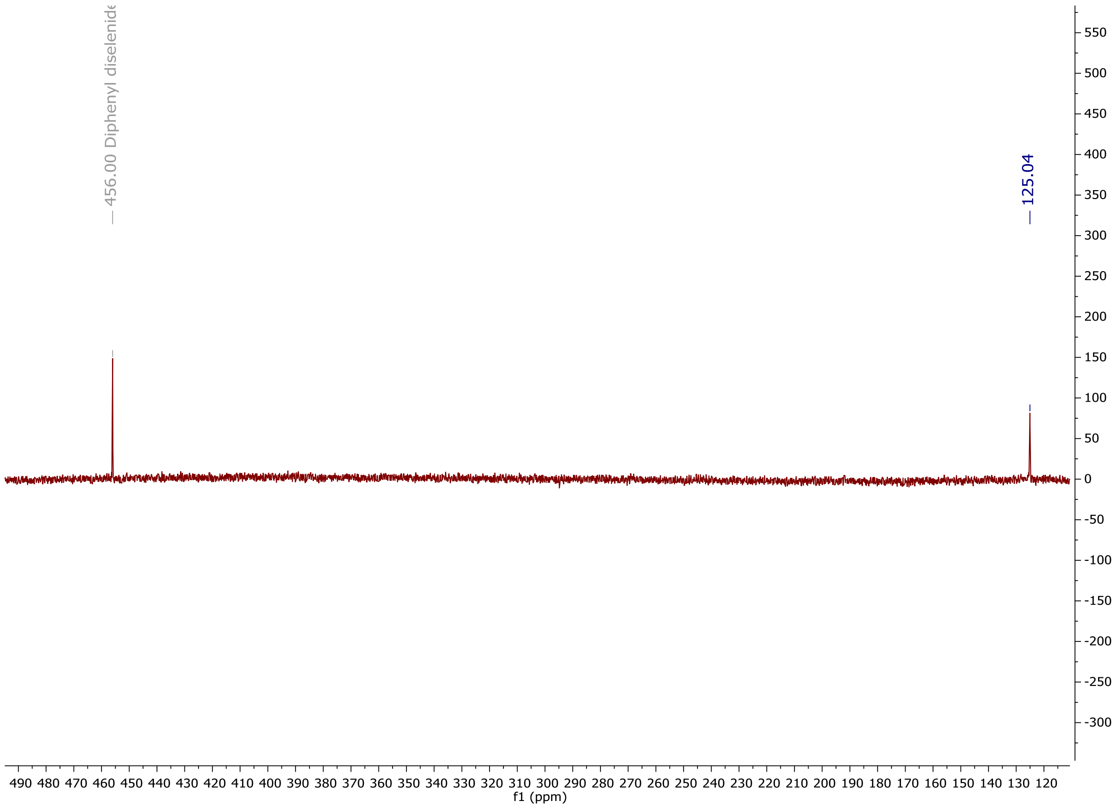

Figure S45. NMR of ^{77}Se (76 MHz) in DMSO-d_6 of compound **5h**

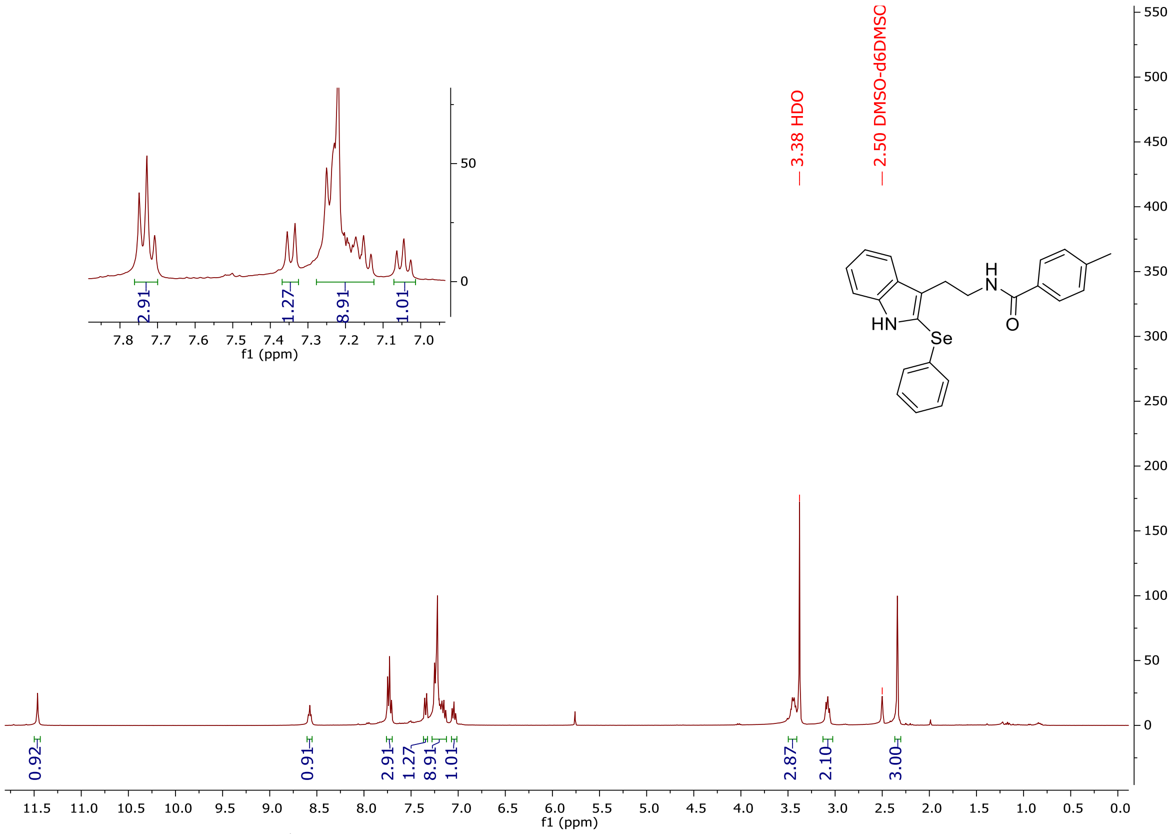

Figure S46. NMR of ^{1}H (400 MHz) in DMSO-d_6 of compound **5i**

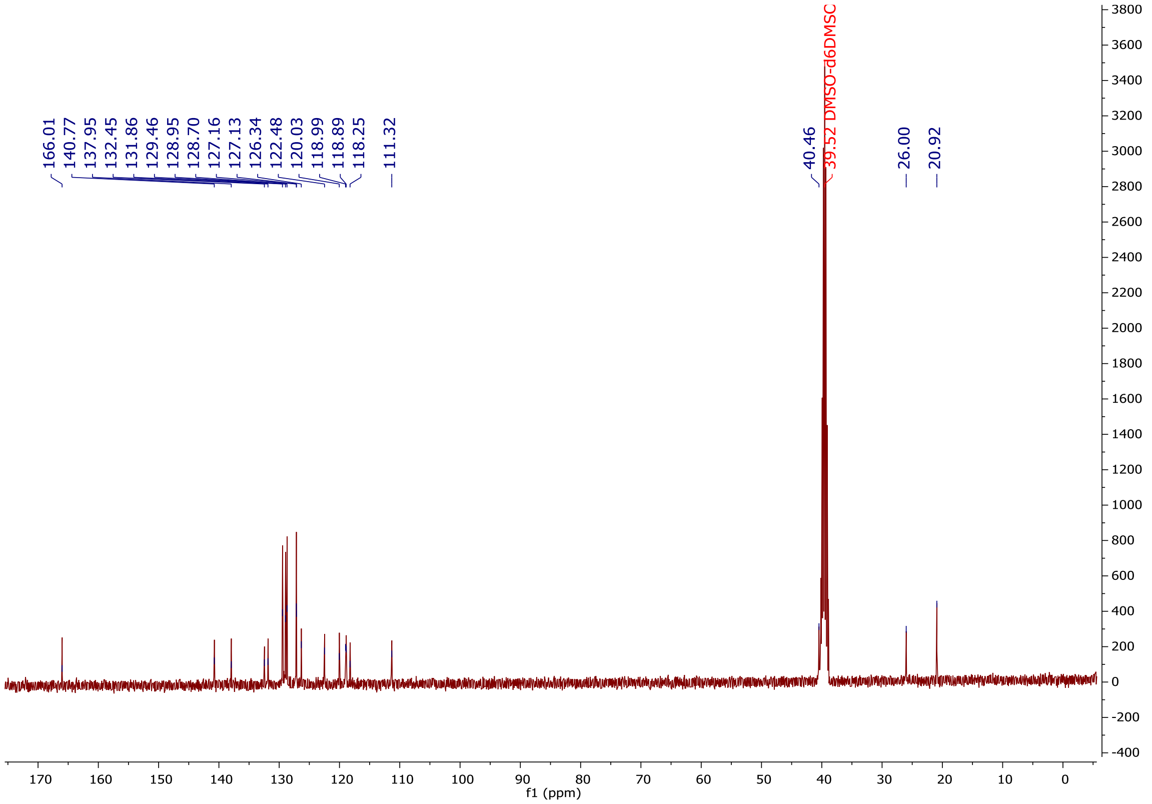

Figure S47. NMR of ^{13}C (101 MHz) in DMSO-d_6 of compound **5i**

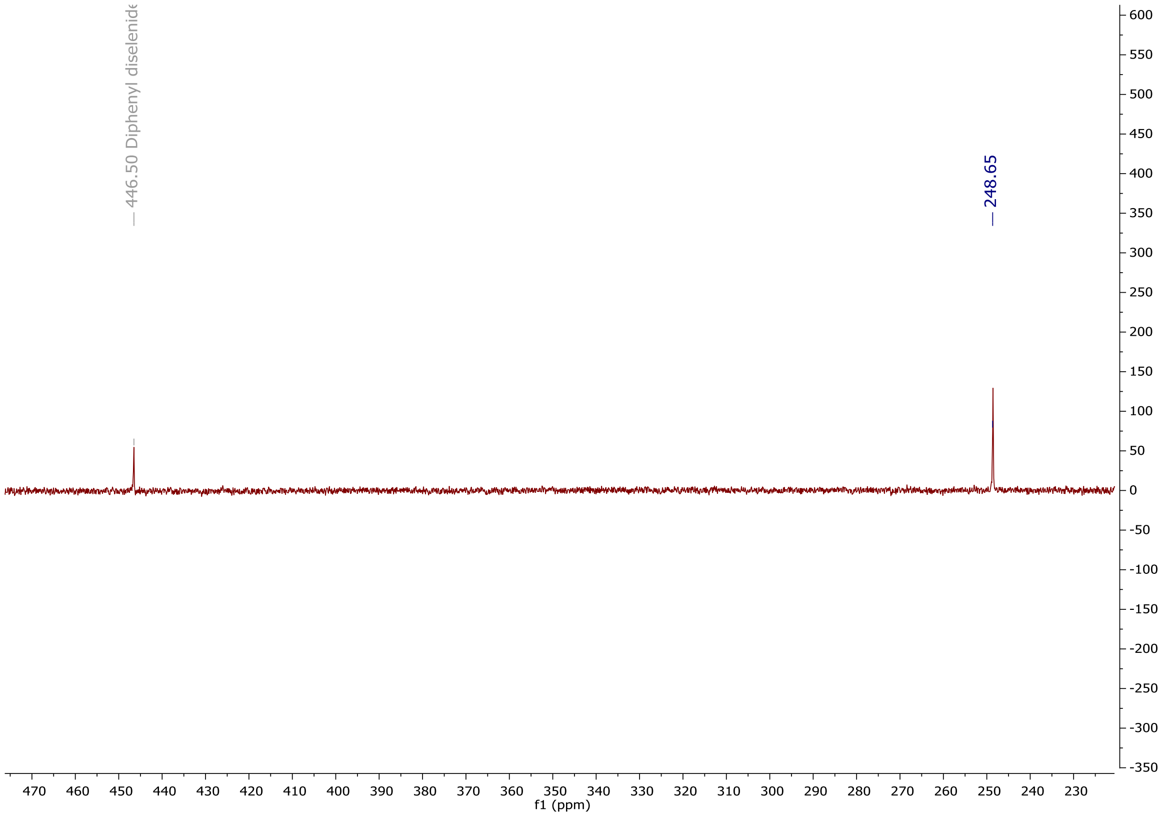

Figure S48. NMR of ^{77}Se (76 MHz) in DMSO-d_6 of compound **5i**

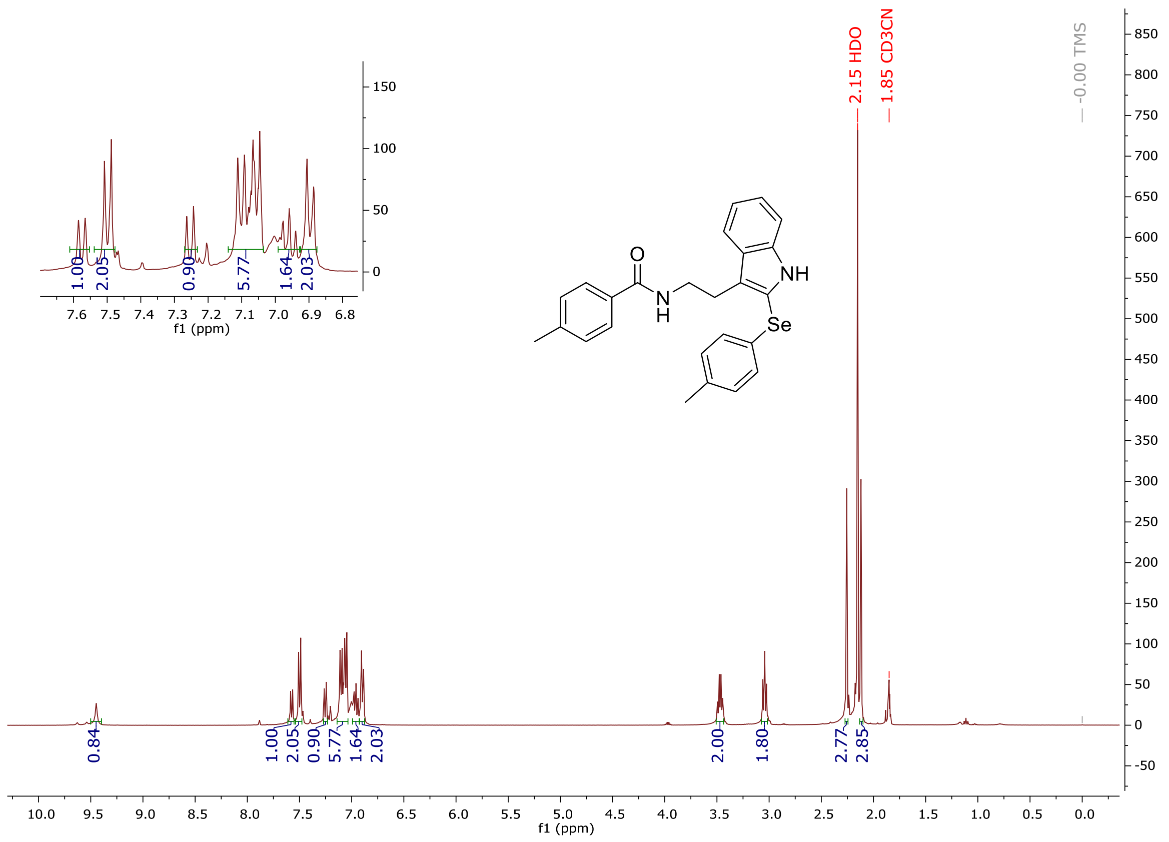

Figure S49. NMR of ^{1}H (400 MHz) in Acetonitrile-d_3 of compound **5j**

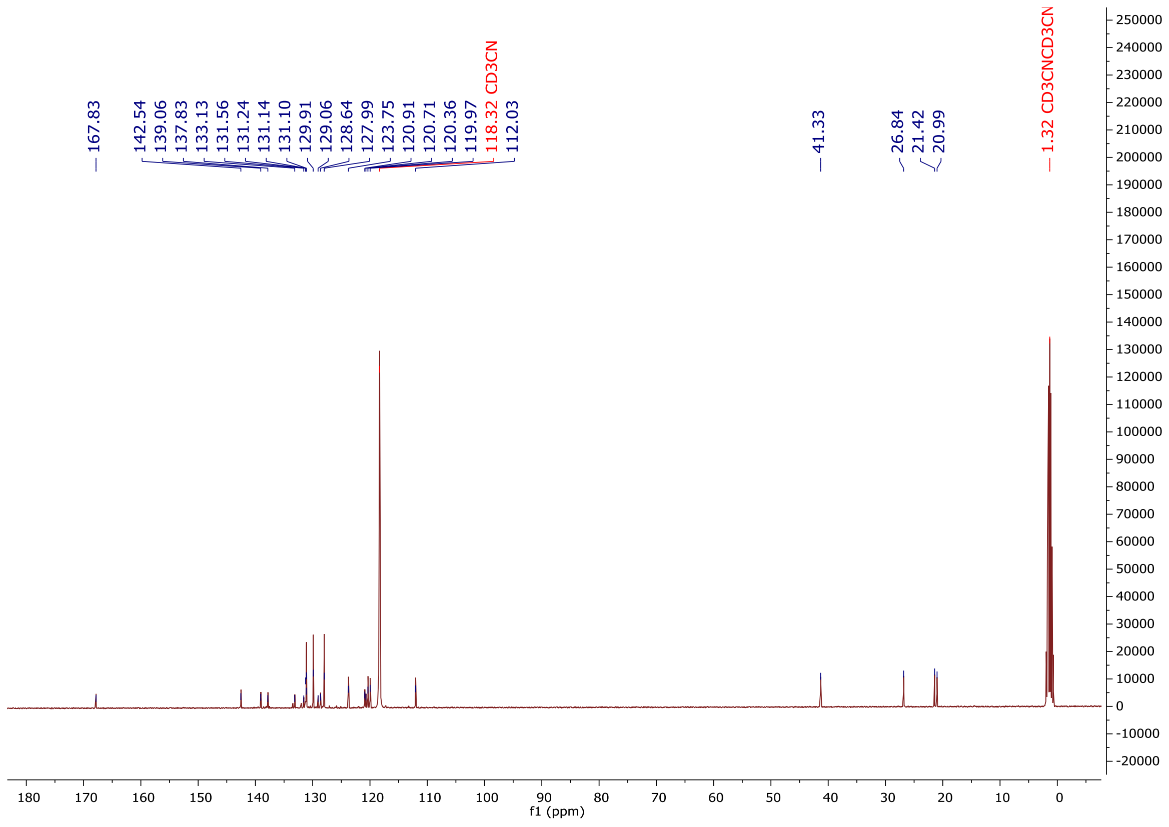

Figure S50. NMR of ^{13}C (101 MHz) in Acetonitrile-d_3 of compound **5j**

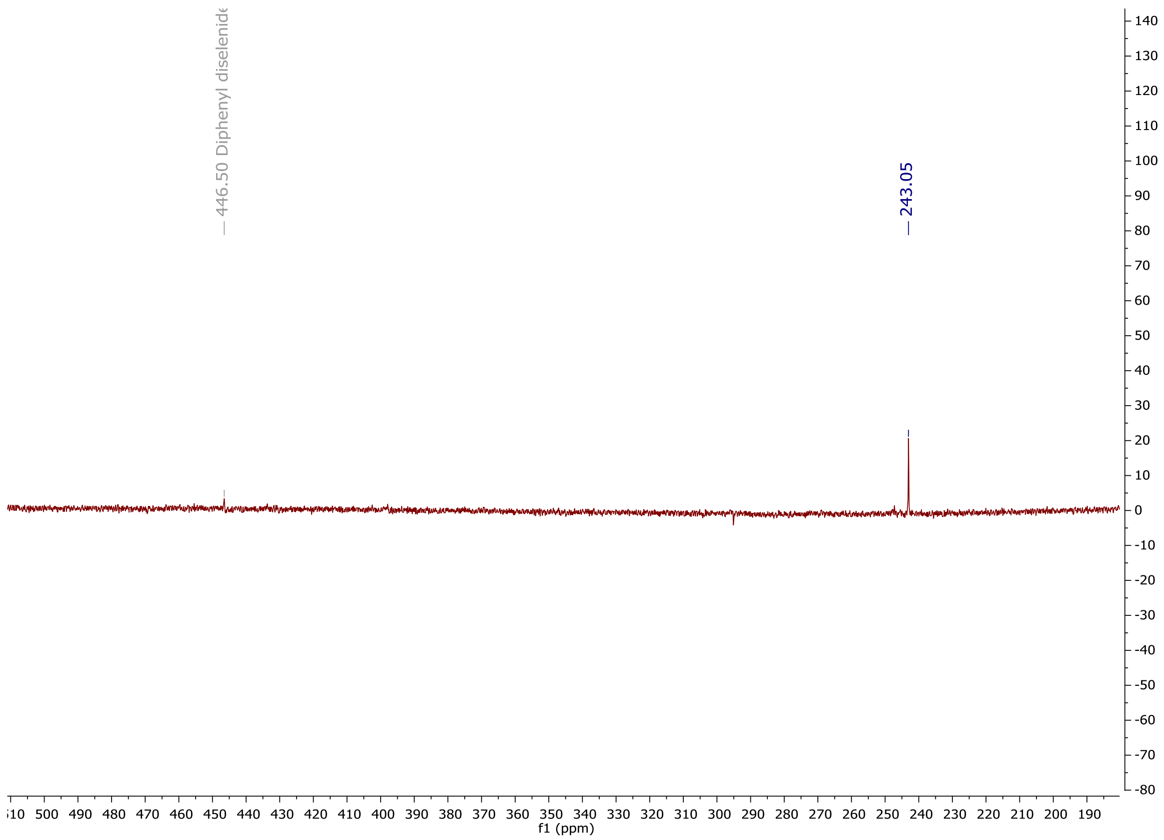

Figure S51. NMR of ^{77}Se (76 MHz) in Acetonitrile-d3 of compound **5j**

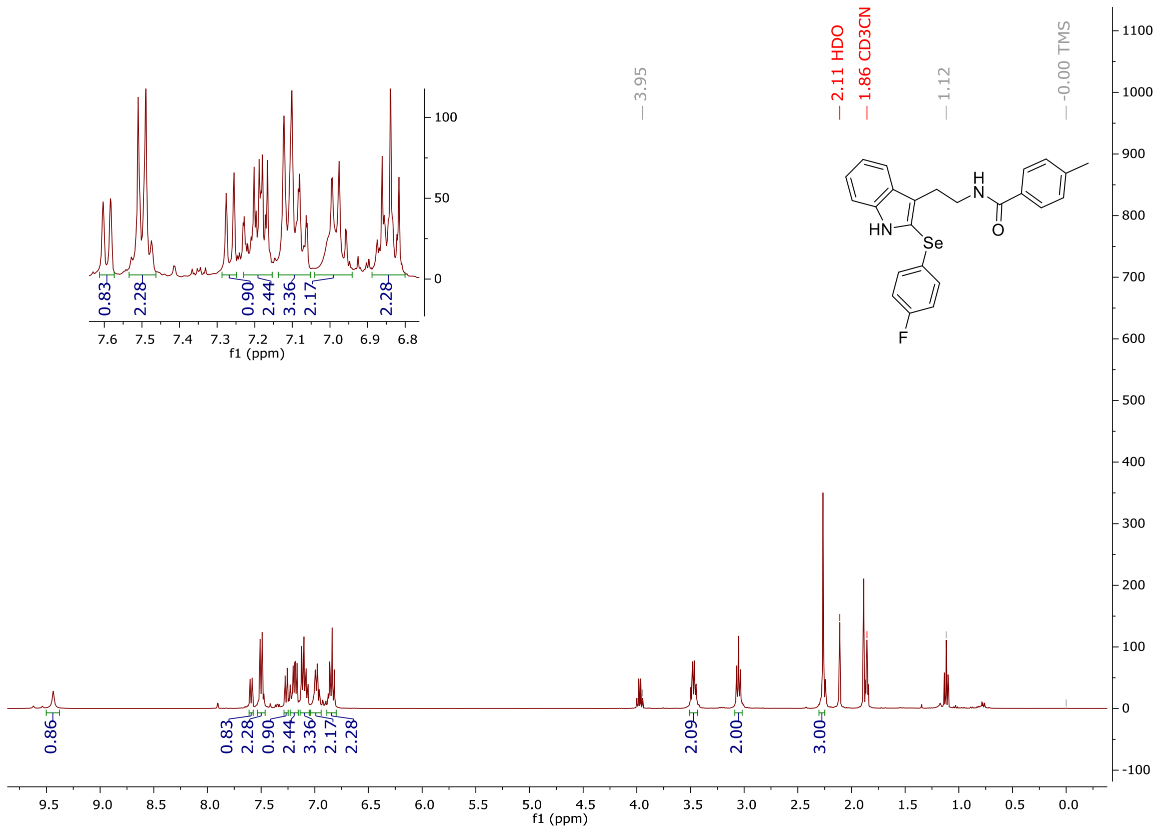

Figure S52. NMR of ^{1}H (400 MHz) in Acetonitrile-d3 of compound **5k**

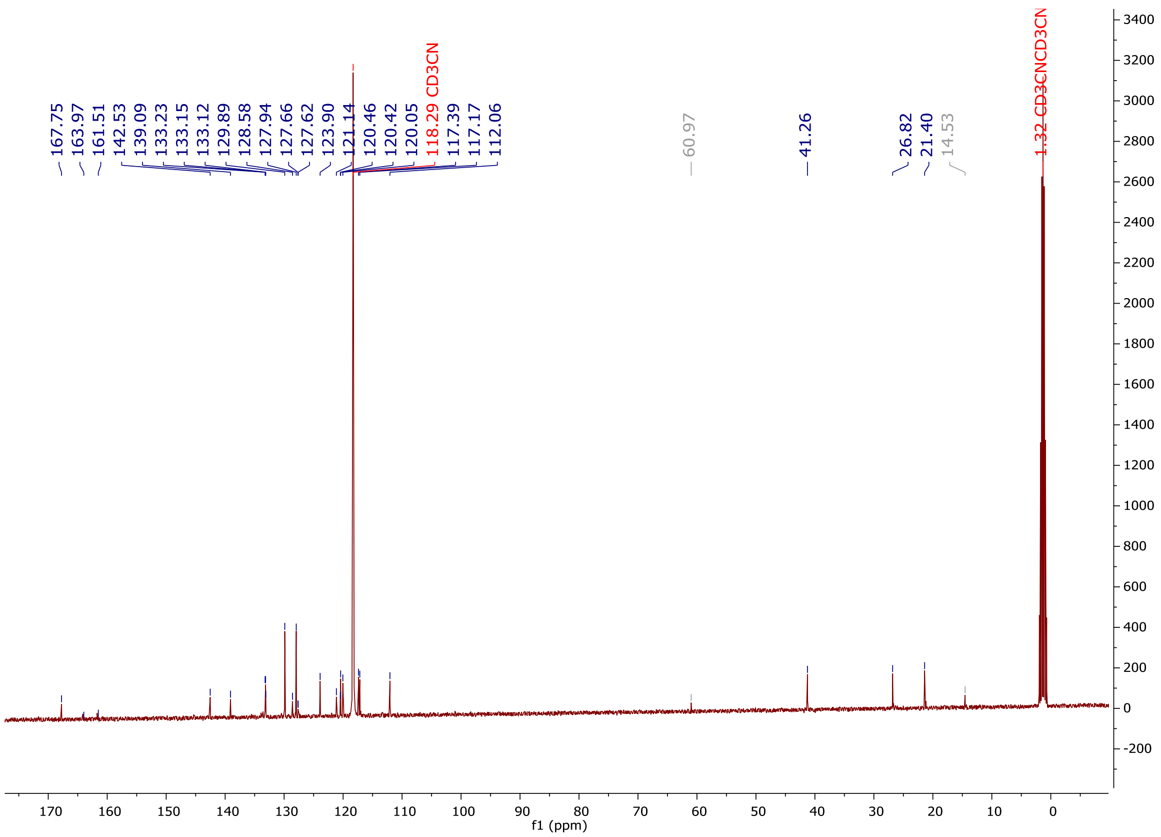

Figure S53. NMR of ^{13}C (101 MHz) in Acetonitrile-d$_3$ of compound **5k**

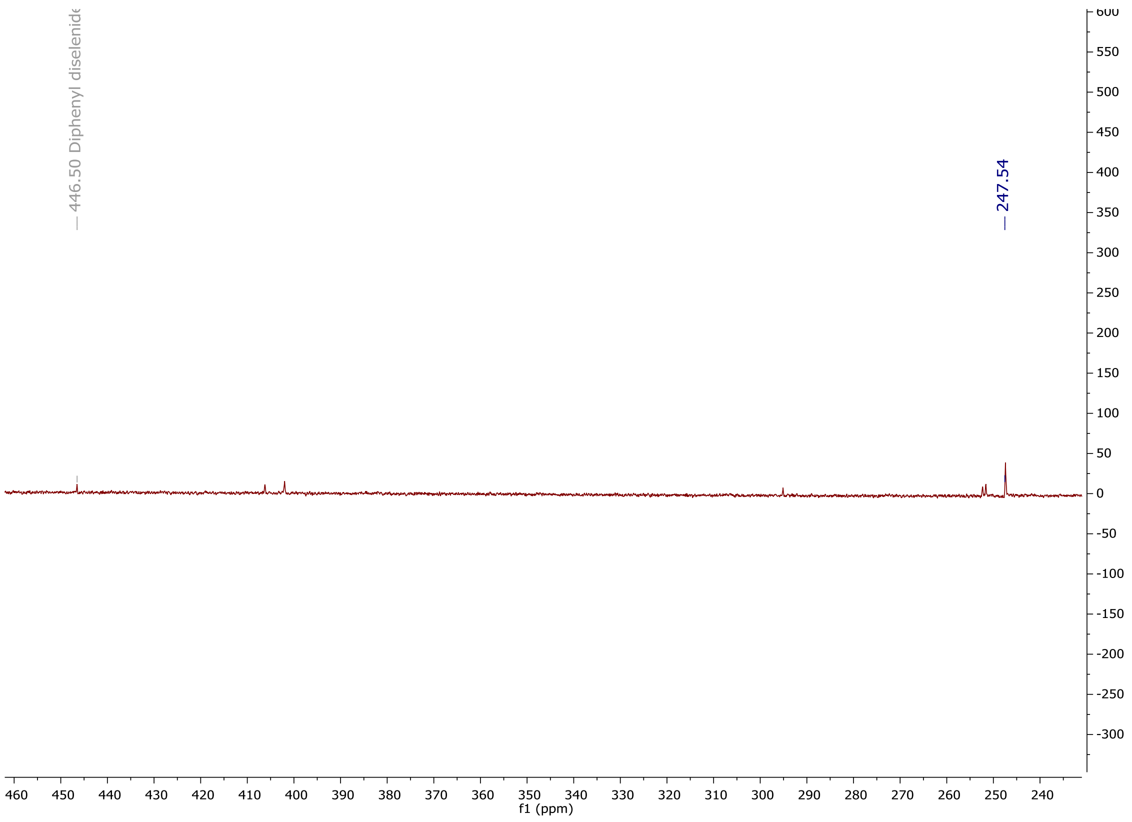

Figure S54. NMR of ^{77}Se (76 MHz) in Acetonitrile-d$_3$ of compound **5k**

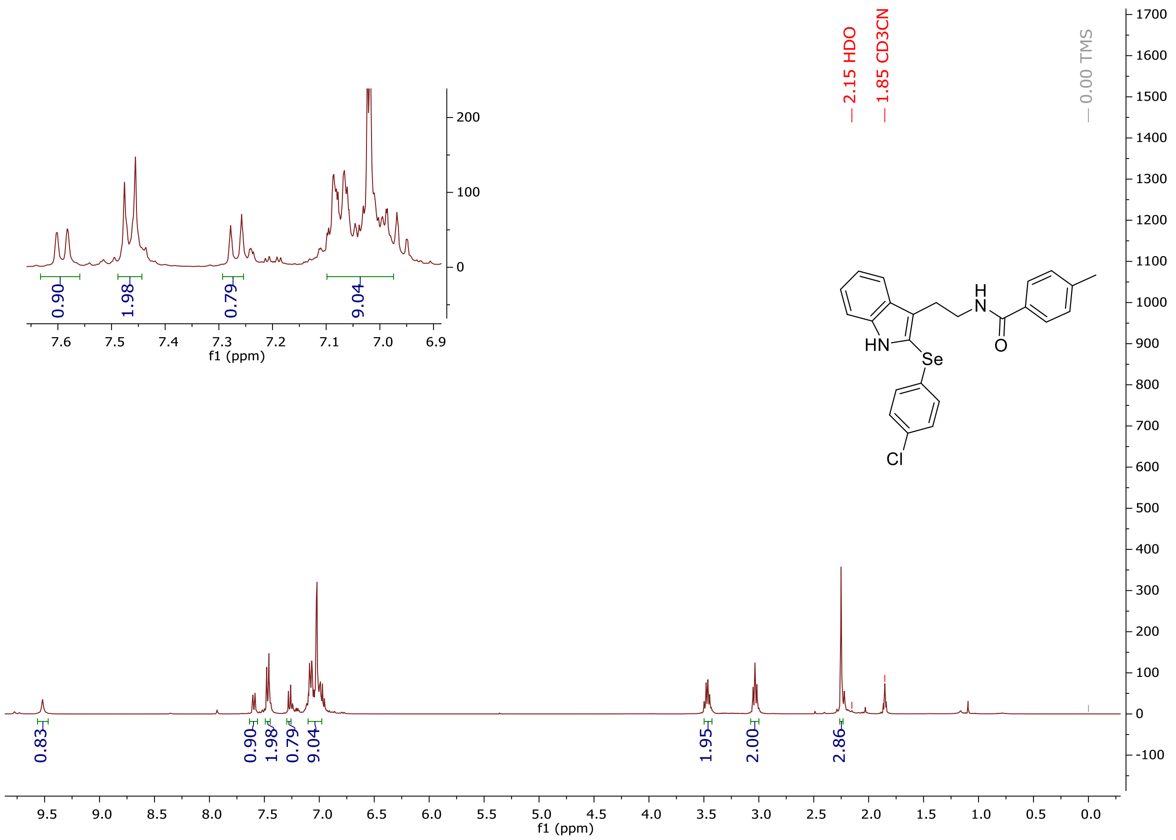

Figure S55. NMR of ^{1}H (400 MHz) in Acetonitrile-d_3 of compound **5l**

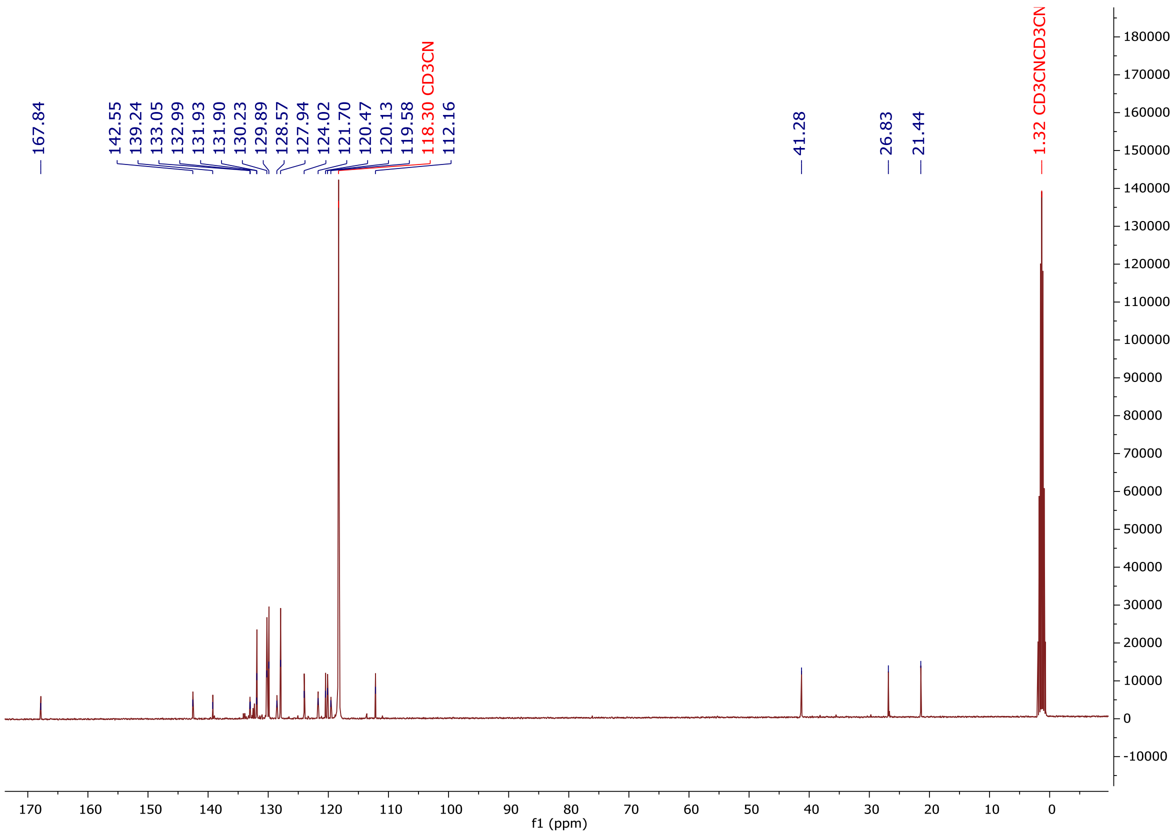

Figure S56. NMR of ^{13}C (101 MHz) in Acetonitrile-d_3 of compound **5l**

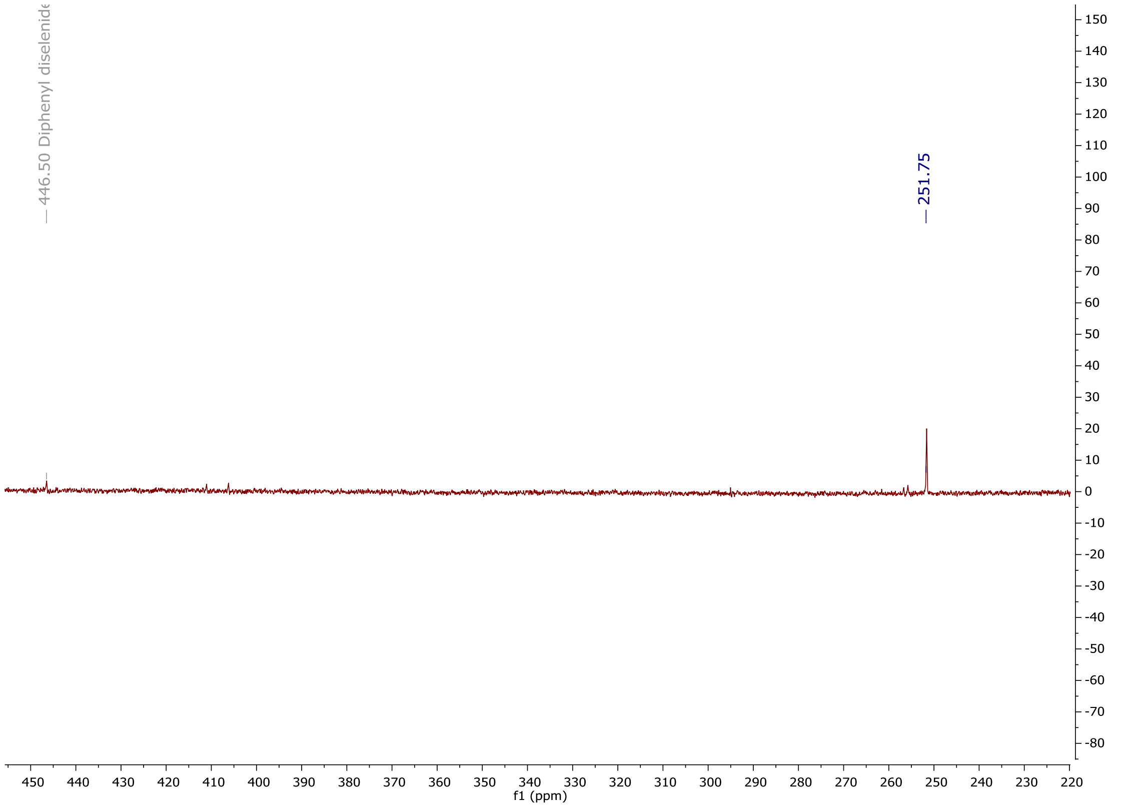

Figure S57. NMR of ^{77}Se (76 MHz) in Acetonitrile-d$_3$ of compound **5l**

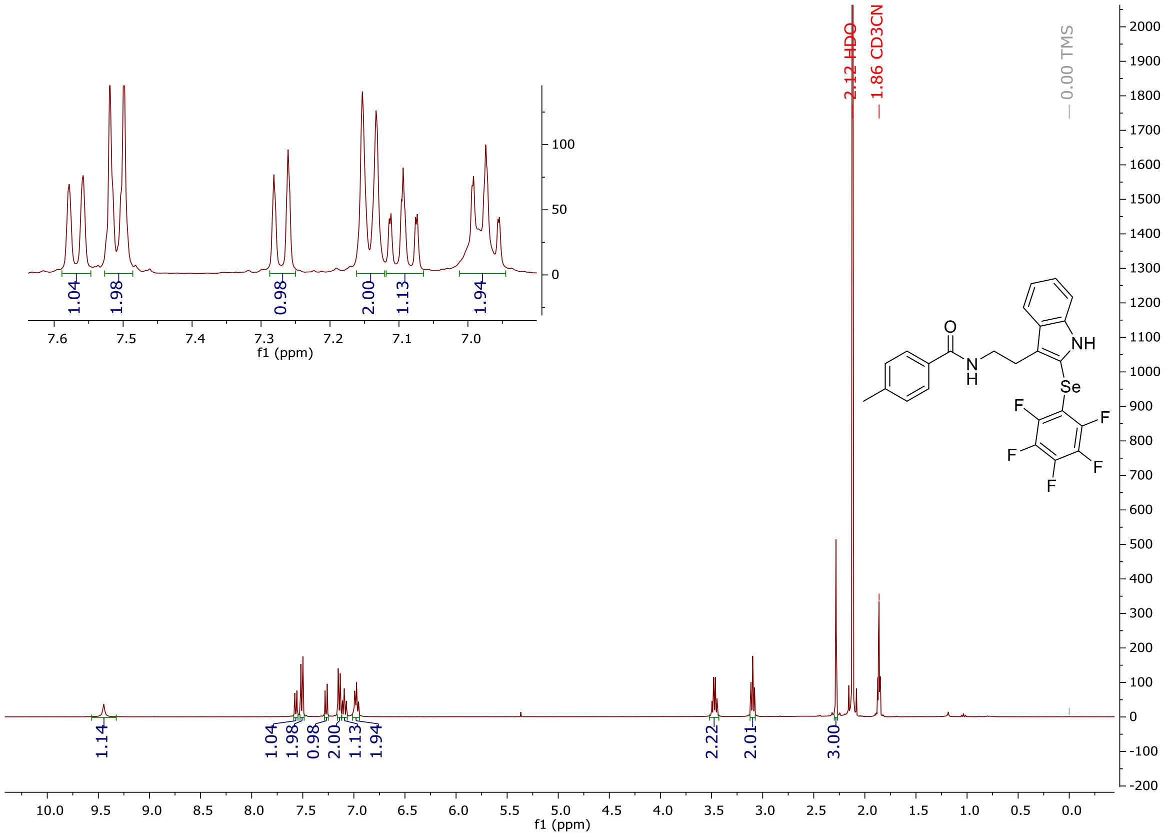

Figure S58. NMR of ^{1}H (400 MHz) in Acetonitrile-d$_3$ of compound **5m**

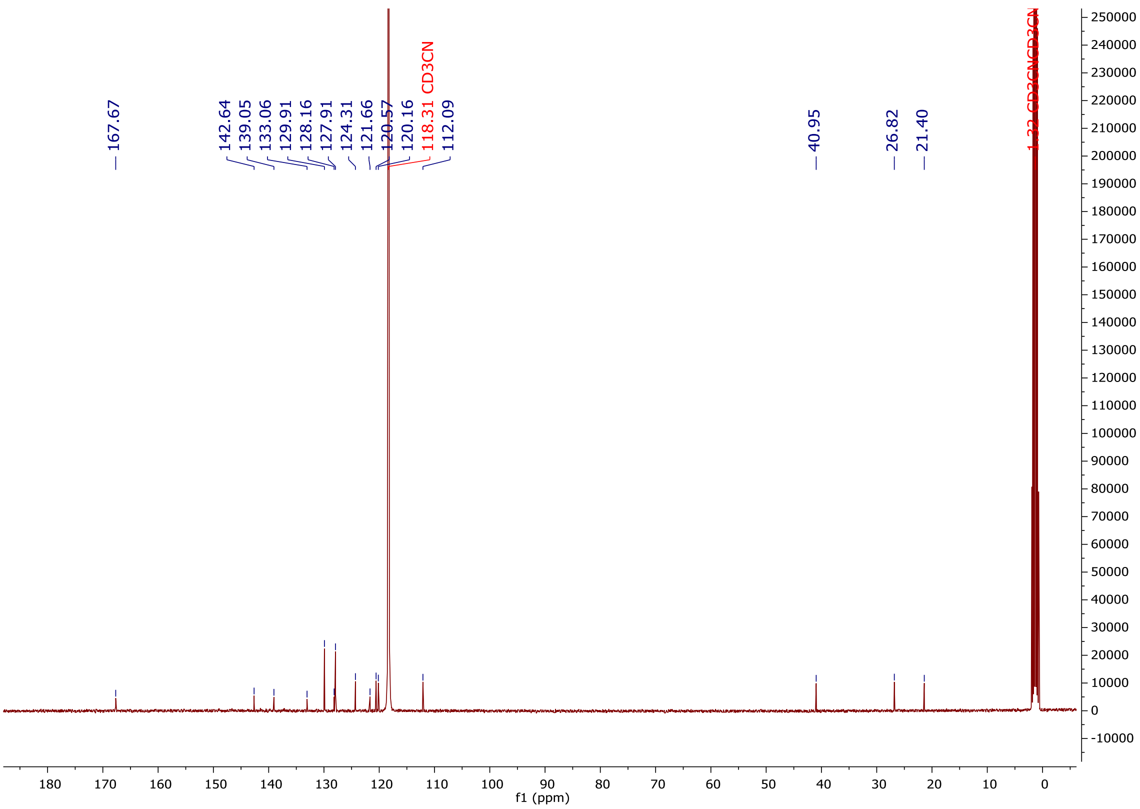

Figure S59. NMR of ^{13}C (101 MHz) in Acetonitrile-d_3 of compound **5m**

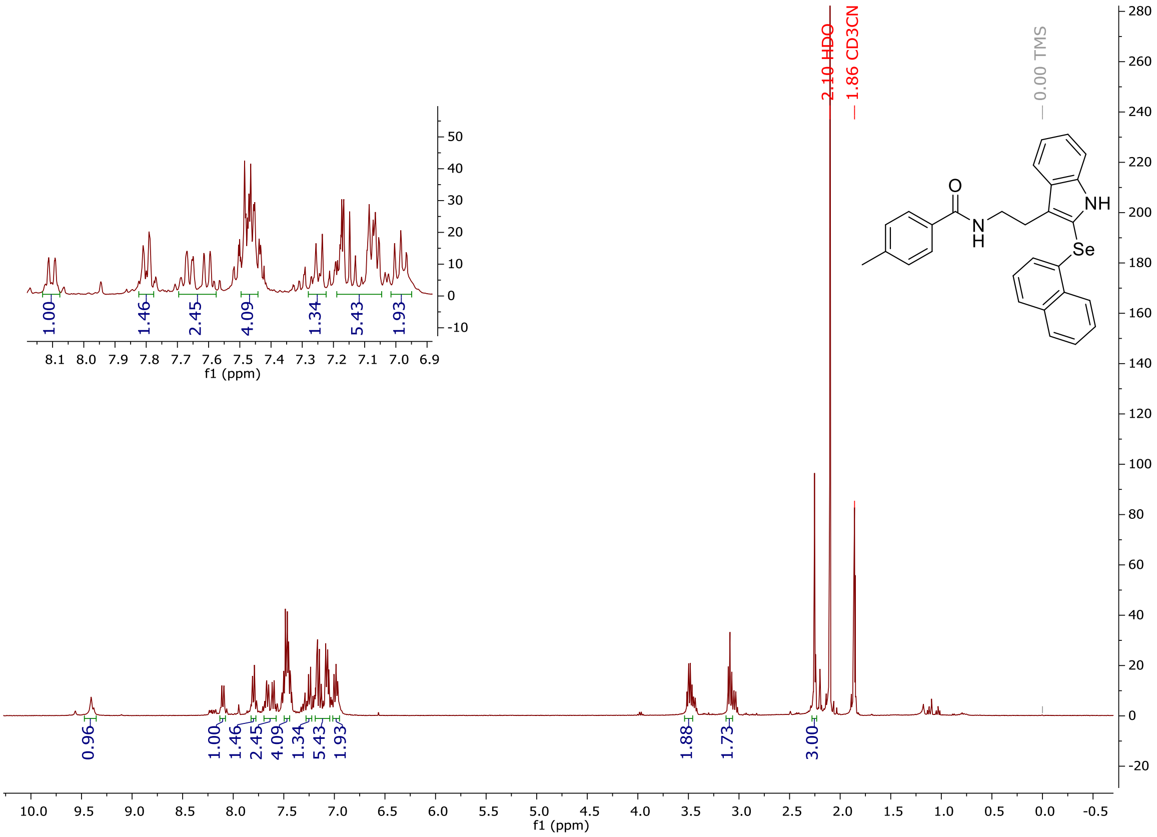

Figure S60. NMR of ^{1}H (400 MHz) in Acetonitrile-d_3 of compound **5n**

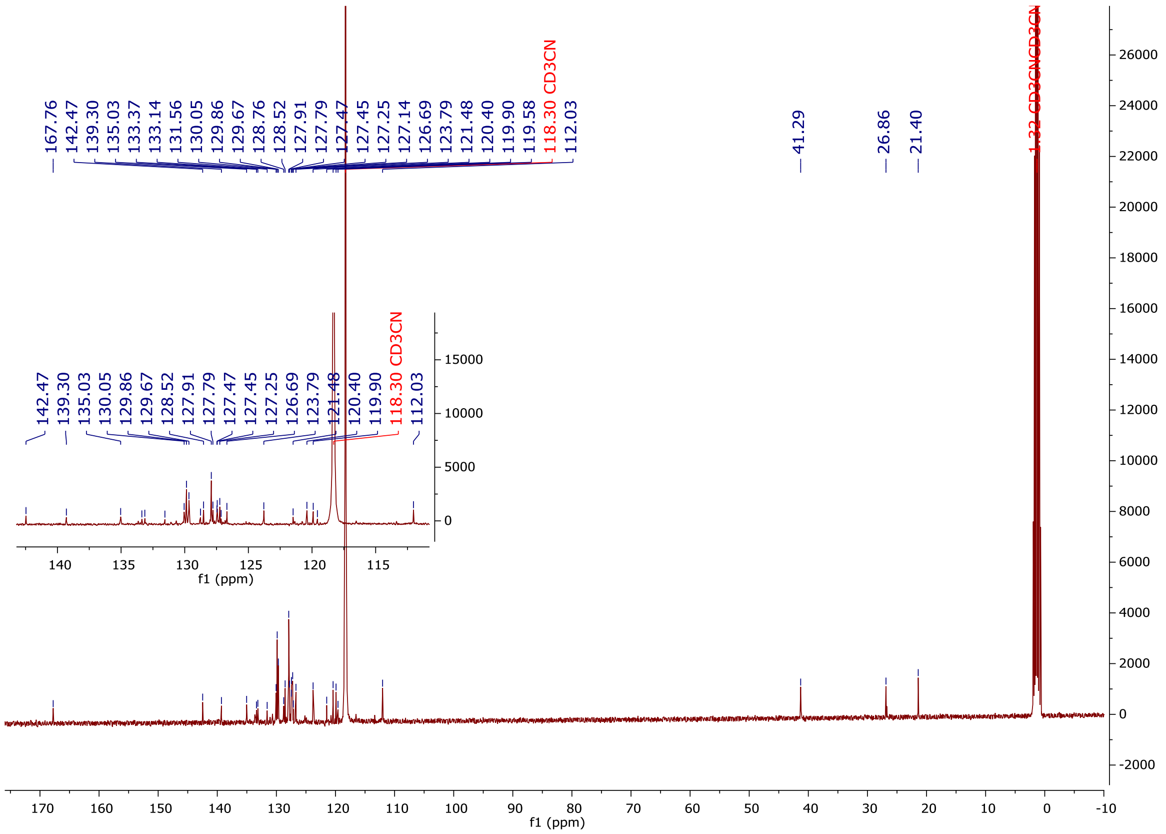

Figure S61. NMR of ^{13}C (101 MHz) in Acetonitrile-d_3 of compound **5n**

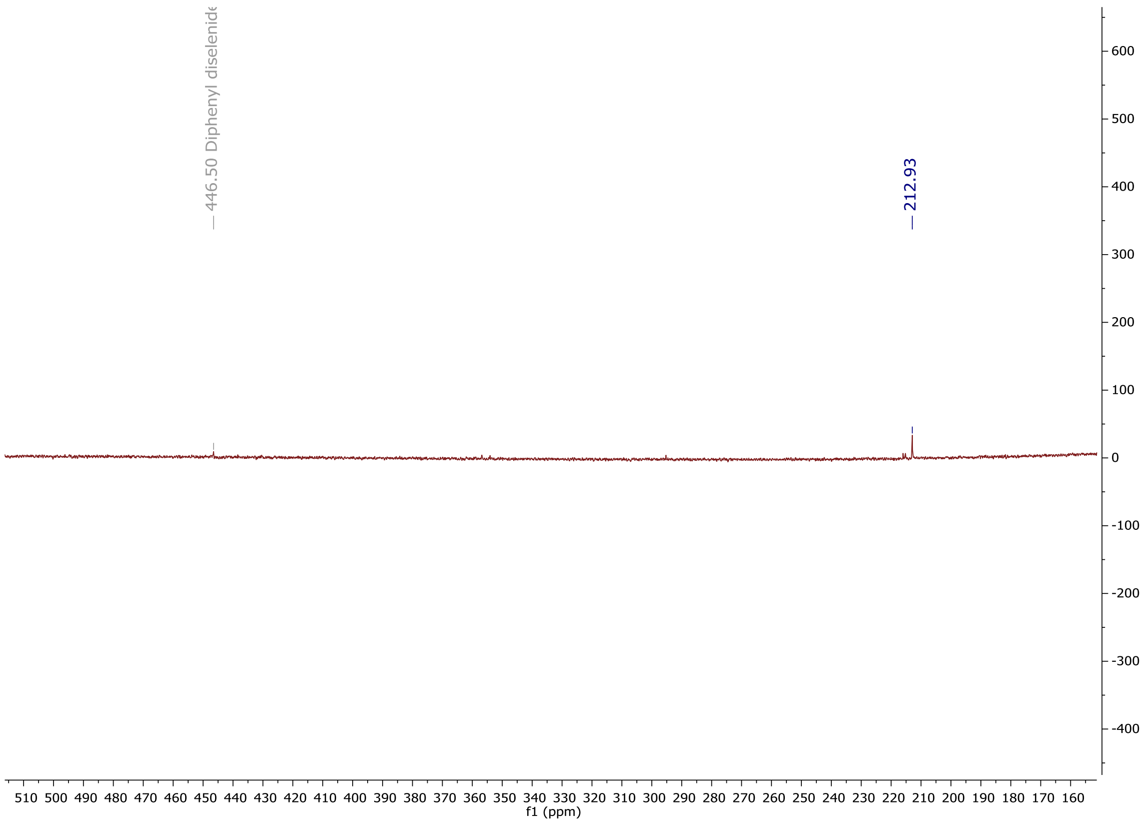

Figure S62. NMR of ^{77}Se (76 MHz) in Acetonitrile-d_3 of compound **5n**

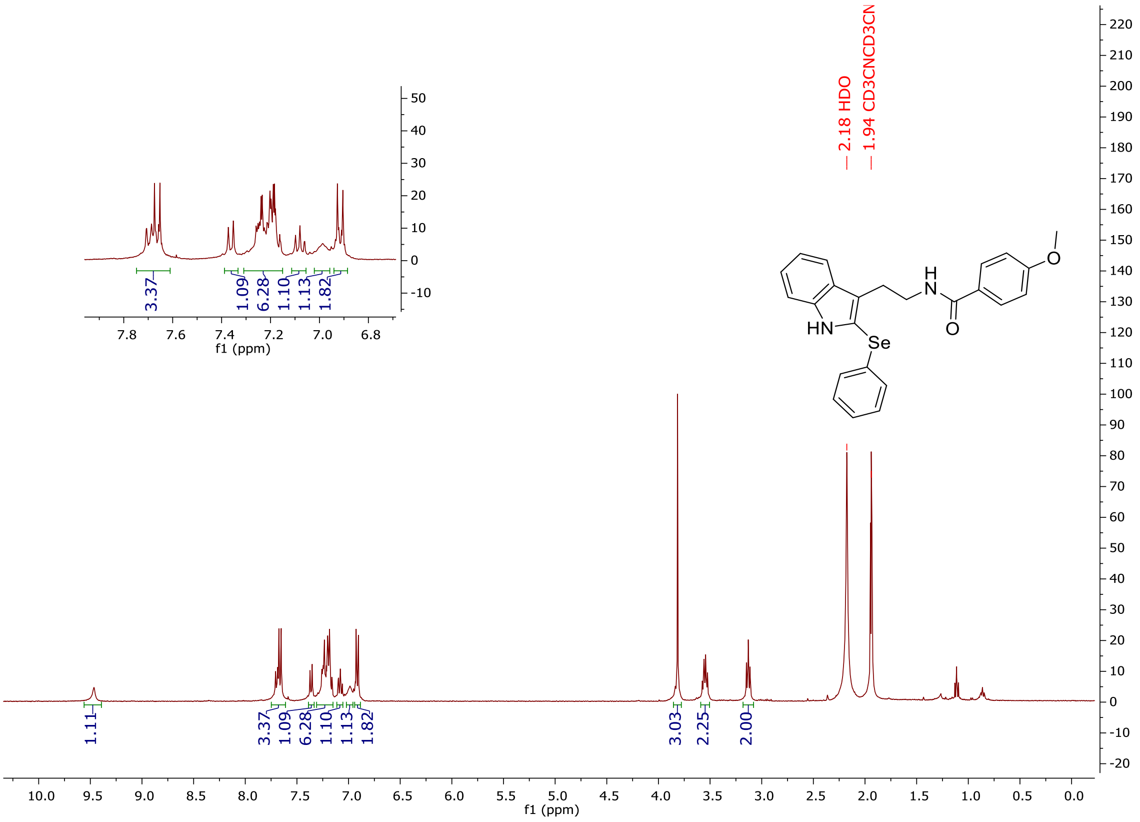

Figure S63. NMR of ^{1}H (400 MHz) in Acetonitrile-d3 of compound **5p**

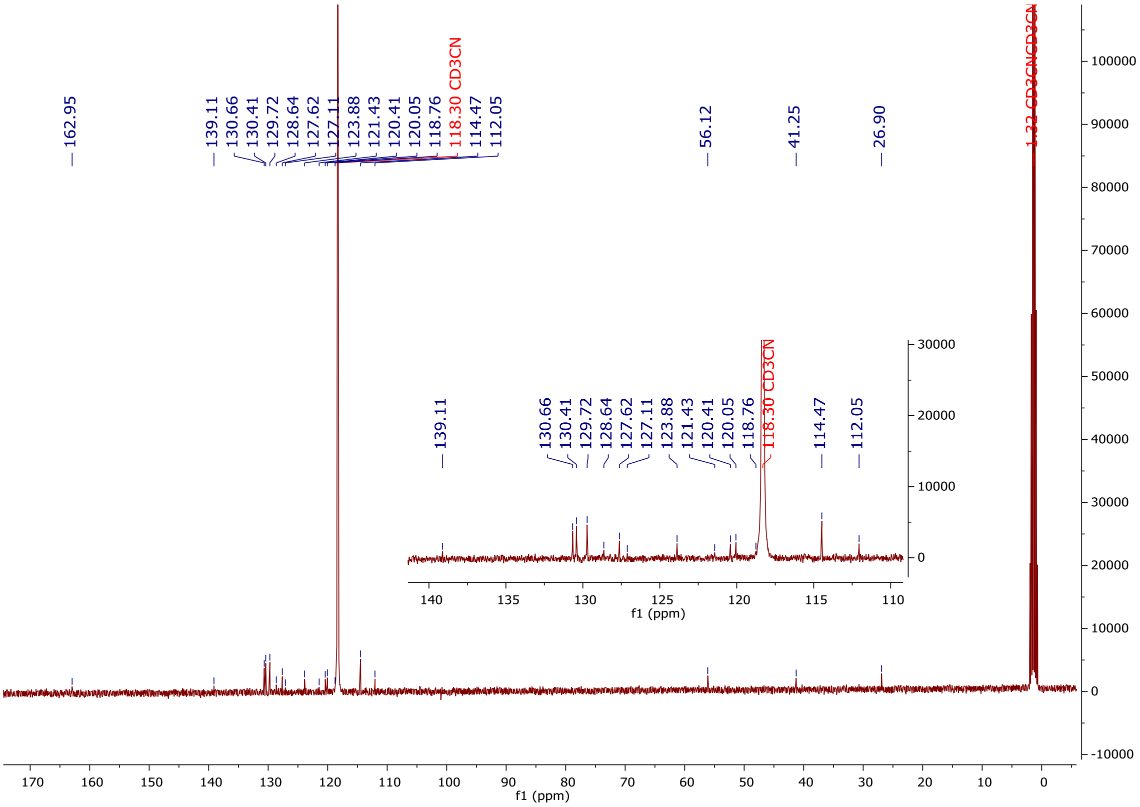

Figure S64. NMR of ^{13}C (101 MHz) in Acetonitrile-d3 of compound **5p**

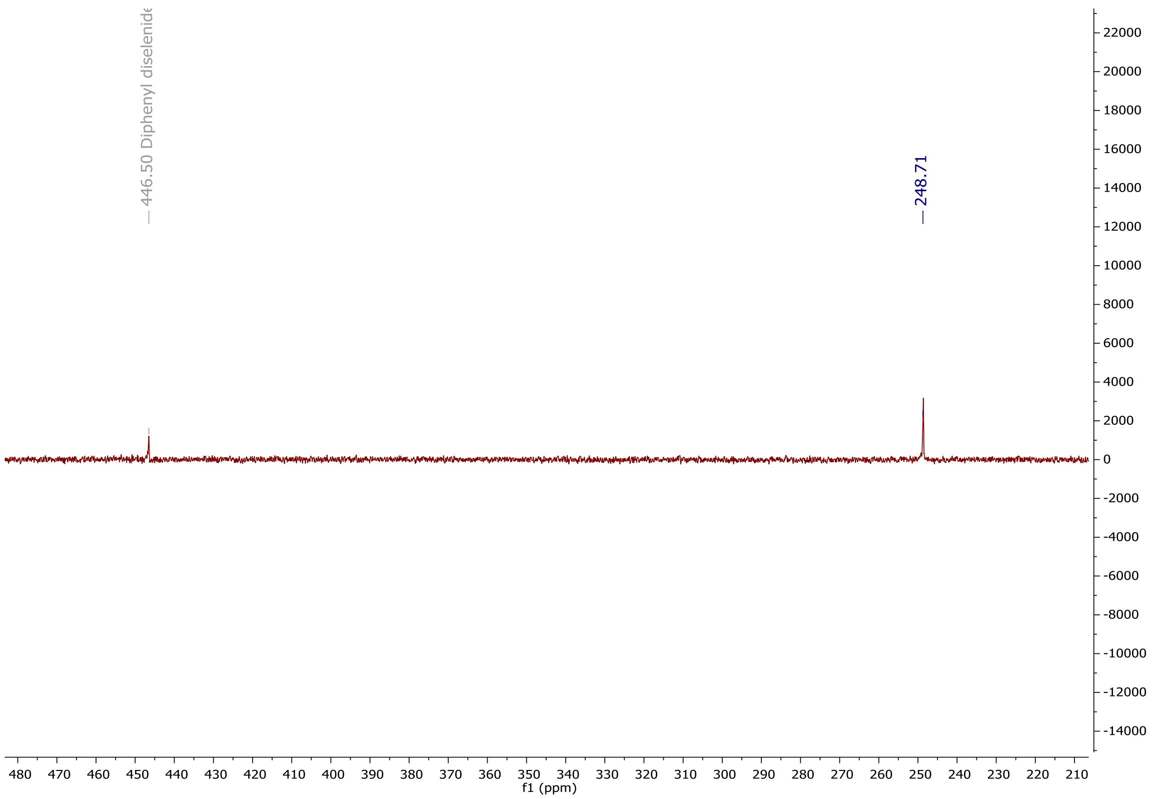

Figure S65. NMR of ^{77}Se (76 MHz) in Acetonitrile-d_3 of compound **5p**

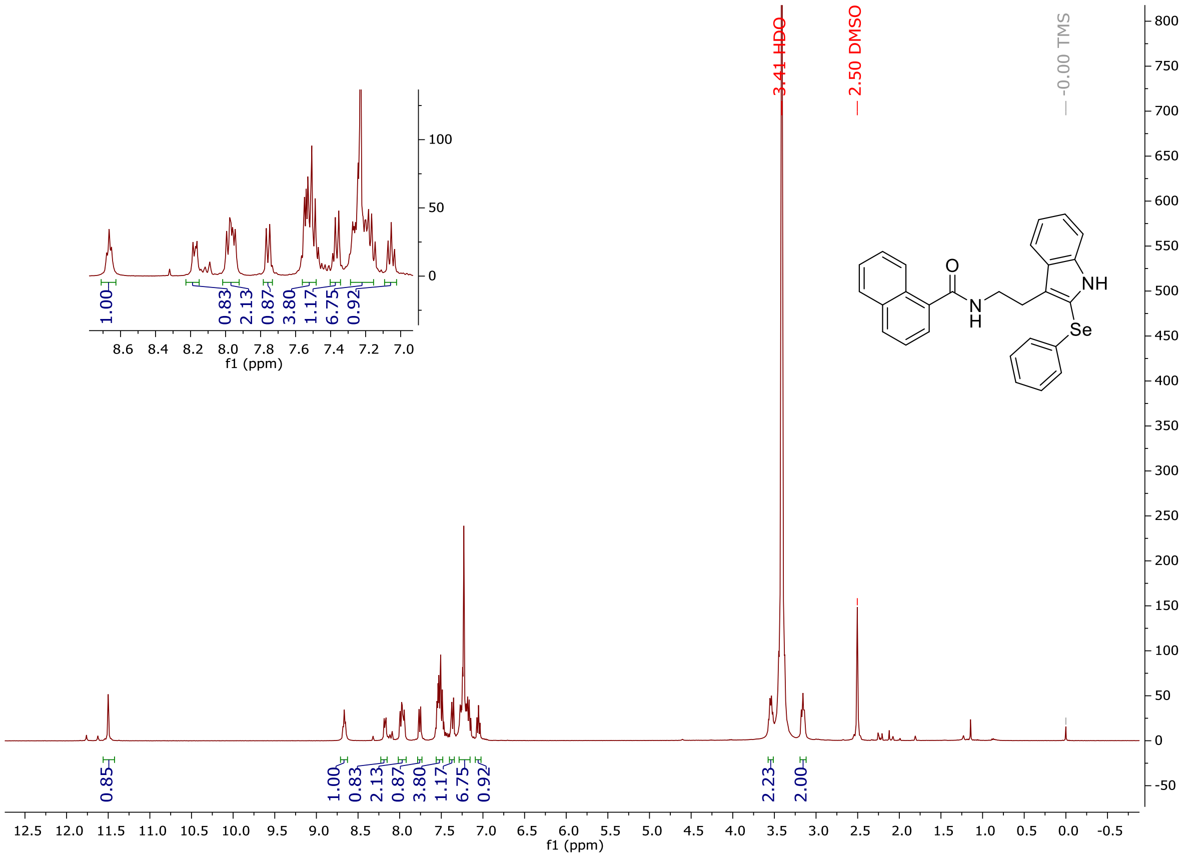

Figure S66. NMR of ^{1}H (400 MHz) in DMSO-d_6 of compound **5q**

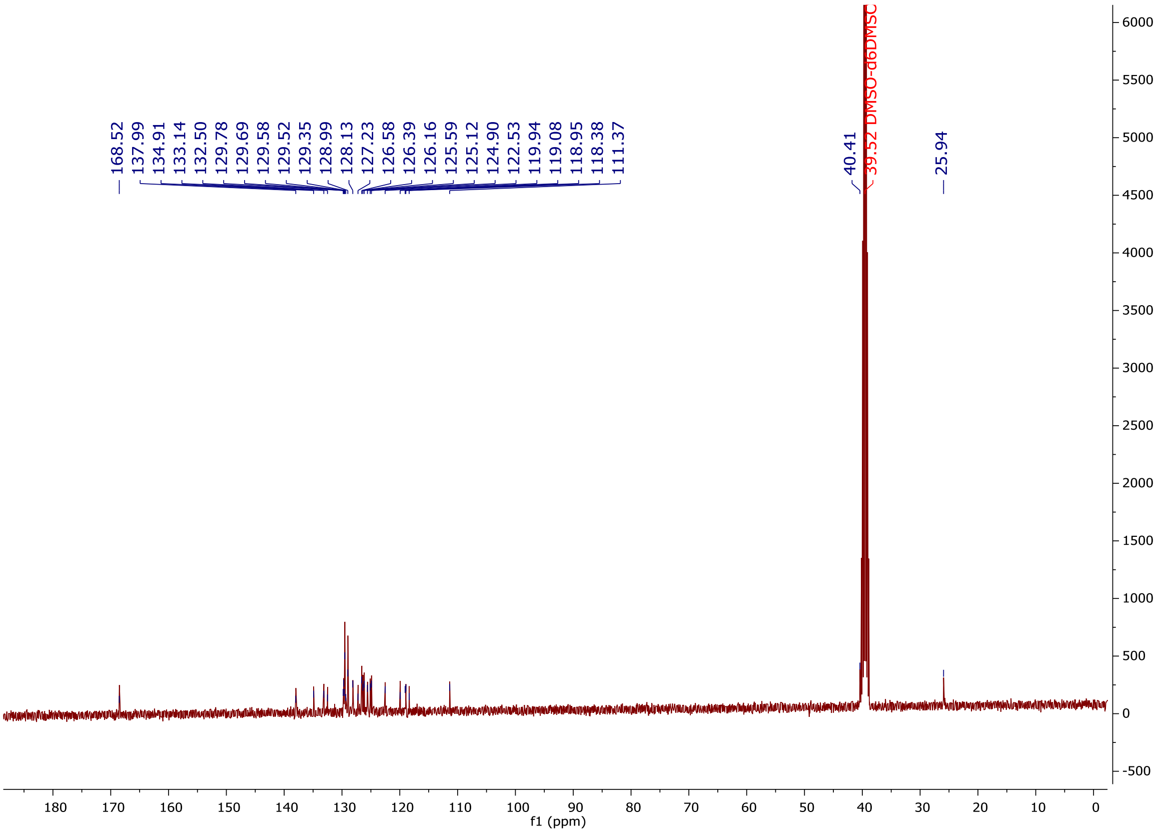

Figure S67. NMR of ^{13}C (101 MHz) in DMSO-d_6 of compound **5q**

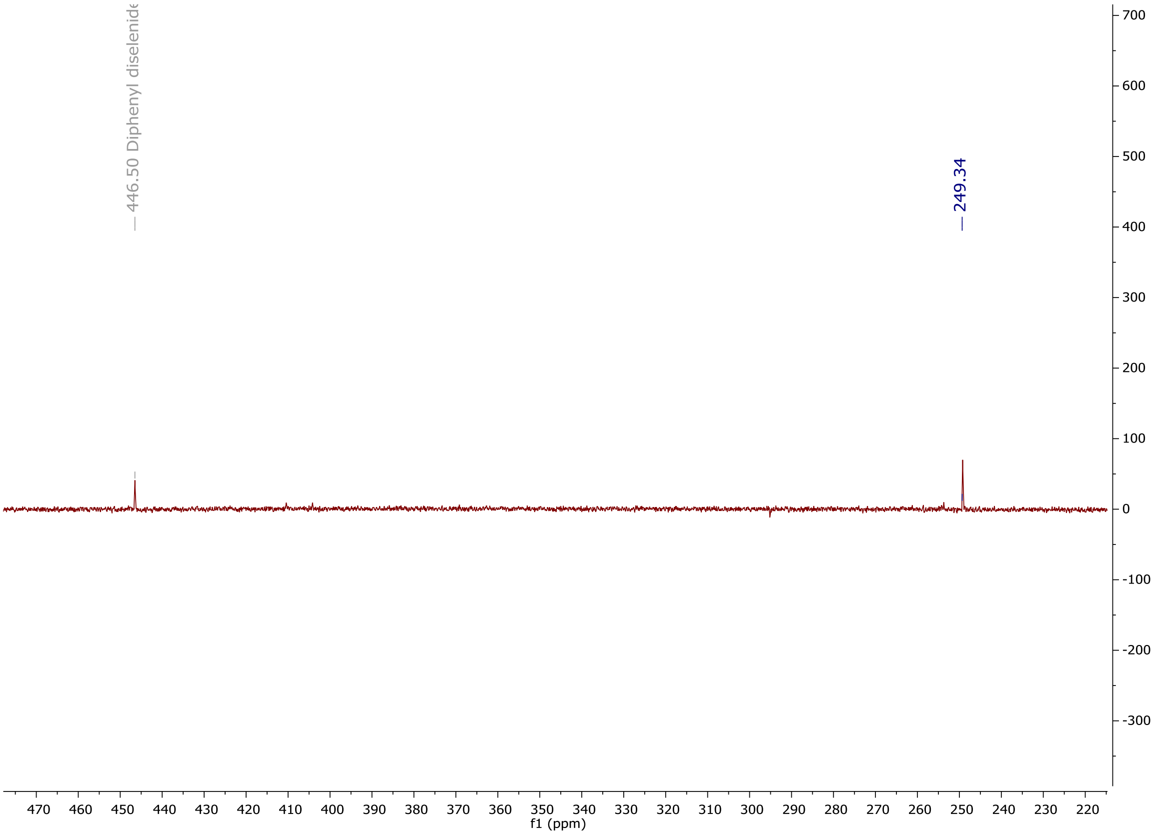

Figure S68. NMR of ^{77}Se (76 MHz) in DMSO-d_6 of compound **5q**

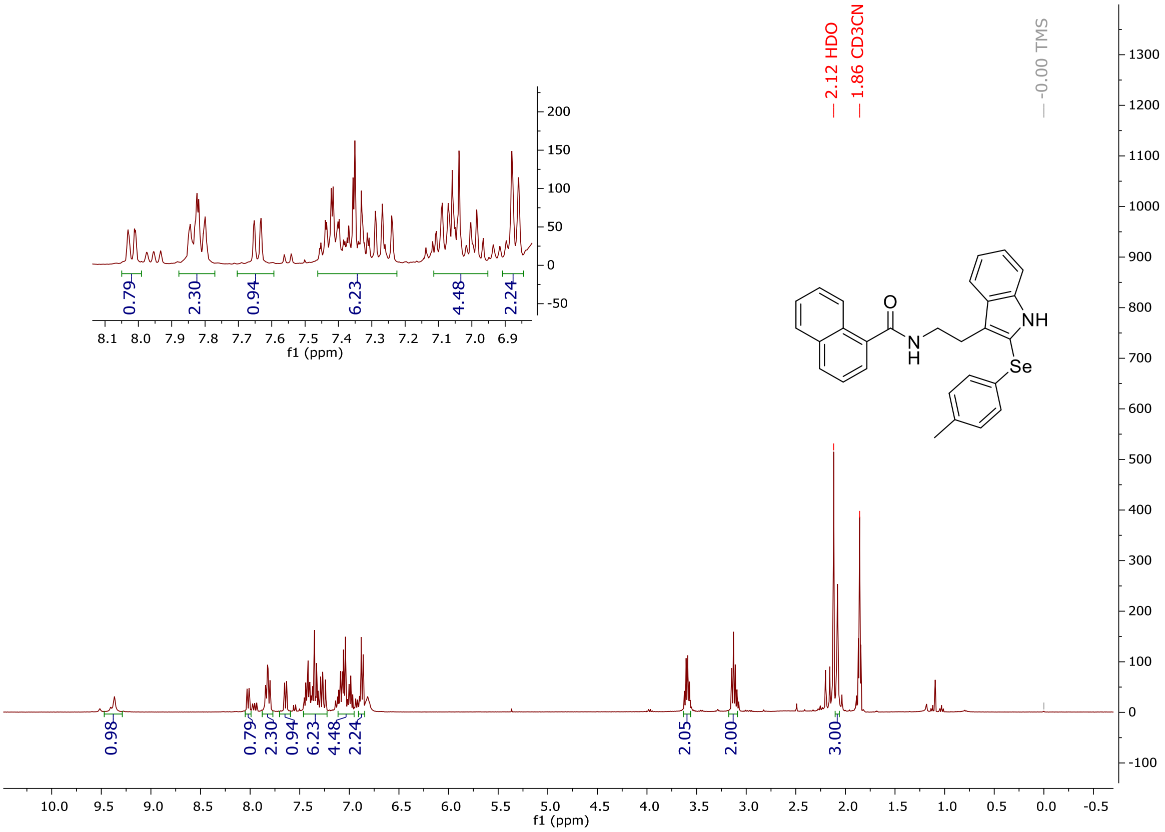

Figure S69. NMR of ^{1}H (400 MHz) in Acetonitrile-d$_3$ of compound **5r**

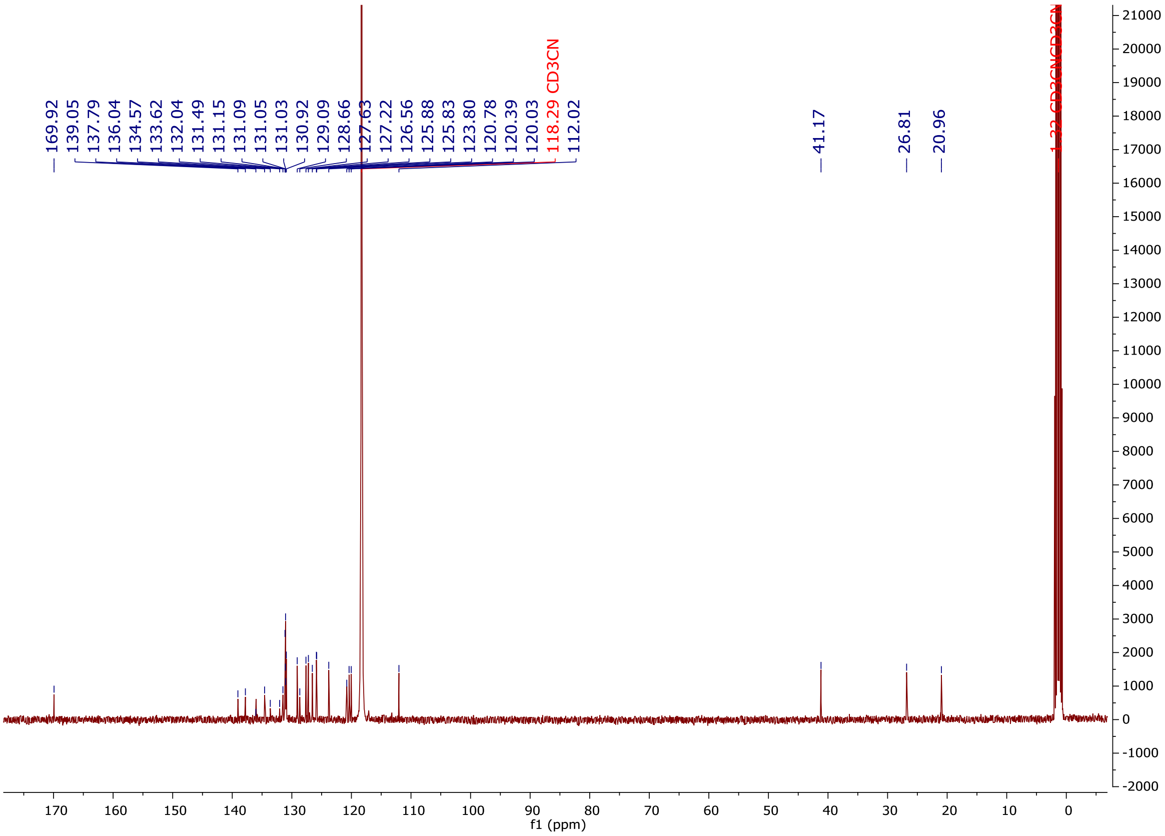

Figure S70. NMR of ^{13}C (101 MHz) in Acetonitrile-d$_3$ of compound **5r**

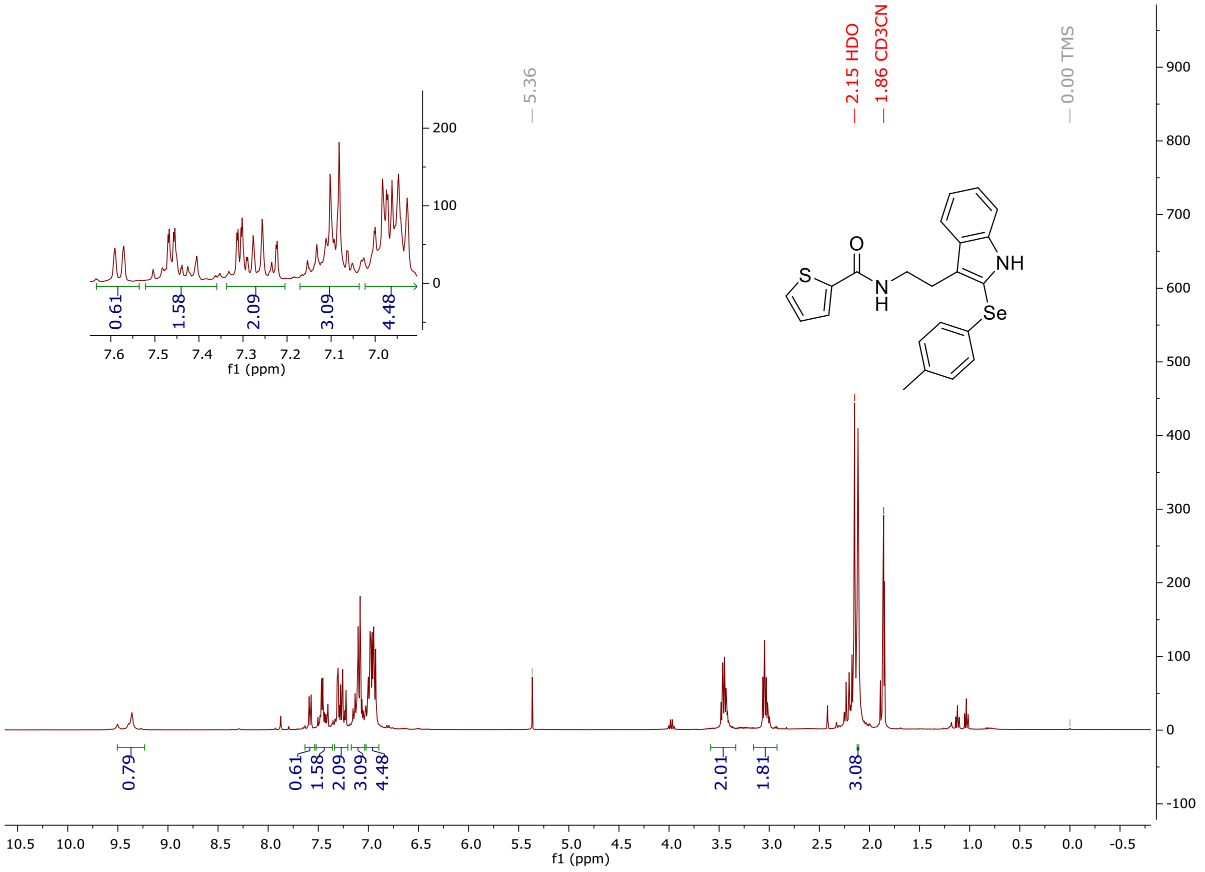

Figure S71. NMR of ^{1}H (400 MHz) in Acetonitrile-d_3 of compound **5s**

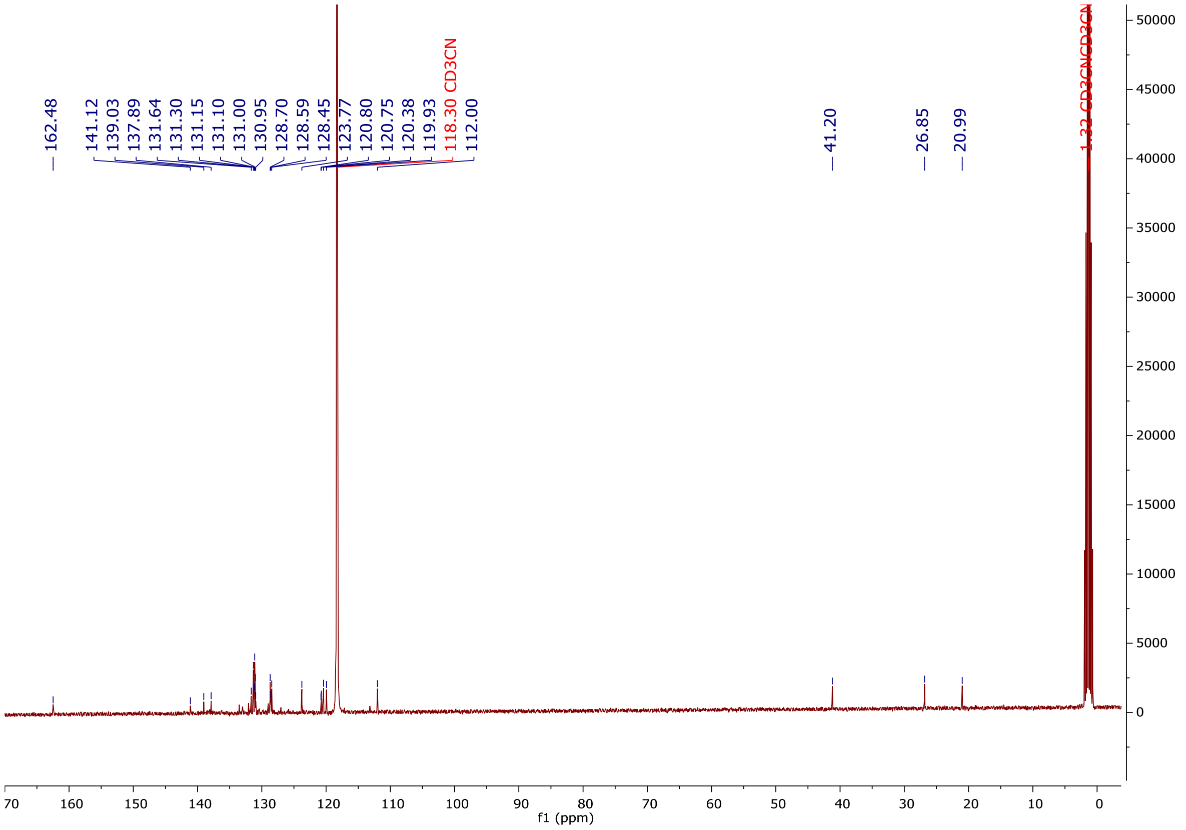

Figure S72. NMR of ^{13}C (101 MHz) in Acetonitrile-d_3 of compound **5s**

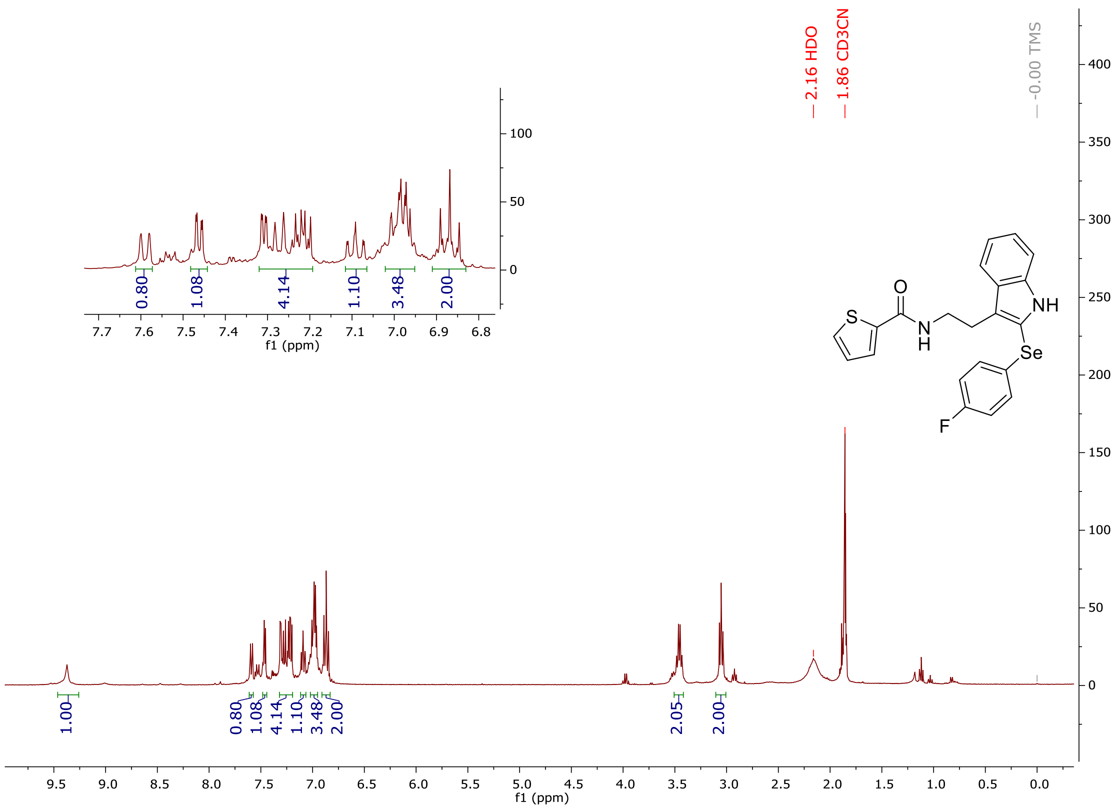

Figure S73. NMR of ^{1}H (400 MHz) in Acetonitrile-d_3 of compound **5t**

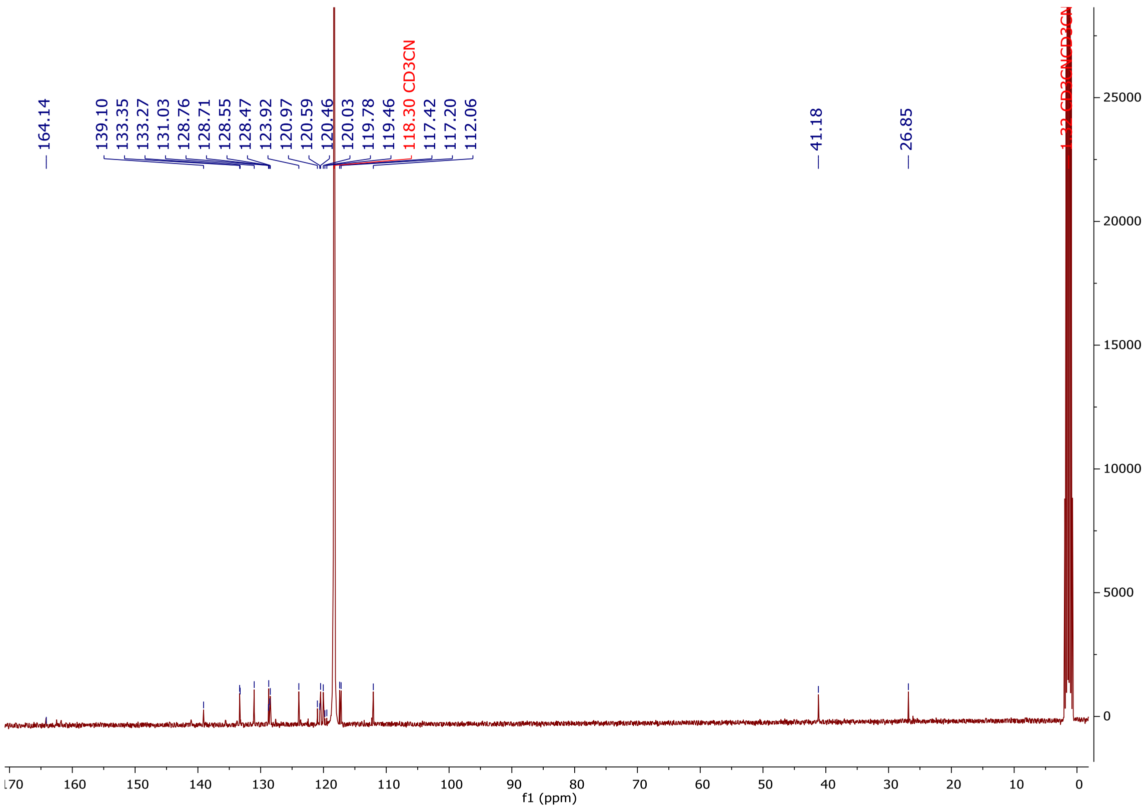

Figure S74. NMR of ^{13}C (101 MHz) in Acetonitrile-d_3 of compound **5t**

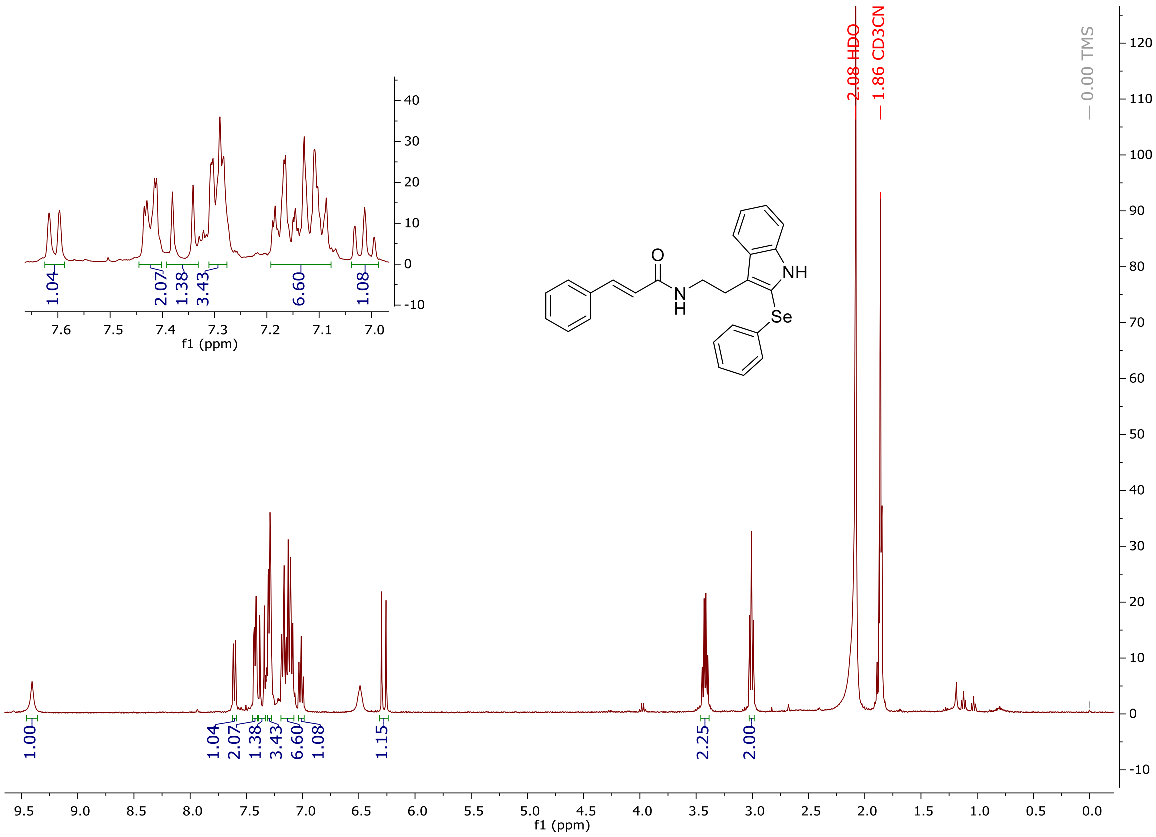

Figure S75. NMR of ^{1}H (400 MHz) in Acetonitrile-d_3 of compound **5u**

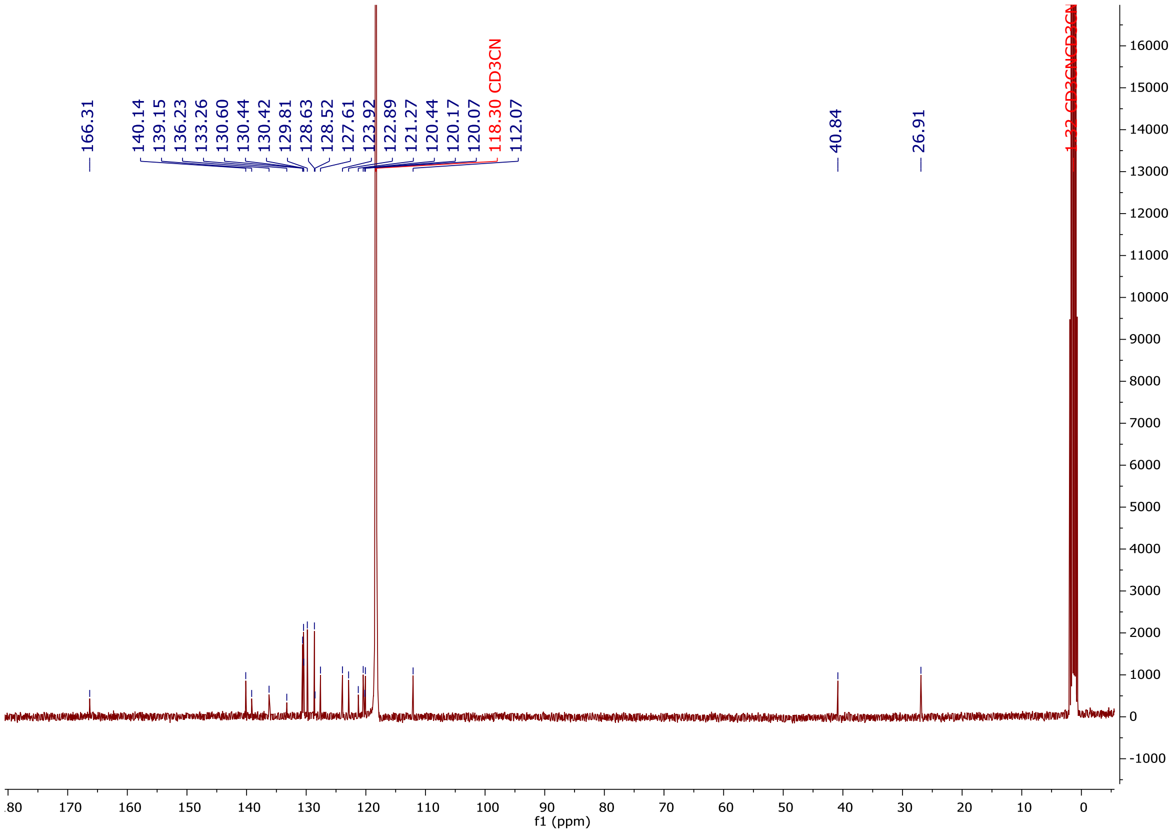

Figure S76. NMR of ^{13}C (101 MHz) in Acetonitrile-d_3 of compound **5u**

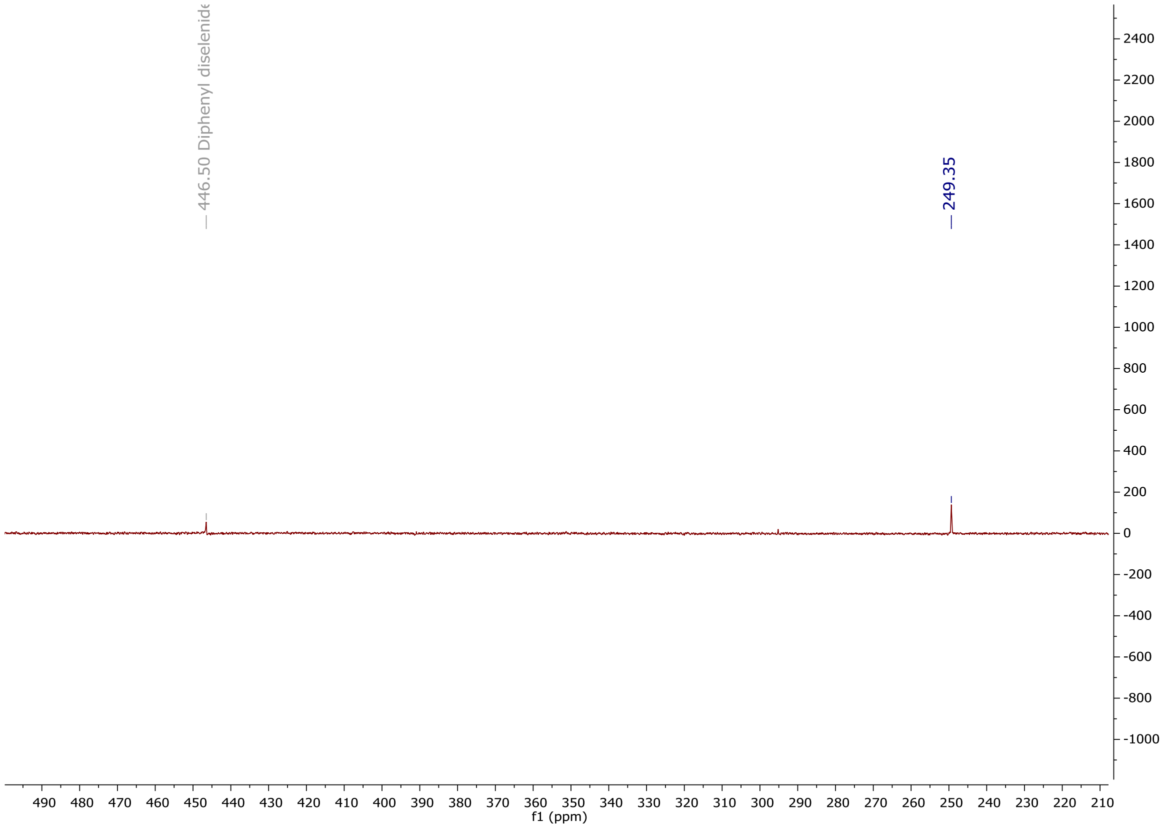

Figure S77. NMR of ^{77}Se (76 MHz) in Acetonitrile-d_3 of compound **5u**

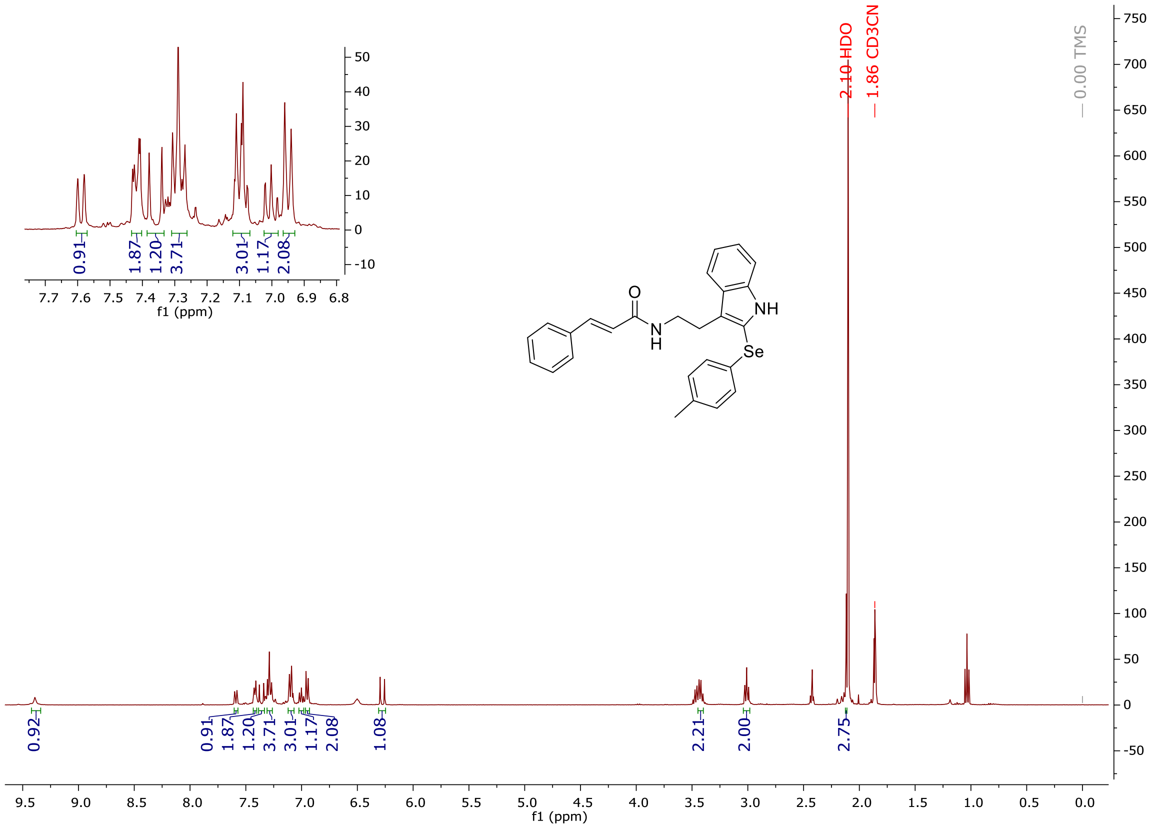

Figure S78. NMR of ^{1}H (400 MHz) in Acetonitrile-d_3 of compound **5v**

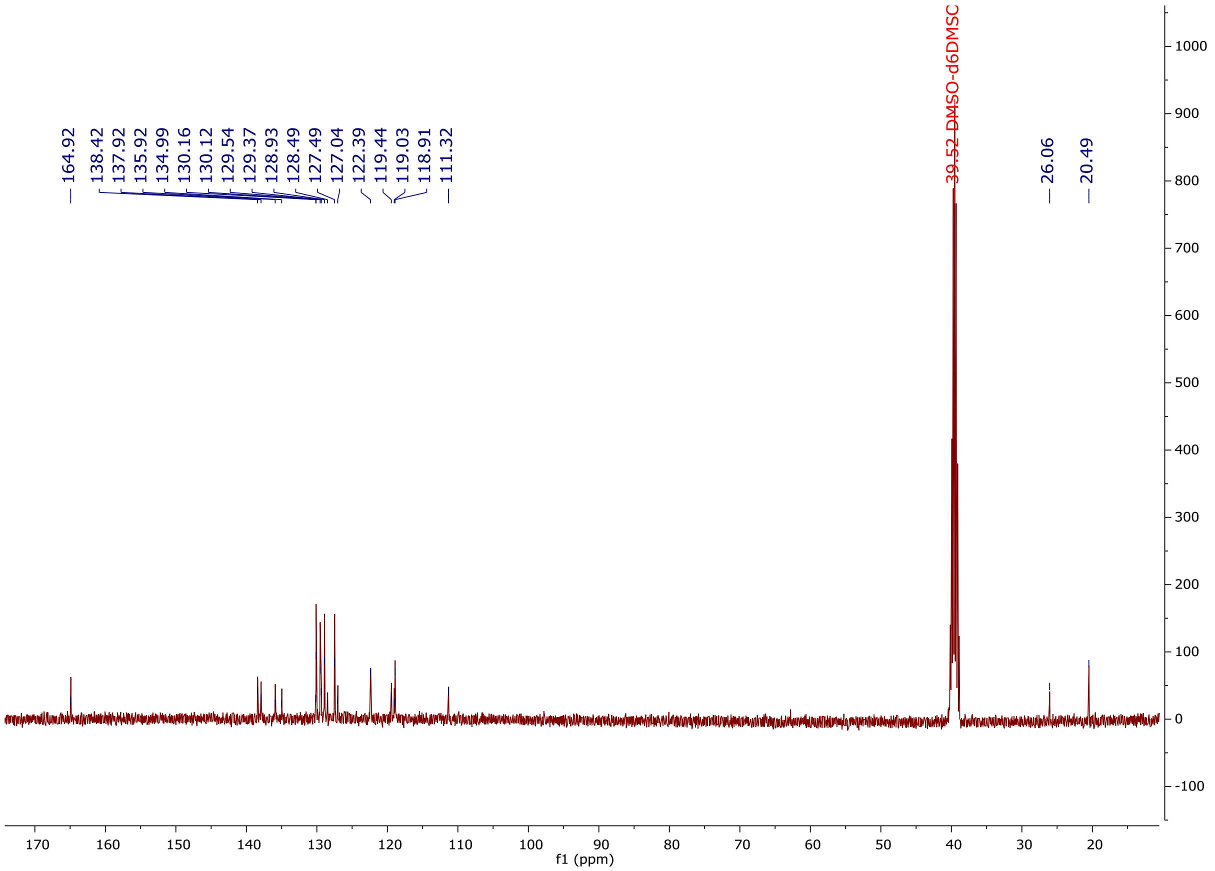

Figure S79. NMR of ^{13}C (101 MHz) in Acetonitrile-d$_3$ of compound **5v**

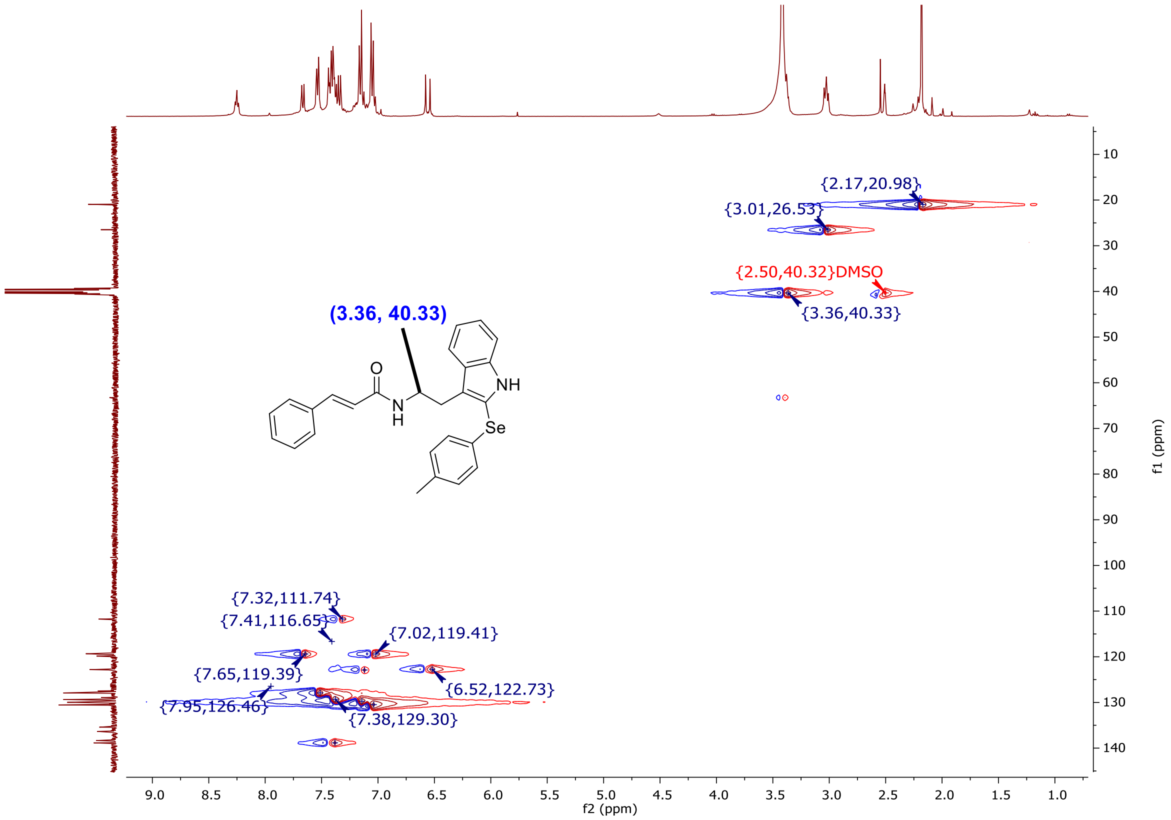

Figure S80. HSQC in Acetonitrile-d$_3$ of compound **5v**

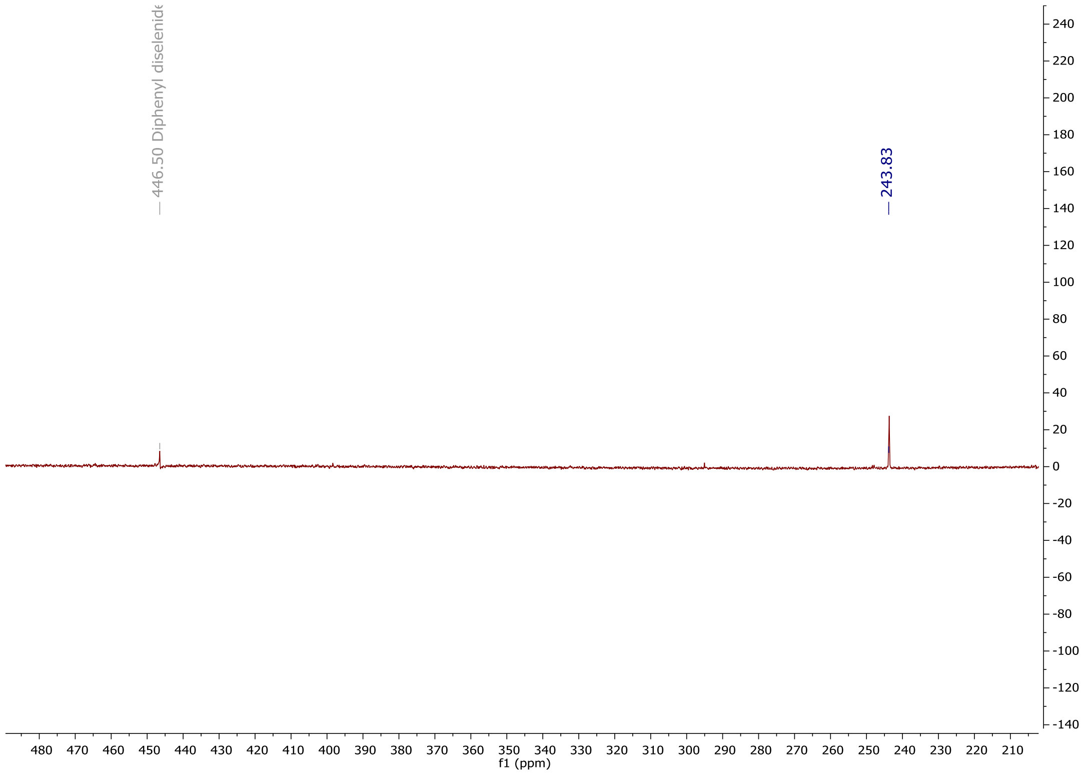

Figure S81. NMR of ^{77}Se (76 MHz) in Acetonitrile-d_3 of compound **5v**

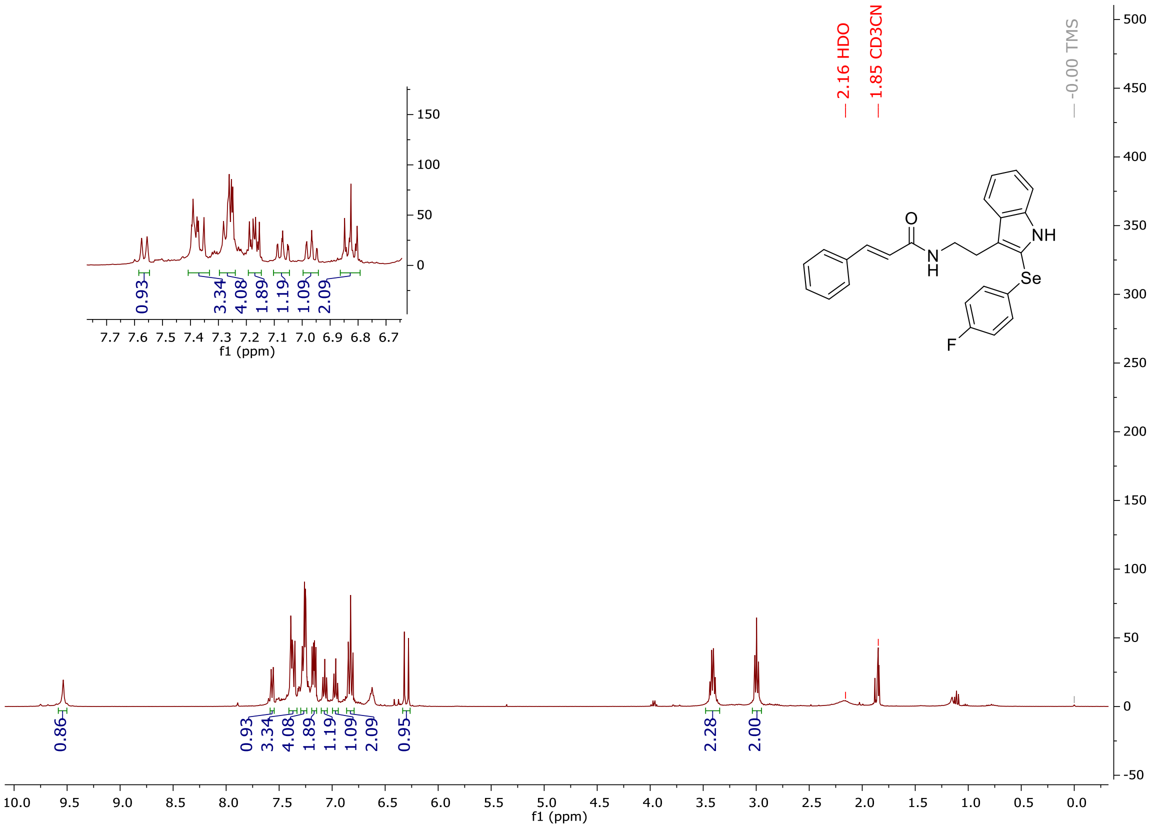

Figure S82. NMR of ^{1}H (400 MHz) in Acetonitrile-d_3 of compound **5w**

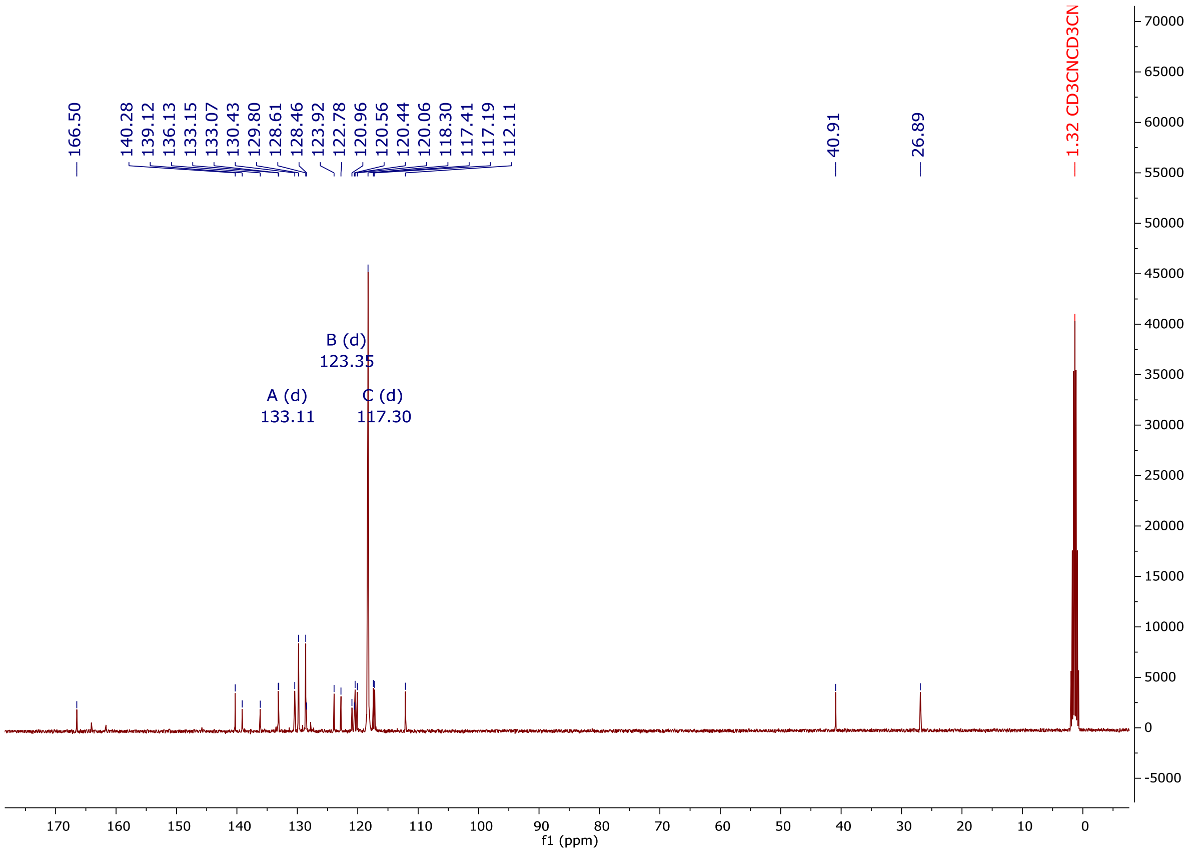

Figure S83. NMR of ^{13}C (101 MHz) in Acetonitrile-d$_3$ of compound **5w**

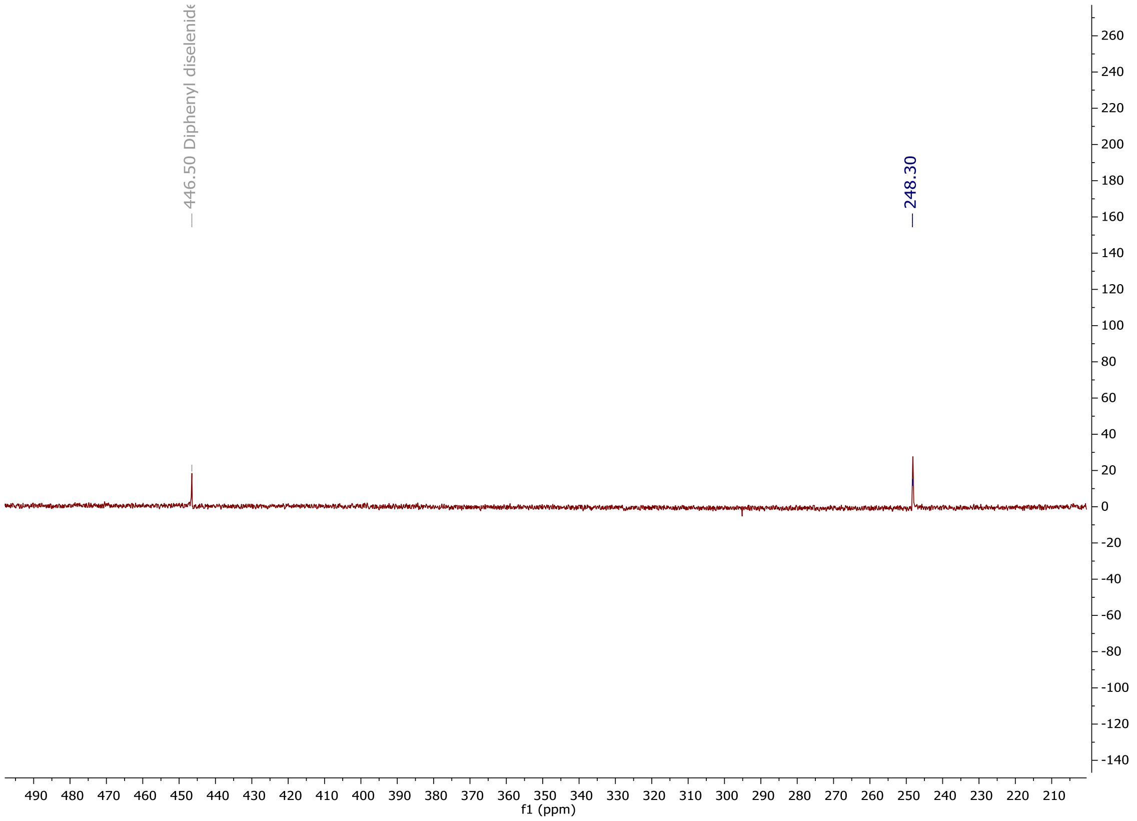

Figure S84. NMR of ^{77}Se (76 MHz) in Acetonitrile-d$_3$ of compound **5w**

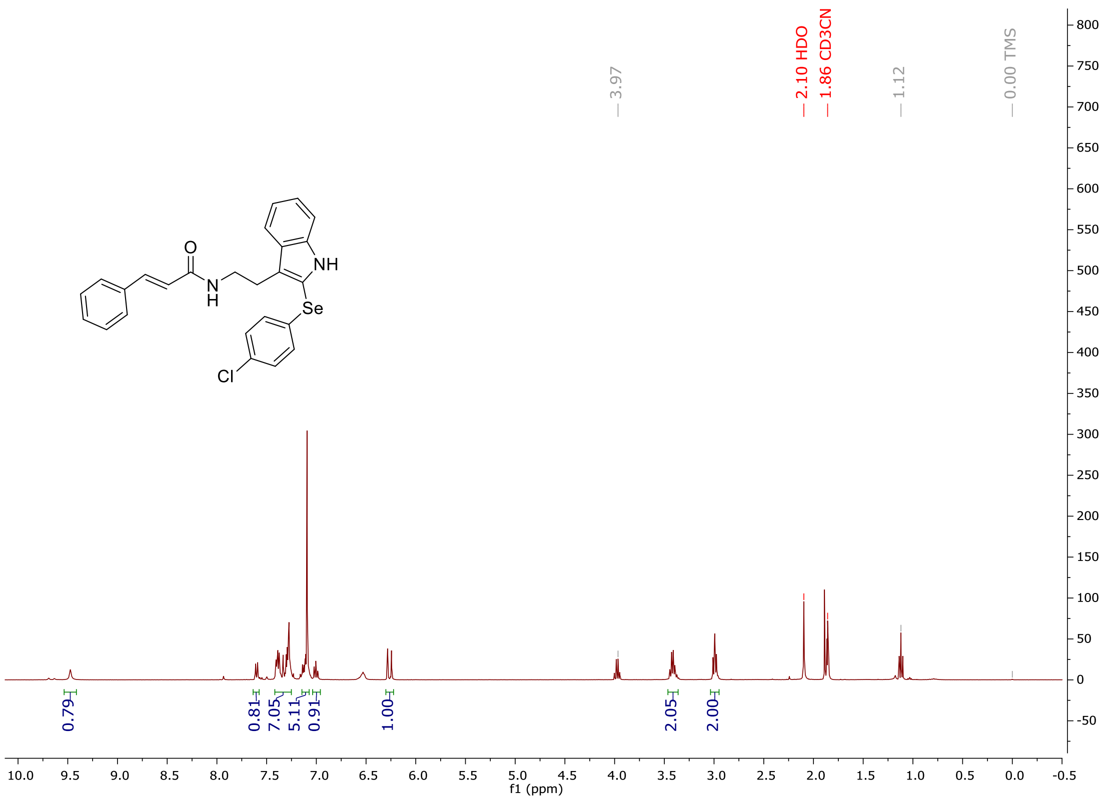

Figure S85. NMR of ^{1}H (400 MHz) in Acetonitrile-d_3 of compound **5x**

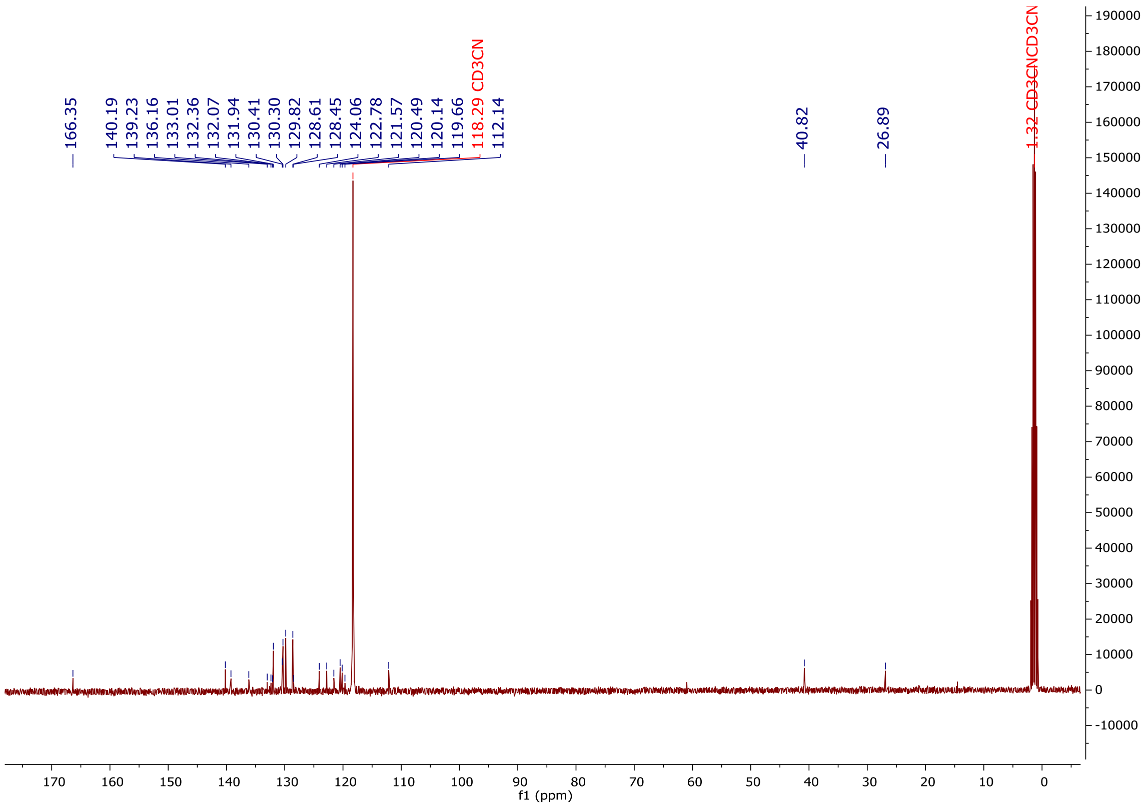

Figure S86. NMR of ^{13}C (101 MHz) in Acetonitrile-d_3 of compound **5x**

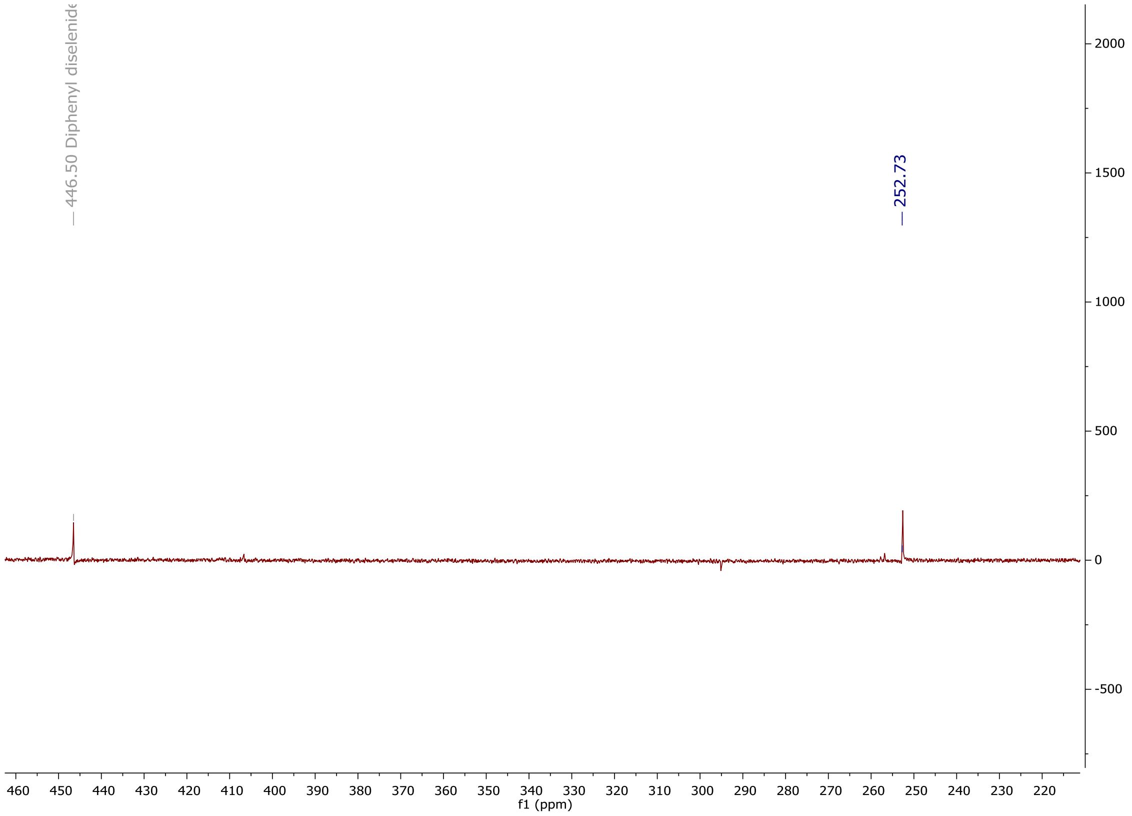

Figure S87. NMR of ^{77}Se (76 MHz) in Acetonitrile-d$_3$ of compound **5x**

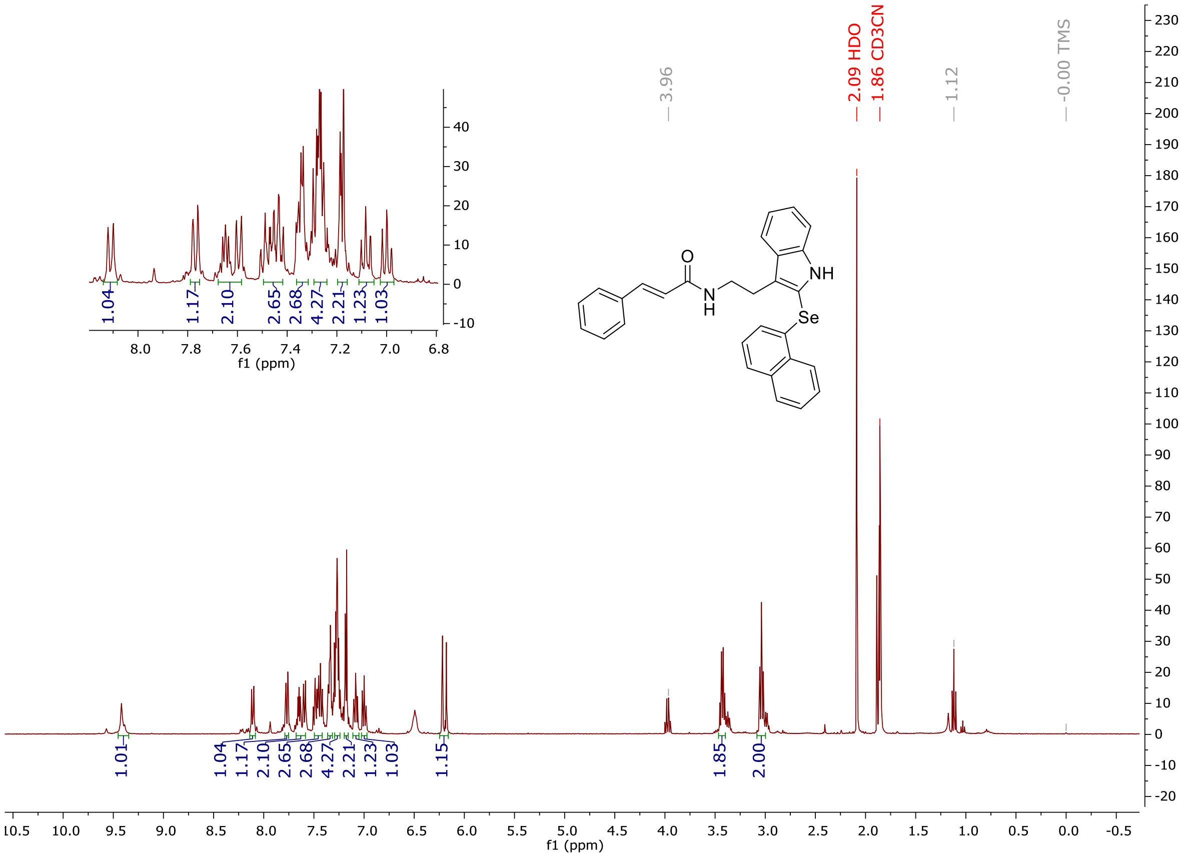

Figure S88. NMR of ^{1}H (400 MHz) in Acetonitrile-d$_3$ of compound **5y**

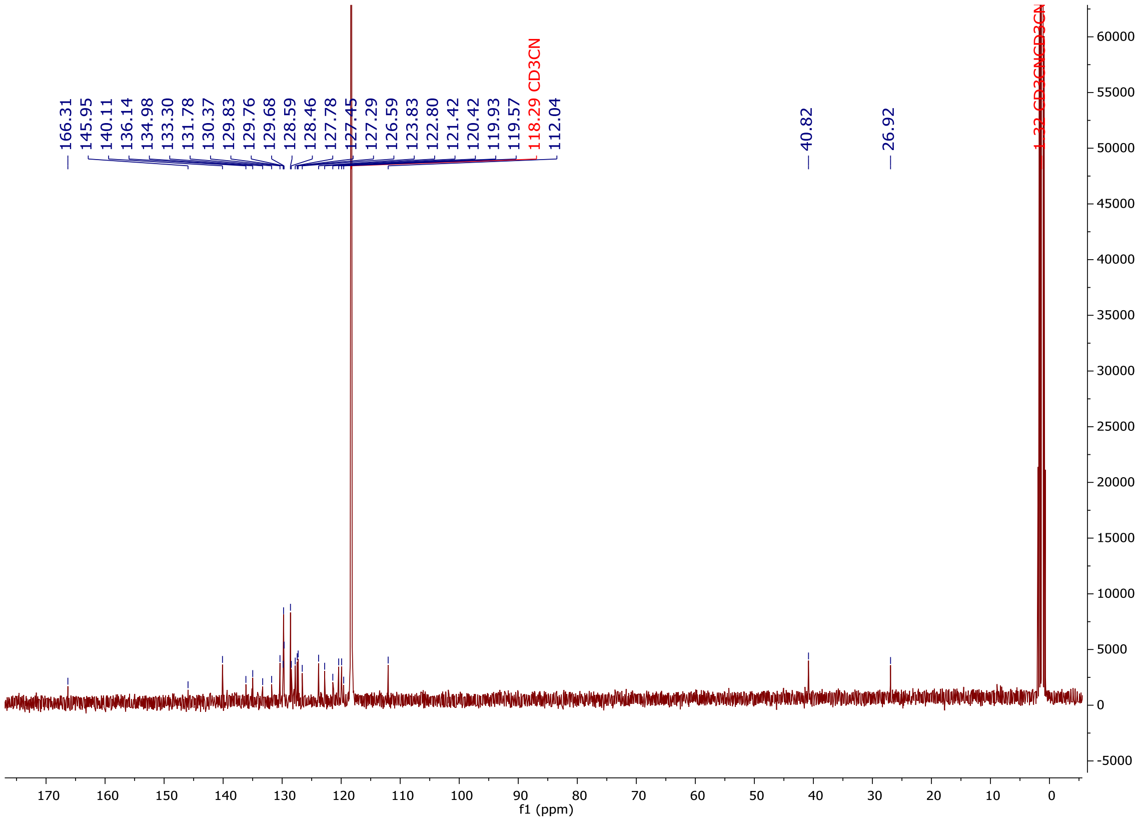

Figure S89. NMR of ^{13}C (101 MHz) in Acetonitrile-d_3 of compound **5y**

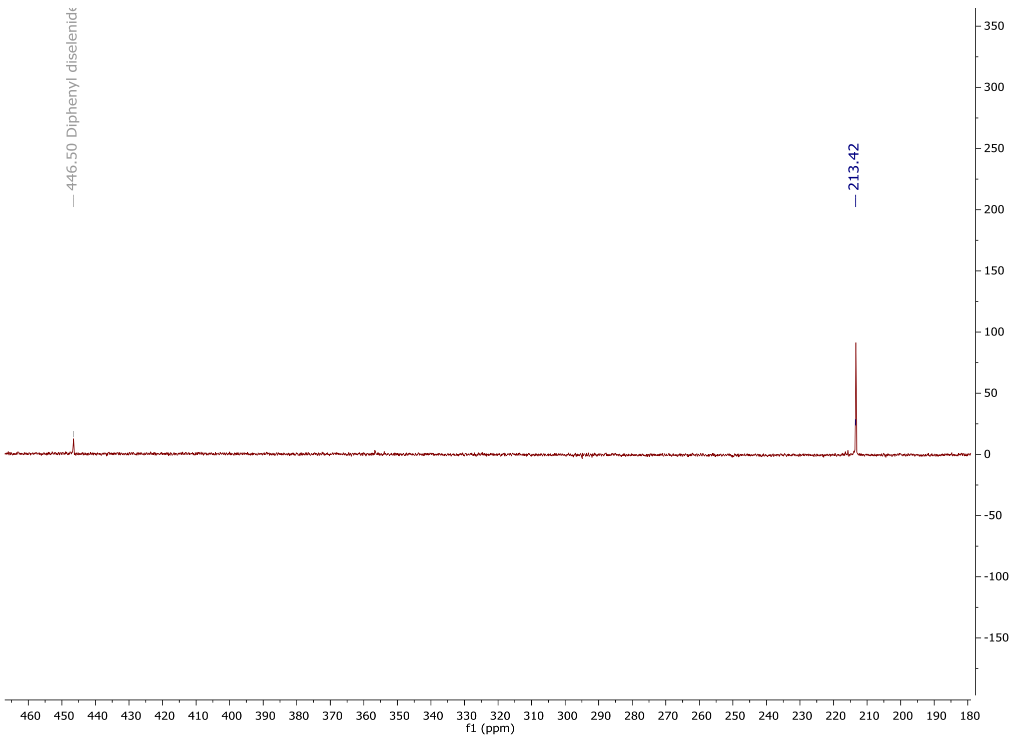

Figure S90. NMR of ^{77}Se (76 MHz) in Acetonitrile-d_3 of compound **5y**

8. References

(1) Jones, G.; Willett, P.; Glen, R. C.; Leach, A. R.; Taylor, R. Development and validation of a genetic algorithm for flexible docking. *J. Mol. Biol.* **1997**, *267*, 727. https://doi.org/10.1006/jmbi.1996.0897.

(2) Dassault Systèmes BIOVIA. *Discovery Studio Modeling Environment*; Dassault Systèmes: San Diego, CA, 2022.

(3) Schrödinger, L. *Schrödinger Release 2022-3: Prime*; Schrödinger, LLC: New York, NY, 2022.

(4) Genheden, S.; Ryde, U. The MM/PBSA and MM/GBSA methods to estimate ligand-binding affinities. *Expert Opin. Drug Discov.* **2015**, *10*, 449. https://doi.org/10.1517/17460441.2015.1032936.

(5) Daina, A.; Michielin, O.; Zoete, V. SwissADME: A free web tool to evaluate pharmacokinetics, drug-likeness and medicinal chemistry friendliness of small molecules. *Sci. Rep.* **2017**, *7*, 42717. https://doi.org/10.1038/srep42717.

(6) Pires, D. E. V.; Blundell, T. L.; Ascher, D. B. pkCSM: Predicting small-molecule pharmacokinetic and toxicity properties using graph-based signatures. *J. Med. Chem.* **2015**, *58*, 4066. https://doi.org/10.1021/acs.jmedchem.5b00104.

(7) Banerjee, P.; Eckert, A. O.; Schrey, A. K.; Preissner, R. ProTox-II: A webserver for the prediction of toxicity of chemicals. *Nucleic Acids Res.* **2018**, *46*, W257. https://doi.org/10.1093/nar/gky318.

(8) Baell, J. B.; Holloway, G. A. New substructure filters for removal of pan assay interference compounds (PAINS) from screening libraries and for their exclusion in bioassays. *J. Med. Chem.* **2010**, *53*, 2719. https://doi.org/10.1021/jm901137j.

(9) Huggins, D. J.; Venkitaraman, A. R.; Spring, D. R. Rational methods for the selection of diverse screening compounds. *ACS Chem. Biol.* **2011**, *6*, 208. https://doi.org/10.1021/cb100420r.

(10) Wager, T. T.; Hou, X.; Verhoest, P. R.; Villalobos, A. Moving beyond rules: The development of a central nervous system multiparameter optimization (CNS MPO) approach to enable alignment of druglike properties. *ACS Chem. Neurosci.* **2010**, *1*, 435. https://doi.org/10.1021/cn100008c.

(11) Bousfield, T. W.; Pearce, K. P. R.; Nyamini, S. B.; Angelis-Dimakis, A.; Camp, J. E. Synthesis of amides from acid chlorides and amines in the bio-based solvent CyreneTM. *Green Chem.* **2019**, *21*, 3675. https://doi.org/10.1039/c9gc01180c.

(12) Reich, H. J.; Cohen, M. L.; Clark, P. S. Reagents for synthesis of organoselenium compounds: Diphenyl diselenide and benzeneselenenyl chloride. *Org. Synth.* **1979**, *59*, 141.

(13) Wu, G.; Lv, T.; Mo, W.; Yang, X.; Gao, Y.; Chen, H. One-pot synthesis of tricyclo-1,4-benzoxazines via visible-light photoredox catalysis in continuous flow. *Tetrahedron Lett.* **2017**, *58*, 1395. https://doi.org/10.1016/j.tetlet.2017.02.068.
